# Kidney Function Following COVID-19 in Children and Adolescents

**DOI:** 10.1001/jamanetworkopen.2025.4129

**Published:** 2025-04-11

**Authors:** Lu Li, Ting Zhou, Yiwen Lu, Jiajie Chen, Yuqing Lei, Qiong Wu, Jonathan Arnold, Michael J. Becich, Yuriy Bisyuk, Saul Blecker, Elizabeth Chrischilles, Dimitri A. Christakis, Carol Reynolds Geary, Ravi Jhaveri, Leslie Lenert, Mei Liu, Parsa Mirhaji, Hiroki Morizono, Abu S. M. Mosa, Ali Mirza Onder, Ruby Patel, William E. Smoyer, Bradley W. Taylor, David A. Williams, Bradley P. Dixon, Joseph T. Flynn, Caroline Gluck, Lyndsay A. Harshman, Mark M. Mitsnefes, Zubin J. Modi, Cynthia G. Pan, Hiren P. Patel, Priya S. Verghese, Christopher B. Forrest, Michelle R. Denburg, Yong Chen

**Affiliations:** 1 The Center for Health AI and Synthesis of Evidence, University of Pennsylvania, Philadelphia; 2 The Graduate Group in Applied Mathematics and Computational Science, School of Arts and Sciences, University of Pennsylvania, Philadelphia; 3 Department of Biostatistics and Health Data Science, University of Pittsburgh, Pittsburgh, Pennsylvania; 4 Division of General Internal Medicine, University of Pittsburgh School of Medicine, Pittsburgh, Pennsylvania; 5 Department of Biomedical Informatics, University of Pittsburgh School of Medicine, Pittsburgh, Pennsylvania; 6 Office of Research, University Medical Center New Orleans, New Orleans, Louisiana; 7 Department of Population Health, NYU (New York University) Grossman School of Medicine, New York, New York; 8 Department of Epidemiology, College of Public Health, University of Iowa, Iowa City; 9 Center for Child Health, Behavior and Development, Seattle Children’s Research Institute, Seattle, Washington; 10 Department of Pathology, Microbiology, and Immunology, University of Nebraska Medical Center, Omaha; 11 Division of Infectious Diseases, Ann & Robert H. Lurie Children’s Hospital of Chicago, Chicago, Illinois; 12 Biomedical Informatics Center, Medical University of South Carolina, Charleston; 13 Department of Health Outcomes and Biomedical Informatics, University of Florida, College of Medicine, Gainesville; 14 Albert Einstein College of Medicine, Bronx, New York; 15 Center for Genetic Medicine Research, Children’s National Hospital, Washington, DC; 16 Department of Biomedical Informatics, Biostatistics and Medical Epidemiology, University of Missouri School of Medicine, Columbia; 17 Division of Pediatric Nephrology, Nemours Children’s Hospital, Wilmington, Delaware; 18 Division of Pediatric Nephrology, Stanford Medicine Children’s Health, Palo Alto, California; 19 Center for Clinical and Translational Research, Nationwide Children’s Hospital, Department of Pediatrics, The Ohio State University, Columbus; 20 Clinical and Translational Science Institute, The Medical College of Wisconsin, Milwaukee; 21 Department of Anesthesiology, University of Michigan, Ann Arbor; 22 Renal Section, Department of Pediatrics, University of Colorado School of Medicine, Aurora; 23 Department of Pediatrics, University of Washington, Seattle; 24 Division of Nephrology, Seattle Children’s Hospital, Seattle, Washington; 25 Division of Pediatric Nephrology, Nemours Children’s Health, Wilmington, Delaware; 26 Stead Family Department of Pediatrics, University of Iowa, Iowa City; 27 Division of Pediatric Nephrology, Department of Pediatrics, Cincinnati Children’s Hospital Medical Center and University of Cincinnati, Cincinnati, Ohio; 28 Susan B. Meister Child Health Evaluation and Research Center, Department of Pediatrics, University of Michigan, Ann Arbor; 29 Division of Pediatric Nephrology, Department of Pediatrics, University of Michigan, Ann Arbor; 30 Department of Pediatrics, Section of Nephrology, Medical College of Wisconsin, Milwaukee; 31 Section of Nephrology and Hypertension, Nationwide Children’s Hospital, Columbus, Ohio; 32 Department of Pediatrics, Ohio State University College of Medicine; 33 Department of Pediatrics, Division of Nephrology, Ann & Robert H Lurie Children’s Hospital, Chicago, Illinois; 34 Department of Pediatrics, Northwestern University, Feinberg School of Medicine, Chicago, Illinois; 35 Applied Clinical Research Center, Department of Pediatrics, Children’s Hospital of Philadelphia, Philadelphia, Pennsylvania; 36 Division of Pediatric Nephrology, Children’s Hospital of Philadelphia, Philadelphia, Pennsylvania; 37 Department of Pediatrics, Perelman School of Medicine at the University of Pennsylvania, Philadelphia; 38 Department of Biostatistics, Epidemiology and Informatics, Perelman School of Medicine at the University of Pennsylvania, Philadelphia

## Abstract

**Importance:**

It remains unclear whether children and adolescents with SARS-CoV-2 infection are at heightened risk for long-term kidney complications.

**Objective:**

To investigate whether SARS-CoV-2 infection is associated with an increased risk of postacute kidney outcomes among pediatric patients, including those with preexisting kidney disease or acute kidney injury (AKI).

**Design, Setting, and Participants:**

This retrospective cohort study used data from 19 health institutions in the National Institutes of Health Researching COVID to Enhance Recovery (RECOVER) initiative from March 1, 2020, to May 1, 2023 (follow-up ≤2 years completed December 1, 2024; index date cutoff, December 1, 2022). Participants included children and adolescents (aged <21 years) with at least 1 baseline visit (24 months to 7 days before the index date) and at least 1 follow-up visit (28 to 179 days after the index date).

**Exposures:**

SARS-CoV-2 infection, determined by positive laboratory test results (polymerase chain reaction, antigen, or serologic) or relevant clinical diagnoses. A comparison group included children with documented negative test results and no history of SARS-CoV-2 infection.

**Main Outcomes and Measures:**

Outcomes included new-onset chronic kidney disease (CKD) stage 2 or higher or CKD stage 3 or higher among those without preexisting CKD; composite kidney events (≥50% decline in estimated glomerular filtration rate [eGFR], eGFR ≤15 mL/min/1.73 m^2^, dialysis, transplant, or end-stage kidney disease diagnosis), and at least 30%, 40%, or 50% eGFR decline among those with preexisting CKD or acute-phase AKI. Hazard ratios (HRs) were estimated using Cox proportional hazards regression models with propensity score stratification.

**Results:**

Among 1 900 146 pediatric patients (487 378 with and 1 412 768 without COVID-19), 969 937 (51.0%) were male, the mean (SD) age was 8.2 (6.2) years, and a range of comorbidities was represented. SARS-CoV-2 infection was associated with higher risk of new-onset CKD stage 2 or higher (HR, 1.17; 95% CI, 1.12-1.22) and CKD stage 3 or higher (HR, 1.35; 95% CI, 1.13-1.62). In those with preexisting CKD, COVID-19 was associated with an increased risk of composite kidney events (HR, 1.15; 95% CI, 1.04-1.27) at 28 to 179 days. Children with acute-phase AKI had elevated HRs (1.29; 95% CI, 1.21-1.38) at 90 to 179 days for composite outcomes.

**Conclusions and Relevance:**

In this large US cohort study of children and adolescents, SARS-CoV-2 infection was associated with a higher risk of adverse postacute kidney outcomes, particularly among those with preexisting CKD or AKI, suggesting the need for vigilant long-term monitoring.

## Key Points

**Question:**

Is SARS-CoV-2 infection associated with an increased risk of adverse kidney outcomes in children and adolescents, particularly among those with preexisting kidney disease or acute kidney injury during the acute phase?

**Findings:**

In this cohort study including 1 900 146 pediatric patients with and without COVID-19, infection was associated with a higher incidence of new-onset chronic kidney disease and worsening kidney function, especially among those with preexisting kidney disease or acute kidney injury.

**Meaning:**

These findings underscore the need for vigilant monitoring and management of kidney health in pediatric patients following SARS-CoV-2 infection.

## Teaser

This cohort study investigates the association of SARS-CoV-2 infection with risk of postacute adverse kidney outcomes among children and adolescents.

## Introduction

Research has shed light on the postacute sequelae of SARS-CoV-2 (PASC) infection, commonly referred to as long COVID or post–COVID-19 condition.^1,2,3^ The National Institutes of Health and the Centers for Disease Control and Prevention define PASC as new, returning, or ongoing health problems present at least 4 weeks after infection.^4^ While initially recognized predominantly among adults, the emergence of PASC has raised questions about its outcomes in the pediatric population. In the US, PASC has affected 5% to 10% of children, which is comparable to the incidence among adults (6.9%).^5,6^ However, there are important differences in the presentation and outcomes of acute SARS-CoV-2 infection between children and adults. Children can have different symptoms compared with adults and tend to have a milder disease course, with a lower risk of hospitalization and death, particularly among children without preexisting conditions.^7,8,9^ Given these differences in acute infection, as well as the differences in prevalence between children and adults, the characteristics of PASC require further study in children.

One notable study by Bowe et al^10^ used national health databases from the US Department of Veterans Affairs and reported that after the first 30 days post infection, individuals with COVID-19 exhibited increased risks of several adverse kidney outcomes, including acute kidney injury (AKI); declines in estimated glomerular filtration rate (eGFR) of at least 30%, at least 40%, and at least 50% from baseline; kidney replacement therapy; and major adverse kidney events, defined as a composite of at least 50% eGFR decline, kidney replacement therapy, or all-cause mortality.^10^ Conversely, studies focusing on children and adolescents have been limited by short duration of follow-up, sample size (mostly <100 patients), and a narrow selection of outcomes: AKI, mortality, and multisystem inflammatory syndrome in children.^11,12,13,14,15^

This report aims to bridge these gaps in knowledge by examining a set of postacute kidney outcomes among children and adolescents after SARS-CoV-2 infection. To assess the association of SARS-CoV-2 infection with preexisting kidney injury in pediatric patients, we stratified all analyses on the presence of preexisting chronic kidney disease (CKD) and AKI experienced during the acute phase of the infection. Our objective was to assess the risks of postacute kidney manifestations of SARS-CoV-2 infection in the pediatric population, in the hope of informing future care strategies for those affected by PASC. Postacute kidney manifestations of PASC may include persistent or progressive kidney dysfunction, which aligns with our focus on evaluating postacute kidney outcomes following COVID-19. We hypothesized that children and adolescents with COVID-19 would exhibit higher risks of adverse kidney outcomes compared with those without COVID-19, particularly among those with preexisting CKD or who developed AKI during the acute phase.

## Methods

### Data Sources

This study is part of the National Institutes of Health Researching COVID to Enhance Recovery (RECOVER) Initiative, which aims to learn about the long-term effects of COVID-19. This study was approved by the University of Pennsylvania’s institutional review board, with a waiver of informed consent for the use of deidentified data. It follows the Strengthening the Reporting of Observational Studies in Epidemiology (STROBE) reporting guideline for cohort studies. We included 19 health institutions listed in eAppendix 1 in Supplement 1.

### Cohort Construction

**Figure 1.**
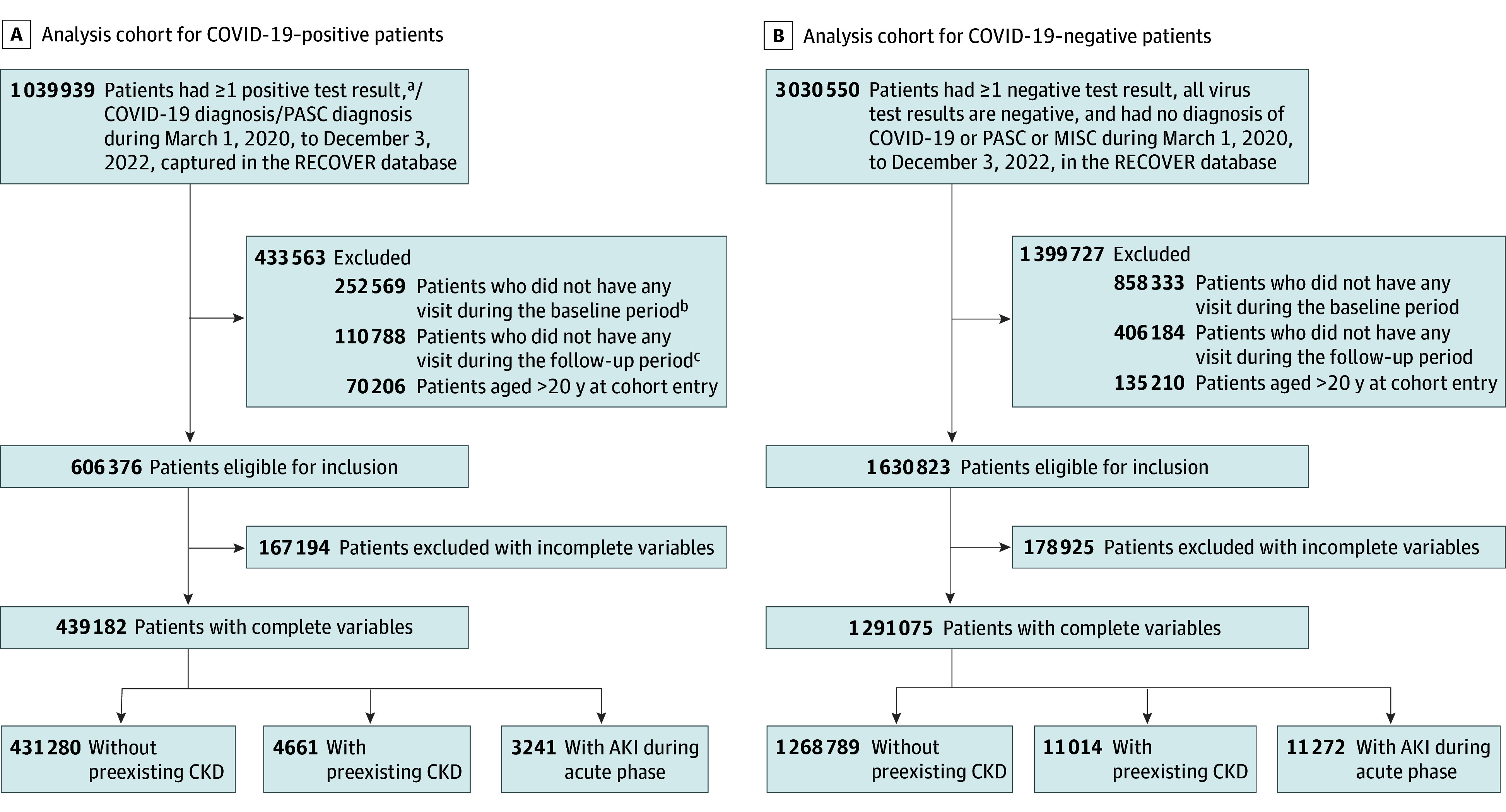
Flowchart of Study Cohort Selection of Patients for COVID-19–Positive and COVID-19–Negative Groups AKI indicates acute kidney injury; CKD, chronic kidney disease; MISC, multisystem inflammatory syndrome in children; PASC, postacute sequelae of SARS-CoV-2; and RECOVER, Researching COVID to Enhance Recovery. ^a^Includes polymerase chain reaction, antigen, and serologic testing. ^b^Indicates 24 months to 7 days before the index date. ^c^Indicates 28 to 179 days after the index date.

We conducted a retrospective study spanning from March 1, 2020, to May 1, 2023, with a cohort entry date cutoff of December 1, 2022 (179 days before the end of the study period). The index date for these patients was defined as the first indication of SARS-CoV-2 infection (ie, the date of the first positive COVID-19 test result or diagnosis). We included patients younger than 21 years who had at least 1 visit within 24 months to 7 days prior to the index date (defined as the baseline period) and at least 1 visit within 28 to 179 days after the index date (defined as the follow-up period). For COVID-19–positive patients, we included those who had positive findings of polymerase chain reaction, serologic, or antigen tests, diagnosis of COVID-19, or diagnosis of PASC. For COVID-19–negative patients, we included patients who had no documented SARS-CoV-2 infection and had at least 1 negative COVID-19 test result within the same study period. A random negative test was chosen as the index date for COVID-19–negative patients. Details of study variables can be found in eAppendix 2 in Supplement 1. The selection of participants for the COVID-19–positive and COVID-19–negative patient groups is summarized in Figure 1.

Patients with either a diagnosis of multisystem inflammatory syndrome in children or evidence of end-stage kidney disease (ESKD) prior to 28 days after the index date were excluded, where ESKD was defined as having kidney dialysis, kidney transplant, diagnosis of ESKD by *International Statistical Classification of Diseases, Tenth Revision* codes, or any eGFR of 15 mL/min/1.73 m^2^ or less. This exclusion facilitates a more focused analysis on the direct impacts of SARS-CoV-2 in kidney health in the pediatric population.^15,16^ We conduct a sensitivity analysis in which we do not exclude patients with kidney dialysis during the acute phase for the AKI group (eAppendix 11 in Supplement 1), and a sensitivity analysis for CDK subgroup without excluding patients with ESKD during baseline (eAppendix 13 in Supplement 1).

### Kidney Disease Status and Outcome Definitions

Onset date for CKD stage 2 and higher was defined as the date of the second eGFR of the earliest pair of eGFR values less than 90 mL/min/1.73 m^2^, separated by at least 90 days, without an intervening eGFR 90 mL/min/1.73 m^2^ or greater. Onset date for CKD stage 3 and higher was defined as the date of the second eGFR of the earliest pair of eGFR values less than 60 mL/min/1.73 m^2^, separated by at least 90 days, without an intervening eGFR 90 mL/min/1.73 m^2^ or greater. Additionally, we included 2 variations to the above definitions by either requiring the eGFR values to never return back to greater than 90 mL/min/1.73 m^2^ (for CKD stage 2 or higher and CKD stage 3 or higher) or 60 mL/min/1.73 m^2^ (for CKD stage 3 or higher), or requiring most eGFR values never return to above 90 or 60 mL/min/1.73 m^2^, where *most* is defined as greater than 50%. The adjusted hazard ratios (HRs) for outcomes using varied definitions of CKD stage 2 or higher and CKD stage 3 or higher are given in eAppendix 15 in Supplement 1. Note that for the definition of CKD outcomes, we require the date of the first eGFR of the pair to be 28 days after the infection. AKI was defined as having a serum creatinine measurement within 28 days after the index date that was 0.3 mg/dL (to convert to μmol/L, multiply by 88.4) or 50% greater than baseline, where baseline was defined as the closest value within 90 days before index date, and if this was not available, established age and sex reference values were used.^17^ We performed a sensitivity analysis using the lowest creatinine measurement within 90 days before the index date as the baseline (eAppendix 12 in Supplement 1). We used the U25 serum creatinine–based equations^18^ to estimate eGFR, due to limited availability of serum cystatin C data. Age was computed at the date of the serum creatinine measurement and the closest height within 180 days of the date of the serum creatinine measurement was used.

For patients without CKD stage 2 or higher in the 24 months before the SARS-CoV-2 infection, we examined the outcomes of new onset of CKD stage 2 or higher or CKD stage 3 or higher within 28 to 729 days after SARS-CoV-2 infection. For patients with preexisting CKD in the 24 months before the SARS-CoV-2 infection, we examined a composite outcome including therapeutic interventions (long-term kidney dialysis, kidney transplant, eGFR decline of at least 50%, ESKD diagnosis, or eGFR 15 mL/min/1.73 m^2^ or less within 28 to 179 days and within 180 to 729 days after SARS-CoV-2 infection). We also looked at eGFR decline of at least 30%, 40%, and 50% from within 28 to 179 days and within 180 to 729 days after SARS-CoV-2 infection. For patients with AKI during the acute phase (within 28 days) of SARS-CoV-2 infection, we examined the same sets of outcomes as with patients in the other 2 strata and focused on ascertainment of these outcomes within 90 to 179 days and within 180 to 729 days.

### Covariates

We examined a comprehensive set of 138 patient characteristics as potential confounders. These included patient age at cohort entry; sex (female or male); electronic health record (EHR)–derived race and ethnicity (combined Asian American and Native Hawaiian or Other Pacific Islander, Hispanic, non-Hispanic Black, non-Hispanic White, multiracial, or other [including no information, refuse to answer, or unknown from EHR concept source values]); month and year of cohort entry (from March 1, 2020, to May 1, 2023); site index for cohort entry; obesity defined using the 95th percentile or greater of body mass index for age and sex; a chronic condition indicator as defined by the Pediatric Medical Complexity Algorithm^19^ (consisting of no chronic condition, noncomplex chronic condition, or complex chronic condition); a list of preexisting chronic conditions; the number of inpatient, outpatient, and emergency department visits; the number of unique medications or prescriptions (0, 1, 2, or ≥3) at baseline; the number of negative test results during the baseline period (grouped as 0, 1, 2, or ≥3); the number of vaccine doses before cohort entry date (0, 1, or ≥2); and interval since the last immunization date (no vaccine, <4 months, or ≥4 months). Race and ethnicity were included in the analysis because they are potential confounders in this study.

### Statistical Analysis

We calculated the incidence of postacute kidney outcomes in both COVID-19–positive and COVID-19–negative cohorts stratified by preexisting kidney health status. To quantify the risks of postacute kidney manifestations of SARS-CoV-2 infection, we used the HR as the comparative measure, derived from Cox proportional hazards regression models.

To eliminate potential measured confounders, we used a propensity score stratification technique with the covariates detailed in the Covariates section. To handle the large number of covariates, we used L1-penalized logistic regression. To mitigate aggregation bias and account for potential clustering due to site-specific variations, we stratified by propensity scores, incorporating site as a covariate in the propensity score model. After performing the stratification, we assessed the standardized mean difference between each covariate value for COVID-19–positive and COVID-19–negative patients, with a difference of 0.1 or less indicating an acceptable balance.^20,21^ The clinical equipoise before and after stratification and standardized mean difference of covariates before and after stratification for the primary analysis appear in eAppendix 3 in Supplement 1.

## Results

### Cohort Identification

Among 1 900 146 pediatric patients, a total of 487 378 with COVID-19 and 1 412 768 without COVID-19 in the RECOVER database were included to evaluate the risks of postacute kidney manifestations of SARS-CoV-2 infection. Of these patients, 930 209 (49.0%) were female and 969 937 (51.0%) were male; mean (SD) age was 8.2 (6.2) years. By race and ethnicity, 91 628 patients (4.8%) were Asian American or Native Hawaiian or Other Pacific Islander; 399 307 (21.0%), Hispanic; 328 002 (17.3%), non-Hispanic Black; 852 723 (44.9%), non-Hispanic White; 43 790 (2.3%), multiracial; and 184 696 (9.7%), other or unknown. Baseline comorbidities by COVID-19 status and preexisting kidney health status are presented in Table 1. Site information and detailed breakdown of age distribution are in eAppendix 14 in Supplement 1.

**Table 1.** Baseline Characteristics of Patients

Characteristic	Patient group, No. (%)^a^							
	COVID-19 negative				COVID-19 positive			
	No AKI or CKD (n = 1 383 464)	CKD (n = 14 999)	AKI (n = 14 305)	Overall (n = 1 412 768)	No AKI or CKD (n = 477 031)	CKD (n = 6483)	AKI (n = 3864)	Overall (n = 487 378)
Age at cohort entry, y								
Mean (SD)	7.9 (6.1)	12.8 (5.8)	8.6 (7.0)	8.0 (6.1)	8.7 (6.3)	13.6 (5.7)	10.4 (7.0)	8.8 (6.4)
Median (IQR)	7.0 (2.0-13.0)	15.0 (8.0-17.0)	8.0 (1.0-15.0)	7.0 (2.0-13.0)	8.0 (3.0-14.0)	16.0 (10.0-18.0)	12.0 (3.0-17.0)	9.0 (3.0-14.0)
Sex								
Female	671 071 (48.5)	8963 (59.8)	6477 (45.3)	686 511 (48.6)	237 969 (49.9)	4052 (62.5)	1677 (43.4)	243 698 (50.0)
Male	712 393 (51.5)	6036 (40.2)	7828 (54.7)	726 257 (51.4)	239 062 (50.1)	2431 (37.5)	2187 (56.6)	243 680 (50.0)
Race and ethnicity								
Asian American and Native Hawaiian or Other Pacific Islander	67 455 (4.9)	554 (3.7)	624 (4.4)	68 633 (4.9)	22 661 (4.8)	193 (3.0)	141 (3.6)	22 995 (4.7)
Hispanic	285 725 (20.7)	2234 (14.9)	2673 (18.7)	290 632 (20.6)	106 979 (22.4)	969 (14.9)	727 (18.8)	108 675 (22.3)
Non-Hispanic Black	234 968 (17.0)	3058 (20.4)	4045 (28.3)	242 071 (17.1)	83 295 (17.5)	1478 (22.8)	1158 (30.0)	85 931 (17.6)
Non-Hispanic White	625 621 (45.2)	8091 (53.9)	5725 (40.0)	639 437 (45.3)	208 246 (43.7)	3478 (53.6)	1562 (40.4)	213 286 (43.8)
Multiracial	33 778 (2.4)	252 (1.7)	164 (1.1)	34 194 (2.4)	9479 (2.0)	79 (1.2)	38 (1.0)	9596 (2.0)
Other or unknown^b^	135 917 (9.8)	810 (5.4)	1074 (7.5)	137 801 (9.8)	46 371 (9.7)	286 (4.4)	238 (6.2)	46 895 (9.6)
Cohort entry period								
March to May 2020	25 471 (1.8)	594 (4.0)	436 (3.0)	26 501 (1.9)	4778 (1.0)	96 (1.5)	114 (3.0)	4988 (1.0)
March to May 2021	137 964 (10.0)	1497 (10.0)	1534 (10.7)	140 995 (10.0)	28 730 (6.0)	448 (6.9)	280 (7.2)	29 458 (6.0)
March to May 2022	114 323 (8.3)	1110 (7.4)	1401 (9.8)	116 834 (8.3)	36 985 (7.8)	509 (7.9)	337 (8.7)	37 81 (7.8%)
June to August 2020	96 457 (7.0)	1731 (11.5)	938 (6.6)	99 126 (7.0)	17 368 (3.6)	248 (3.8)	169 (4.4)	17 785 (3.6)
June to August 2021	135 823 (9.8)	1431 (9.5)	1477 (10.3)	138 731 (9.8)	26 162 (5.5)	409 (6.3)	268 (6.9)	26 839 (5.5)
June to August 2022	83 688 (6.0)	958 (6.4)	1336 (9.3)	85 982 (6.1)	51 659 (10.8)	757 (11.7)	456 (11.8)	52 872 (10.8)
September to November 2020	140 935 (10.2)	1798 (12.0)	1506 (10.5)	144 239 (10.2)	32 030 (6.7)	408 (6.3)	224 (5.8)	32 662 (6.7)
September to November 2021	217 656 (15.7)	1645 (11.0)	1556 (10.9)	220 857 (15.6)	49 458 (10.4)	630 (9.7)	409 (10.6)	50 497 (10.4)
September to November 2022	132 831 (9.6)	1216 (8.1)	1393 (9.7)	135 440 (9.6)	26 934 (5.6)	422 (6.5)	272 (7.0)	27 628 (5.7)
December 2020 to February 2021	135 978 (9.8)	1673 (11.2)	1341 (9.4)	138 992 (9.8)	51 601 (10.8)	665 (10.3)	356 (9.2)	52 622 (10.8)
December 2021 to February 2022	162 338 (11.7)	1346 (9.0)	1387 (9.7)	165 071 (11.7)	151 326 (31.7)	1891 (29.2)	979 (25.3)	154 196 (31.6)
Obesity								
Absent	722 415 (52.2)	7439 (49.6)	7852 (54.9)	737 706 (52.2)	214 869 (45.0)	2723 (42.0)	1910 (49.4)	219 502 (45.0)
Present	497 849 (36.0)	7541 (50.3)	4836 (33.8)	510 226 (36.1)	206 568 (43.3)	3759 (58.0)	1567 (40.6)	211 894 (43.5)
Unknown	163 200 (11.8)	19 (0.1)	1617 (11.3)	164 836 (11.7)	55 594 (11.7)	1 (0.02)	387 (10.0)	55 982 (11.5)
Chronic disease status								
No chronic condition	1 006 146 (72.7)	2840 (18.9)	6272 (43.8)	1 015 258 (71.9)	351 288 (73.6)	1283 (19.8)	1641 (42.5)	354 212 (72.7)
Noncomplex chronic condition	220 119 (15.9)	3280 (21.9)	2400 (16.8)	225 799 (16.0)	75 835 (15.9)	1403 (21.6)	585 (15.1)	77 823 (16.0)
Complex chronic condition comorbidities	157 199 (11.4)	8879 (59.2)	5633 (39.4)	171 711 (12.2)	49 908 (10.5)	3797 (58.6)	1638 (42.4)	55 343 (11.4)
No. of tests								
0	1 049 258 (75.8)	9026 (60.2)	9148 (63.9)	1 067 432 (75.6)	291 958 (61.2)	2415 (37.3)	1916 (49.6)	296 289 (60.8)
1	218 747 (15.8)	2818 (18.8)	2534 (17.7)	224 099 (15.9)	101 336 (21.2)	1459 (22.5)	709 (18.3)	103 504 (21.2)
2	66 084 (4.8)	1316 (8.8)	1059 (7.4)	68 459 (4.8)	41 027 (8.6)	844 (13.0)	360 (9.3)	42 231 (8.7)
≥3	49 375 (3.6)	1839 (12.3)	1564 (10.9)	52 778 (3.7)	42 710 (9.0)	1765 (27.2)	879 (22.7)	45 354 (9.3)
No. of vaccine doses								
0	1 248 073 (90.2)	12 690 (84.6)	13 165 (92.0)	1 273 928 (90.2)	416 466 (87.3)	5189 (80.0)	3399 (88.0)	425 054 (87.2)
1	25 661 (1.9)	417 (2.8)	260 (1.8)	26 338 (1.9)	12 185 (2.6)	288 (4.4)	135 (3.5)	12 608 (2.6)
≥2	109 730 (7.9)	1892 (12.6)	880 (6.2)	112 502 (8.0)	48 380 (10.1)	1006 (15.5)	330 (8.5)	49 716 (10.2)
No. of drugs								
0	379 837 (27.5)	355 (2.4)	2029 (14.2)	382 221 (27.1)	117 622 (24.7)	124 (1.9)	529 (13.7)	118 275 (24.3)
1	173 275 (12.5)	393 (2.6)	1040 (7.3)	174 708 (12.4)	59 593 (12.5)	182 (2.8)	258 (6.7)	60 033 (12.3)
2	136 222 (9.8)	490 (3.3)	898 (6.3)	137 610 (9.7)	49 144 (10.3)	181 (2.8)	228 (5.9)	49 553 (10.2)
≥3	694 130 (50.2)	13 761 (91.7)	10 338 (72.3)	718 229 (50.8)	250 672 (52.5)	5996 (92.5)	2849 (73.7)	259 517 (53.2)

Abbreviations: AKI, acute kidney injury; CKD, chronic kidney disease.

^a^ AKI indicates occurring during acute phase of COVID-19 infection; CKD, stage 2 or higher.

^b^ Includes no information, refused to answer, or unknown from electronic health record concept source values.

### Incidence and Risk of Postacute Kidney Outcomes 

Table 2 presents the incidence of individual and composite postacute kidney outcomes in the COVID-19–positive cohort compared with the COVID-19–negative cohort, stratified by preexisting kidney health status. Notably, for kidney outcomes within the postacute phase, the incidence among COVID-19–positive patients was higher than that among COVID-19–negative patients for all outcomes across all 3 strata.

**Table 2.** Rate of Individual and Composite Kidney Outcomes

Outcome by preexisting kidney health status^a^	Rate, person-years, No. (%) of total follow-up time	
	COVID-19 positive	COVID-19 negative
AKI		
Composite (days 90-179)	206 (11.03)	477 (6.86)
eGFR decline of ≥50% (days 90-179)	88 (4.67)	191 (2.73)
eGFR decline of ≥40% (days 90-179)	129 (6.87)	312 (4.47)
eGFR decline of ≥30% (days 90-179)	203 (10.87)	477 (6.86)
Composite (days 180-729)	370 (6.74)	796 (3.66)
eGFR decline of ≥50% (days 180-729)	162 (2.88)	308 (1.39)
eGFR decline of ≥40% (days 180-729)	256 (4.60)	516 (2.35)
eGFR decline of ≥30% (days 180-729)	365 (6.64)	796 (3.66)
CKD stage 2 or higher (days 28-729)	194 (3.48)	505 (2.31)
CKD stage 2 or higher (majority not returned to 90 mL/min/1.73 m^2^) (days 28-729)	175 (3.13)	442 (2.02)
CKD stage 2 or higher (not returned to 90 mL/min/1.73 m^2^) (days 28-729)	159 (2.83)	399 (1.82)
CKD stage 3 or higher (days 28-729)	62 (1.09)	117 (0.53)
CKD stage 3 or higher (majority not returned to 90 mL/min/1.73 m^2^) (days 28-729)	53 (0.93)	93 (0.42)
CKD stage 3 or higher (not returned to 90 mL/min/1.73 m^2^) (days 28-729)	59 (1.04)	104 (0.47)
CKD stage 3 or higher (not returned to 60 mL/min/1.73 m^2^) (days 28-729)	50 (0.88)	87 (0.39)
CKD		
Composite outcome (days 28-729)	905 (30.27)	1746 (24.94)
eGFR decline of ≥50% (days 28-729)	191 (6.10)	405 (5.58)
eGFR decline of ≥40% (days 28-729)	484 (15.77)	957 (13.40)
eGFR decline of ≥30% (days 28-729)	894 (30.02)	1746 (25.07)
Composite outcome (days 180-729)	1167 (13.16)	2516 (11.04)
eGFR decline of ≥50% (days 180-729)	246 (2.60)	575 (2.38)
eGFR decline of ≥40% (days 180-729)	615 (6.69)	1345 (5.69)
eGFR decline of ≥30% (days 180-729)	1159 (13.06)	2516 (11.04)
No AKI or CKD		
CKD stage 2 or higher (days 28-729)	1569 (0.22)	3900 (0.18)
CKD stage 2 or higher (majority not returned to ≥90 mL/min/1.73 m^2^) (days 28-729)	1354 (0.19)	3265 (0.15)
CKD stage 2 or higher (not returned to 90 mL/min/1.73 m^2^) (days 28-729)	1250 (0.18)	2909 (0.13)
CKD stage 3 or higher (days 28-729)	132 (0.02)	309 (0.01)
CKD stage 3 or higher (majority not returned to ≥90 mL/min/1.73 m^2^) (days 28-729)	98 (0.01)	224 (0.01)
CKD stage 3 or higher (not returned to 90 mL/min/1.73 m^2^) (days 28-729)	111 (0.02)	271 (0.01)
CKD stage 3 or higher (not returned to 60 mL/min/1.73 m^2^) (days 28-729)	88 (0.01)	200 (0.01)

Abbreviations: AKI, acute kidney injury; CKD, chronic kidney disease.

^a^ AKI indicates occurring during acute phase of COVID-19 infection.

**Figure 2.**
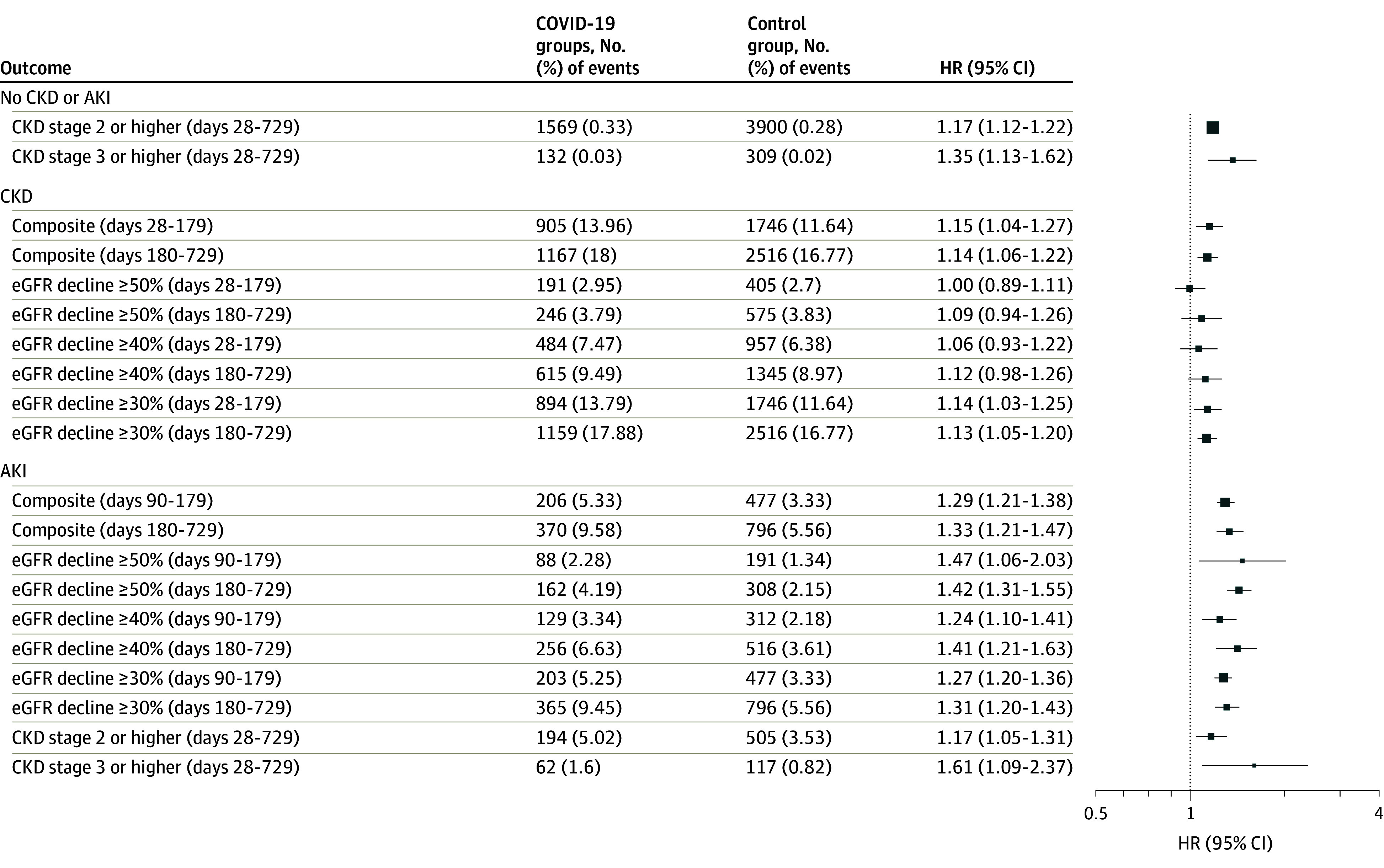
Adjusted Hazard Ratios (HRs) for Kidney Outcomes in COVID-19–Positive vs –Negative Patients by Phase The kidney outcomes are defined for each subgroup of patients based on preexisting kidney function status (acute kidney injury [AKI] during acute phase of COVID-19 infection, chronic kidney disease [CKD] stage 2 or higher, and no AKI or CKD). eGFR indicates estimated glomerular filtration rate.

Figure 2 shows the risks of postacute kidney manifestations of SARS-CoV-2 infection, stratified by preexisting kidney health status. For patients without preexisting CKD, our findings indicate increased risks in several postacute kidney outcomes following SARS-CoV-2 infection. The risk of new-onset CKD stage 2 or higher between days 28 and 729 was increased with an HR of 1.17 (95% CI, 1.12-1.22), and the HR for CKD stage 2 or higher with eGFR never returning to 90 mL/min/1.73 m^2^ or greater was 1.20 (95% CI, 1.13-1.28). For the outcome of CKD stage 3 or higher during days 28 to 729, the HR was 1.35 (95% CI, 1.13-1.62); for CKD stage 3 or higher without return of eGFR to 60 mL/min/1.73 m^2^ or greater, the HR was 1.35 (95% CI, 1.15-1.59).

For patients with preexisting CKD, we observed increased risks of post–SARS-CoV-2 infection in several outcomes. Specifically, there was an increased risk of the composite kidney outcome between days 28 and 179, with an HR of 1.15 (95% CI, 1.04-1.27), as well as an HR of 1.14 (95% CI, 1.06-1.22) for days 180 to 729. Additionally, an increase in risk was found for an eGFR decline of at least 30% between days 28 and 179, with an HR of 1.14 (95% CI, 1.03-1.25); for an eGFR decline of at least 30% between days 180 and 729, the HR was 1.13 (95% CI, 1.05-1.20). These findings indicate a heightened risk for kidney function decline in individuals with preexisting CKD following COVID-19 infection.

For patients who experienced AKI during the acute phase of SARS-CoV-2 infection, the study results indicate increased risks during the postacute phases. The HR for the composite kidney outcome was 1.29 (95% CI, 1.21-1.38) for days 90 to 179 and 1.33 (95% CI, 1.21-1.47) for days 180 to 729. There was an increased risk for an eGFR decline of at least 50% in both the earlier postacute phase (HR, 1.47; 95% CI, 1.06-2.03) and the later postacute phase (HR, 1.42; 95% CI, 1.31-1.55). There was an increased risk for an eGFR decline of at least 40% in both the earlier postacute phase (HR, 1.24; 95% CI, 1.10-1.41) and the later postacute phase (HR 1.41; 95% CI, 1.21-1.63). Additionally, an increased risk was observed for an eGFR decline of at least 30% at an HR of 1.27 (95% CI, 1.20-1.36) for days 28 to 179 and remained high at 1.31 (95% CI, 1.20-1.43) for days 180 to 729. These findings highlight a sustained increased risk for adverse kidney outcomes in patients with AKI after COVID-19.

To address residual confounding, we conducted sensitivity analyses to stratify patients by age (eAppendix 10 in Supplement 1), sex (eAppendix 5 in Supplement 1), race and ethnicity (eAppendix 6 in Supplement 1), hospitalization status (eAppendix 8 in Supplement 1), obesity defined using 95th percentile or greater for age and sex (eAppendix 7 in Supplement 1), dominant variant periods (eAppendix 4 in Supplement 1), and COVID infection severity levels (eAppendix 9 in Supplement 1). Our findings remained consistent and robust.

## Discussion

### Principal Findings

In this population-based study, using a cohort of 1 900 146 patients younger than 21 years in the US, our results found an increase in the risk of various kidney outcomes associated with COVID-19 infection. This heightened risk includes a new onset of mild-to-moderate CKD during the postacute phase of the infection. For patients with preexisting CKD and patients who experienced AKI during the acute phase, we observed an increased risk of a composite outcome of at least 50% eGFR decline, eGFR of 15 mL/min/1.73 m^2^ or less, dialysis, kidney transplant, or ESKD diagnosis.

### Comparison With Other Studies

Our findings align with prior research indicating that COVID-19 infection elevates the risk of kidney outcomes.^7,11,12,13,14,22^ Many of these earlier studies were limited by insufficient sample sizes for precise estimations and restricted their risk assessments to specific outcomes, such as AKI, and time frames.^7,11,12,13,14^ Our results are also consistent with a study that used the US Department of Veterans Affairs national health care databases (n = 1 726 683) to examine the risk of adverse kidney outcomes in the postacute phase following infection.^10^ Bowe et al^10^ observed a significantly higher risk of adverse kidney outcomes among COVID-19–infected individuals, including eGFR decline of at least 30% (adjusted HR [AHR], 1.25; 95% CI, 1.14-1.37), at least 40% (AHR, 1.44; 95% CI, 1.37-1.51), and at least 50% (AHR, 1.62; 95% CI, 1.51-1.74) and ESKD (AHR, 2.96; 95% CI, 2.49-3.51). Our study uniquely focused on children and adolescents, extending the evaluation of risk into the chronic phase beyond the postacute period. We also included additional outcomes of new-onset CKD. In addition, we stratified the patients based on their preexisting kidney function status to examine the association of SARS-CoV-2 infection with adverse kidney outcomes.

### Interpretation

The precise mechanisms underlying the observed associations in our study remain unclear. One plausible explanation could be attributed to the direct impact of COVID-19 on the kidneys, evidenced by the persistence of SARS-CoV-2 in tissues and prolonged virus shedding.^23,24^ Concurrently, the chronic inflammation induced by COVID-19 infection might adversely affect hemodynamic stability, potentially leading to kidney injury.^25^ An alternative explanation may lie in the therapeutic interventions used to manage severe COVID-19 cases,^25^ as well as the broader economic and social conditions resulting from the pandemic.^26,27^ Further investigations are warranted to elucidate the intricate pathways through which these factors interplay, contributing to the observed associations in our study.

### Limitations

Our study has certain limitations. First, while we successfully achieved a balanced distribution of baseline covariates through propensity score stratification, the propensity scores were constructed using only the variables accessible within our study databases. Nevertheless, it is important to highlight that our findings remained consistent and robust through sensitivity analyses aimed at addressing residual confounding. Patients were stratified by age, sex, race and ethnicity, hospitalization status, obesity defined using 95th percentile or greater for age and sex, dominant variant periods, and COVID infection severity levels.

Second, misclassification might exist for both AKI and CKD outcomes. For AKI, if no baseline information was available, we could potentially classify someone with underlying CKD as having AKI. For long-term outcomes, we could potentially capture multiple episodes of AKI as CKD. However, our various definitions of CKD tried to mitigate this possibility.

A third potential limitation is that it is difficult to ascertain from this study whether the increased risk associated with COVID-19 infection in the group with preexisting CKD is inherently due to the COVID-19 infection itself or whether the increased risk reflects a more general risk that patients with preexisting CKD who become ill may see a negative impact on eGFR. However, the findings in the AKI group and the groups with no AKI or CKD raise the possibility that there is an inherent risk in the COVID-19 infection itself.

Fourth, patients with positive home testing results (ie, with antigen testing available in 2021 and 2022) that would not be accessible or visible in the EHR could inadvertently be incorporated into the COVID-19–negative control group. However, this would also serve to bias toward the null hypothesis and would only strength the conclusion of the increased risk of CKD from PASC. However, patients with hospital-based testing may have been more ill from COVID-19 than those patients with positive home testing results, and this could also be a potential bias or limitation of these data.

Last, future studies could adopt dynamic exposure designs, such as matching exposed cases with initially uninfected individuals who may later contract COVID-19. Additionally, they could evaluate the cumulative impact of recurrent infections on kidney function (eAppendix 16 in Supplement 1).

## Conclusions

This cohort study of more than 1.9 million US children and adolescents, leveraging data from 19 health institutions in US, represents, to our knowledge, one of the most comprehensive investigations into the long-term kidney outcomes of SARS-CoV-2 infection in pediatric populations. Results of this study suggest that SARS-CoV-2 infection is associated with an increased risk of adverse kidney outcomes, including new-onset CKD and worsening kidney function, particularly among children with preexisting CKD or acute-phase AKI, underscoring the importance of long-term monitoring for kidney health in children and adolescents affected by COVID-19.

## Notes

Supplement 1. **eAppendix 1.** Data and Methods Description **eAppendix 2.** Study Variable Specifications **eAppendix 3.** Patient Characteristic Balance Between Groups in Primary Analysis **eAppendix 4.** Sensitivity Analysis for Subvariants **eAppendix 5.** Sensitivity Analysis for Sex **eAppendix 6.** Sensitivity Analysis for Race and Ethnicity **eAppendix 7**. Sensitivity Analysis for Subgroups With and Without Obesity **eAppendix 8.** Sensitivity Analysis for Subgroups With Different Hospitalization Status **eAppendix 9.** Sensitivity Analysis for Subgroups With Different Levels of COVID-19 Severity **eAppendix 10.** Sensitivity Analysis for Subgroups With Different Age Group **eAppendix 11.** Sensitivity Analysis for AKI Subgroup Without Excluding Patients With Dialysis During Acute Phase **eAppendix 12.** Sensitivity Analysis for AKI Subgroup Using the Lowest Creatinine Measurement Within 90 Days Before the Index Date as the Baseline **eAppendix 13.** Sensitivity Analysis for CKD Subgroup Without Excluding Patients With ESKD During Baseline **eAppendix 14.** More Detailed Version of Table 1 Including Site Information and Detailed Breakdown of Age Distribution **eAppendix 15.** More Detailed Version of Figure 1, Including Variations of Definitions of CKD Stage 2 or Higher and CKD Stage 3 or Higher **eAppendix 16.** Recurrent COVID-19 Infections and Outcomes

Supplement 2. **Nonauthor Collaborators.** The RECOVER Consortium

Supplement 3.
Data Sharing Statement
