# Errors in Table 2

**DOI:** 10.1001/jamanetworkopen.2025.23267

**Published:** 2025-06-30

In the Original Investigation titled “Kidney Function Following COVID-19 in Children and Adolescents,”^1^ published April 11, 2025, there were errors in Table 2. In the AKI segment and the No AKI or CKD segment, 3 variations in each category were repeated. This article has been corrected.^1^
