# Supplementary material for: Kidney Function Following COVID-19 in Children and Adolescents
Source: JAMA Netw Open. 2025 Apr 11;8(4):e254129. doi: 10.1001/jamanetworkopen.2025.4129 (PMC11992607; doi:10.1001/jamanetworkopen.2025.4129)
Supplement: Supplement 1. — eAppendix 1. Data and Methods Description eAppendix 2. Study Variable Specifications eAppendix 3. Patient Characteristic Balance Between Groups in Primary Analysis eAppendix 4. Sensitivity Analysis for Subvariants eAppendix 5. Sensitivity Analysis for Sex eAppendix 6. Sensitivity Analysis for Race and Ethnicity eAppendix 7. Sensitivity Analysis for Subgroups With and Without Obesity eAppendix 8. Sensitivity Analysis for Subgroups With Different Hospitalization Status eAppendix 9. Sensitivity Analysis for Subgroups With Different Levels of COVID-19 Severity eAppendix 10. Sensitivity Analysis for Subgroups With Different Age Group eAppendix 11. Sensitivity Analysis for AKI Subgroup Without Excluding Patients With Dialysis During Acute Phase eAppendix 12. Sensitivity Analysis for AKI Subgroup Using the Lowest Creatinine Measurement Within 90 Days Before the Index Date as the Baseline eAppendix 13. Sensitivity Analysis for CKD Subgroup Without Excluding Patients With ESKD During Baseline eAppendix 14. More Detailed Version of Table 1 Including Site Information and Detailed Breakdown of Age Distribution eAppendix 15. More Detailed Version of Figure 1, Including Variations of Definitions of CKD Stage 2 or Higher and CKD Stage 3 or Higher eAppendix 16. Recurrent COVID-19 Infections and Outcomes [file jamanetwopen-e254129-s001.pdf]

Supplementary Online Content

Li L, Zhou T, Lu Y, et al; RECOVER Consortium. Kidney function following COVID-19 in children and adolescents in the RECOVER initiative. *JAMA Netw Open.* 2025;8(4):e254129. doi:10.1001/jamanetworkopen.2025.4129

eAppendix 1. Data and Methods Description
eAppendix 2. Study Variable Specifications
eAppendix 3. Patient Characteristic Balance Between Groups in Primary Analysis
eAppendix 4. Sensitivity Analysis for Subvariants
eAppendix 5. Sensitivity Analysis for Sex
eAppendix 6. Sensitivity Analysis for Race and Ethnicity
eAppendix 7. Sensitivity Analysis for Subgroups With and Without Obesity
eAppendix 8. Sensitivity Analysis for Subgroups With Different Hospitalization Status
eAppendix 9. Sensitivity Analysis for Subgroups With Different Levels of COVID-19 Severity
eAppendix 10. Sensitivity Analysis for Subgroups With Different Age Group
eAppendix 11. Sensitivity Analysis for AKI Subgroup Without Excluding Patients With Dialysis During Acute Phase
eAppendix 12. Sensitivity Analysis for AKI Subgroup Using the Lowest Creatinine Measurement Within 90 Days Before the Index Date as the Baseline
eAppendix 13. Sensitivity Analysis for CKD Subgroup Without Excluding Patients With ESKD During Baseline
eAppendix 14. More Detailed Version of Table 1 Including Site Information and Detailed Breakdown of Age Distribution
eAppendix 15. More Detailed Version of Figure 1, Including Variations of Definitions of CKD Stage 2 or Higher and CKD Stage 3 or Higher
eAppendix 16. Recurrent COVID-19 Infections and Outcomes

This supplementary material has been provided by the authors to give readers additional information about their work.

© 2025 Li L et al. *JAMA Network Open*.

eAppendix 1. Data and Methods Description

A. Description of electronic health records data

We based our analysis on real-world data from electronic health records (EHRs), encompassing a broad range of routinely collected information in hospital settings—such as clinical data (diagnoses, treatments), laboratory and test outcomes, and administrative details (patient demographics, billing). Specifically, we utilized the hospital-based EHR data from the Researching COVID to Enhance Recovery (RECOVER) Initiative COVID-19 Database to define exposure, outcomes, and covariates. Unlike general practitioner (GP) data, self-reported information, or external sources, our study drew on the structured EHR entries recorded by healthcare providers in hospital environments. This approach offers a more comprehensive, integrated view of each patient's health status, medical history, and healthcare interactions across various providers and settings.

B. RECOVER Population and generalizability

The National Institutions of Health (NIH) launched the new RECOVER initiative in 2021 to leverage electronic health record (EHR) data to better identify and characterize patients with pos-acute sequelae of SARS-CoV-2 infection (PASC). RECOVER obtains EHRs from three large national healthcare networks within the United States, covering regional catchment areas across 41 states. These networks collectively hold the EHRs of over 60 million patients, including records from more than 7 million individuals who have been affected by COVID-19. RECOVER collaborates with the National Institutes of Health's (NIH) All of Us Research Program, which contributes additional health records to this vast database. Together, these sources comprise one of the world's largest collections of EHRs.

In our study, nineteen sites contributed to the data: Cincinnati Children's Hospital Medical Center, Children's Hospital of Philadelphia, Children's Hospital of Colorado, Duke University, University of Iowa Healthcare, Ann & Robert H. Lurie Children's Hospital of Chicago, Medical College of Wisconsin, University of Michigan, University of Missouri, Children's Hospital at Montefiore, Medical University of South Carolina, Children's National Medical Center, Nationwide Children's Hospital, University of Nebraska Medical Center, Nemours Children's Health System (in Delaware and Florida), Northwestern University, New York University School of Medicine, OCHIN, Ohio State University, University of Pittsburgh, Seattle Children's Hospital, Stanford Children's Health, University of California San Francisco, Vanderbilt University Medical Center, Wake Forest University Health Sciences, Weill Cornell Medical College, Emory University, and Louisiana State University.

For this study, we used the s9 version of the data, collected till June 2023, which comprises 6,868,813 patients.

Our study represents the most comprehensive research conducted in the U.S. on post-acute sequelae of SARS-CoV-2 infection (PASC) in children and adolescents, analyzing a total of

© 2025 Li L et al. *JAMA Network Open*.

1,900,146 samples. It features a longer follow-up period than most existing studies targeting this age group in the U.S., specifically covering the timeframes of both the Delta and Omicron variant outbreaks. We recognize the potential limitations of EHR studies, particularly concerning the completeness of longitudinal follow-up for individuals. To mitigate this, our study only included patients who had an encounter within the 24 months prior to the cohort entry. This criterion ensured that patients had some documented engagement with healthcare providers, improving the likelihood of capturing relevant baseline and follow-up data.

C. Detailed description of the method

To adjust for confounders, we employed propensity score (PS) stratification. We fitted a L1-regularized logistic regression model by regressing the response variable, COVID-19 infection status, on covariates, including demographic, clinical, and healthcare utilization factors as listed in the study variables. The predicted probabilities from the logistic regression model give the PS for each patient, representing their likelihood of belonging to the COVID-19 positive group given the observed covariates. The population was then stratified into six equally sized strata (quintiles) based on the distribution of propensity scores. Each stratum represented a distinct level of risk for COVID-19 positivity, making more comparable groups across strata. Within each stratum, the HR was estimated using a stratified Cox regression model stratified by the PS strata.

eAppendix 2. Study Variable Specifications

Supplement Table 1: Variables used in the study evaluating the effect of the SARS-CoV-2 infection on kidney function outcomes in children and adolescents during the study period.

Variable	Functional form	Values	Detail	Codes/references
**Treatment (i.e., Exposure)**				
**Documented infection**	Indicator	Yes/No	Based on the observation and visit occurrence domains. Defined as a polymerase-chain-reaction (PCR), serology, or antigen tests positive for COVID-19, or diagnoses of COVID-19, post-acute sequelae of SARS-CoV-2 (PASC), or multisystem inflammatory syndrome in children (MIS-C) regardless of the presence of symptoms.	See https://github.com/PEDSnet/PASC/tree/main/observation_derivation_recover_ml_phenotype/specs for detailed codes.
**Outcomes**				
**Chronic kidney disease (CKD) stage 2 and higher (2+)**	Indicator and Date	Yes/No and Date	Date of the second eGFR of the earliest pair of eGFR values <90 mL/min/1.73m2, separated by >=90 days, without an intervening eGFR >=90	
**CKD stage 3 and higher (3+)**	Indicator and Date	Yes/No and Date	Date of the second eGFR of the earliest pair of eGFR values <60 mL/min/1.73m2, separated by >=90 days, without an intervening eGFR >=90	
**Composite kidney outcome**	Indicator and Date	Yes/No and Date	A composite outcome of chronic kidney dialysis, kidney transplant, eGFR decline of ≥50%, ESKD diagnosis, or eGFR ≤15 mL/min/1.73m2	
**eGFR decline**	Indicator and Date	Yes/No and Date	eGFR decline of >=30%, >=40%, and >=50%	
**Confounding variables**				
**Age (years)**	Linear	NA	Based on records in the person domain.	Age is defined as the integer of (date – birth date)/365.25
**Sex**	Indicator	Male/Female	Based on records in the person domain.	NA
**Race/Ethnicity**	4 categories	Non-Hispanic White<br>Non-Hispanic Black<br>Hispanic<br>Other/unknown	Based on records in the person domain.	NA
**Site**	25 categories	25 different sites (hospitals)	Based on records in the person domain	NA

**Obesity**	Indicator	Yes/No	Based on records in the measurement domain.<br>If measured at age < 24*30.5 days, NHANES weight z score > 1.64<br>If measured at 24*30.5 < age <240*30.5, NHANES BMI z score > 1.64	NA
**PMCA (Pediatric Medical Complexity Algorithm)**	3 categories	No chronic condition (PMCA = 0)<br>Non-complex chronic condition (PMCA = 1)<br>Complex chronic condition comorbidities (PMCA = 2)	Based on the condition occurrence and visit occurrence domains.	Simon, T. D., Haaland, W., Hawley, K., Lambka, K., & Mangione-Smith, R. (2018). Development and validation of the Pediatric Medical Complexity Algorithm (PMCA) version 3.0. *Academic pediatrics*, *18*(5), 577-580.
**Number of visits to emergency department in 18 months to 7 days prior to the entry**	3 categories	0<br>1<br>>=2	Based on the condition occurrence and visit occurrence domains.	NA
**Number of inpatient visits in 18 months to 7 days prior to the entry**	3 categories	0<br>1<br>>=2	Based on the condition occurrence and visit occurrence domains, including Inpatient Hospital Stay, Emergency Department Admit to Inpatient Hospital Stay, and Observation Stay	NA
**Number of outpatient visits in 18 months to 7 days prior to the entry**	3 categories	0<br>1<br>>=2	Based on the condition occurrence and visit occurrence domains including Ambulatory/Outpatient Visit (With a Physician) and Interactive Telemedicine Service	NA
**Number of Covid-19 negative tests prior to the entry**	3 categories	0<br>1<br>>=2	Based on the condition occurrence and visit occurrence domains.	NA
**Variables for sensitivity analysis**				
**Mild COVID-19**	Indicator	Yes/No	Based on the condition occurrence domain.	Forrest, C. B., Burrows, E. K., Mejias, A., Razzaghi, H., Christakis, D., Jhaveri, R., Lee, G.

				M., Pajor, N. M., Rao, S., Thacker, D., & Bailey, L. C. (2022). Severity of Acute COVID-19 in Children <18 Years Old March 2020 to December 2021. *Pediatrics*, *149*(4). https://doi.org/10.1542/peds.2021-055765 Code sets are available through https://github.com/PEDSnet/COVID19_Severity/tree/main.
**Moderate or severe COVID-19**	Indicator	Yes/No	Based on the condition occurrence, procedure occurrence, drug, device and death domains.	Forrest, C. B., Burrows, E. K., Mejias, A., Razzaghi, H., Christakis, D., Jhaveri, R., Lee, G. M., Pajor, N. M., Rao, S., Thacker, D., & Bailey, L. C. (2022). Severity of Acute COVID-19 in Children <18 Years Old March 2020 to December 2021. *Pediatrics*, *149*(4). https://doi.org/10.1542/peds.2021-055765 Code sets are available through https://github.com/PEDSnet/COVID19_Severity/tree/main.
**ICU admission with COVID-19**	Indicator	Yes/No	Based on the adt_occurrence domain	NICU, CICU, PICU

Note: All of the domains in the table above are based on the PEDSnet common data model (CDM). More details are available through this link: https://data-models-service.research.chop.edu.

eAppendix 3. Patient Characteristic Balance Between Groups in Primary Analysis A. Propensity-score (PS) models and stratification

We built large-scale PS model for the cohort with baseline patient characteristics including

- Demographics (age at index date; race/ethnicity; gender)
- Obesity
- Chronic condition indicator as defined by the Pediatric Medical Complexity Algorithm (PMCA)
  - No chronic condition (PMCA = 0)
  - Non-complex chronic condition (PMCA = 1)
  - Complex chronic condition comorbidities (PMCA = 2)
- Healthcare utilization 24 months ~ 7 days prior to index date categorized to 0,1,2,>=3
  - Number of outpatient visits
  - Number of inpatient visits
  - Number of ED visits
  - Number of unique medications/prescriptions
  - Number of COVID tests
- Cohort entry date (index date) categorized to 1 month
- Hospital index
- Existence of a list of 205 chronic conditions 24 months ~ 7 days prior to index

We exclude all covariates that occur in fewer than 0.1% of participants in the cohort for computational efficiency.

B. Empirical equipoise assessment

To ensure that matching patients exist, we used clinical equipoise to evaluate the similarity between study groups [1, 2]. Equipoise is defined using preference score, which transforms the propensity score by incorporating treatment prevalence, facilitating interpretation. Mathematically, the preference score (F) can be written as follows, where S is the propensity score and P is the treatment prevalence:

$$\ln\left(\frac{F}{1-F}\right) = \ln\left(\frac{S}{1-S}\right) - \ln\left(\frac{P}{1-P}\right).$$

Equipoise is obtained by computing the fraction of the population (the union of the target and comparator cohorts) who had preference score between 0.3 and 0.7, i.e. a reasonable chance of receiving either target or comparator.

To evaluate the comparability between the study groups, we present the preference score, which offers an intuitive interpretation by transforming the propensity score to incorporate the prevalence of treatment. Specifically, the preference score (F) is linked to the propensity score (S) and the prevalence of treatment (P) through the equation: ln(F/(1-F)) = ln(S/(1-S)) - ln(P/(1-P)). Supplement s 1-3 below shows the distribution of preference scores across two treatment groups in each study cohort, which indicates high comparability of these studies.

Supplement Figure 1: Preference score distributions for COVID-19 positive and negative groups in no AKI or CKD group. A greater convergence of these distributions indicates a higher similarity in the predicted likelihood of being infected between the COVID-19 positive (red) and negative (blue) participants.

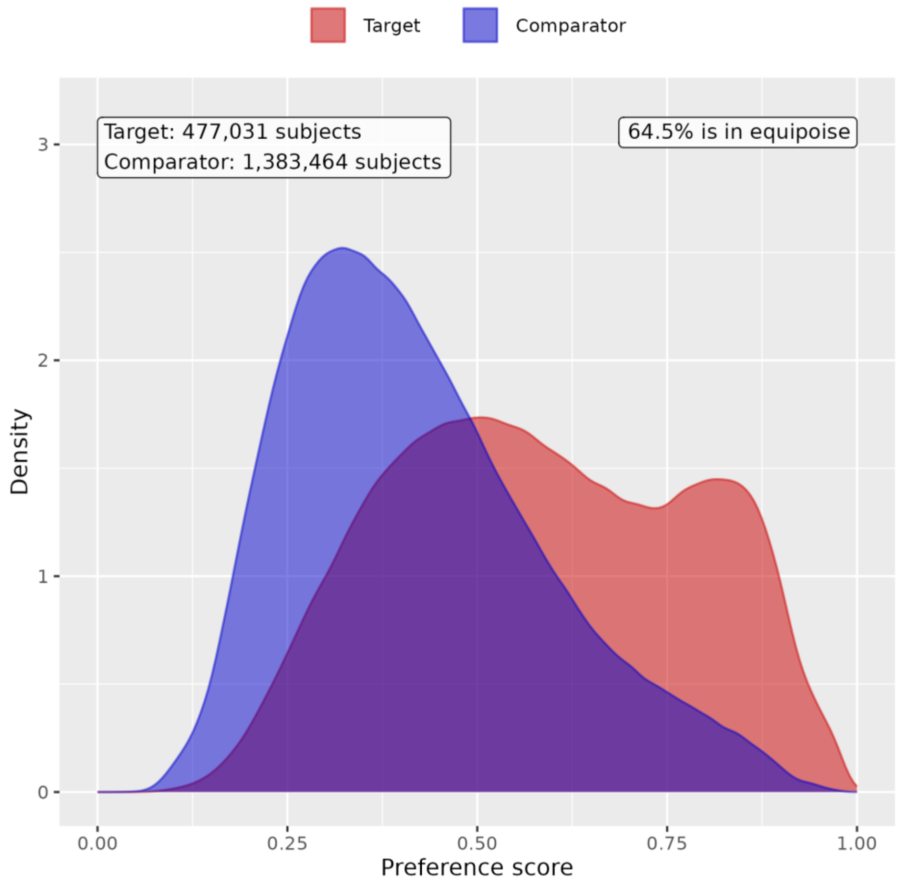
Target
Comparator
Target: 477,031 subjects
Comparator: 1,383,464 subjects
64.5% is in equipoise
Density
Preference score
0.00
0.25
0.50
0.75
1.00
0
1
2
3

Supplement Figure 2: Preference score distributions for COVID-19 positive and negative groups in CKD group. A greater convergence of these distributions indicates a higher similarity in the predicted likelihood of being infected between the COVID-19 positive (red) and negative (blue) participants.

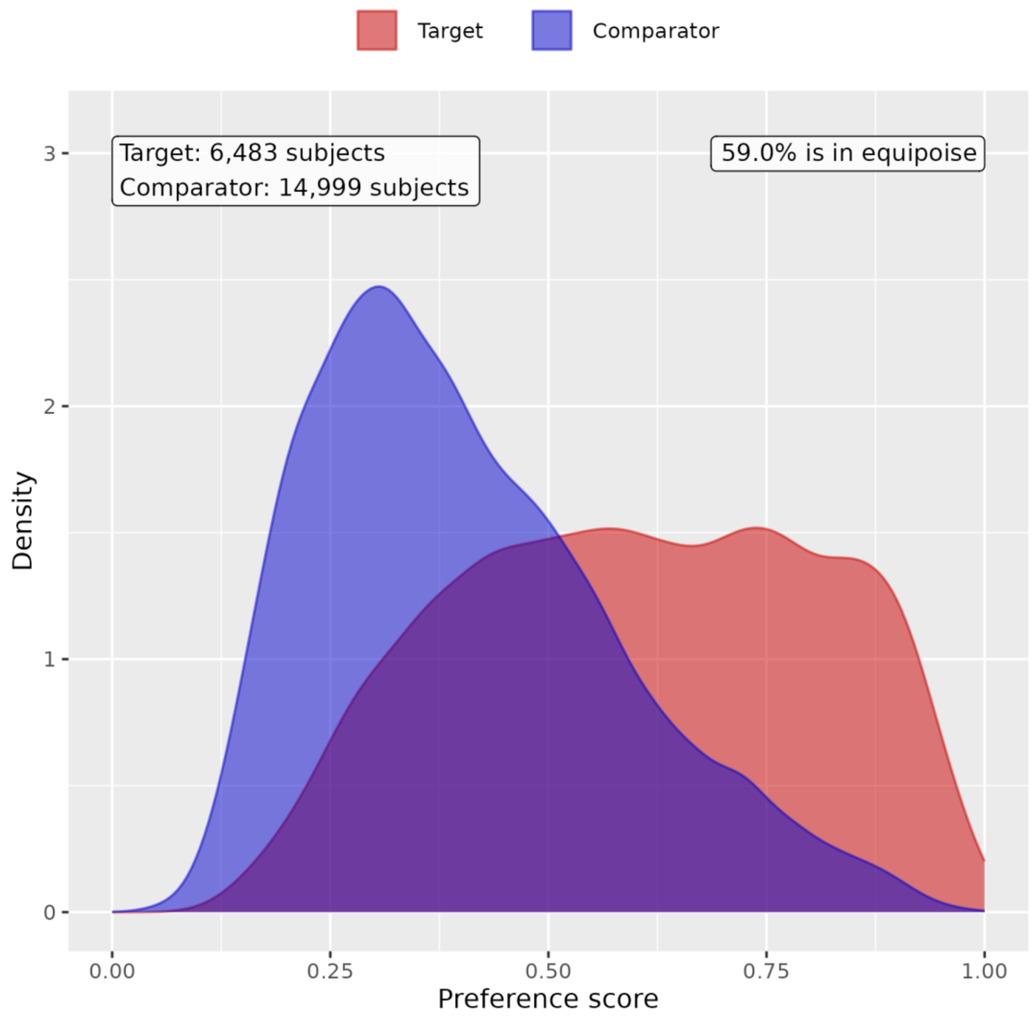

,

Supplement Figure 3: Preference score distributions for COVID-19 positive and negative groups in AKI group. A greater convergence of these distributions indicates a higher similarity in the predicted likelihood of being infected between the COVID-19 positive (red) and negative (blue) participants.

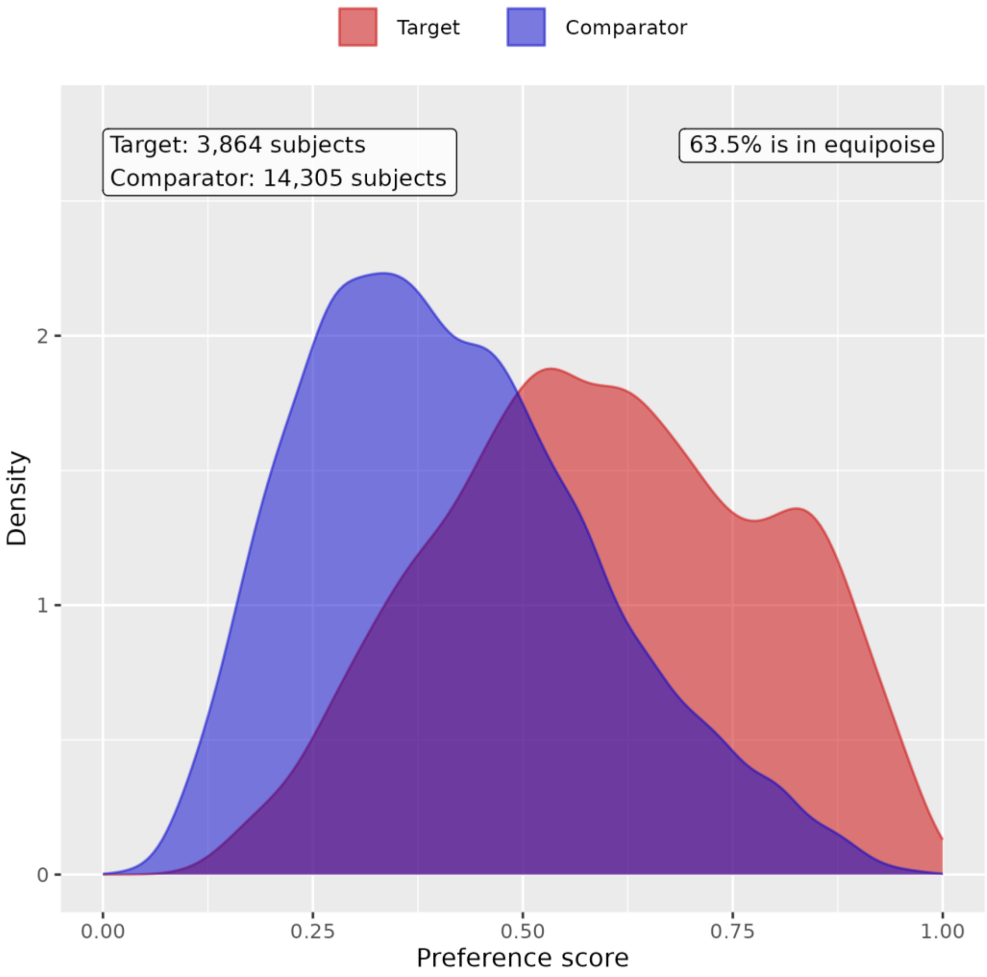

C. Patient characteristic balance in the primary analysis

We evaluate the balance of patient characteristics using the standardized difference of means (SMD). Supplement Figures 4-6 presents the SMD of three cohorts before and after PS score stratification.

Supplement Figure 4: Patient characteristic balance before and after large-scale PS stratification with 6 strata in no AKI or CKD group. The upper panel displays the top 20 covariates with the largest standardized difference of means before stratification, while the lower panel displays the top 20 covariates with the largest standardized difference of means after stratification.

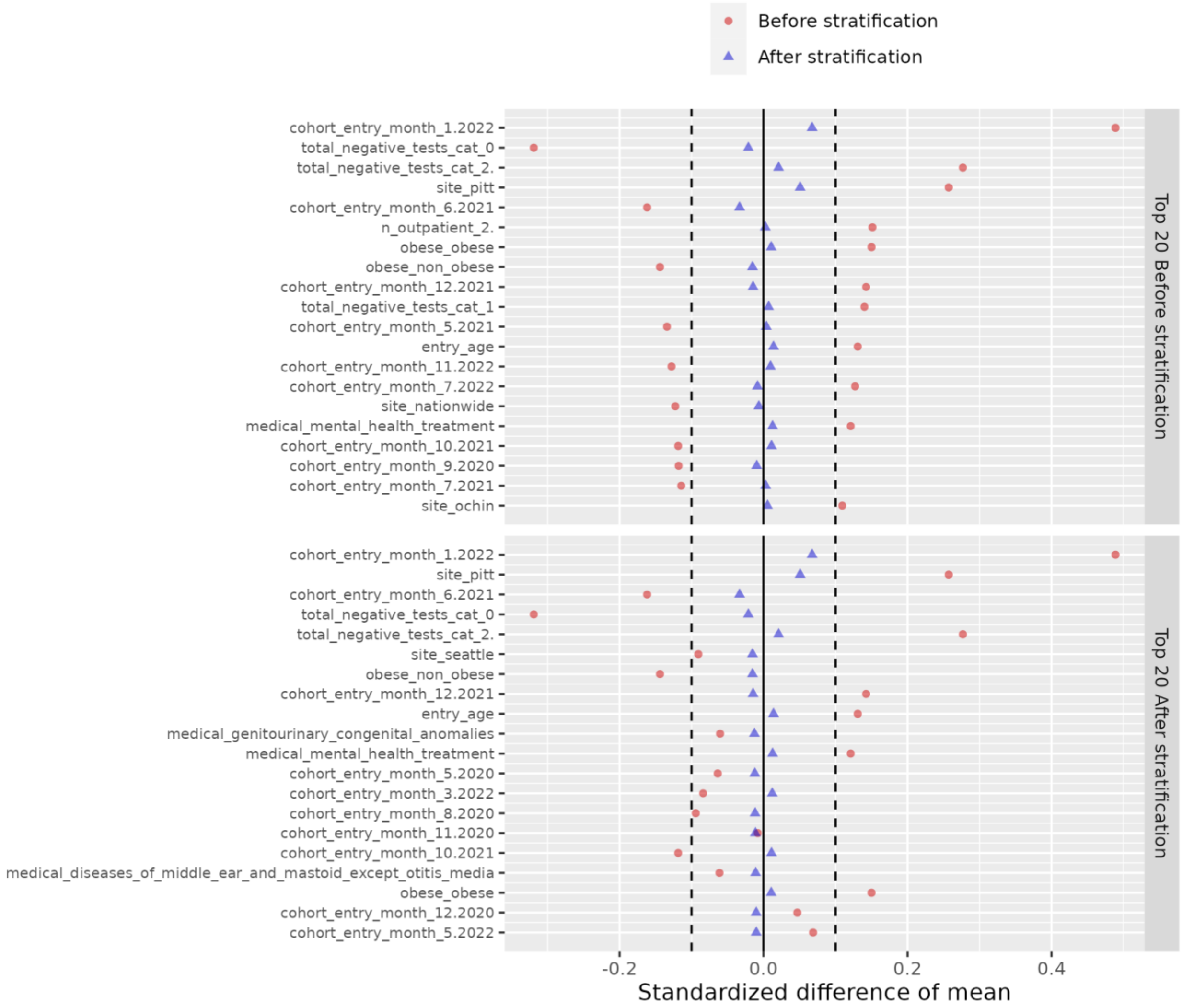

Supplement Figure 5: Patient characteristic balance before and after large-scale PS stratification with 6 strata in CKD group. The upper panel displays the top 20 covariates with the largest standardized difference of means before stratification, while the lower panel displays the top 20 covariates with the largest standardized difference of means after stratification.

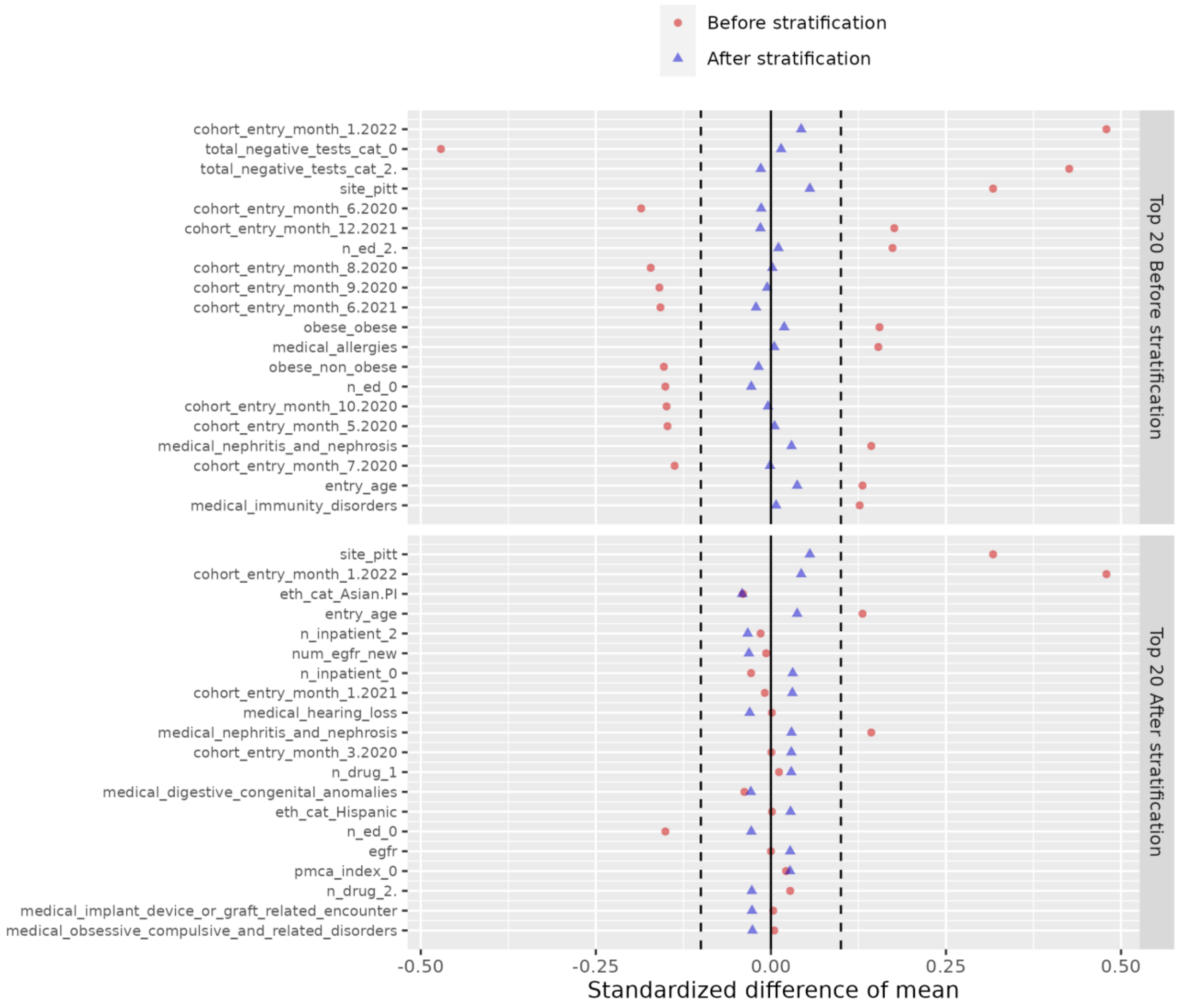

Supplement Figure 6: Patient characteristic balance before and after large-scale PS stratification with 6 strata in AKI group. The upper panel displays the top 20 covariates with the largest standardized difference of means before stratification, while the lower panel displays the top 20 covariates with the largest standardized difference of means after stratification.

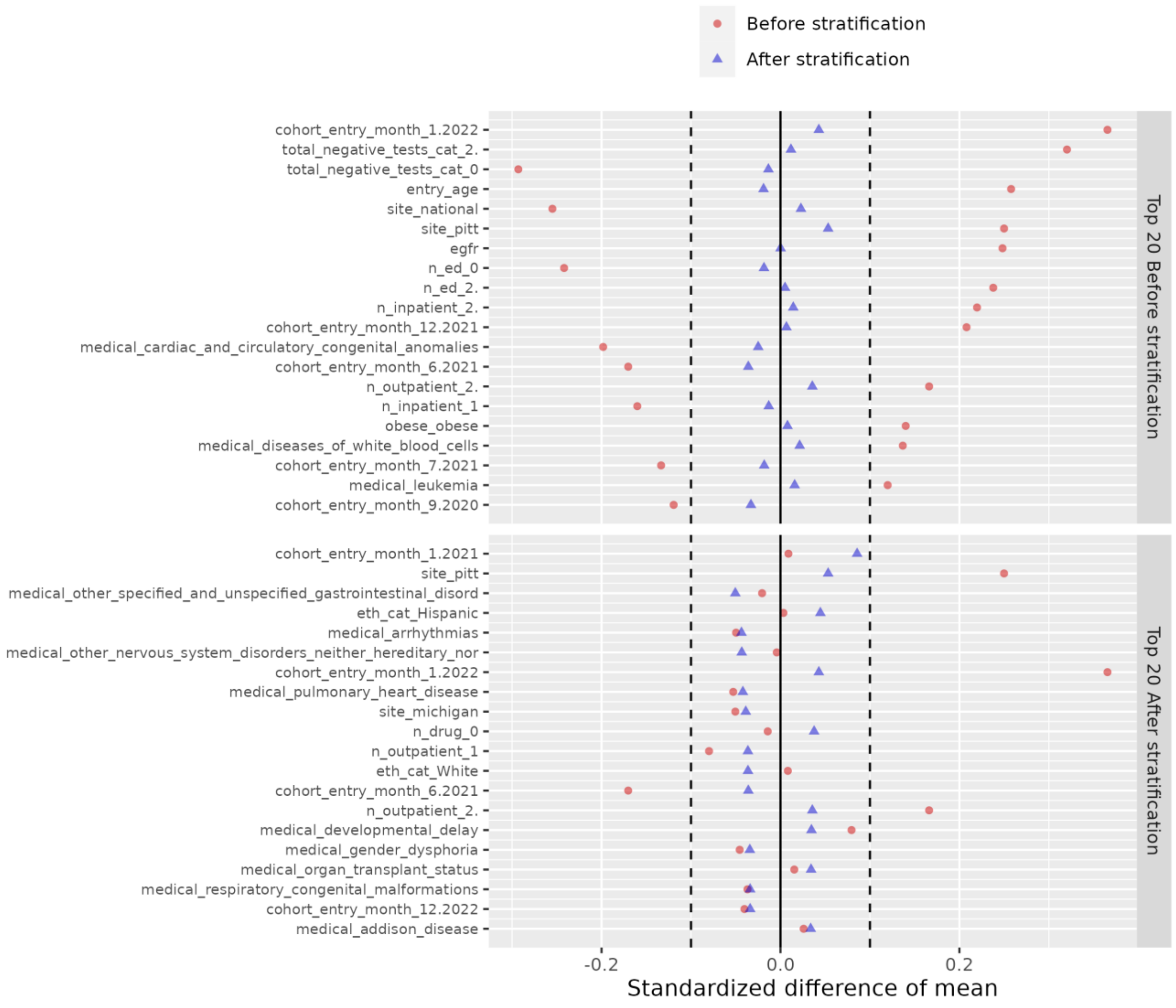

eAppendix 4. Sensitivity Analysis for Subvariants

We conducted sensitivity analyses to investigate if the risk of kidney function outcomes would be affected by the sub-variants. Therefore, we conducted the subgroup analysis based on three time periods: pre-Delta (2020/03/01 – 2021/05/31), Delta (2021/06/01 – 2021/12/31), and Omicron (2022/01/01 – 2023/03/06). The subgroups for the time periods were defined based on the index date of the patients.

A. Empirical equipoise assessment

Supplement Figure 7: Preference score distributions of COVID-19 positive and negative groups in children and adolescents with AKI during pre-Delta period. A greater convergence of these distributions indicates a higher similarity in the predicted likelihood of being infected between the COVID-19 positive (red) and negative (blue) participants.

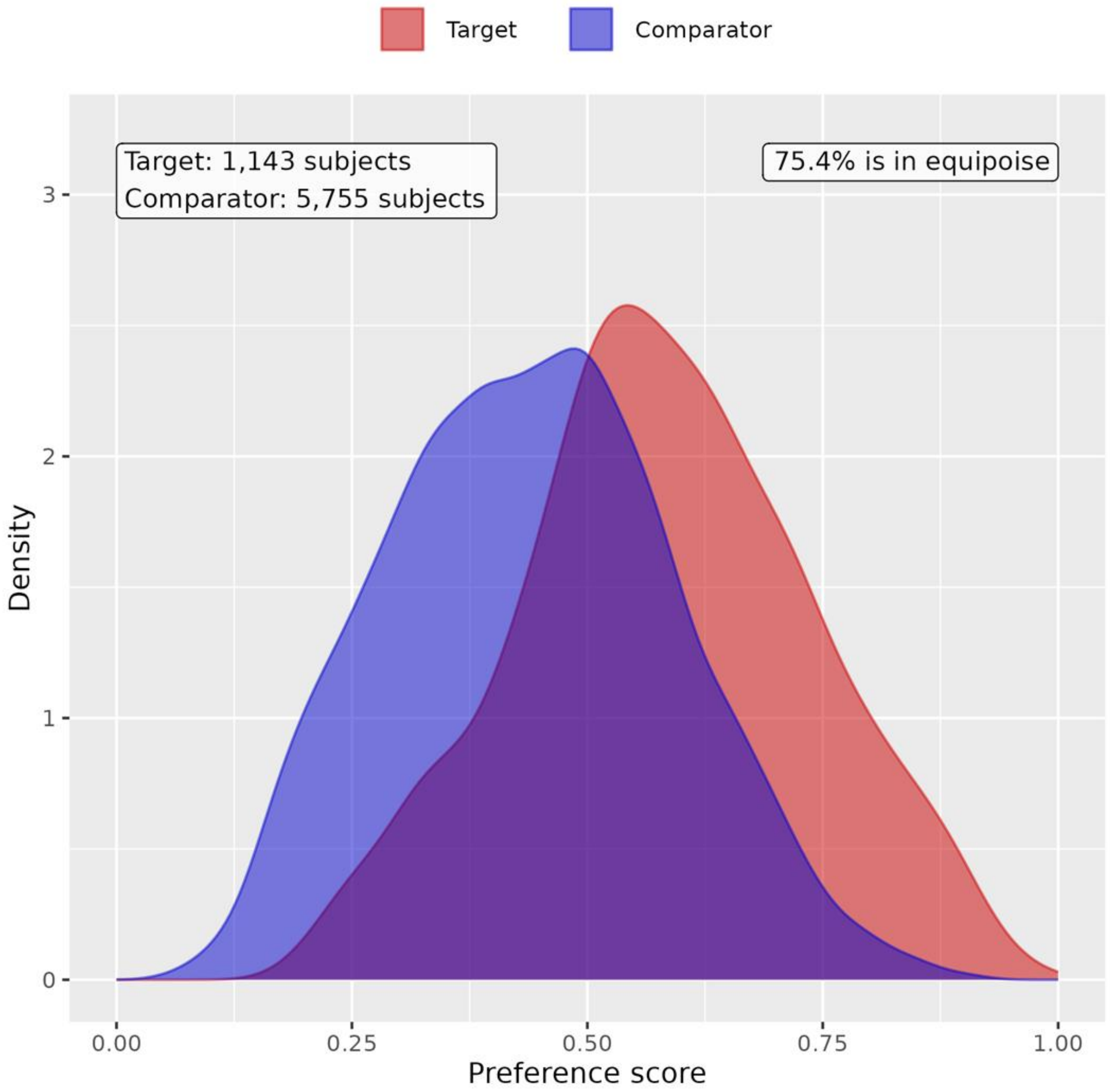
Target
Comparator
Target: 1,143 subjects
Comparator: 5,755 subjects
75.4% is in equipoise
Density
3
2
1
0
0.00
0.25
0.50
0.75
1.00
Preference score

Supplement Figure 8: Preference score distributions of COVID-19 positive and negative groups in children and adolescents with AKI during Delta period. A greater convergence of these distributions indicates a higher similarity in the predicted likelihood of being infected between the COVID-19 positive (red) and negative (blue) participants.

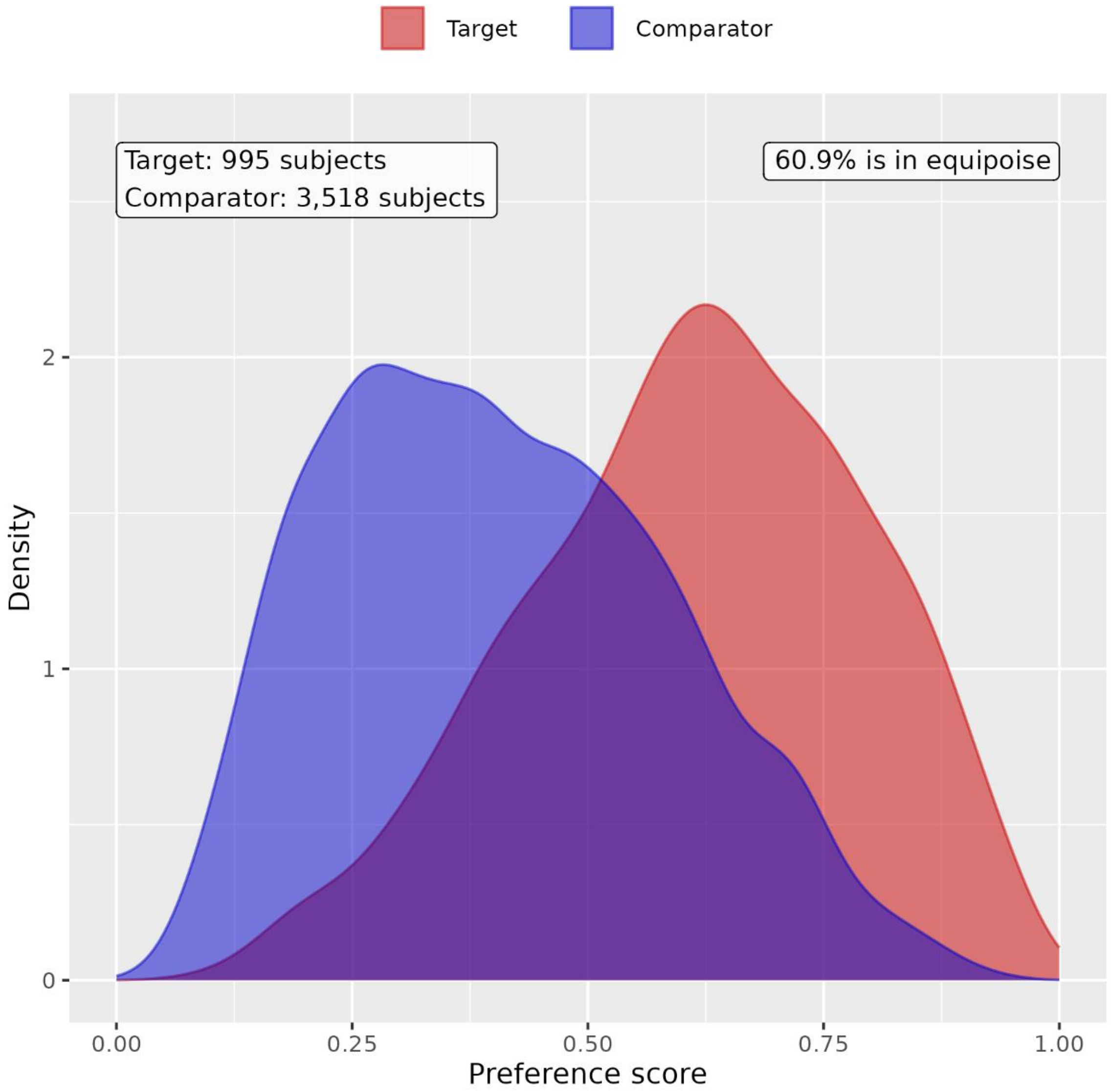

Supplement Figure 9: Preference score distributions of COVID-19 positive and negative groups in children and adolescents with AKI during Omicron period. A greater convergence of these distributions indicates a higher similarity in the predicted likelihood of being infected between the COVID-19 positive (red) and negative (blue) participants.

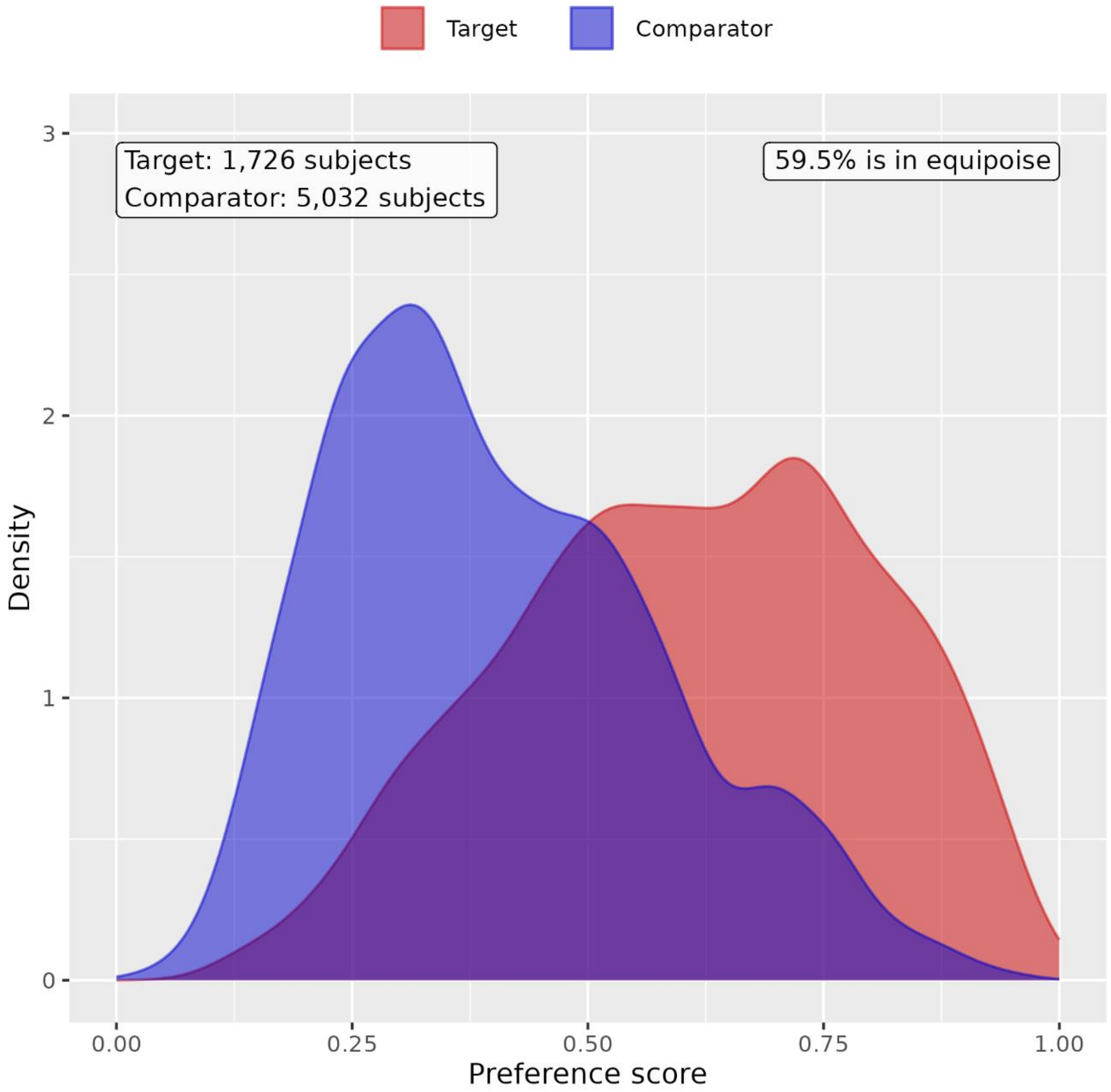

Supplement Figure 10: Preference score distributions of COVID-19 positive and negative groups for children and adolescents with CKD in pre-Delta period. A greater convergence of these distributions indicates a higher similarity in the predicted likelihood of being infected between the COVID-19 positive (red) and negative (blue) participants.

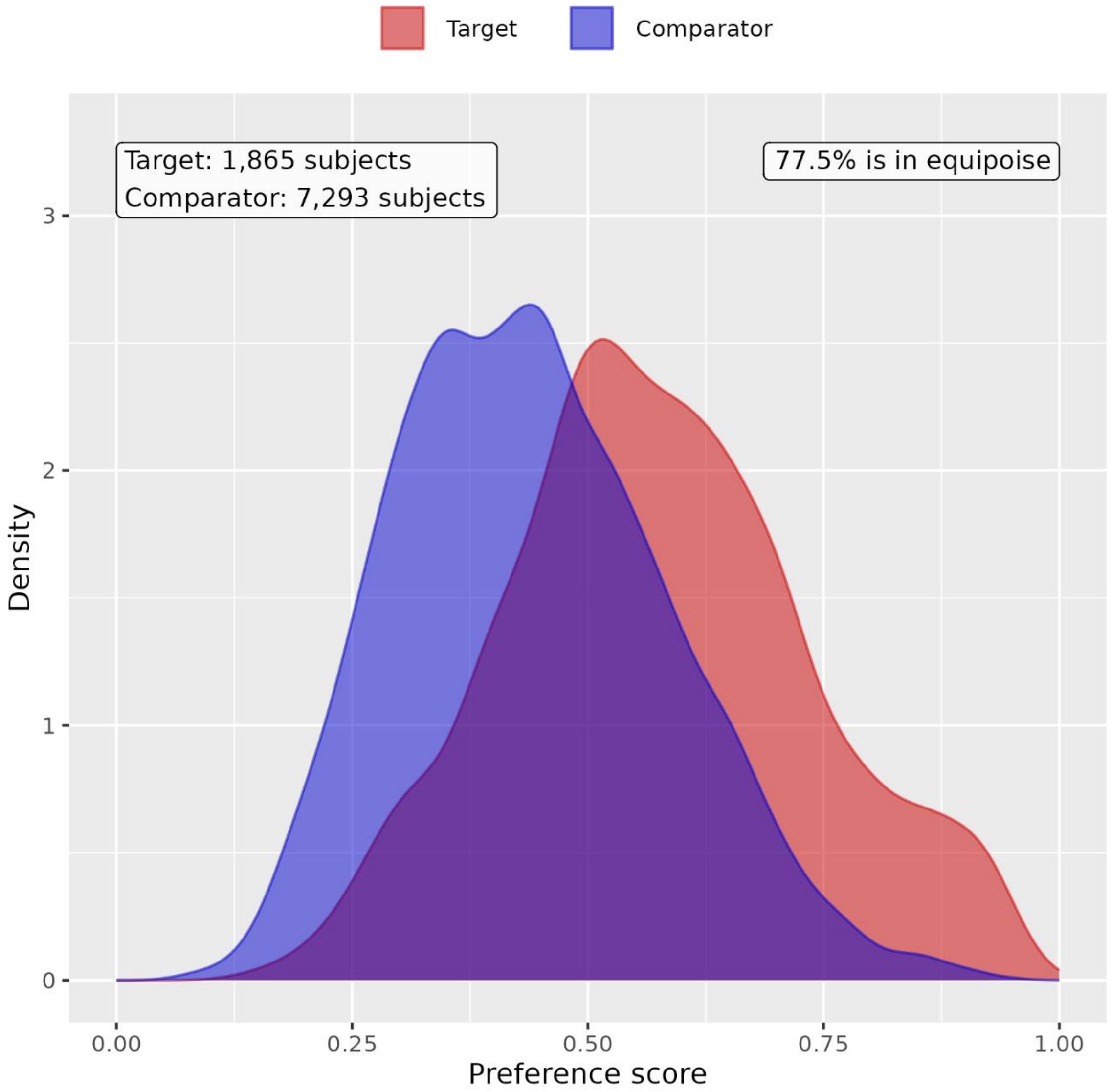

Supplement Figure 11: Preference score distributions of COVID-19 positive and negative groups for children and adolescents with CKD in Delta period. A greater convergence of these distributions indicates a higher similarity in the predicted likelihood of being infected between the COVID-19 positive (red) and negative (blue) participants.

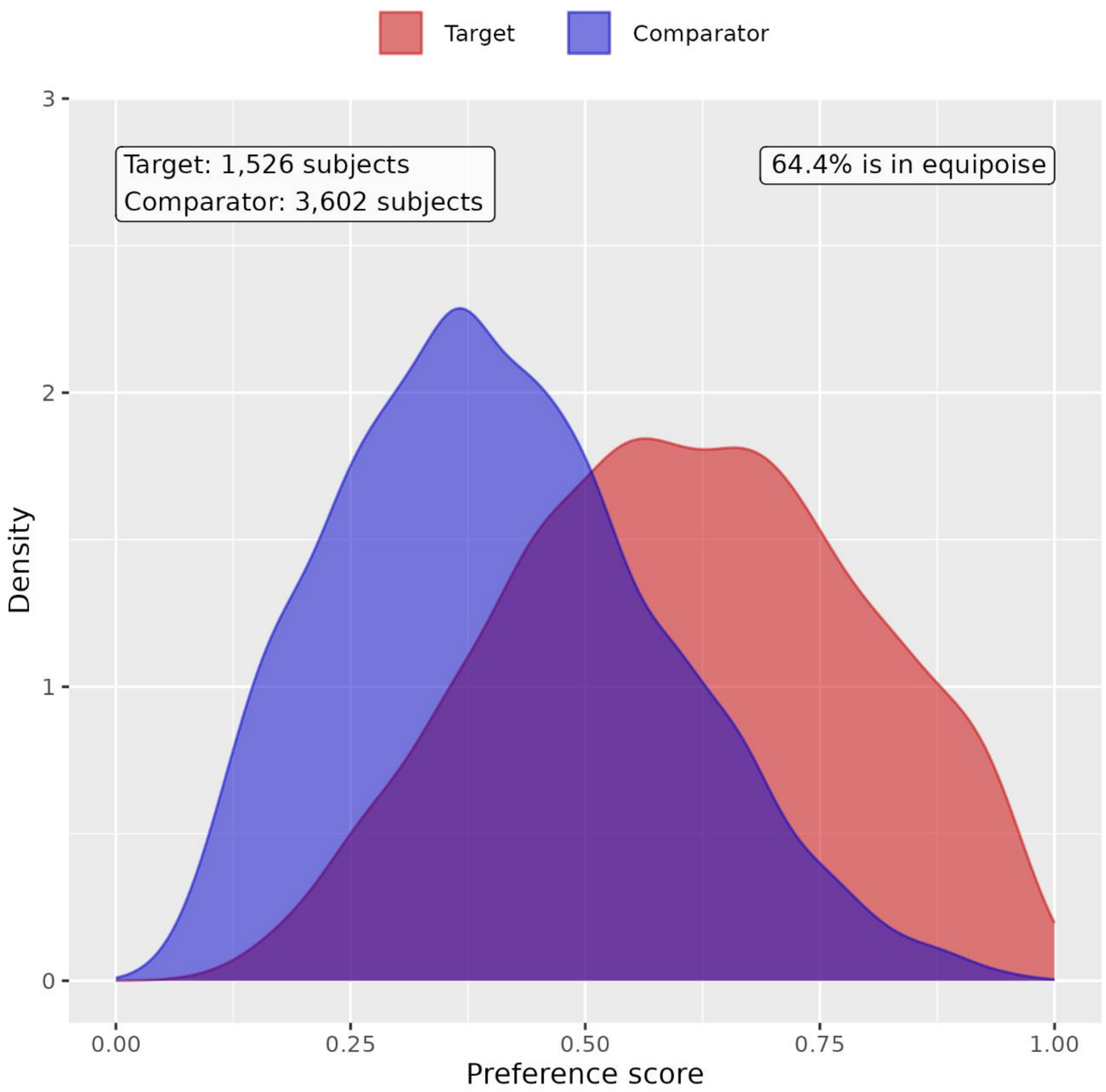

Supplement Figure 12: Preference score distributions of COVID-19 positive and negative groups for children and adolescents with CKD in Omicron period. A greater convergence of these distributions indicates a higher similarity in the predicted likelihood of being infected between the COVID-19 positive (red) and negative (blue) participants.

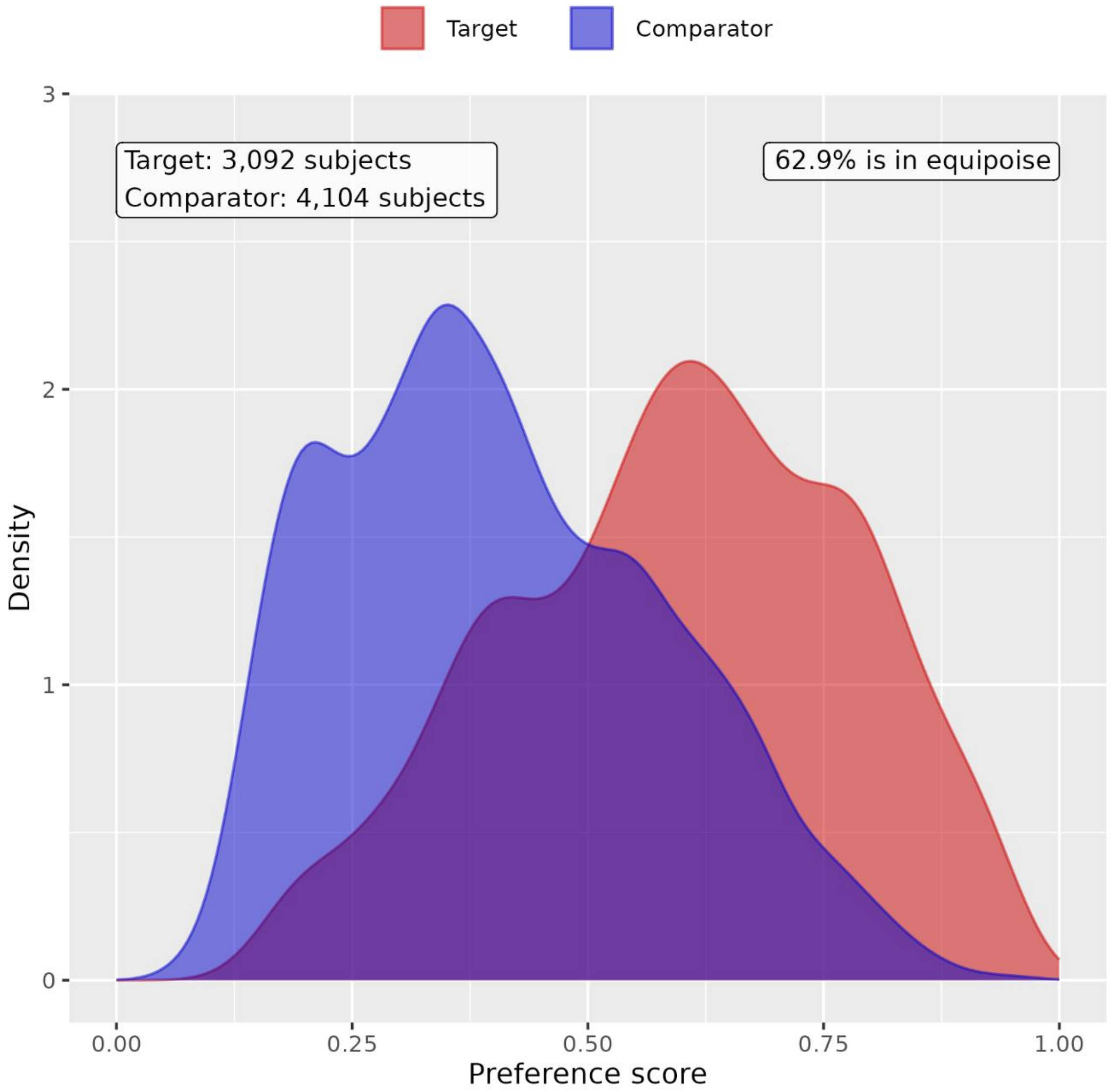

Supplement Figure 13: Preference score distributions of COVID-19 positive and negative groups for children and adolescents with no AKI or CKD in pre-Delta period. A greater convergence of these distributions indicates a higher similarity in the predicted likelihood of being infected between the COVID-19 positive (red) and negative (blue) participants.

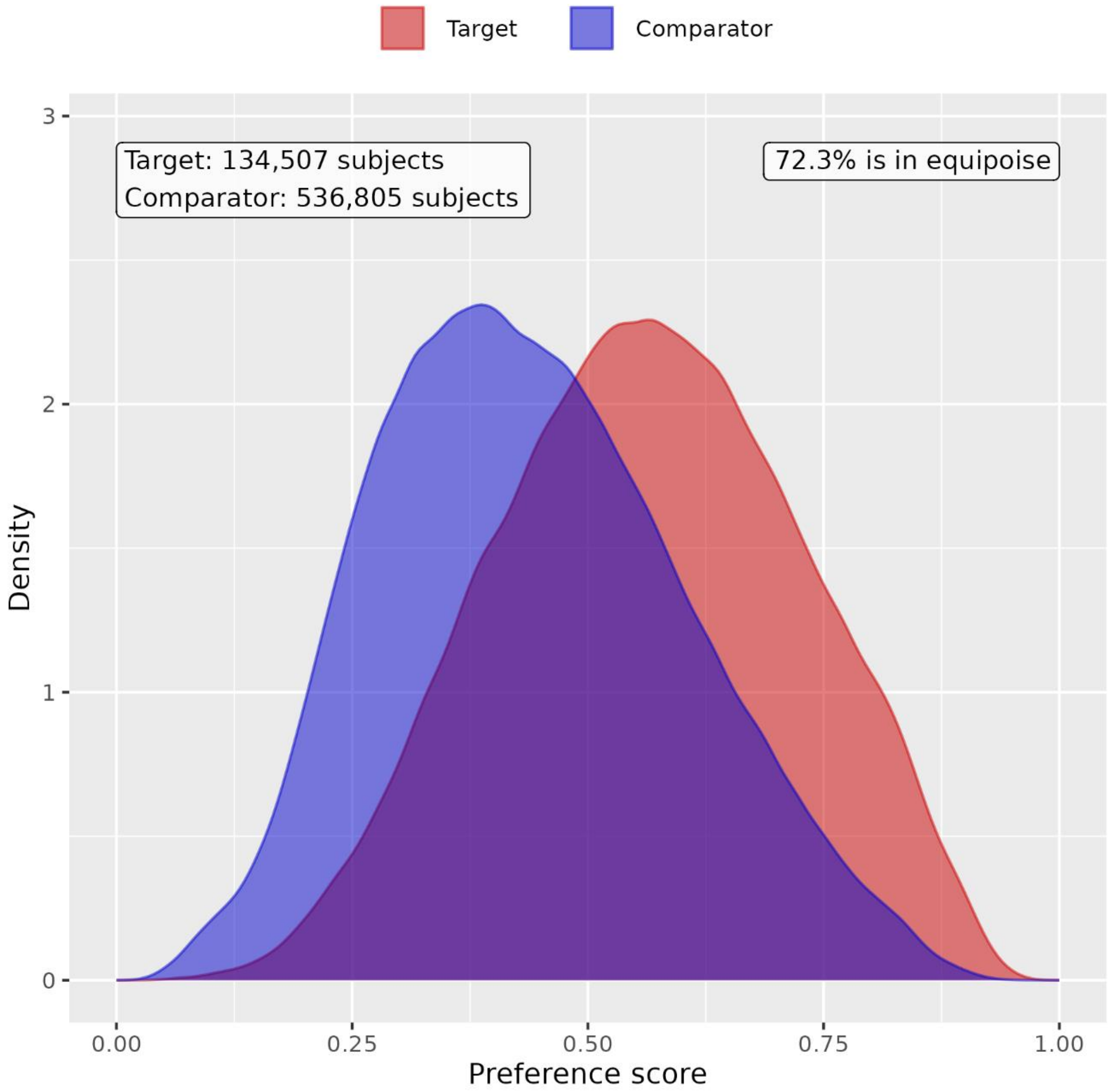

Supplement Figure 14: Preference score distributions of COVID-19 positive and negative groups for children and adolescents with no AKI or CKD in Delta period. A greater convergence of these distributions indicates a higher similarity in the predicted likelihood of being infected between the COVID-19 positive (red) and negative (blue) participants.

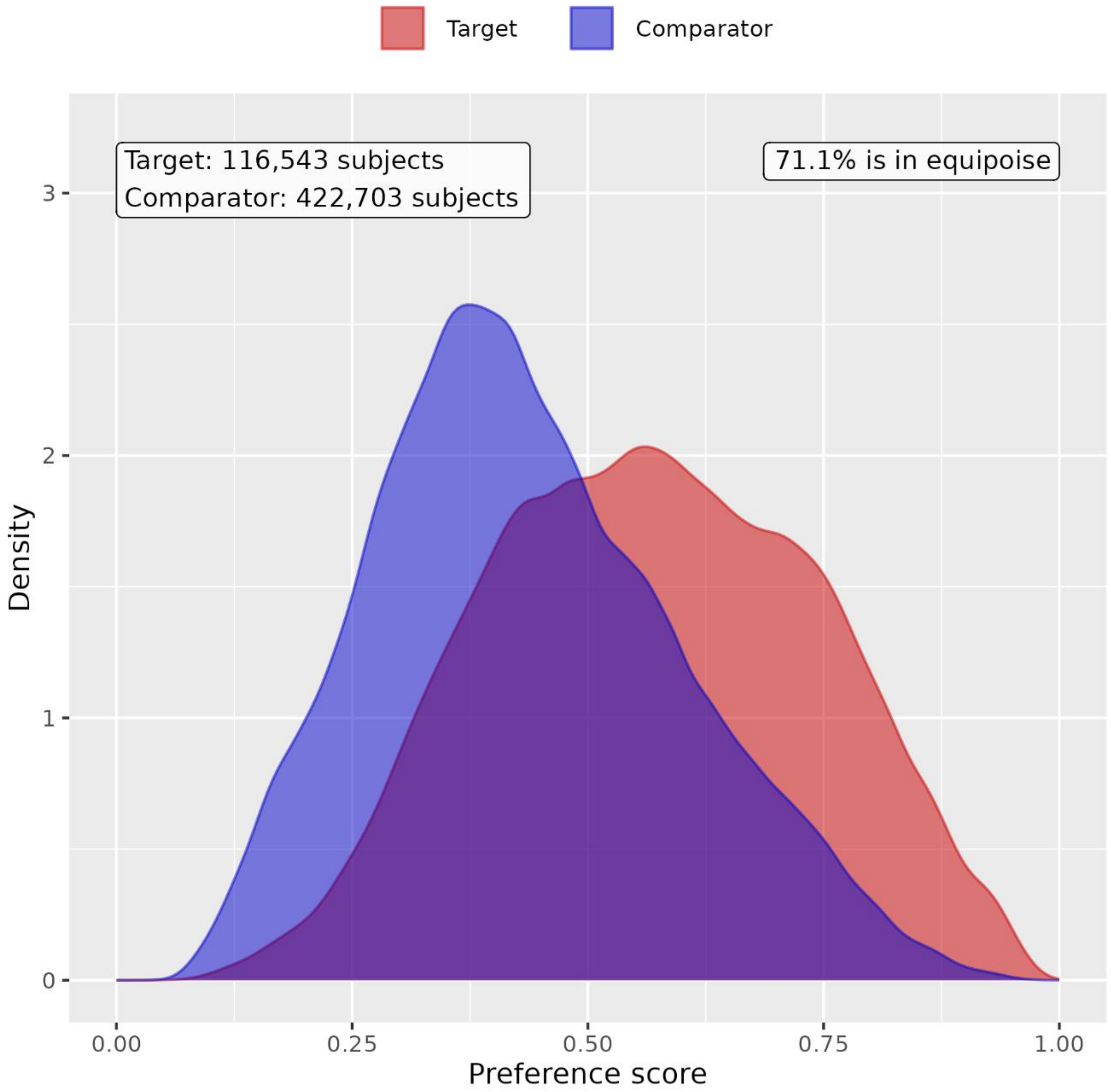

Supplement Figure 15: Preference score distributions of COVID-19 positive and negative groups for children and adolescents with no AKI or CKD in Omicron period. A greater convergence of these distributions indicates a higher similarity in the predicted likelihood of being infected between the COVID-19 positive (red) and negative (blue) participants.

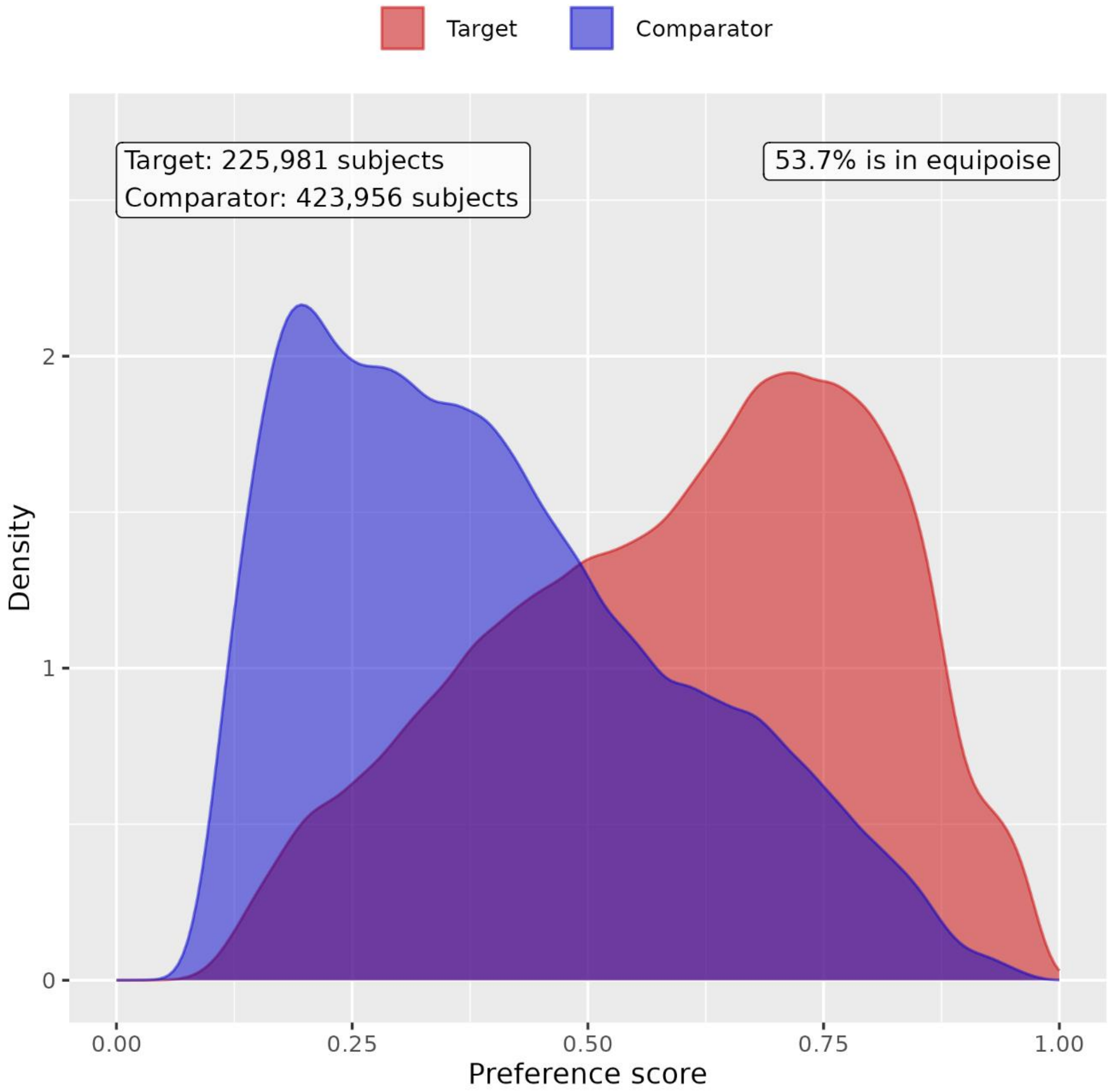

B. Patient characteristic balance

Supplement Figure 16: Patient characteristic balance before and after large-scale PS stratification with 6 strata for children and adolescents with AKI in pre-Delta period. The upper panel displays the top 20 covariates with the largest standardized difference of means before stratification, while the lower panel displays the top 20 covariates with the largest standardized difference of means after stratification.

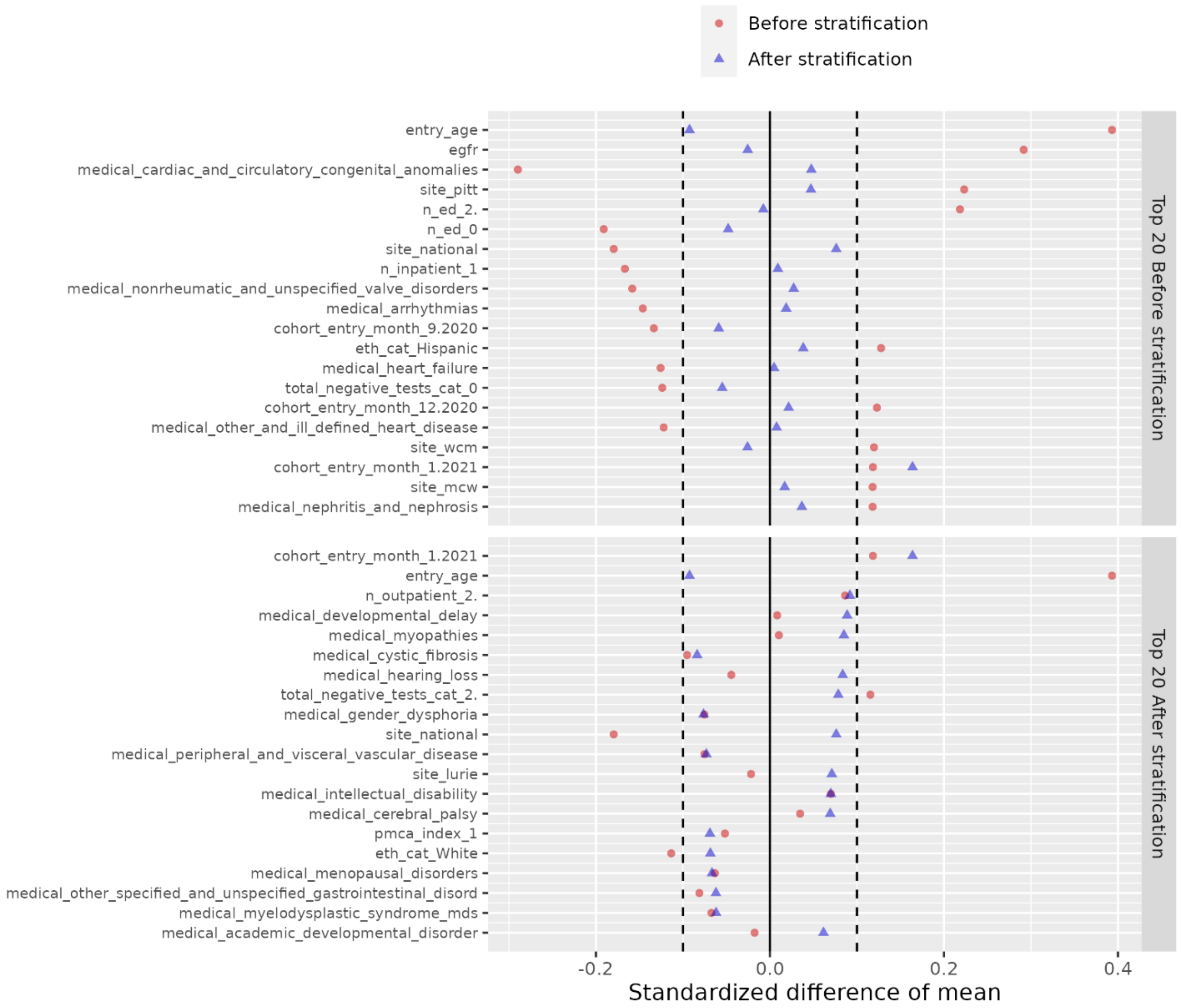

Supplement Figure 17: Patient characteristic balance before and after large-scale PS stratification with 6 strata for children and adolescents with AKI in Delta period. The upper panel displays the top 20 covariates with the largest standardized difference of means before stratification, while the lower panel displays the top 20 covariates with the largest standardized difference of means after stratification.

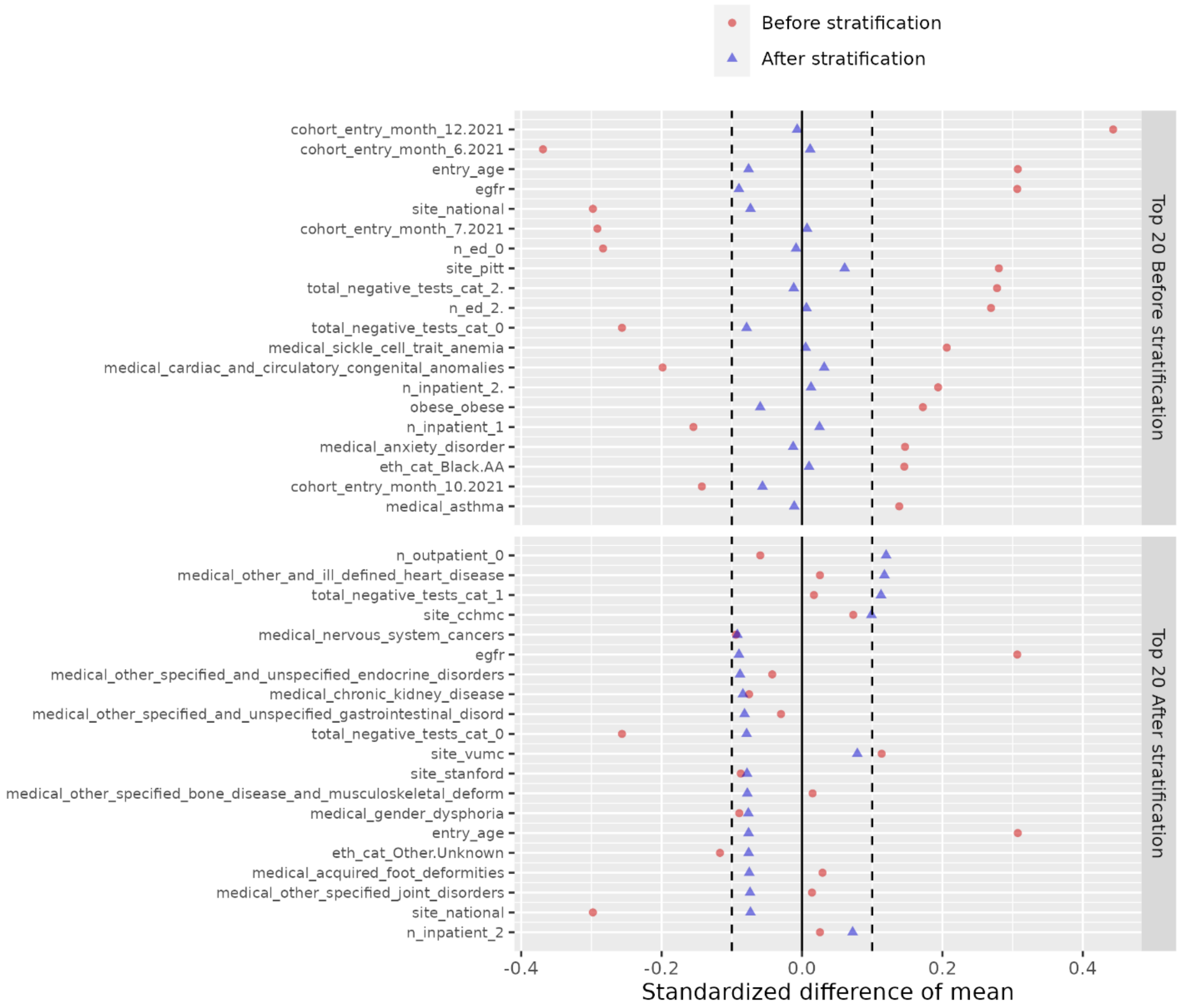

Supplement Figure 18: Patient characteristic balance before and after large-scale PS stratification with 6 strata for children and adolescents with AKI in Omicron period. The upper panel displays the top 20 covariates with the largest standardized difference of means before stratification, while the lower panel displays the top 20 covariates with the largest standardized difference of means after stratification.

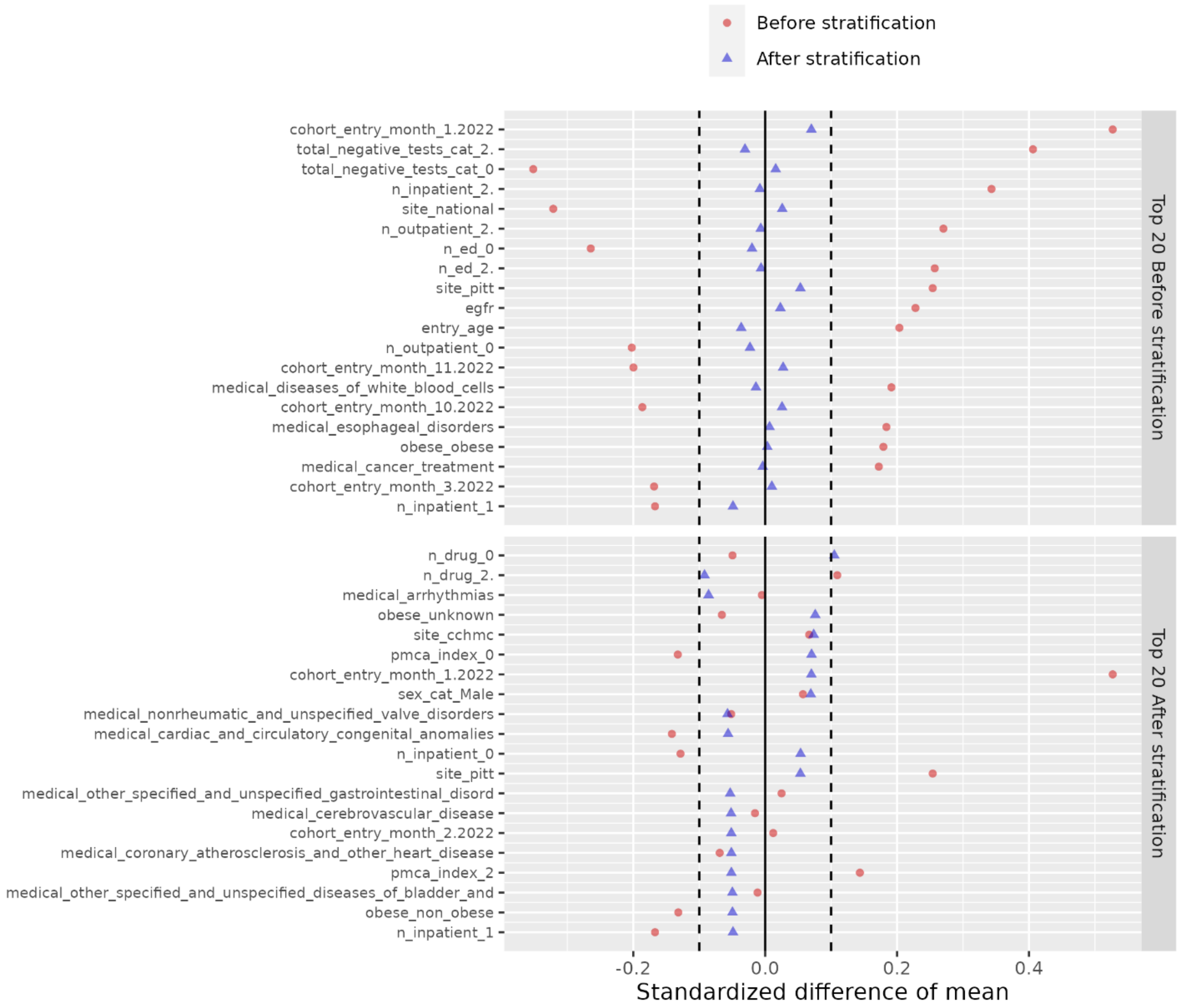

Supplement Figure 19: Patient characteristic balance before and after large-scale PS stratification with 6 strata for children and adolescents with CKD in pre-Delta period. The upper panel displays the top 20 covariates with the largest standardized difference of means before stratification, while the lower panel displays the top 20 covariates with the largest standardized difference of means after stratification.

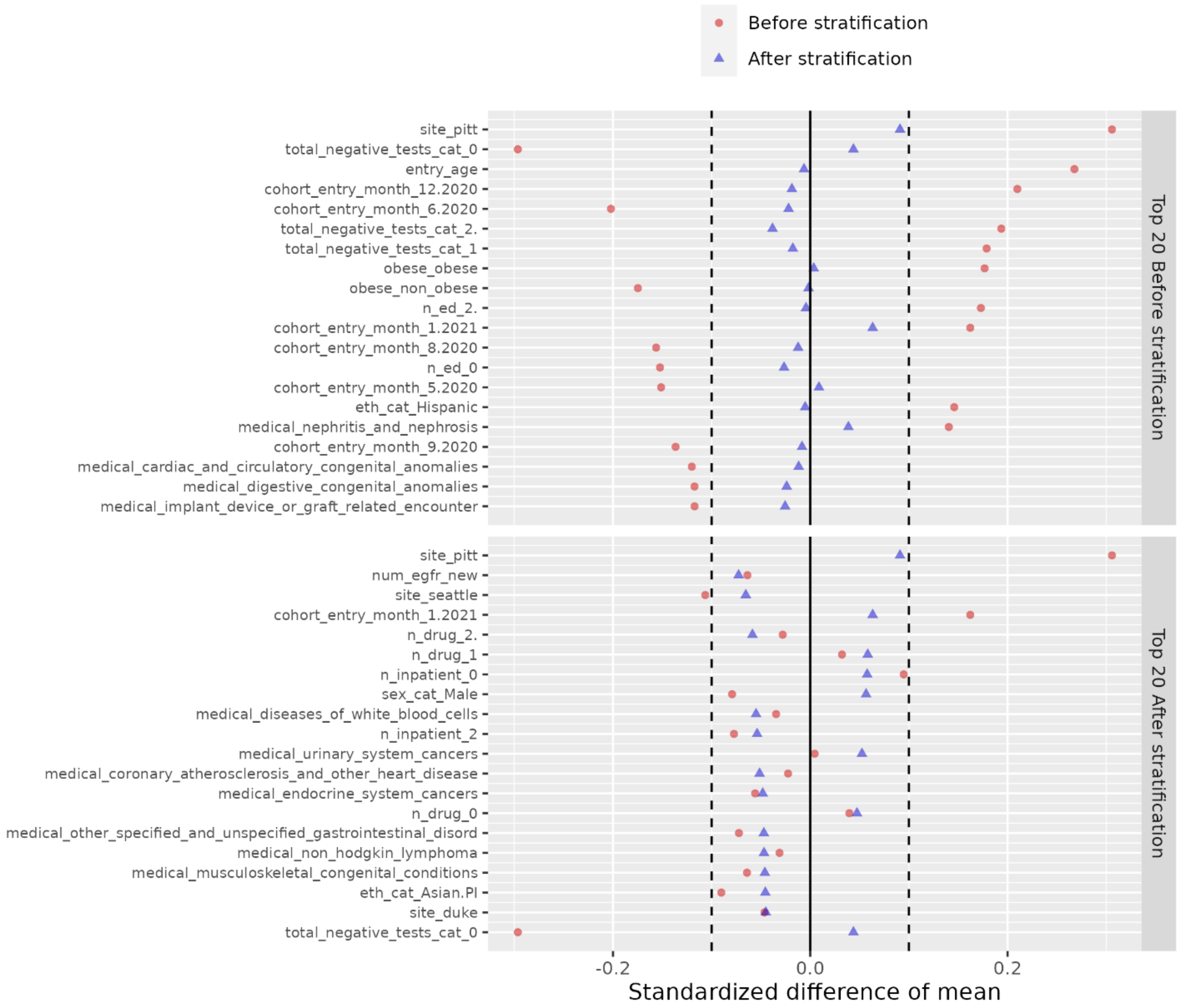

Supplement Figure 20: Patient characteristic balance before and after large-scale PS stratification with 6 strata for children and adolescents with CKD in Delta period. The upper panel displays the top 20 covariates with the largest standardized difference of means before stratification, while the lower panel displays the top 20 covariates with the largest standardized difference of means after stratification.

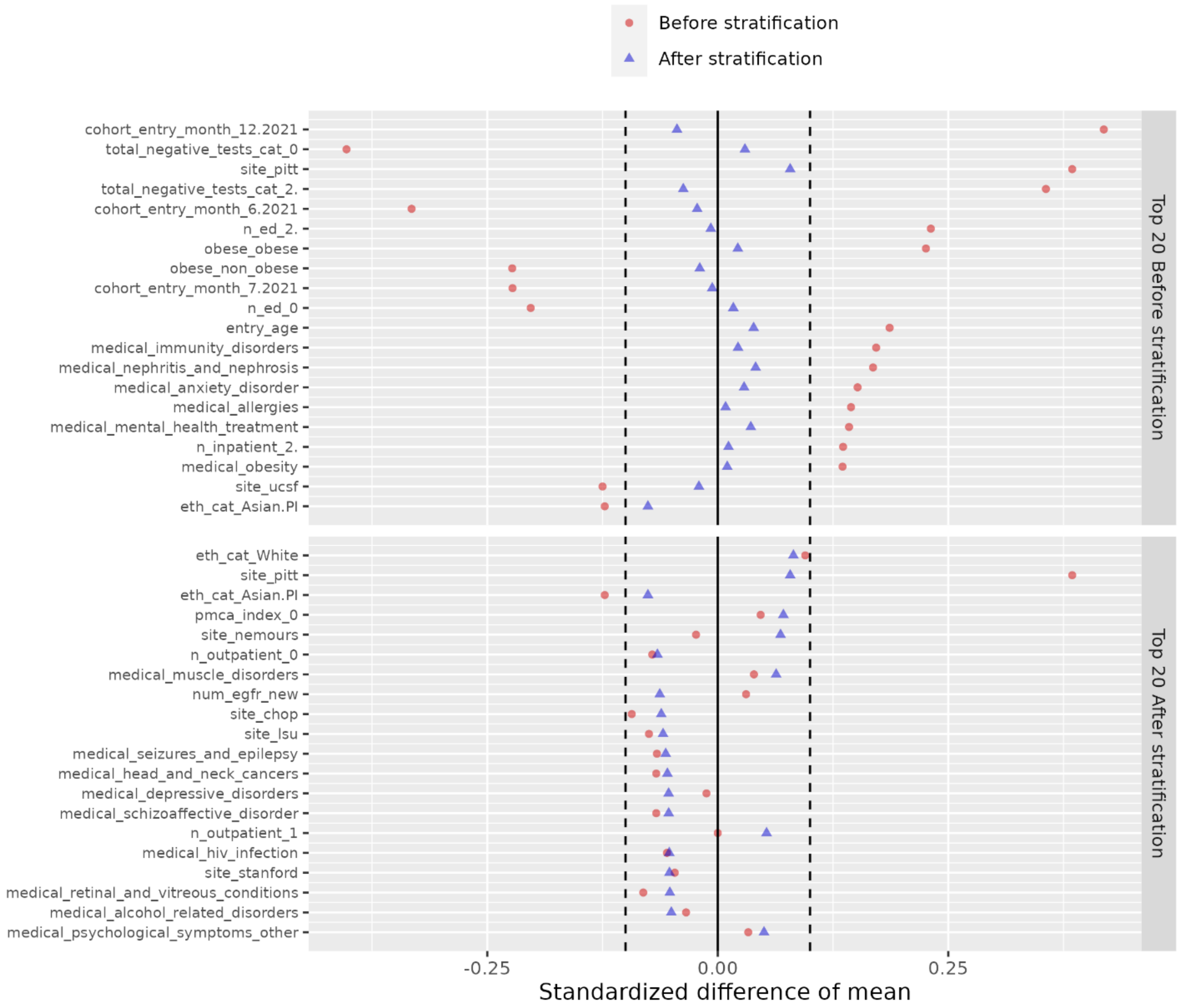

Supplement Figure 21: Patient characteristic balance before and after large-scale PS stratification with 6 strata for children and adolescents with CKD in Omicron period. The upper panel displays the top 20 covariates with the largest standardized difference of means before stratification, while the lower panel displays the top 20 covariates with the largest standardized difference of means after stratification.

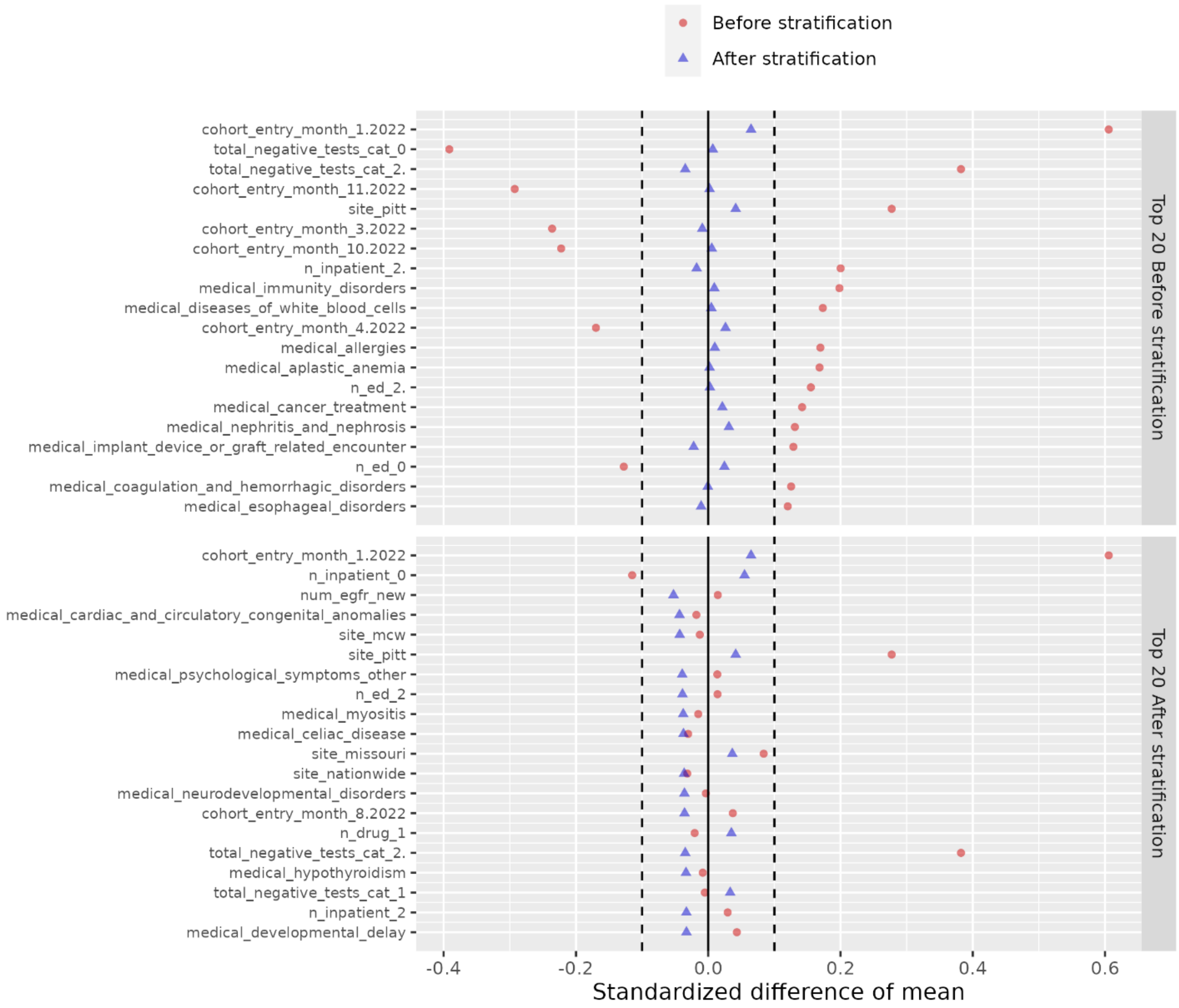

Supplement Figure 22: Patient characteristic balance before and after large-scale PS stratification with 6 strata for children and adolescents with no AKI or CKD in pre-Delta period. The upper panel displays the top 20 covariates with the largest standardized difference of means before stratification, while the lower panel displays the top 20 covariates with the largest standardized difference of means after stratification.

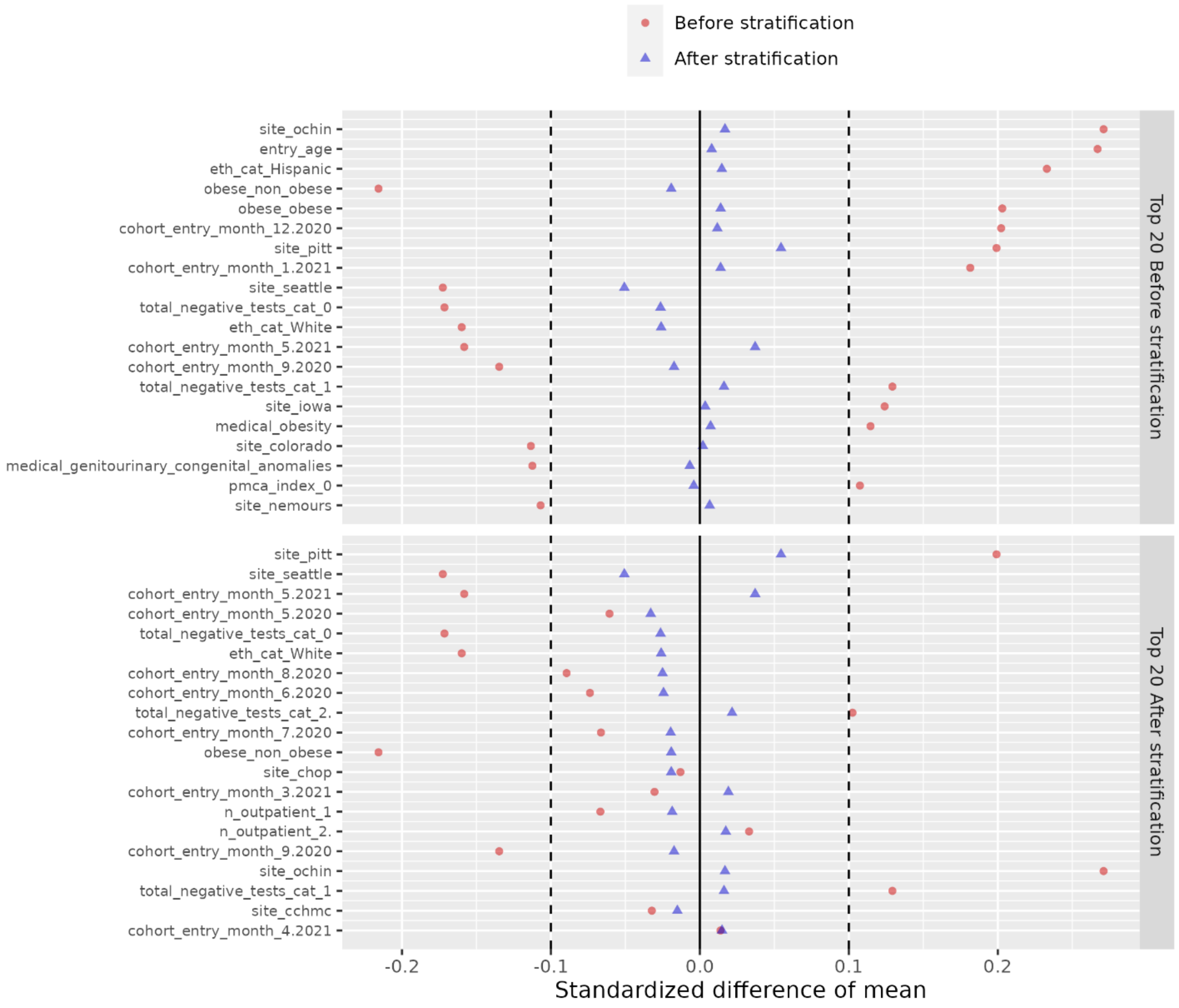

Supplement Figure 23: Patient characteristic balance before and after large-scale PS stratification with 6 strata for children and adolescents with no AKI or CKD in Delta period. The upper panel displays the top 20 covariates with the largest standardized difference of means before stratification, while the lower panel displays the top 20 covariates with the largest standardized difference of means after stratification.

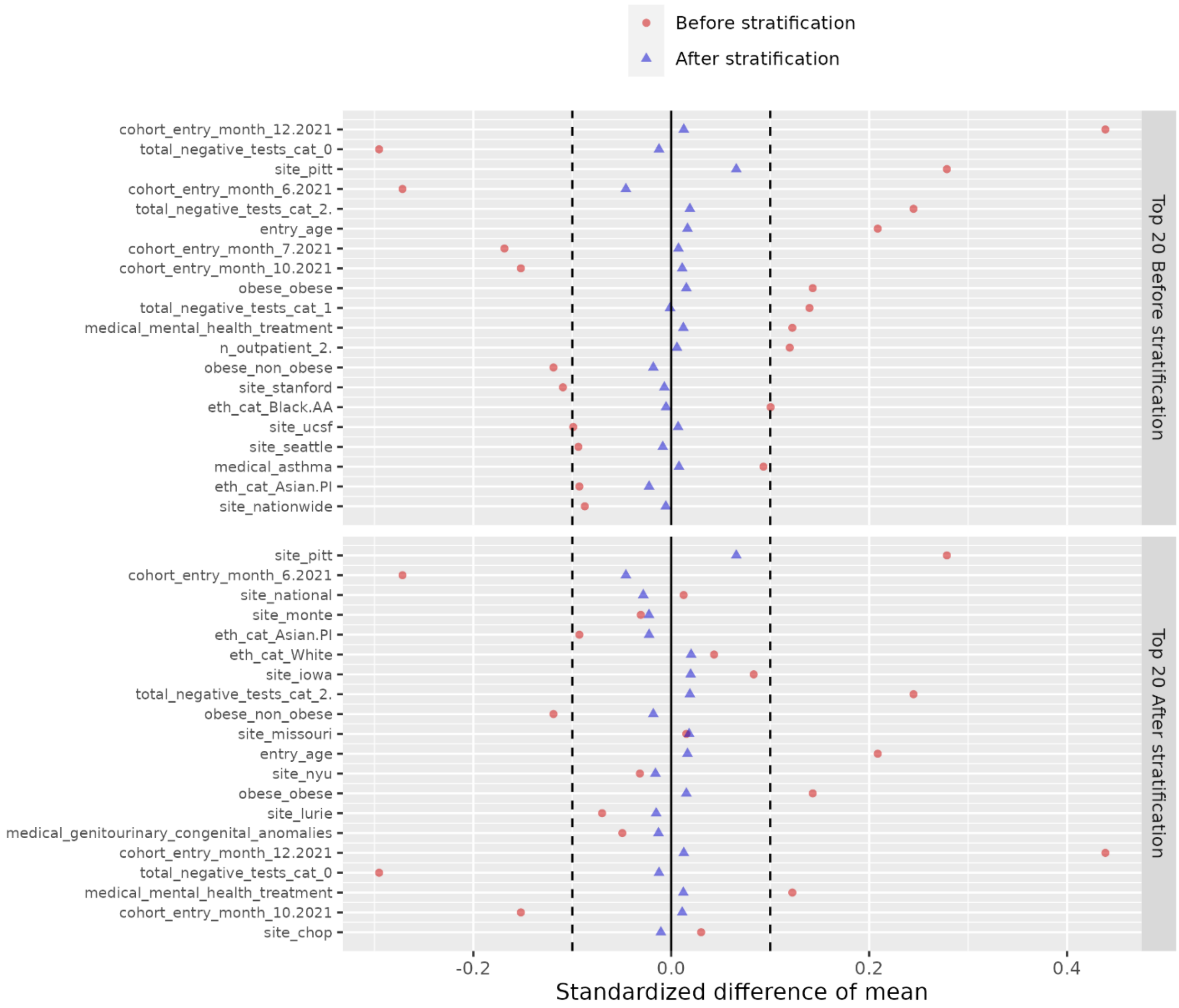

Supplement Figure 24: Patient characteristic balance before and after large-scale PS stratification with 6 strata for children and adolescents with no AKI or CKD in Omicron period. The upper panel displays the top 20 covariates with the largest standardized difference of means before stratification, while the lower panel displays the top 20 covariates with the largest standardized difference of means after stratification.

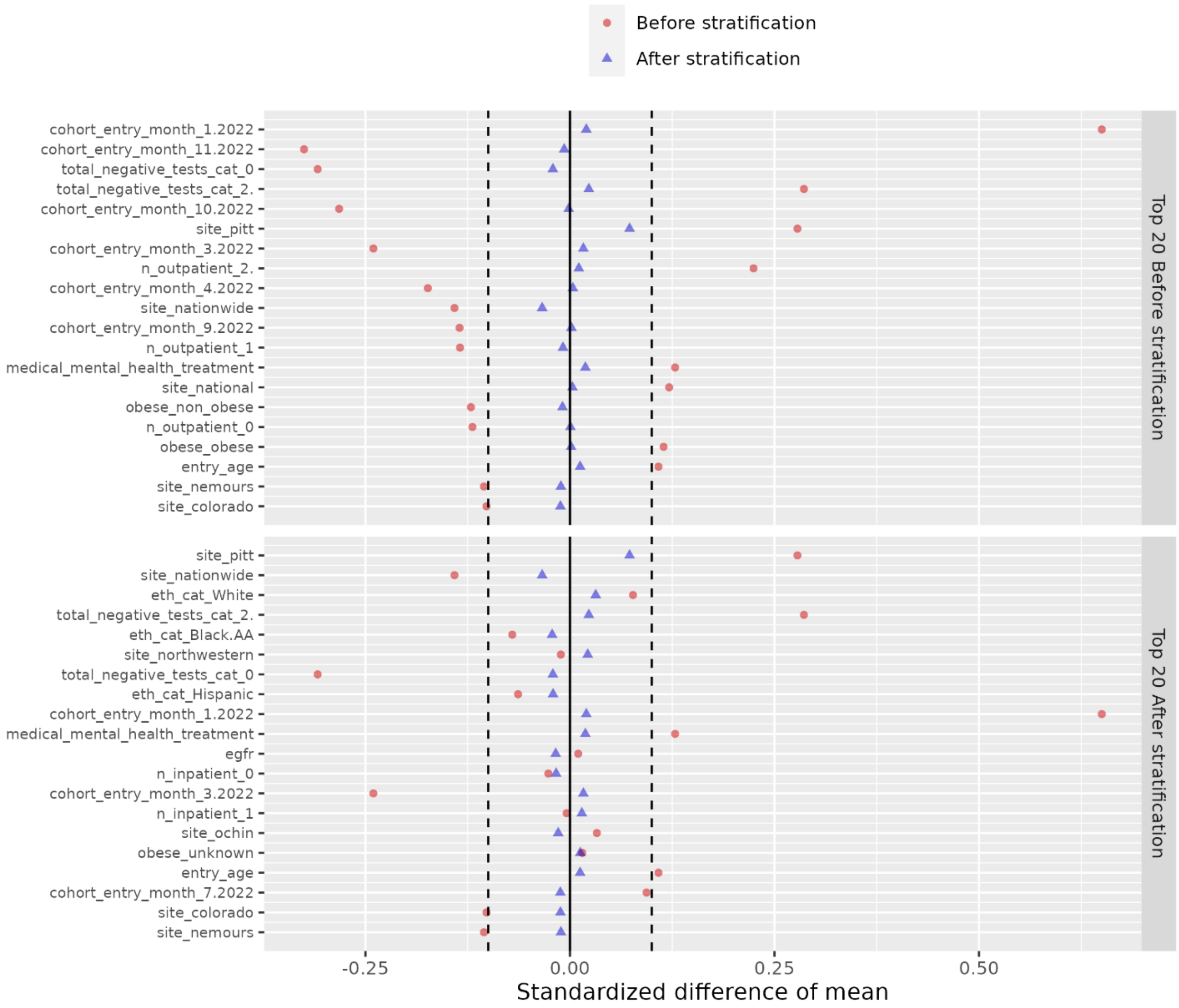

C. Hazard ratio of COVID-19 positive group compared to control group

Supplement Table 2: Estimated hazard ratio in kidney function outcomes between the COVID-19 positive cohort and the control cohort for children and adolescents in pre-Delta period

Dominant Variant: Pre Delta	COVID-19 Groups Event (%)	Control Group Event (%)	HR (95% CI)
No CKD or AKI			
CKD 2+ (days 28-729)	684 (0.51%)	2310 (0.43%)	1.19 [1.12, 1.26]
CKD 2+ (majority not returned to 90 and above) (days 28-729)	588 (0.44%)	1885 (0.35%)	1.21 [1.14, 1.29]
CKD 2+ (not returned to 90 and above) (days 28-729)	521 (0.39%)	1625 (0.3%)	1.21 [1.12, 1.31]
CKD 3+ (days 28-729)	45 (0.03%)	159 (0.03%)	1.45 [1.00, 2.10]
CKD 3+ (majority not returned to 90 and above) (days 28-729)	32 (0.02%)	110 (0.02%)	1.46 [1.05, 2.04]
CKD 3+ (not returned to 90 and above) (days 28-729)	35 (0.03%)	134 (0.02%)	1.28 [0.93, 1.75]
CKD 3+ (not returned to 60 and above) (days 28-729)	27 (0.02%)	93 (0.02%)	1.37 [0.87, 2.16]
CKD			
Composite outcome (days 28-179)	243 (13.03%)	827 (11.34%)	1.19 [1.00, 1.42]
Composite outcome (days 180-729)	410 (21.98%)	1399 (19.18%)	1.19 [1.07, 1.33]
eGFR decline of 50% or more (days 28-179)	43 (2.31%)	196 (2.69%)	0.87 [0.73, 1.04]
eGFR decline of 50% or more (days 180-729)	74 (3.97%)	325 (4.46%)	1.01 [0.77, 1.33]
eGFR decline of 40% or more (days 28-179)	114 (6.11%)	442 (6.06%)	1.00 [0.78, 1.27]
eGFR decline of 40% or more (days 180-729)	217 (11.64%)	740 (10.15%)	1.19 [1.04, 1.35]
eGFR decline of 30% or more (days 28-179)	238 (12.76%)	827 (11.34%)	1.16 [0.97, 1.38]
eGFR decline of 30% or more (days 180-729)	407 (21.82%)	1399 (19.18%)	1.18 [1.06, 1.31]
AKI			
Composite outcome (days 90-179)	53 (4.64%)	200 (3.48%)	1.22 [1.03, 1.44]
Composite outcome (days 180-729)	130 (11.37%)	419 (7.28%)	1.32 [1.15, 1.52]
eGFR decline of 50% or more (days 180-729)	60 (5.25%)	146 (2.54%)	2.06 [1.69, 2.51]
eGFR decline of 40% or more (days 90-179)	29 (2.54%)	122 (2.12%)	1.15 [0.84, 1.56]
eGFR decline of 40% or more (days 180-729)	90 (7.87%)	263 (4.57%)	1.49 [1.40, 1.59]
eGFR decline of 30% or more (days 90-179)	52 (4.55%)	200 (3.48%)	1.20 [1.03, 1.41]
eGFR decline of 30% or more (days 180-729)	127 (11.11%)	419 (7.28%)	1.28 [1.09, 1.50]
CKD 2+ (days 28-729)	71 (6.21%)	271 (4.71%)	1.08 [0.90, 1.30]
CKD 3+ (days 28-729)	22 (1.92%)	53 (0.92%)	2.03 [1.23, 3.37]
CKD 3+ (not returned to 90 and above) (days 28-729)	20 (1.75%)	47 (0.82%)	1.99 [1.11, 3.55]

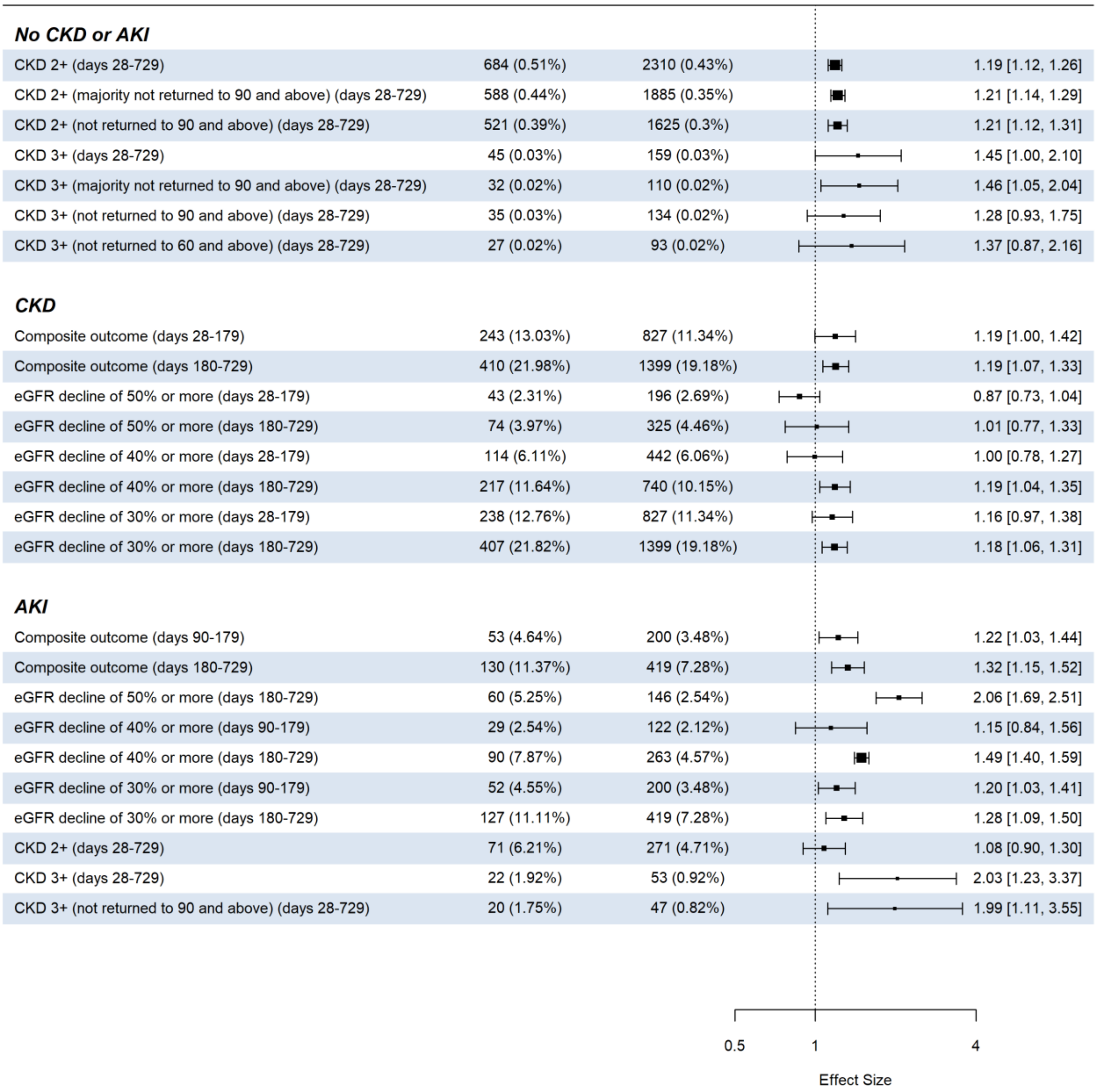

Supplement Table 3: Estimated hazard ratio in kidney function outcomes between the COVID-19 positive cohort and the control cohort for children and adolescents in Delta period

Dominant Variant: Delta	COVID-19 Groups Event (%)	Control Group Event (%)	HR (95% CI)
No CKD or AKI			
CKD 2+ (days 28-729)	420 (0.36%)	1027 (0.24%)	1.32 [1.20, 1.46]
CKD 2+ (majority not returned to 90 and above) (days 28-729)	364 (0.31%)	878 (0.21%)	1.33 [1.21, 1.48]
CKD 2+ (not returned to 90 and above) (days 28-729)	343 (0.29%)	818 (0.19%)	1.33 [1.21, 1.46]
CKD 3+ (days 28-729)	34 (0.03%)	90 (0.02%)	1.43 [0.96, 2.12]
CKD 3+ (majority not returned to 90 and above) (days 28-729)	23 (0.02%)	69 (0.02%)	1.29 [0.74, 2.25]
CKD 3+ (not returned to 90 and above) (days 28-729)	30 (0.03%)	81 (0.02%)	1.36 [0.87, 2.13]
CKD 3+ (not returned to 60 and above) (days 28-729)	20 (0.02%)	67 (0.02%)	1.11 [0.60, 2.05]
CKD			
Composite outcome (days 28-179)	205 (13.43%)	406 (11.27%)	1.19 [1.07, 1.34]
Composite outcome (days 180-729)	295 (19.33%)	686 (19.04%)	1.04 [0.88, 1.23]
eGFR decline of 50% or more (days 180-729)	63 (4.13%)	152 (4.22%)	1.17 [0.76, 1.80]
eGFR decline of 40% or more (days 28-179)	109 (7.14%)	224 (6.22%)	1.22 [0.88, 1.69]
eGFR decline of 40% or more (days 180-729)	155 (10.16%)	360 (9.99%)	1.12 [0.93, 1.34]
eGFR decline of 30% or more (days 28-179)	202 (13.24%)	406 (11.27%)	1.18 [1.05, 1.32]
eGFR decline of 30% or more (days 180-729)	292 (19.13%)	686 (19.04%)	1.03 [0.87, 1.21]
AKI			
Composite outcome (days 90-179)	50 (5.03%)	108 (3.07%)	1.41 [0.91, 2.17]
Composite outcome (days 180-729)	107 (10.75%)	207 (5.88%)	1.40 [1.07, 1.82]
eGFR decline of 40% or more (days 90-179)	35 (3.52%)	70 (1.99%)	1.63 [1.00, 2.66]
eGFR decline of 30% or more (days 90-179)	50 (5.03%)	108 (3.07%)	1.41 [0.91, 2.17]
eGFR decline of 30% or more (days 180-729)	106 (10.65%)	207 (5.88%)	1.39 [1.05, 1.83]

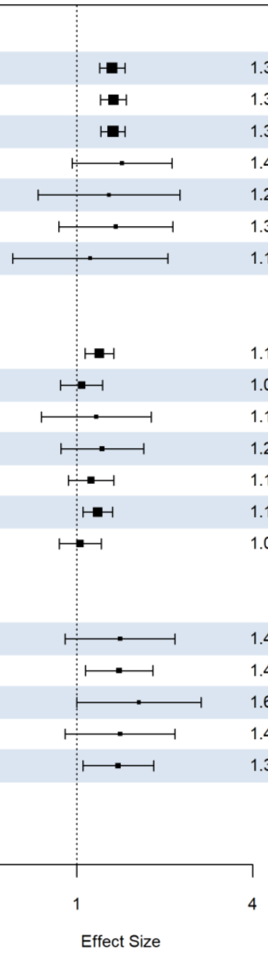

Supplement Table 4: Estimated hazard ratio in kidney function outcomes between the COVID-19 positive cohort and the control cohort for children and adolescents in Omicron period

Dominant Variant: Omicron	COVID-19 Groups Event (%)	Control Group Event (%)	HR (95% CI)
No CKD or AKI			
CKD 2+ (days 28-729)	465 (0.21%)	563 (0.13%)	1.13 [1.06, 1.21]
CKD 2+ (majority not returned to 90 and above) (days 28-729)	402 (0.18%)	502 (0.12%)	1.09 [0.96, 1.23]
CKD 2+ (not returned to 90 and above) (days 28-729)	386 (0.17%)	466 (0.11%)	1.12 [0.96, 1.31]
CKD 3+ (days 28-729)	53 (0.02%)	60 (0.01%)	1.34 [0.93, 1.92]
CKD			
Composite outcome (days 28-179)	457 (14.78%)	513 (12.5%)	1.15 [1.01, 1.31]
Composite outcome (days 180-729)	462 (14.94%)	431 (10.5%)	1.22 [1.07, 1.39]
eGFR decline of 50% or more (days 28-179)	103 (3.33%)	119 (2.9%)	1.02 [0.78, 1.35]
eGFR decline of 50% or more (days 180-729)	109 (3.53%)	98 (2.39%)	1.33 [1.00, 1.75]
eGFR decline of 40% or more (days 28-179)	261 (8.44%)	291 (7.09%)	1.12 [0.95, 1.32]
eGFR decline of 40% or more (days 180-729)	243 (7.86%)	245 (5.97%)	1.13 [0.91, 1.40]
eGFR decline of 30% or more (days 28-179)	454 (14.68%)	513 (12.5%)	1.14 [1.00, 1.31]
eGFR decline of 30% or more (days 180-729)	460 (14.88%)	431 (10.5%)	1.22 [1.07, 1.39]
AKI			
Composite outcome (days 90-179)	103 (5.97%)	169 (3.36%)	1.29 [1.00, 1.68]
Composite outcome (days 180-729)	133 (7.71%)	170 (3.38%)	1.24 [1.06, 1.45]
eGFR decline of 40% or more (days 180-729)	91 (5.27%)	111 (2.21%)	1.34 [1.15, 1.57]
eGFR decline of 30% or more (days 90-179)	101 (5.85%)	169 (3.36%)	1.26 [0.98, 1.62]
eGFR decline of 30% or more (days 180-729)	132 (7.65%)	170 (3.38%)	1.22 [1.03, 1.45]
CKD 2+ (days 28-729)	64 (3.71%)	127 (2.52%)	0.92 [0.82, 1.04]
CKD 2+ (majority not returned to 90 and above) (days 28-729)	59 (3.42%)	117 (2.33%)	0.93 [0.84, 1.03]
CKD 2+ (not returned to 90 and above) (days 28-729)	55 (3.19%)	105 (2.09%)	0.94 [0.81, 1.09]

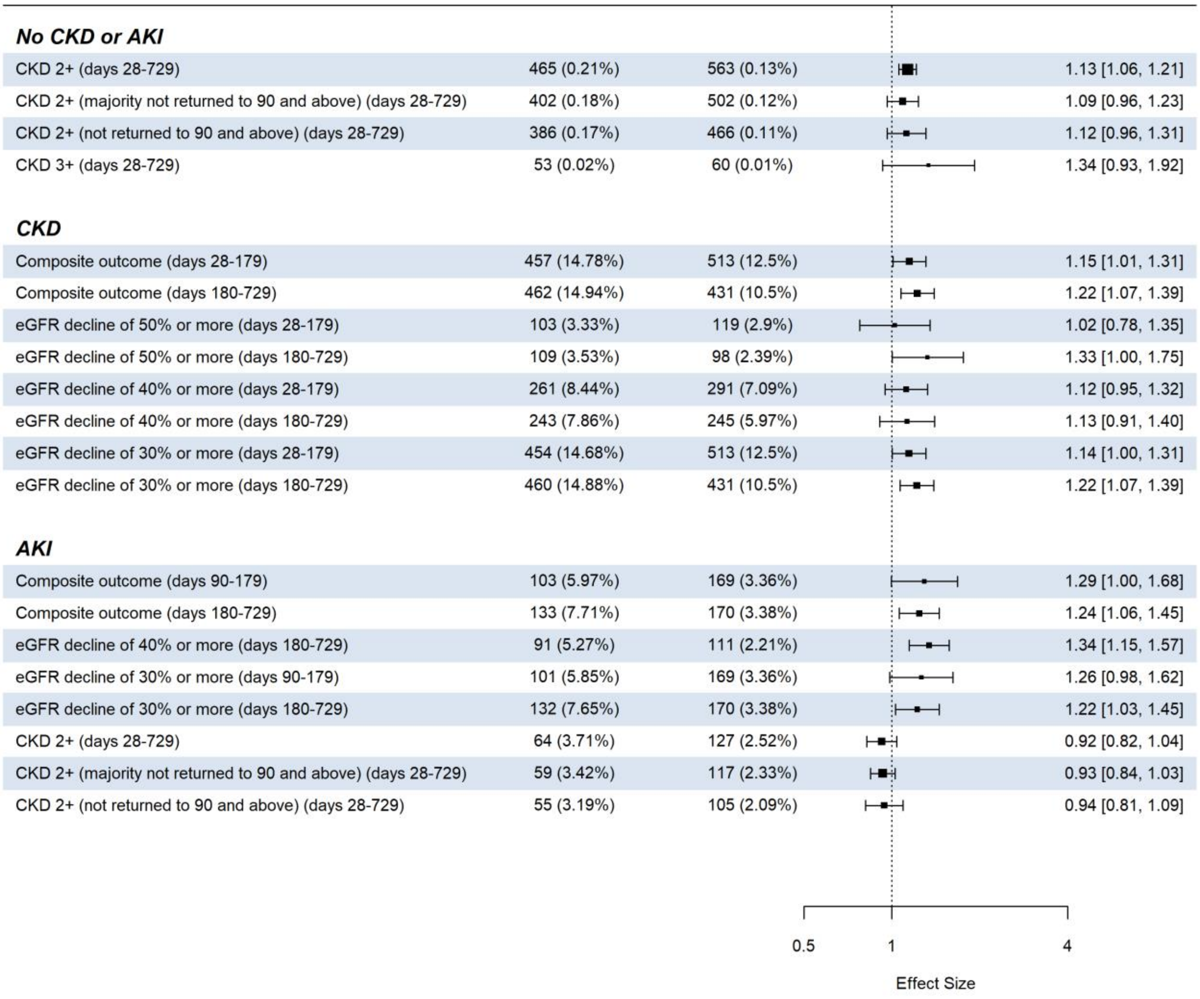

eAppendix 5. Sensitivity Analysis for Sex

We conducted sensitivity analyses on both cohorts stratified by sex to investigate the potential effect of sex on the risk of kidney function outcomes. We performed the same PS stratification procedure and Section 5used Cox proportional hazard model to estimate the hazard ratio.

A. Empirical equipoise assessment

Supplement Figure 25: Preference score distributions of COVID-19 positive and negative groups for female children and adolescents with AKI. A greater convergence of these distributions indicates a higher similarity in the predicted likelihood of being infected between the COVID-19 positive (red) and negative (blue) participants.

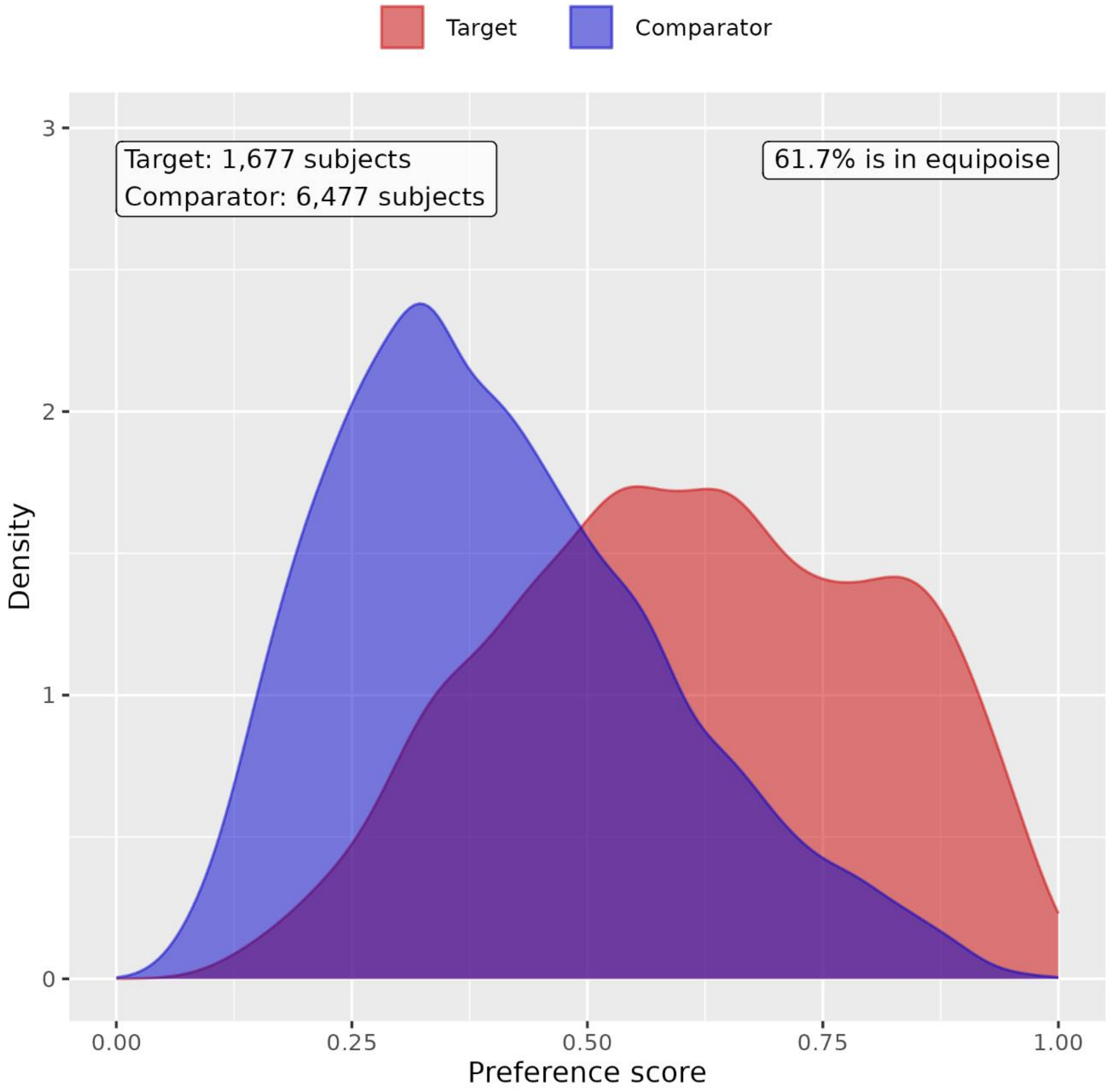

Supplement Figure 26: Preference score distributions of COVID-19 positive and negative groups for male children and adolescents with AKI. A greater convergence of these distributions indicates a higher similarity in the predicted likelihood of being infected between the COVID-19 positive (red) and negative (blue) participants.

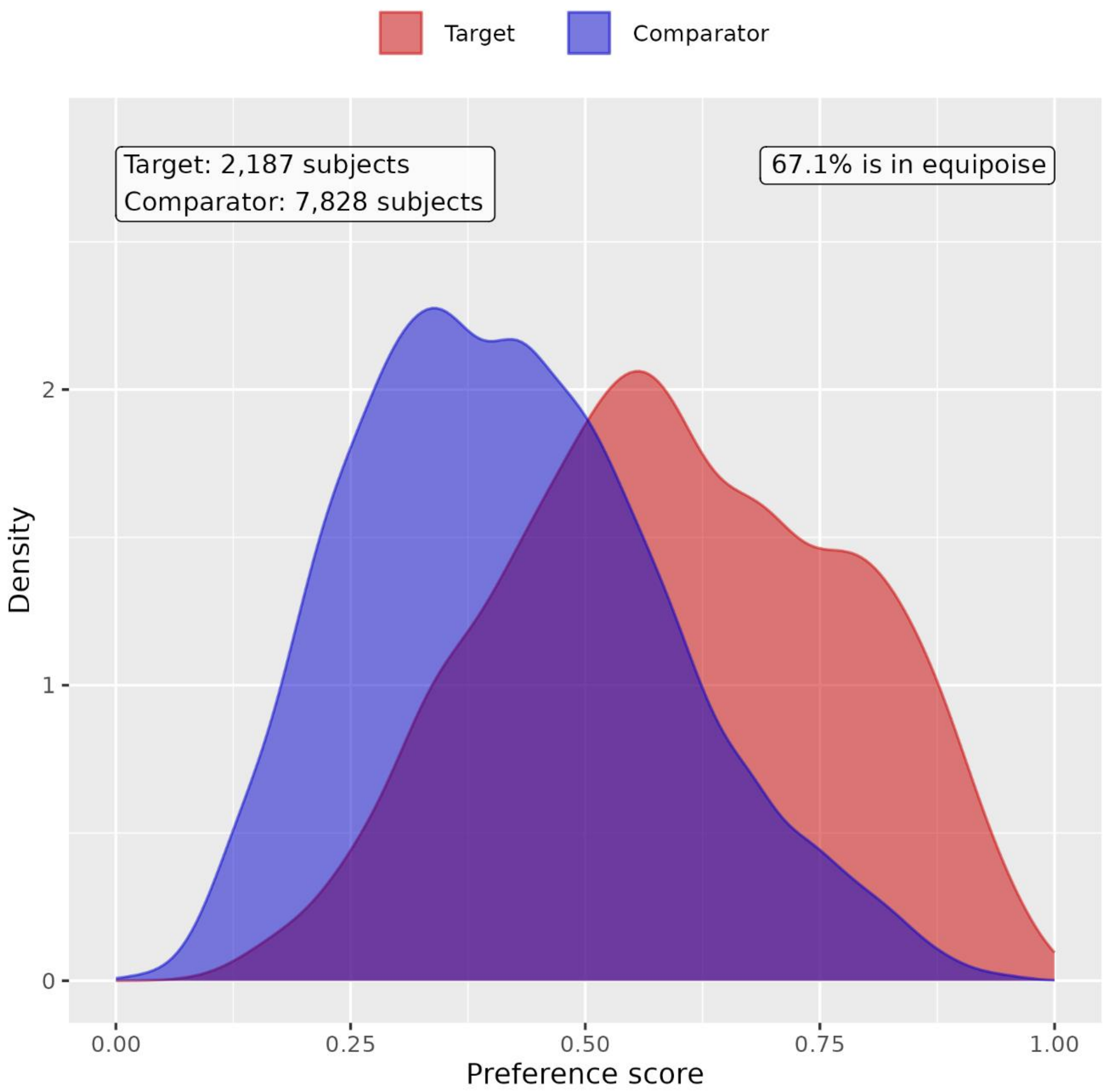

Supplement Figure 27: Preference score distributions of COVID-19 positive and negative groups for female children and adolescents with CKD. A greater convergence of these distributions indicates a higher similarity in the predicted likelihood of being infected between the COVID-19 positive (red) and negative (blue) participants.

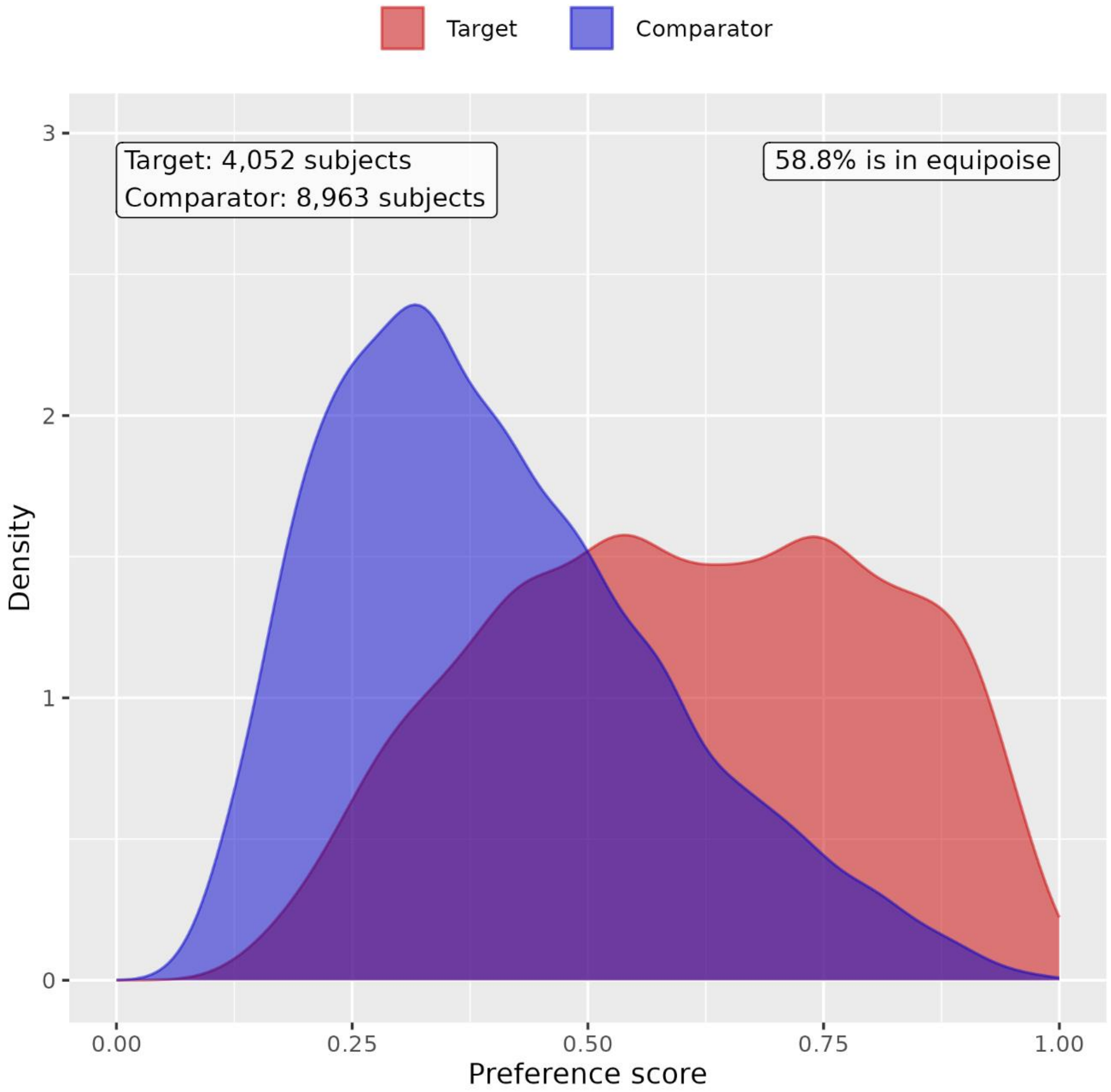

Supplement Figure 28: Preference score distributions of COVID-19 positive and control groups for male children and adolescents with CKD. A greater convergence of these distributions indicates a higher similarity in the predicted likelihood of being infected between the COVID-19 positive (red) and negative (blue) participants.

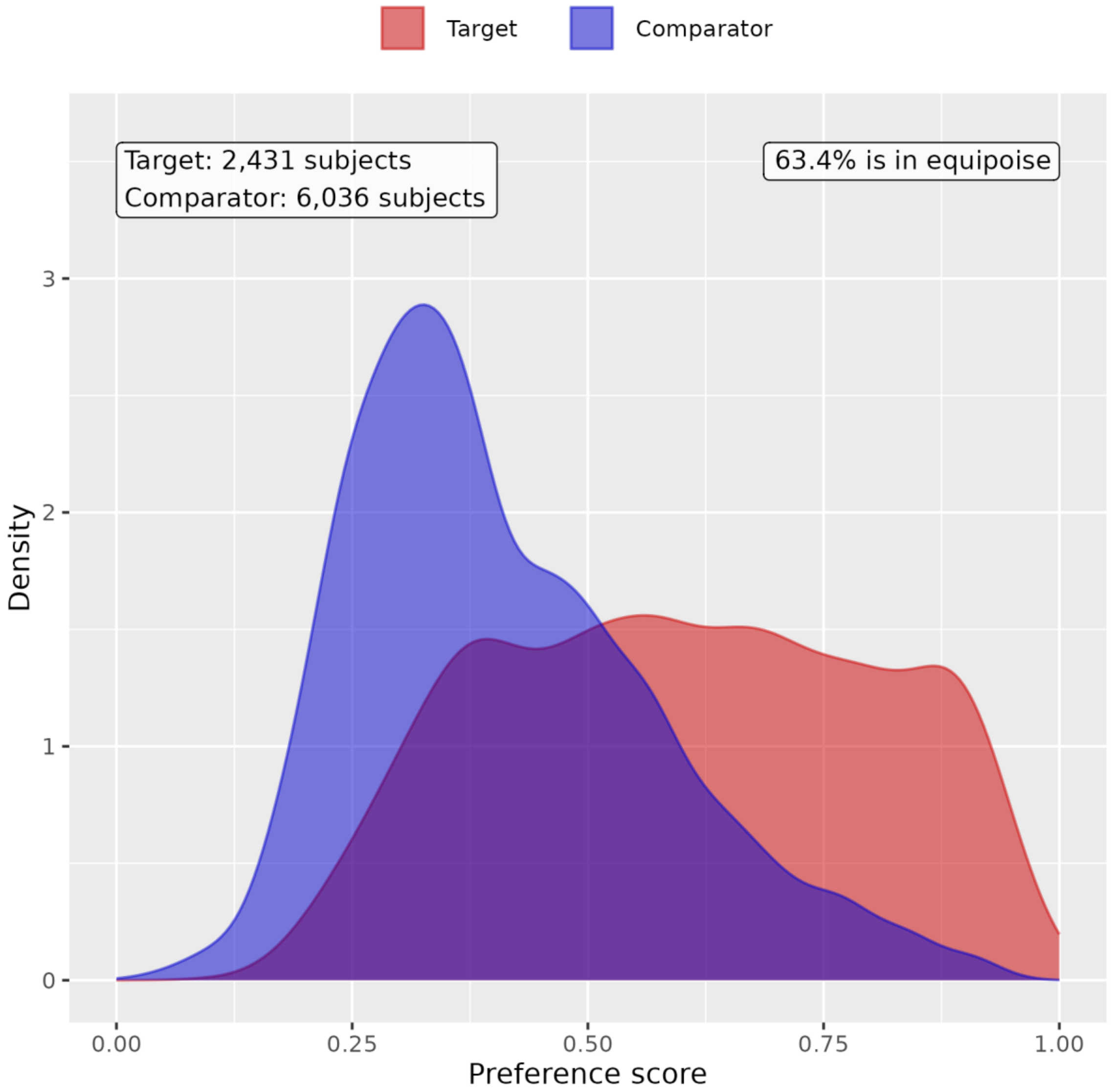

Supplement Figure 29: Preference score distributions of COVID-19 positive and control groups for female children and adolescents with no AKI or CKD. A greater convergence of these distributions indicates a higher similarity in the predicted likelihood of being infected between the COVID-19 positive (red) and negative (blue) participants.

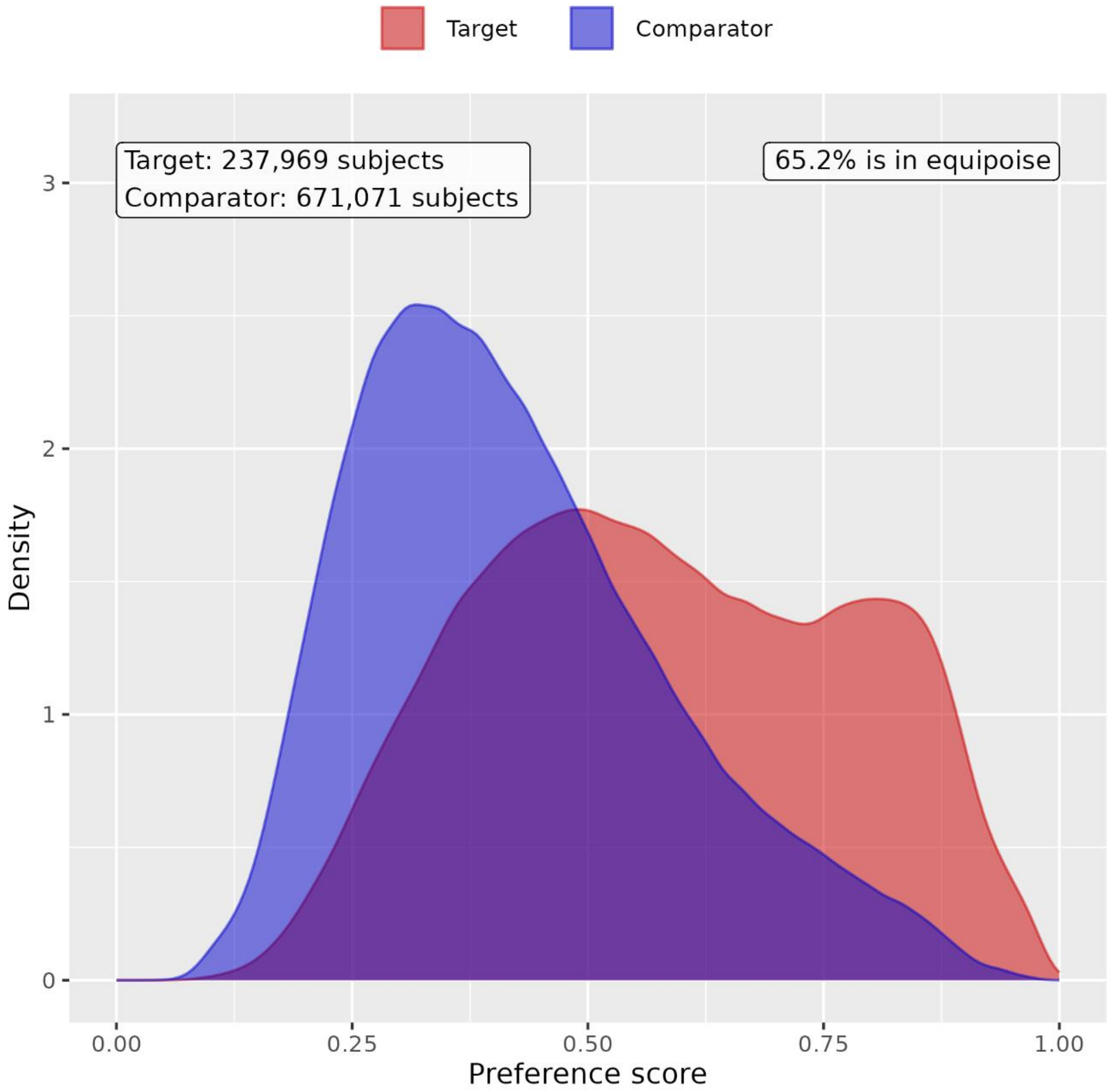

Supplement Figure 30: Preference score distributions of COVID-19 positive and control groups for male children and adolescents with no AKI or CKD. A greater convergence of these distributions indicates a higher similarity in the predicted likelihood of being infected between the COVID-19 positive (red) and negative (blue) participants.

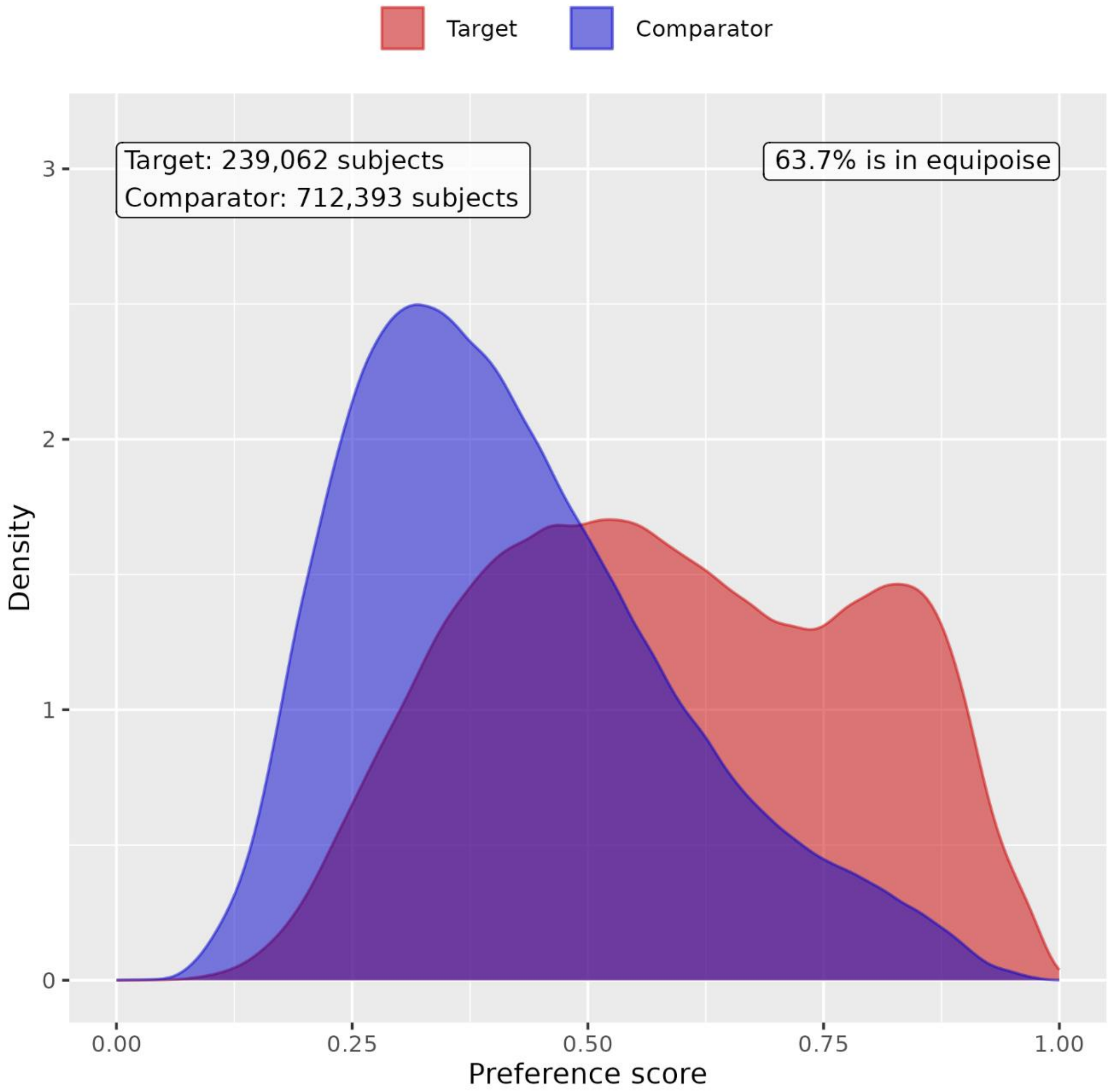

B. Patient characteristic balance

Supplement Figure 31: Patient characteristic balance before and after large-scale PS stratification with 6 strata for female children and adolescents with AKI. The upper panel displays the top 20 covariates with the largest standardized difference of means before stratification, while the lower panel displays the top 20 covariates with the largest standardized difference of means after stratification.

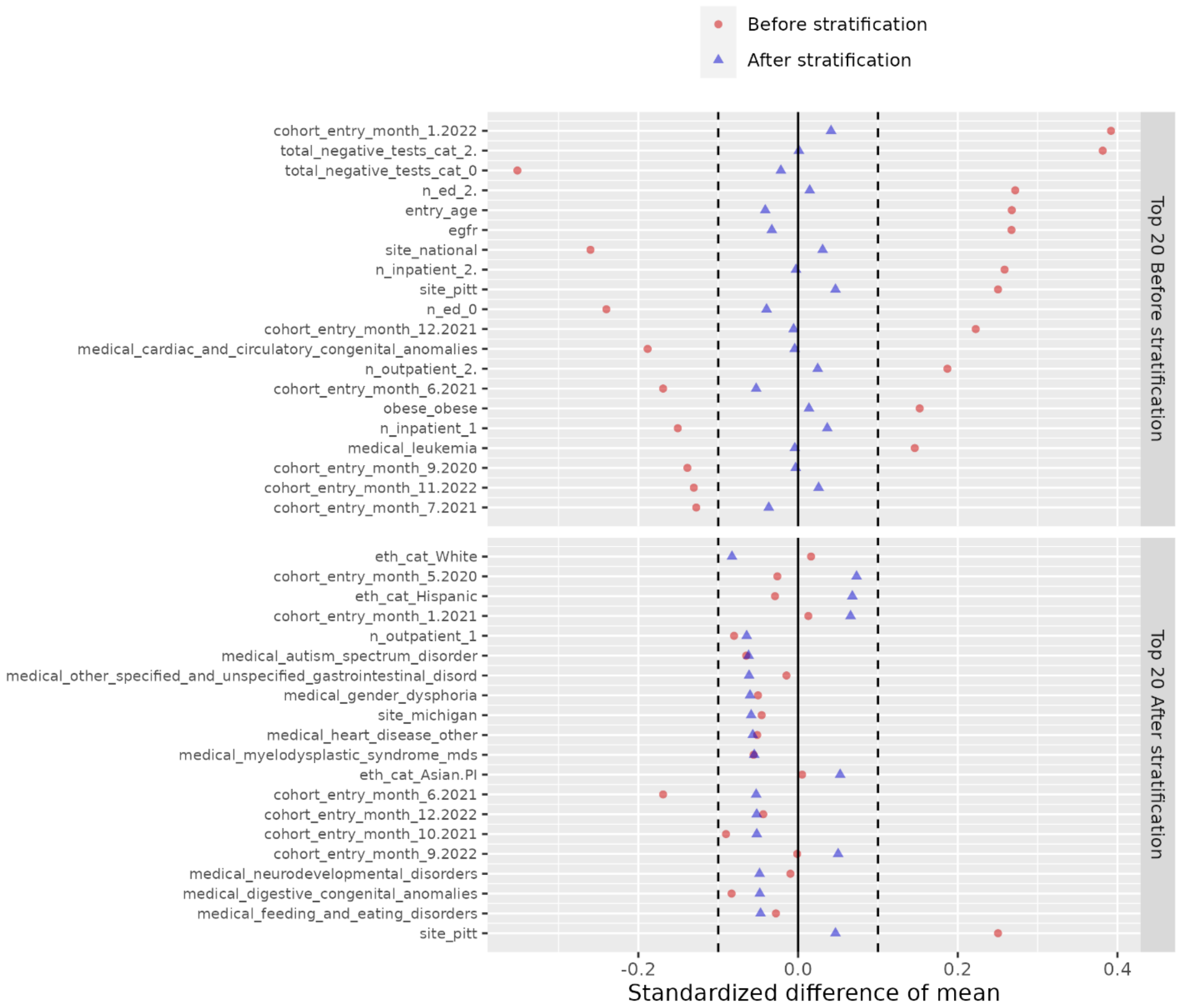

Supplement Figure 32: Patient characteristic balance before and after large-scale PS stratification with 6 strata for male children and adolescents with AKI. The upper panel displays the top 20 covariates with the largest standardized difference of means before stratification, while the lower panel displays the top 20 covariates with the largest standardized difference of means after stratification.

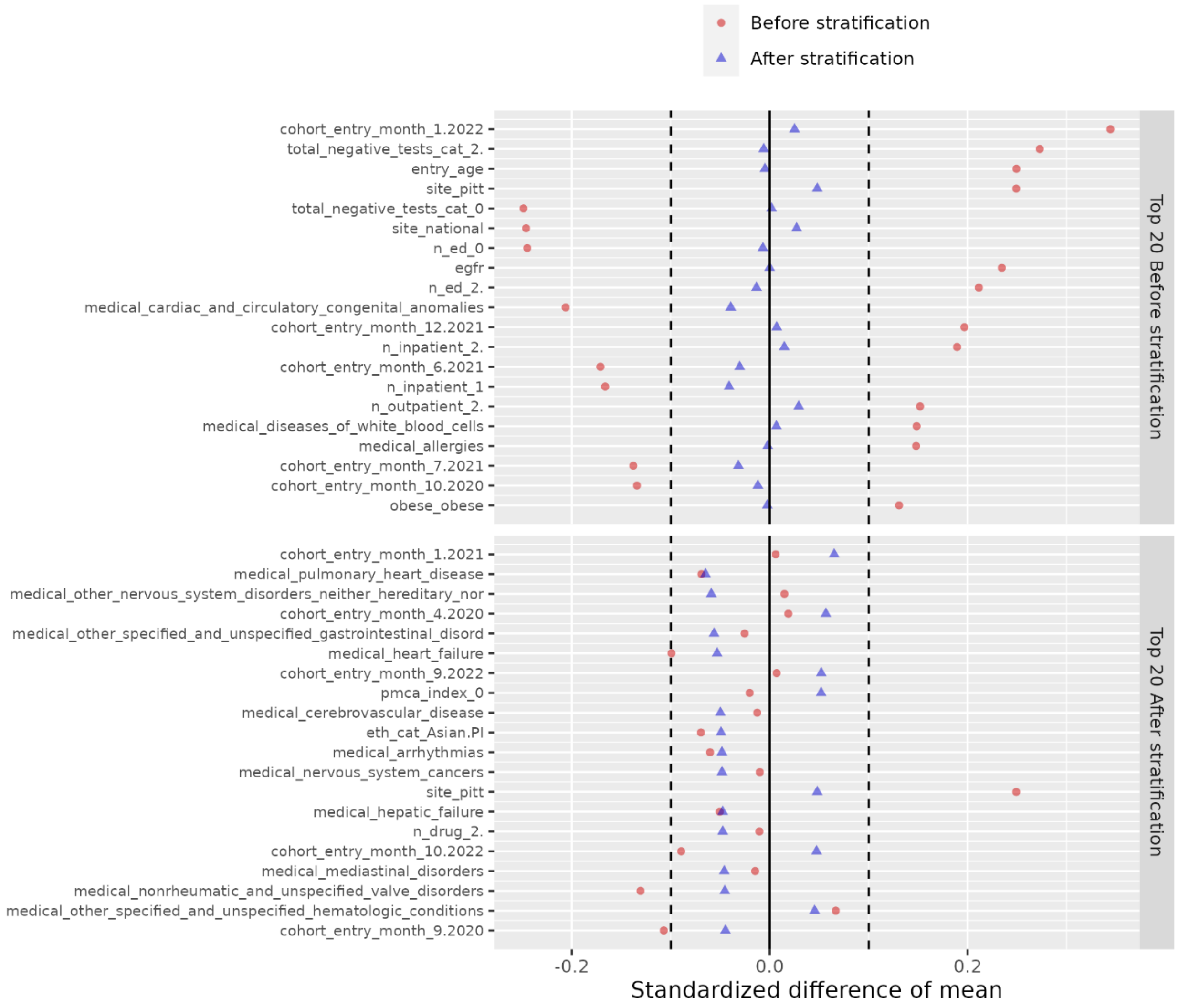

Supplement Figure 33: Patient characteristic balance before and after large-scale PS stratification with 6 strata for female children and adolescents with CKD. The upper panel displays the top 20 covariates with the largest standardized difference of means before stratification, while the lower panel displays the top 20 covariates with the largest standardized difference of means after stratification.

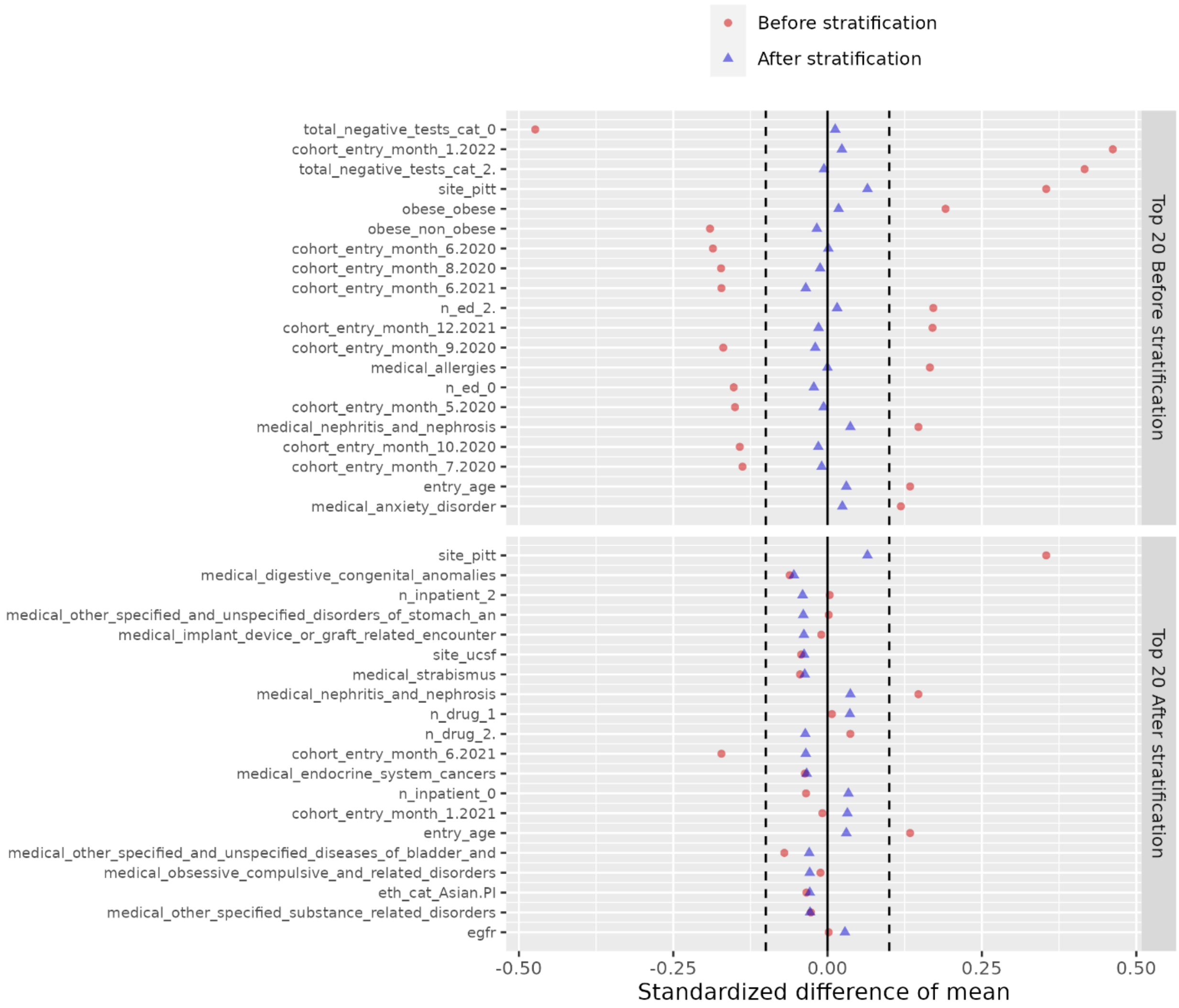

Supplement Figure 34: Patient characteristic balance before and after large-scale PS stratification with 6 strata for male children and adolescents with CKD. The upper panel displays the top 20 covariates with the largest standardized difference of means before stratification, while the lower panel displays the top 20 covariates with the largest standardized difference of means after stratification.

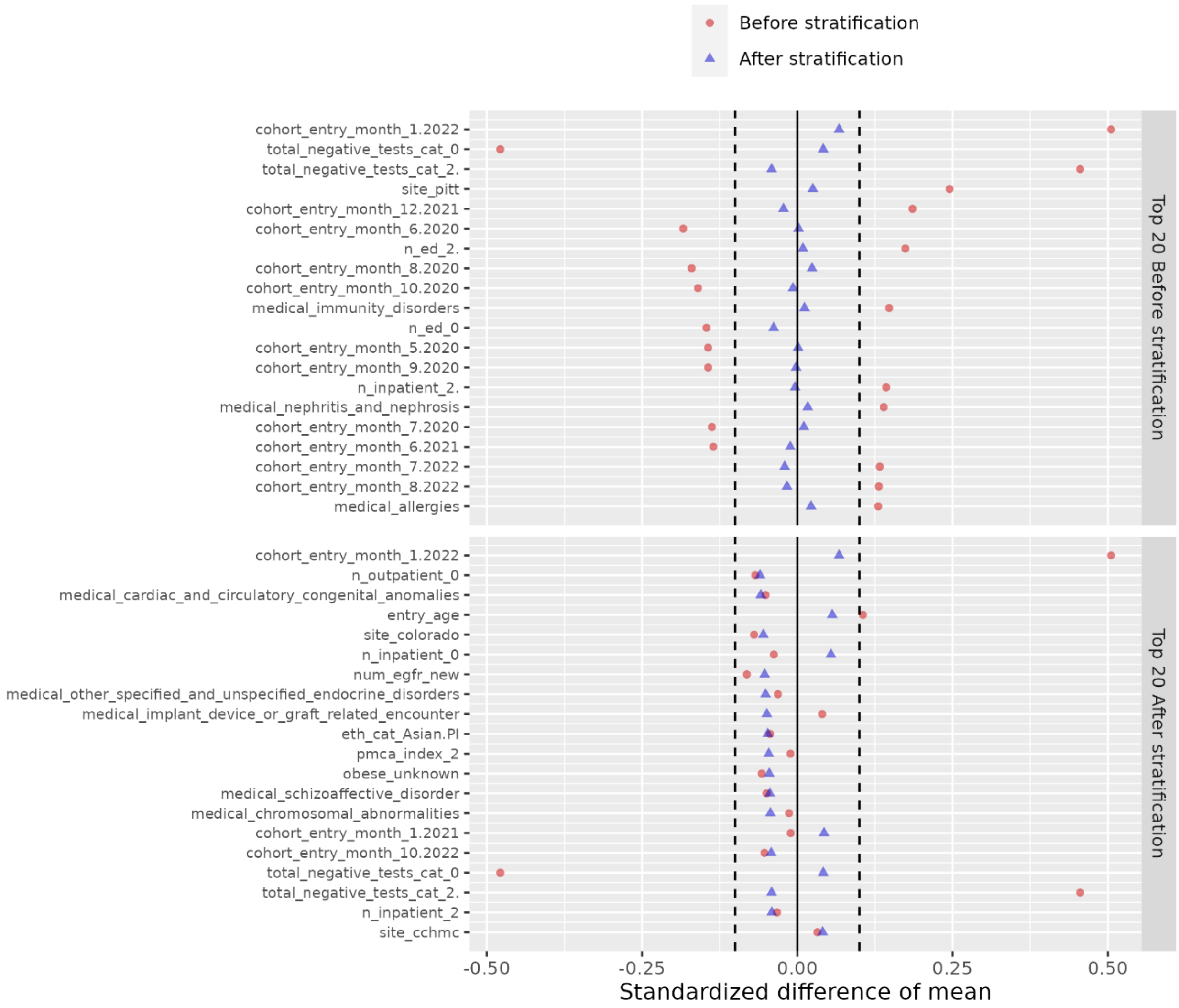

Supplement Figure 35: Patient characteristic balance before and after large-scale PS stratification with 6 strata for female children and adolescents with no AKI or CKD. The upper panel displays the top 20 covariates with the largest standardized difference of means before stratification, while the lower panel displays the top 20 covariates with the largest standardized difference of means after stratification.

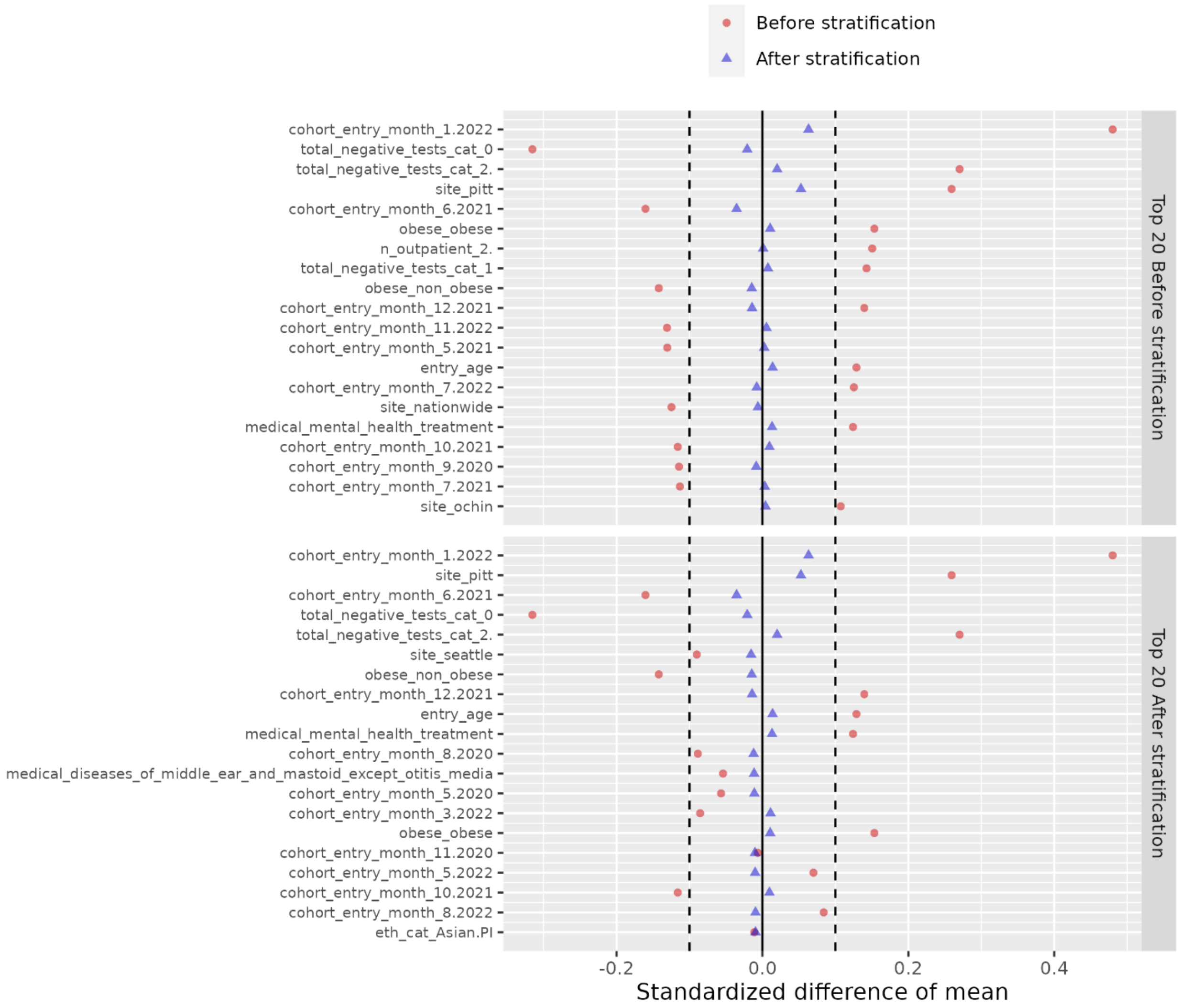

Supplement Figure 36: Patient characteristic balance before and after large-scale PS stratification with 6 strata for male children and adolescents with no AKI or CKD. The upper panel displays the top 20 covariates with the largest standardized difference of means before stratification, while the lower panel displays the top 20 covariates with the largest standardized difference of means after stratification.

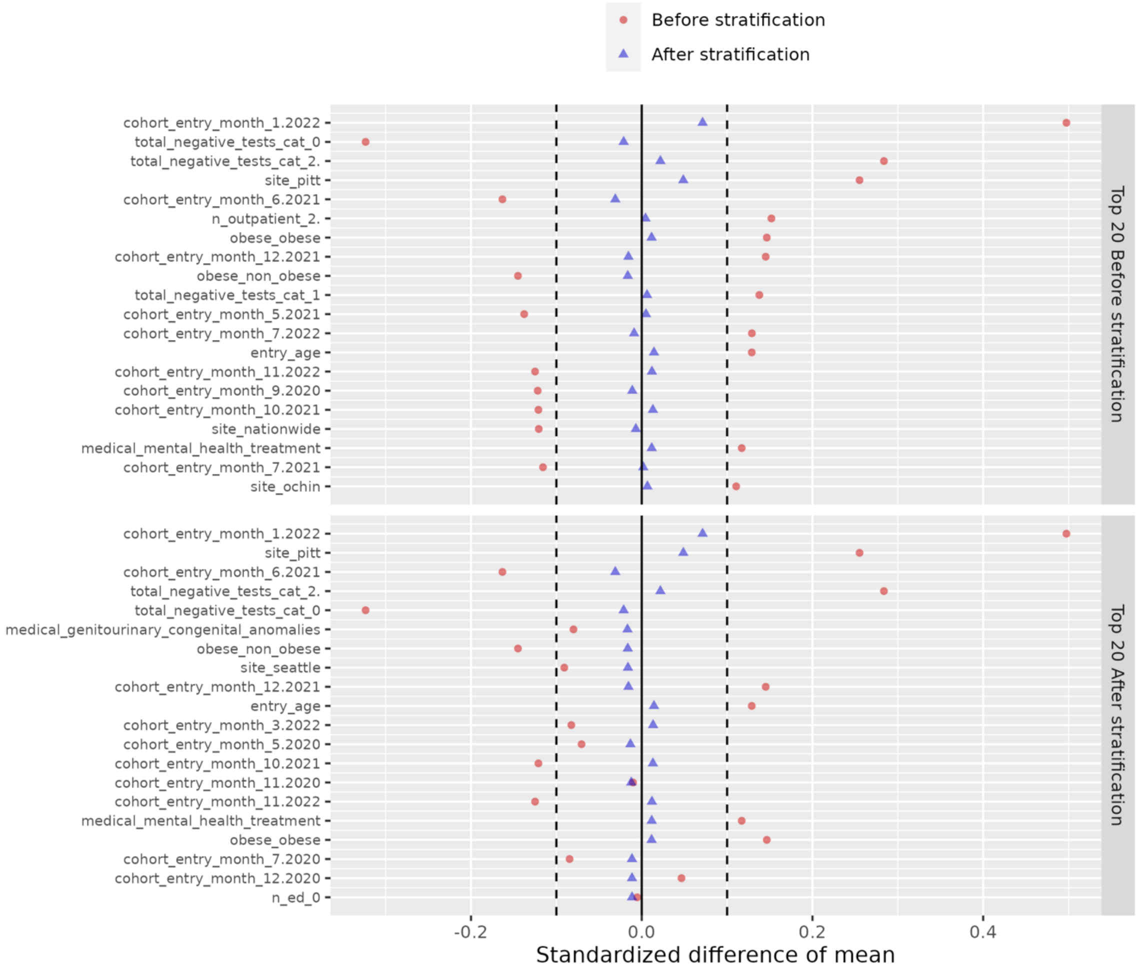

C. Hazard ratio of COVID-19 positive group compared to control group

Supplement Table 5: Estimated hazard ratio in kidney function outcomes between the COVID-19 positive cohort and the control cohort for female children and adolescents

Gender: Female	COVID-19 Groups Event (%)	Control Group Event (%)	HR (95% CI)
No CKD or AKI			
CKD 2+ (days 28-729)	1042 (0.44%)	2496 (0.37%)	1.19 [1.12, 1.26]
CKD 2+ (majority not returned to 90 and above) (days 28-729)	911 (0.38%)	2124 (0.32%)	1.21 [1.14, 1.28]
CKD 2+ (not returned to 90 and above) (days 28-729)	843 (0.35%)	1924 (0.29%)	1.21 [1.11, 1.33]
CKD 3+ (days 28-729)	75 (0.03%)	168 (0.03%)	1.41 [1.13, 1.75]
CKD 3+ (majority not returned to 90 and above) (days 28-729)	56 (0.02%)	119 (0.02%)	1.50 [1.13, 1.98]
CKD 3+ (not returned to 90 and above) (days 28-729)	66 (0.03%)	152 (0.02%)	1.33 [1.10, 1.61]
CKD 3+ (not returned to 60 and above) (days 28-729)	49 (0.02%)	105 (0.02%)	1.41 [1.06, 1.88]
CKD			
Composite outcome (days 28-179)	567 (13.99%)	1063 (11.86%)	1.14 [1.02, 1.26]
Composite outcome (days 180-729)	731 (18.04%)	1511 (16.86%)	1.14 [0.93, 1.40]
eGFR decline of 50% or more (days 28-179)	107 (2.64%)	222 (2.48%)	0.98 [0.71, 1.34]
eGFR decline of 50% or more (days 180-729)	138 (3.41%)	297 (3.31%)	1.22 [1.09, 1.37]
eGFR decline of 40% or more (days 28-179)	304 (7.5%)	591 (6.59%)	1.04 [0.84, 1.29]
eGFR decline of 40% or more (days 180-729)	404 (9.97%)	825 (9.2%)	1.15 [0.88, 1.49]
eGFR decline of 30% or more (days 28-179)	563 (13.89%)	1063 (11.86%)	1.13 [1.02, 1.25]
eGFR decline of 30% or more (days 180-729)	727 (17.94%)	1511 (16.86%)	1.13 [0.92, 1.39]
AKI			
Composite outcome (days 180-729)	173 (10.32%)	375 (5.79%)	1.27 [1.10, 1.47]
eGFR decline of 50% or more (days 180-729)	73 (4.35%)	158 (2.44%)	1.23 [0.98, 1.54]
eGFR decline of 40% or more (days 180-729)	131 (7.81%)	268 (4.14%)	1.34 [1.23, 1.46]
eGFR decline of 30% or more (days 180-729)	171 (10.2%)	375 (5.79%)	1.25 [1.08, 1.46]
CKD 2+ (days 28-729)	90 (5.37%)	217 (3.35%)	1.20 [0.96, 1.48]
CKD 2+ (majority not returned to 90 and above) (days 28-729)	86 (5.13%)	195 (3.01%)	1.26 [1.00, 1.59]
CKD 2+ (not returned to 90 and above) (days 28-729)	81 (4.83%)	177 (2.73%)	1.32 [1.05, 1.65]

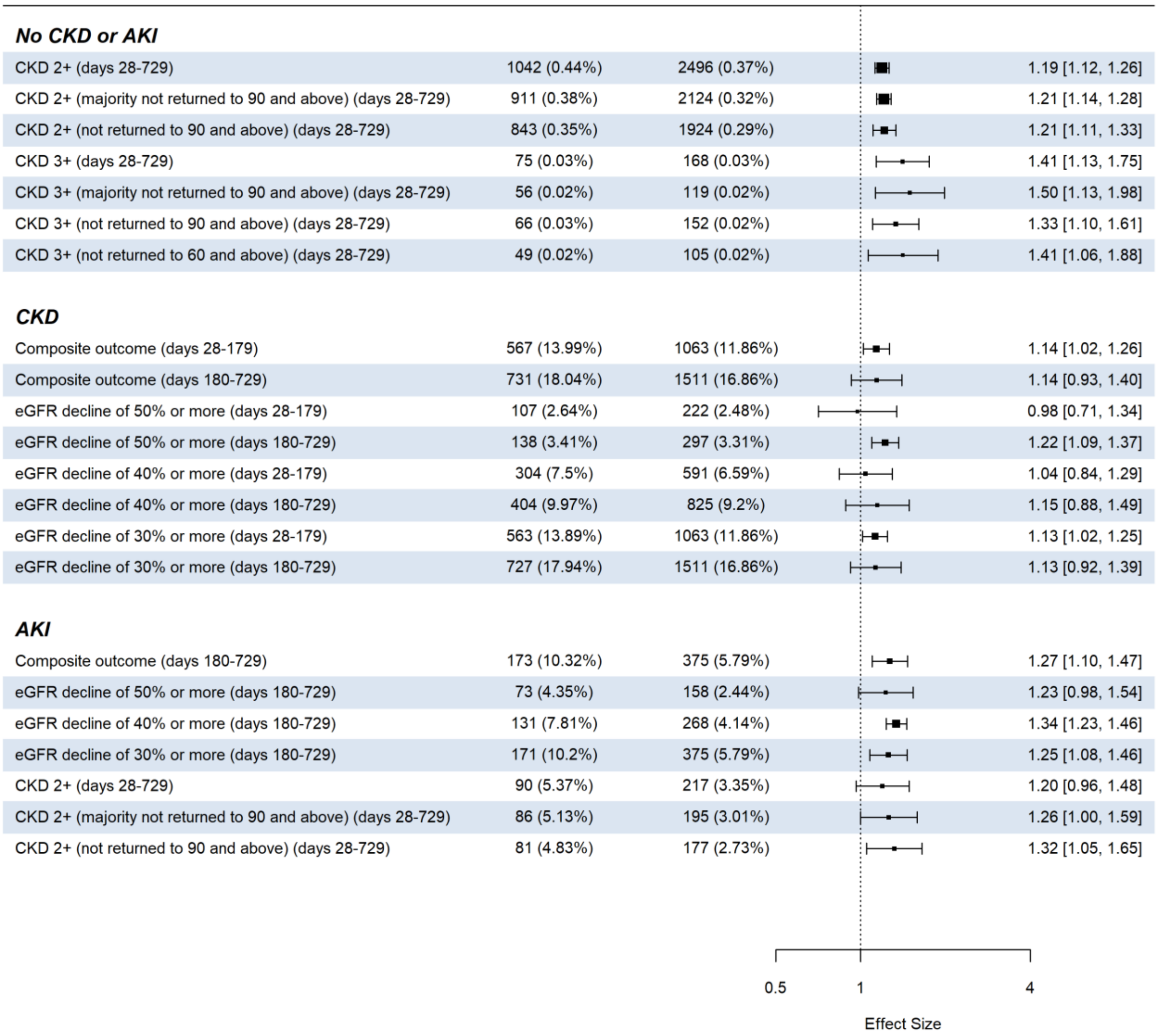

Supplement Table 6: Estimated hazard ratio in kidney function outcomes between the COVID-19 positive cohort and the control cohort for male children and adolescents

Gender: Male	COVID-19 Groups Event (%)	Control Group Event (%)	HR (95% CI)
No CKD or AKI			
CKD 2+ (days 28-729)	527 (0.22%)	1404 (0.2%)	1.14 [1.04, 1.26]
CKD 2+ (majority not returned to 90 and above) (days 28-729)	443 (0.19%)	1141 (0.16%)	1.15 [1.02, 1.29]
CKD 2+ (not returned to 90 and above) (days 28-729)	407 (0.17%)	985 (0.14%)	1.19 [1.06, 1.35]
CKD 3+ (days 28-729)	57 (0.02%)	141 (0.02%)	1.33 [0.84, 2.11]
CKD 3+ (majority not returned to 90 and above) (days 28-729)	42 (0.02%)	105 (0.01%)	1.30 [0.74, 2.28]
CKD 3+ (not returned to 90 and above) (days 28-729)	45 (0.02%)	119 (0.02%)	1.21 [0.68, 2.14]
CKD 3+ (not returned to 60 and above) (days 28-729)	39 (0.02%)	95 (0.01%)	1.30 [0.75, 2.26]
CKD			
Composite outcome (days 28-179)	338 (13.9%)	683 (11.32%)	1.21 [1.03, 1.42]
Composite outcome (days 180-729)	436 (17.94%)	1005 (16.65%)	1.16 [1.08, 1.24]
eGFR decline of 50% or more (days 28-179)	84 (3.46%)	183 (3.03%)	1.08 [0.86, 1.37]
eGFR decline of 50% or more (days 180-729)	108 (4.44%)	278 (4.61%)	1.03 [0.79, 1.33]
eGFR decline of 40% or more (days 28-179)	180 (7.4%)	366 (6.06%)	1.14 [1.01, 1.28]
eGFR decline of 40% or more (days 180-729)	211 (8.68%)	520 (8.61%)	1.09 [0.95, 1.26]
eGFR decline of 30% or more (days 28-179)	331 (13.62%)	683 (11.32%)	1.19 [1.01, 1.40]
eGFR decline of 30% or more (days 180-729)	432 (17.77%)	1005 (16.65%)	1.14 [1.07, 1.23]
AKI			
Composite outcome (days 90-179)	102 (4.66%)	260 (3.32%)	1.25 [1.04, 1.50]
Composite outcome (days 180-729)	197 (9.01%)	421 (5.38%)	1.40 [1.22, 1.60]
eGFR decline of 50% or more (days 90-179)	44 (2.01%)	100 (1.28%)	1.50 [0.84, 2.68]
eGFR decline of 50% or more (days 180-729)	89 (4.07%)	150 (1.92%)	1.65 [1.25, 2.17]
eGFR decline of 40% or more (days 90-179)	61 (2.79%)	157 (2.01%)	1.24 [0.82, 1.86]
eGFR decline of 40% or more (days 180-729)	125 (5.72%)	248 (3.17%)	1.47 [1.26, 1.70]
eGFR decline of 30% or more (days 90-179)	100 (4.57%)	260 (3.32%)	1.22 [1.02, 1.47]
eGFR decline of 30% or more (days 180-729)	194 (8.87%)	421 (5.38%)	1.37 [1.21, 1.54]
CKD 2+ (days 28-729)	104 (4.76%)	288 (3.68%)	1.16 [0.99, 1.36]

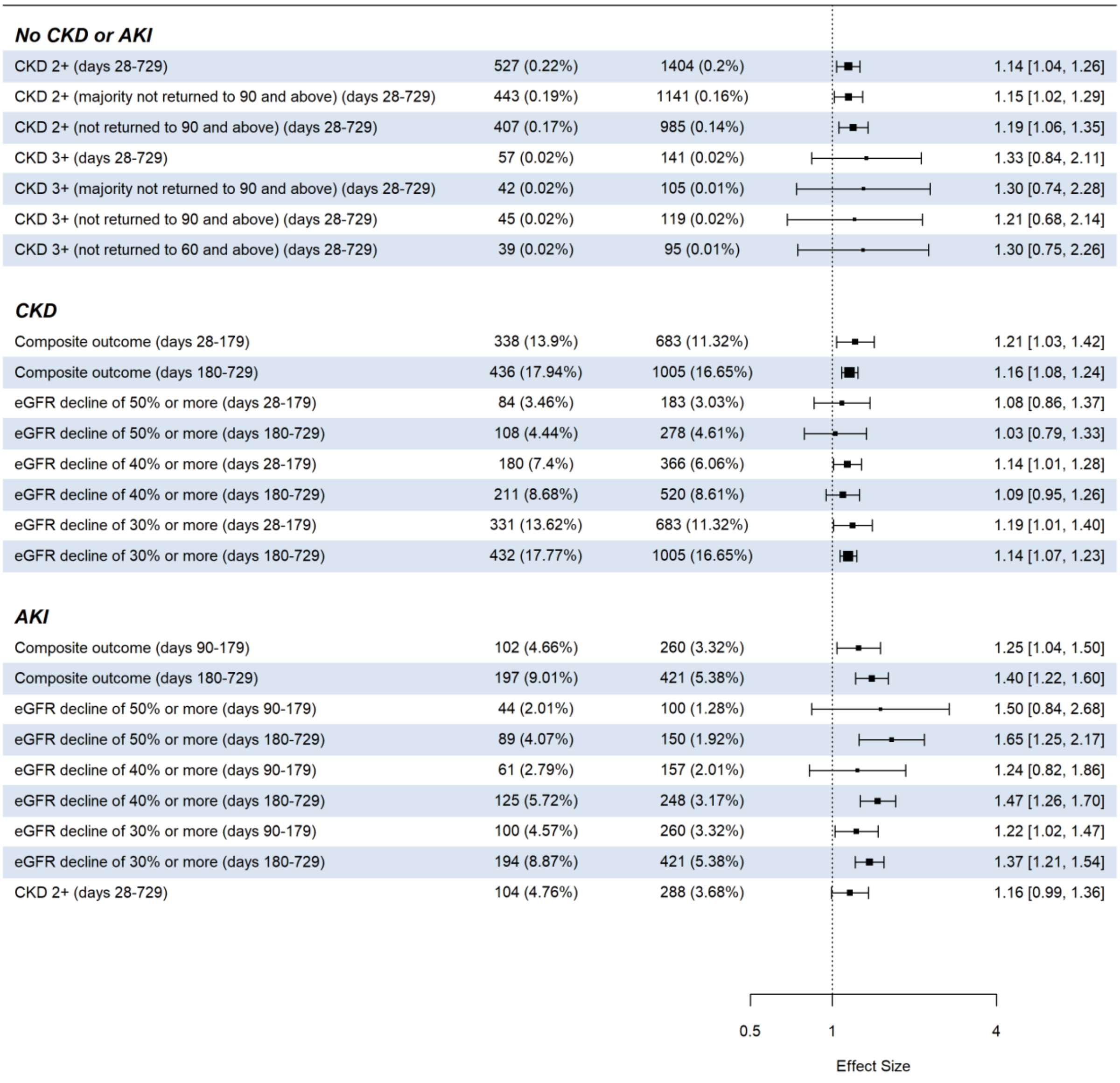

eAppendix 6. Sensitivity Analysis for Race and Ethnicity

We conducted sensitivity analyses on both cohorts stratified by ethnicity (i.e., Asian/PI, Black American and African American, Hispanic, and White) to investigate the potential effect of race on the risk of kidney function outcomes. We performed the same PS stratification procedure and used Cox proportional hazard model to estimate the hazard ratio.

A. Empirical equipoise assessment

Supplement Figure 37: Preference score distributions of COVID-19 positive and negative groups for children and adolescents with AKI in Asian American and Pacific Islanders group. A greater convergence of these distributions indicates a higher similarity in the predicted likelihood of being infected between the COVID-19 positive (red) and negative (blue) participants.

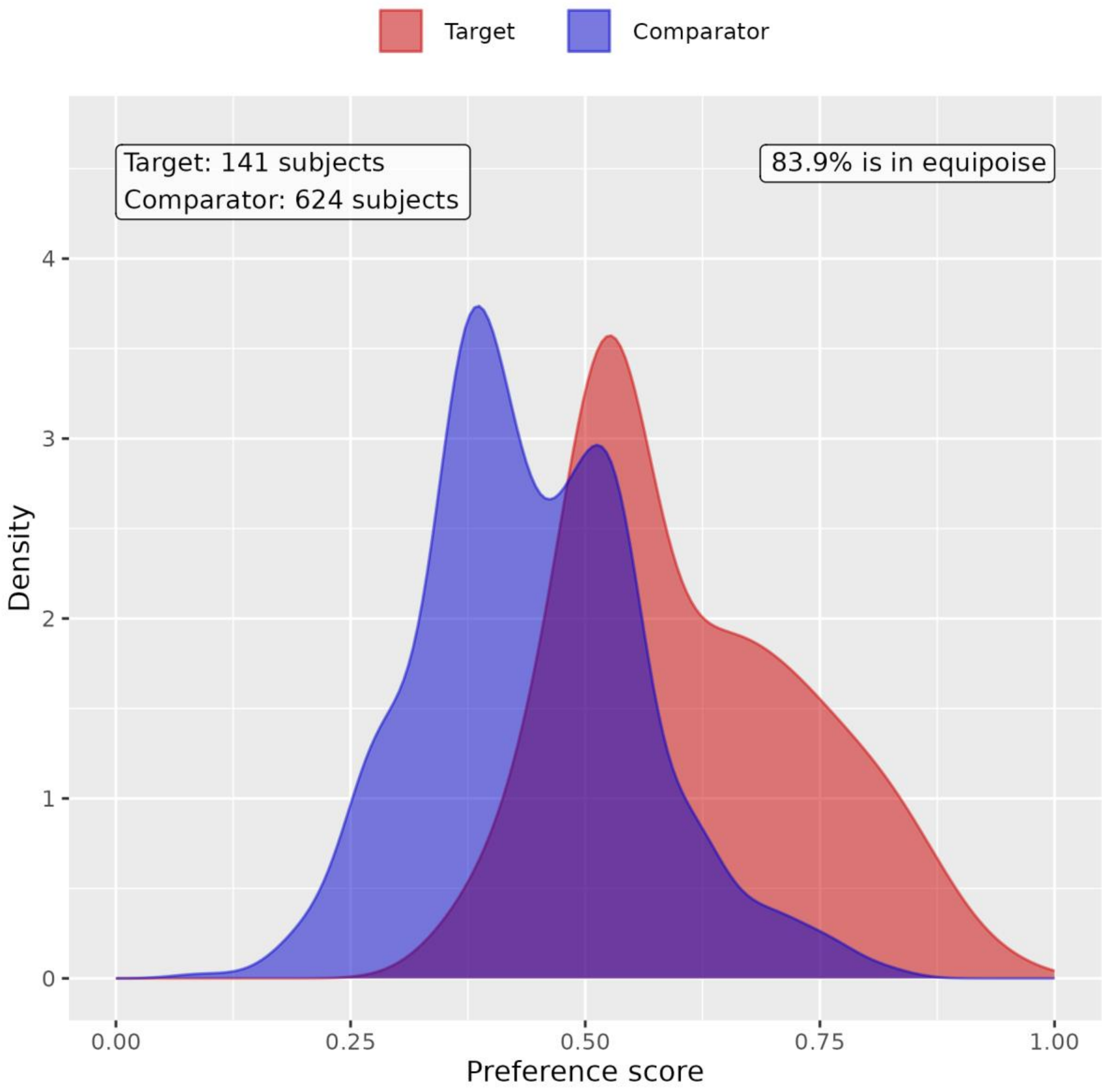

Supplement Figure 38: Preference score distributions of COVID-19 positive and negative groups for children and adolescents with AKI in Black American and African American group. A greater convergence of these distributions indicates a higher similarity in the predicted likelihood of being infected between the COVID-19 positive (red) and negative (blue) participants.

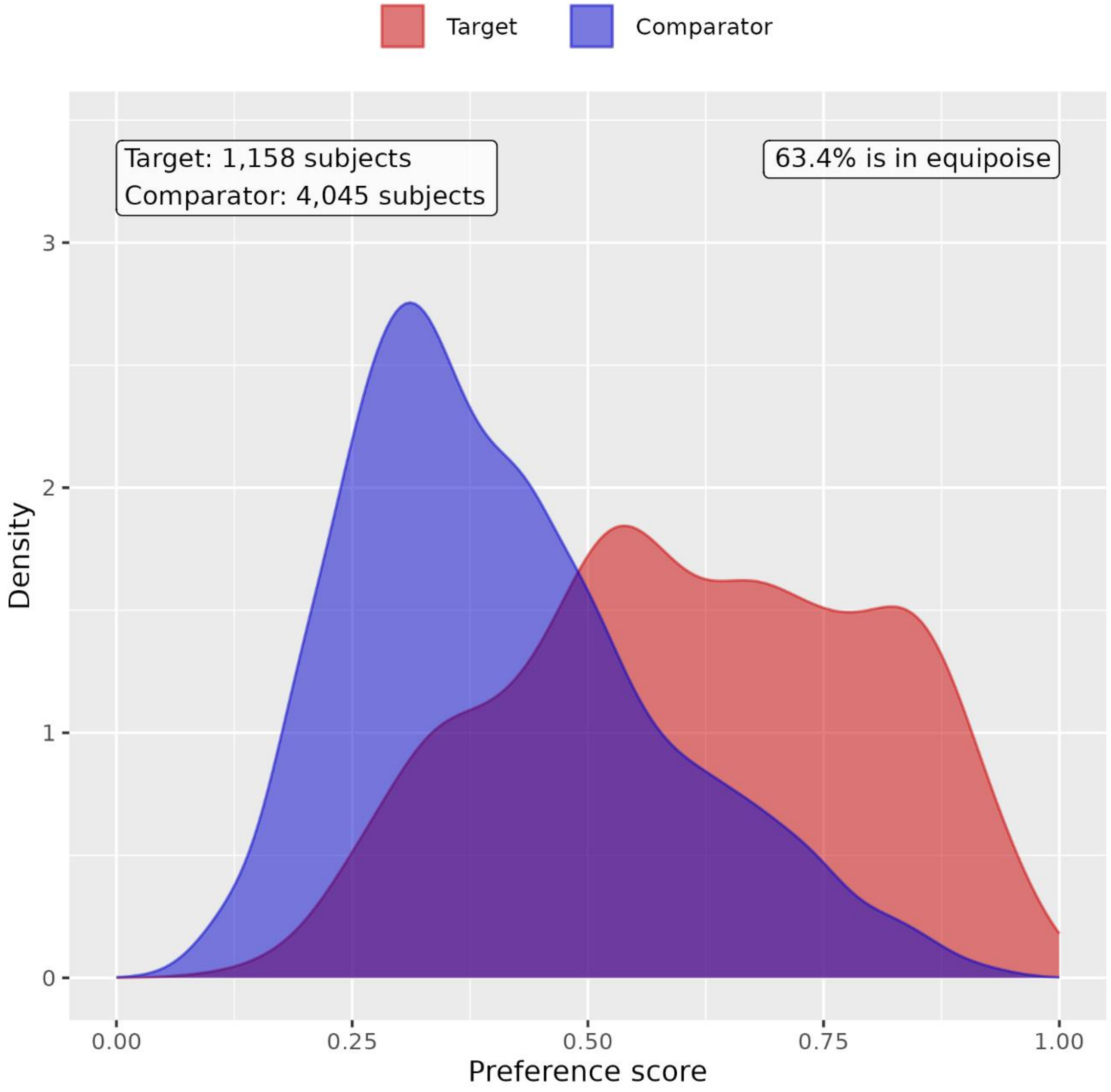
Target
Comparator
Target: 1,158 subjects
Comparator: 4,045 subjects
63.4% is in equipoise
Density
3
2
1
0
0.00
0.25
0.50
0.75
1.00
Preference score

Supplement Figure 39: Preference score distributions of COVID-19 positive and negative groups for children and adolescents with AKI in Hispanic group. A greater convergence of these distributions indicates a higher similarity in the predicted likelihood of being infected between the COVID-19 positive (red) and negative (blue) participants.

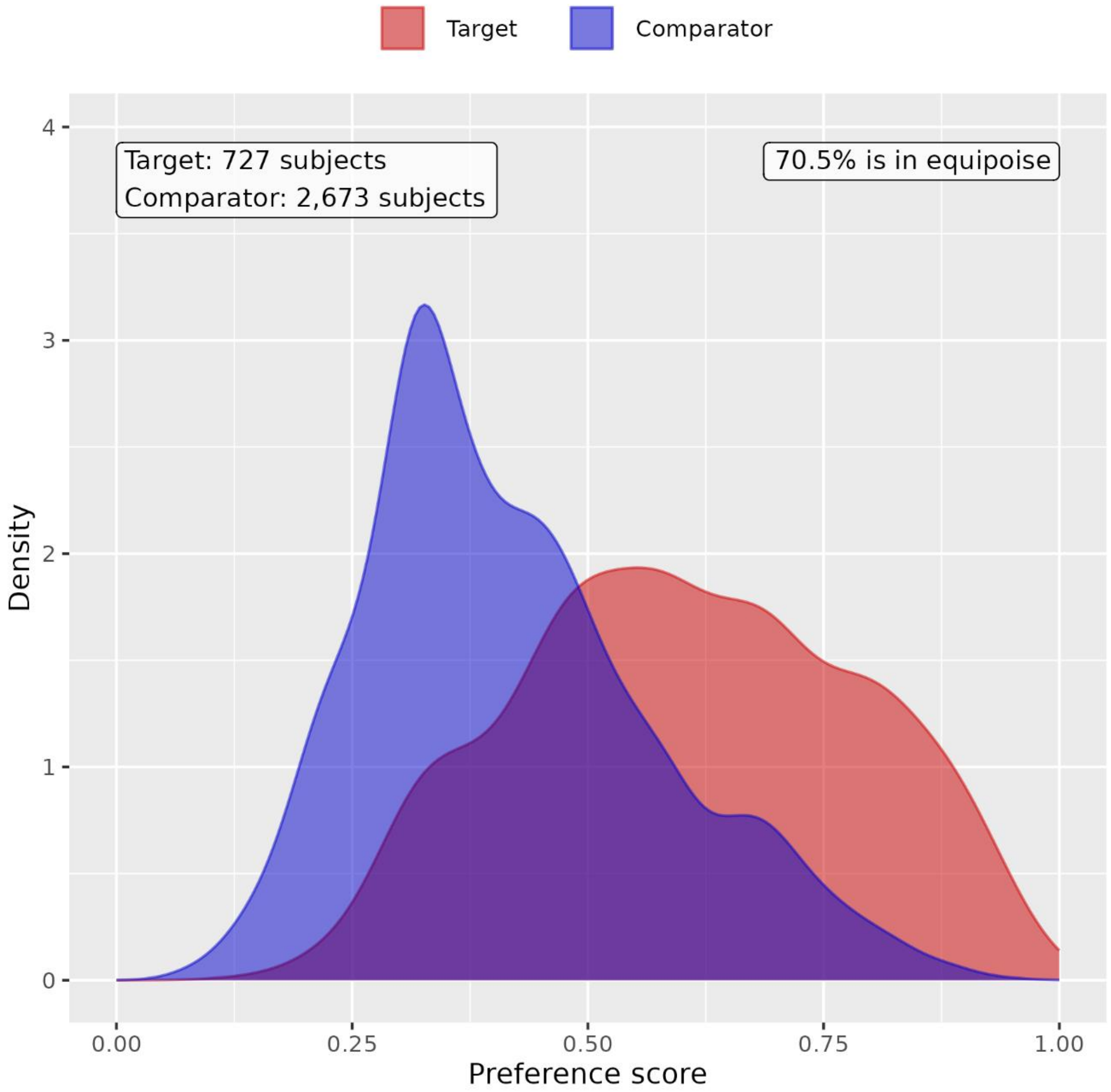

Supplement Figure 40: Preference score distributions of COVID-19 positive and negative groups for children and adolescents with AKI in White group. A greater convergence of these distributions indicates a higher similarity in the predicted likelihood of being infected between the COVID-19 positive (red) and negative (blue) participants.

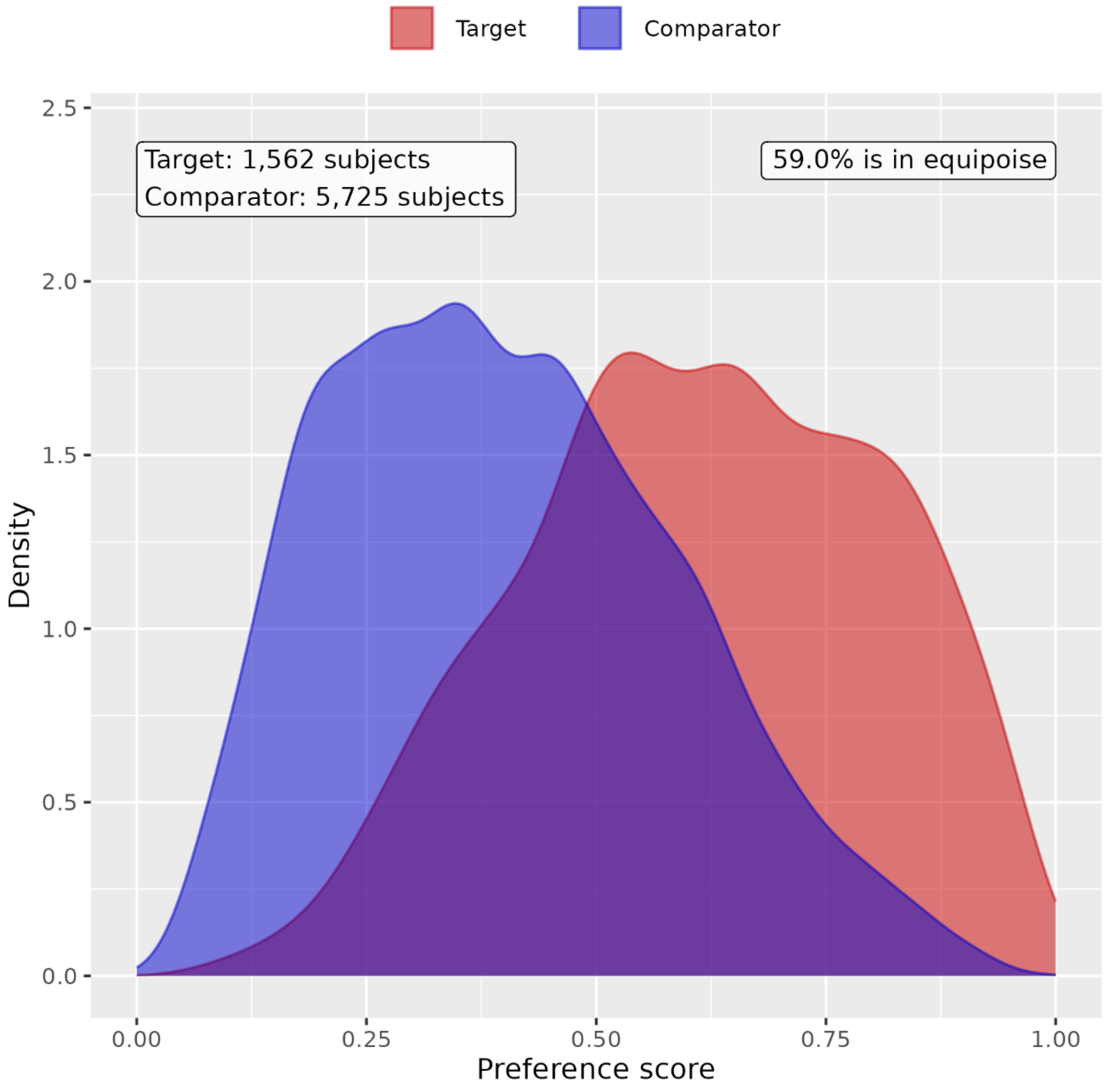

Supplement Figure 41: Preference score distributions of COVID-19 positive and negative groups for children and adolescents with CKD in Asian American and Pacific Islanders group. A greater convergence of these distributions indicates a higher similarity in the predicted likelihood of being infected between the COVID-19 positive (red) and negative (blue) participants.

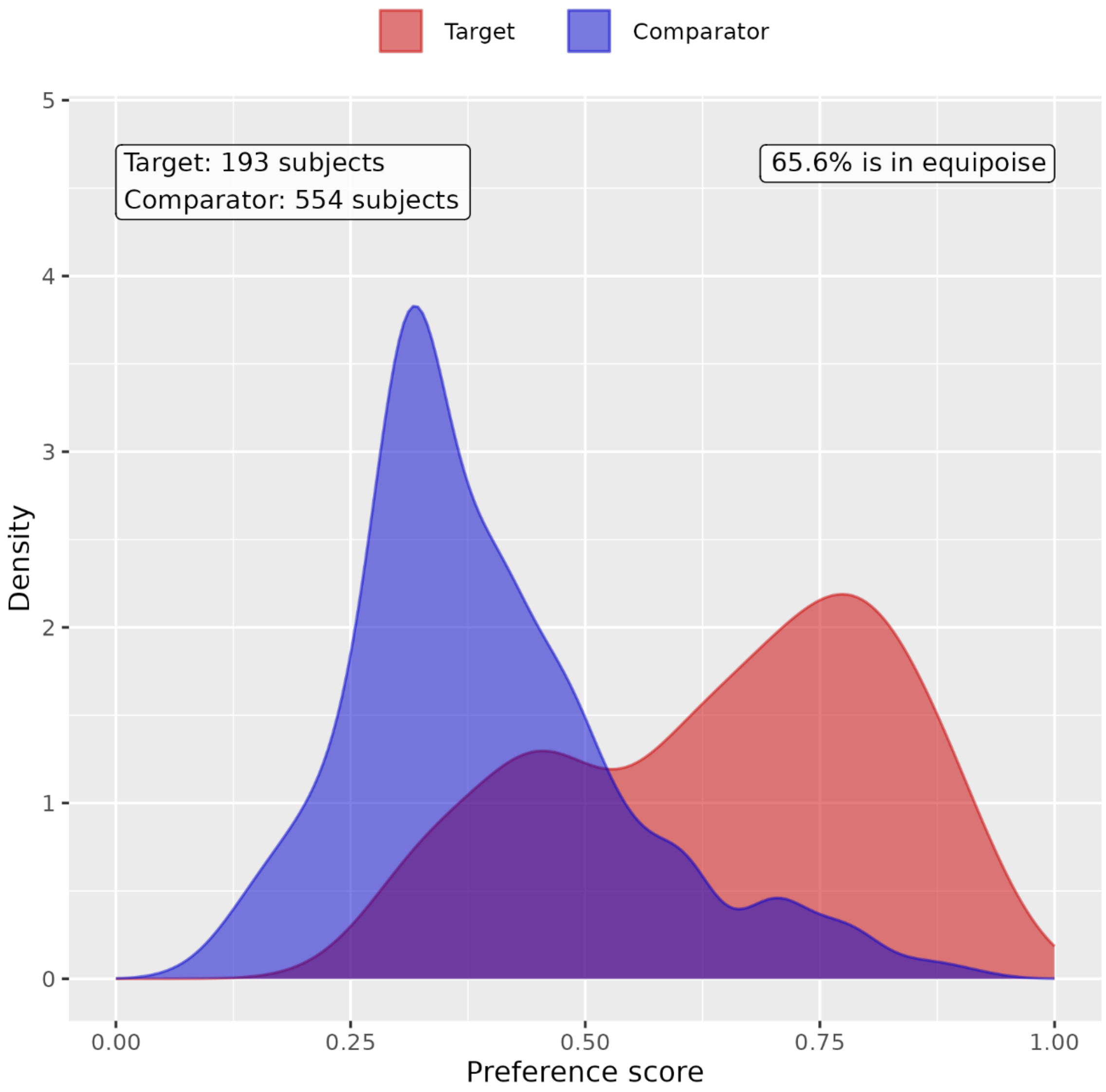

Supplement Figure 42: Preference score distributions of COVID-19 positive and negative groups for children and adolescents with CKD in Black American and African American group. A greater convergence of these distributions indicates a higher similarity in the predicted likelihood of being infected between the COVID-19 positive (red) and negative (blue) participants.

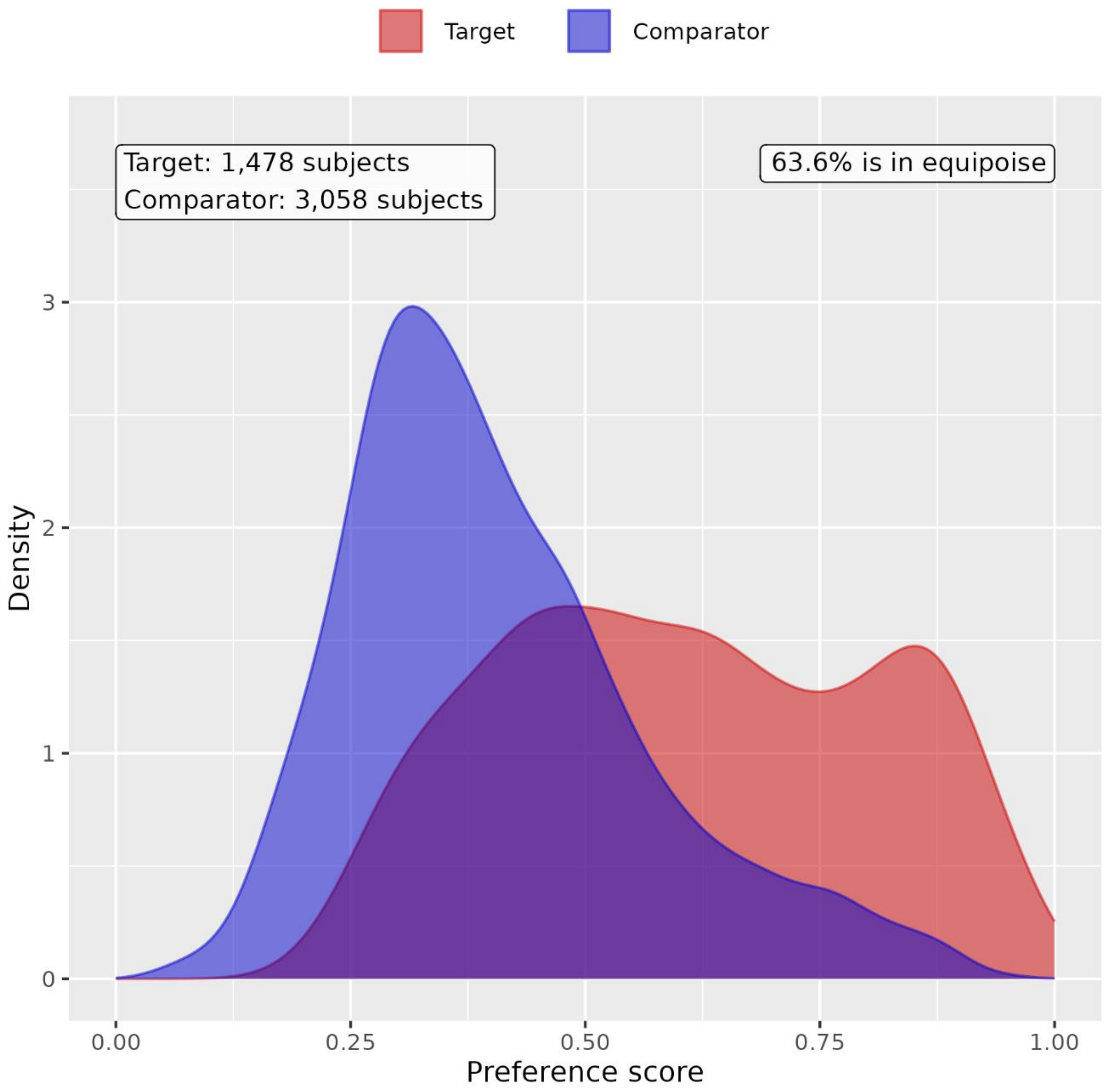

Supplement Figure 43: Preference score distributions of COVID-19 positive and negative groups for children and adolescents with CKD in Hispanic group. A greater convergence of these distributions indicates a higher similarity in the predicted likelihood of being infected between the COVID-19 positive (red) and negative (blue) participants.

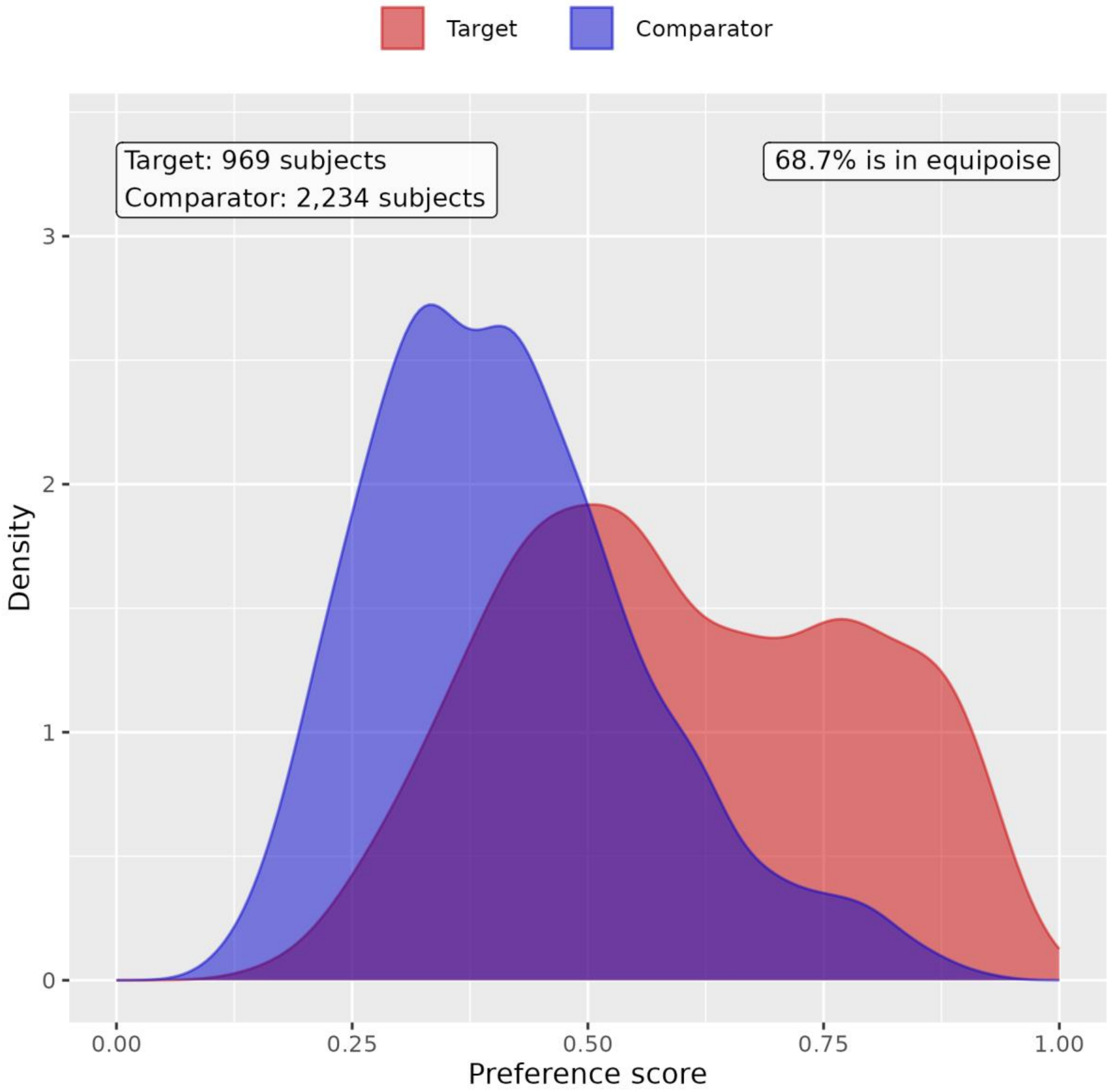

Supplement Figure 44: Preference score distributions of COVID-19 positive and negative groups for children and adolescents with CKD in White group. A greater convergence of these distributions indicates a higher similarity in the predicted likelihood of being infected between the COVID-19 positive (red) and negative (blue) participants.

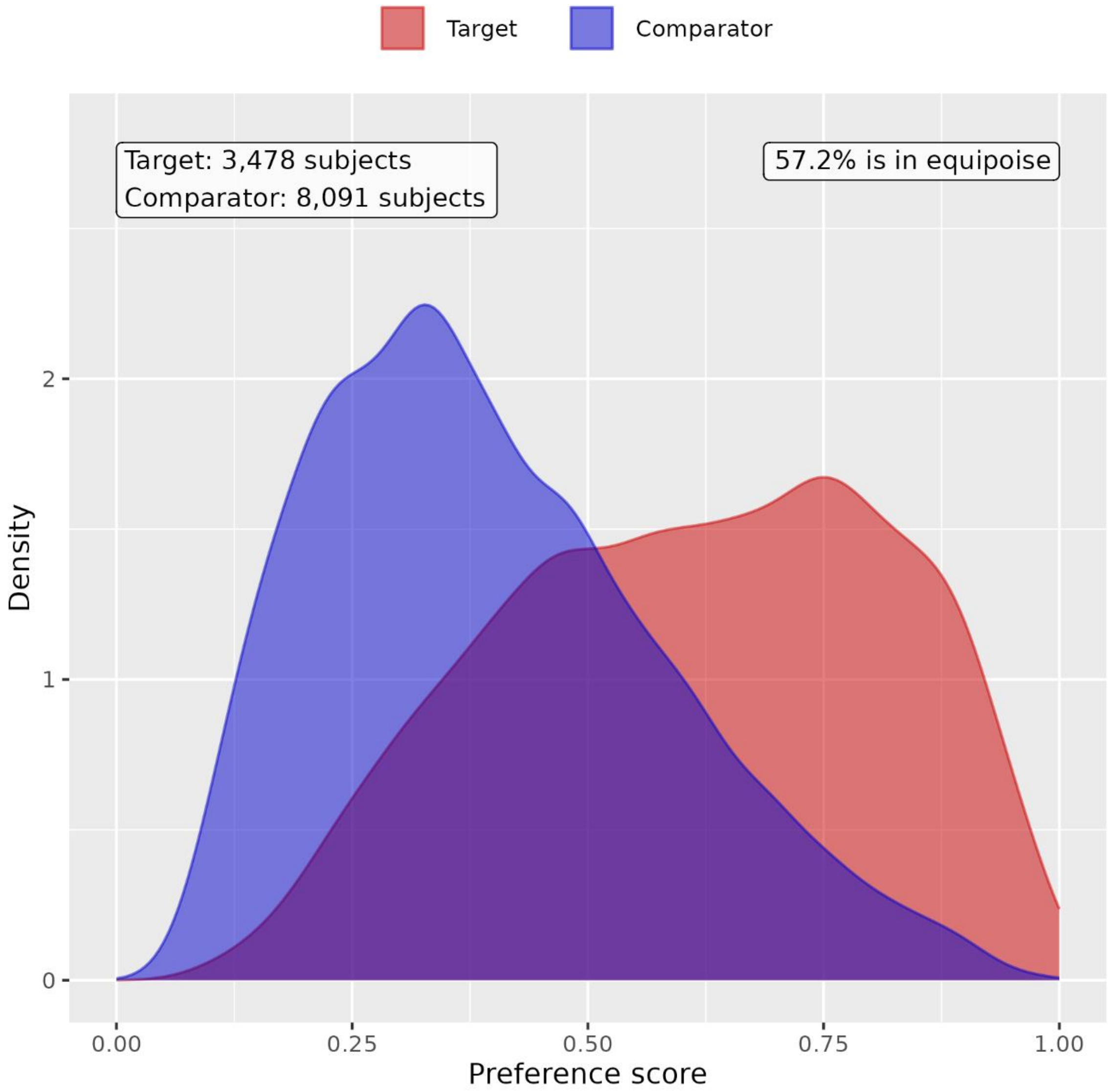

Supplement Figure 45: Preference score distributions of COVID-19 positive and negative groups for children and adolescents with no AKI or CKD in Asian American and Pacific Islanders group. A greater convergence of these distributions indicates a higher similarity in the predicted likelihood of being infected between the COVID-19 positive (red) and negative (blue) participants.

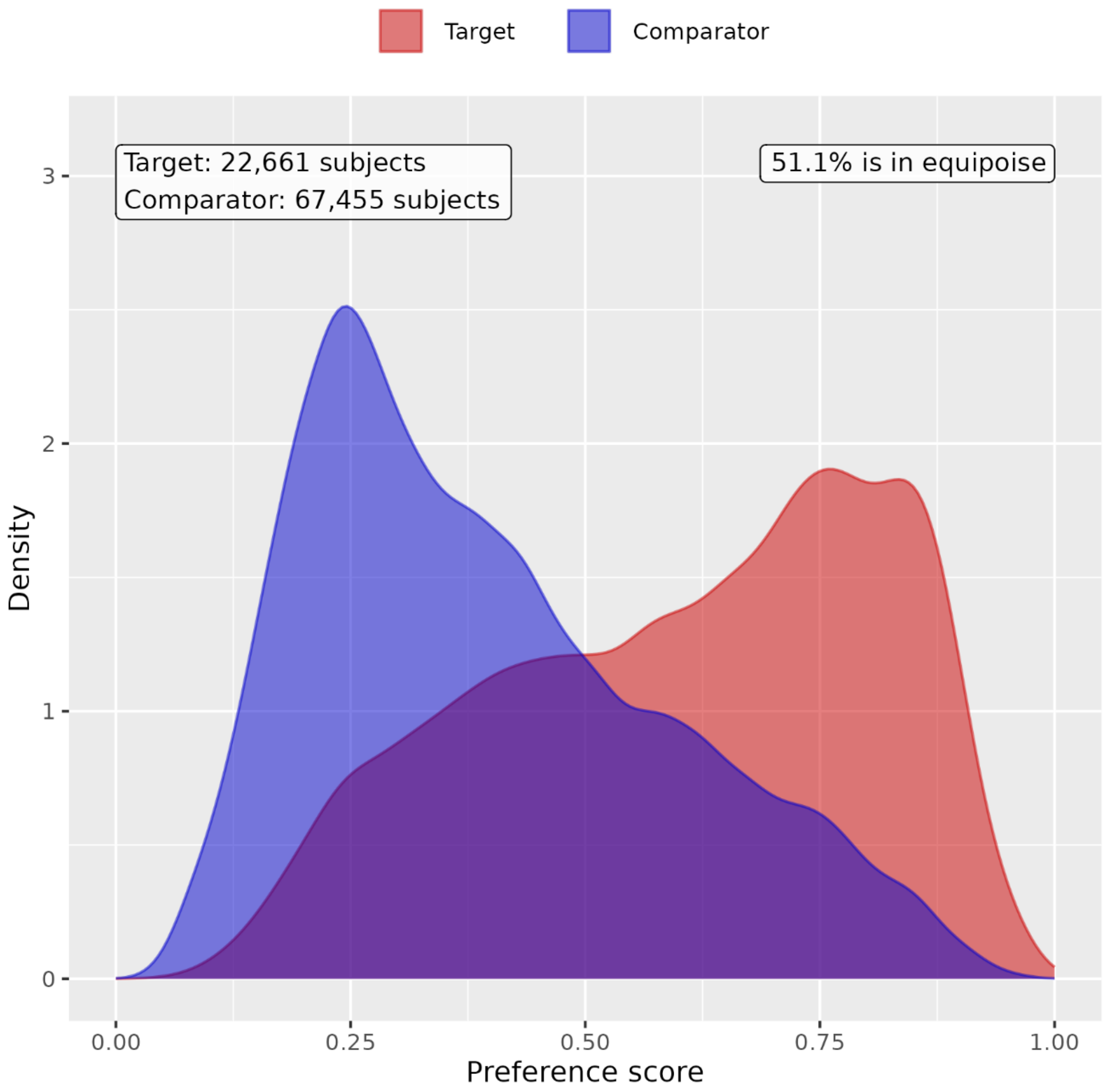

Supplement Figure 46: Preference score distributions of COVID-19 positive and negative groups for children and adolescents with no AKI or CKD in Black American and African American group. A greater convergence of these distributions indicates a higher similarity in the predicted likelihood of being infected between the COVID-19 positive (red) and negative (blue) participants.

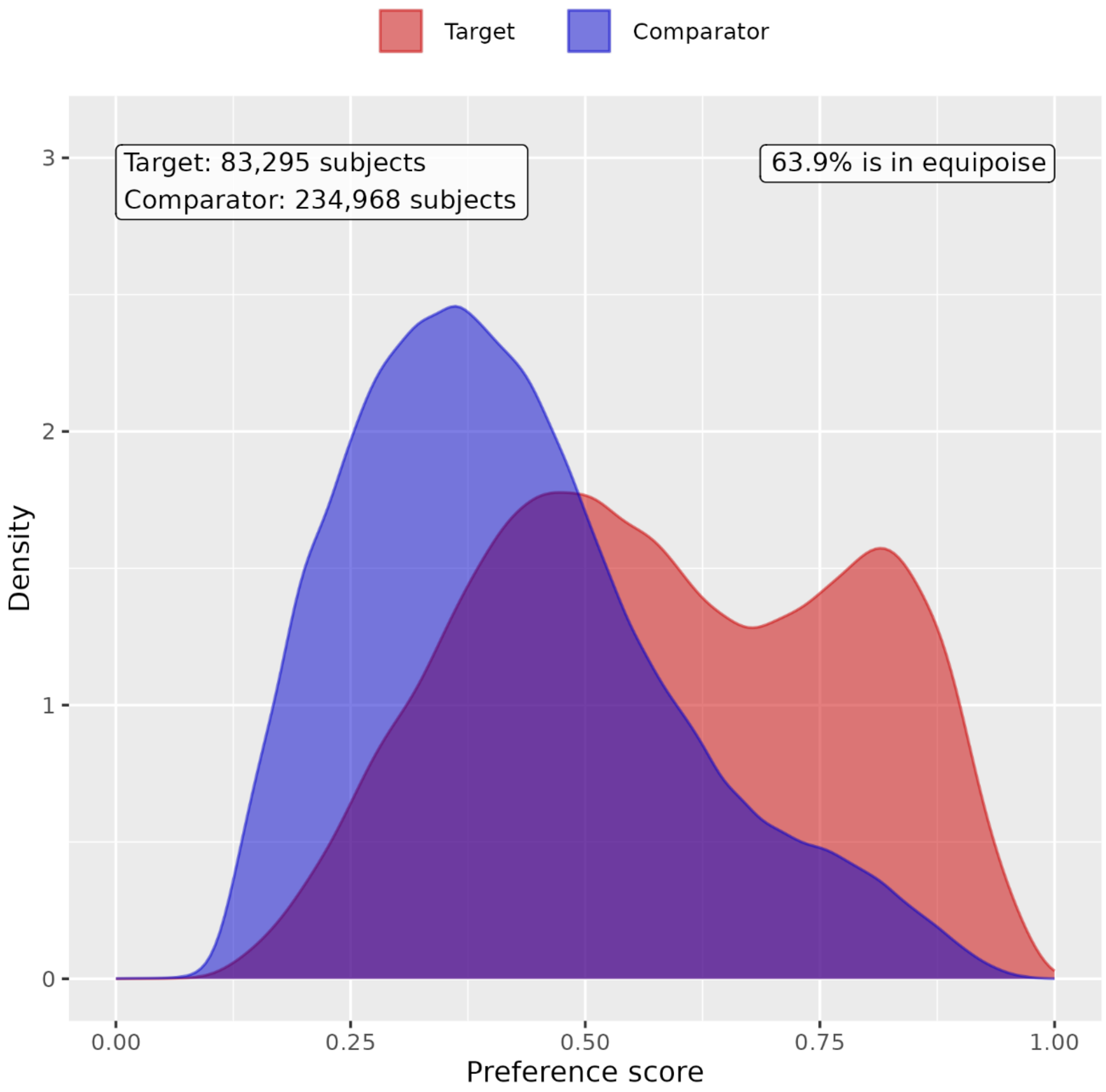

Supplement Figure 47: Preference score distributions of COVID-19 positive and negative groups for children and adolescents with no AKI or CKD in Hispanic group. A greater convergence of these distributions indicates a higher similarity in the predicted likelihood of being infected between the COVID-19 positive (red) and negative (blue) participants.

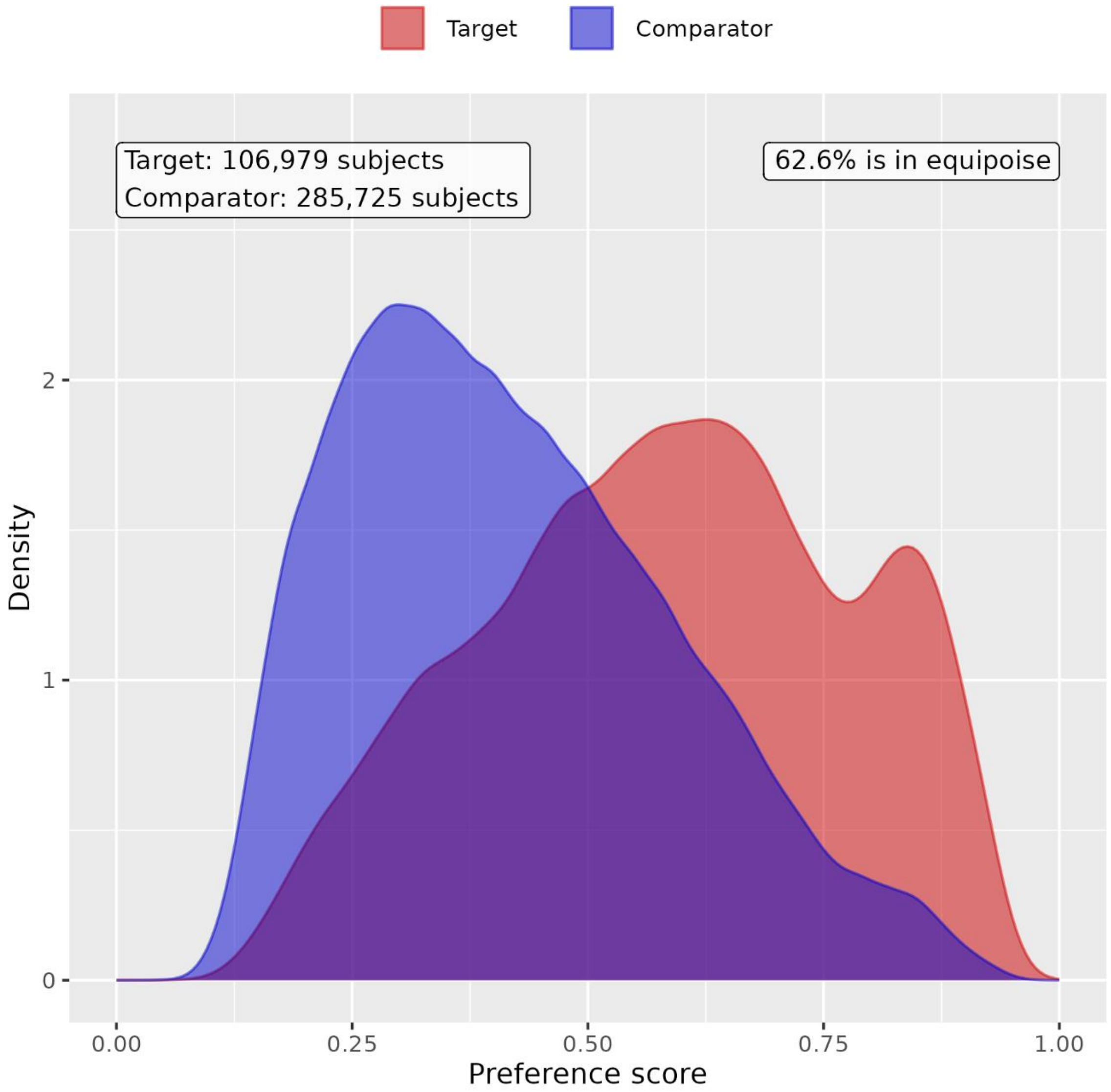

Supplement Figure 48: Preference score distributions of COVID-19 positive and negative groups for children and adolescents with no AKI or CKD in White group. A greater convergence of these distributions indicates a higher similarity in the predicted likelihood of being infected between the COVID-19 positive (red) and negative (blue) participants.

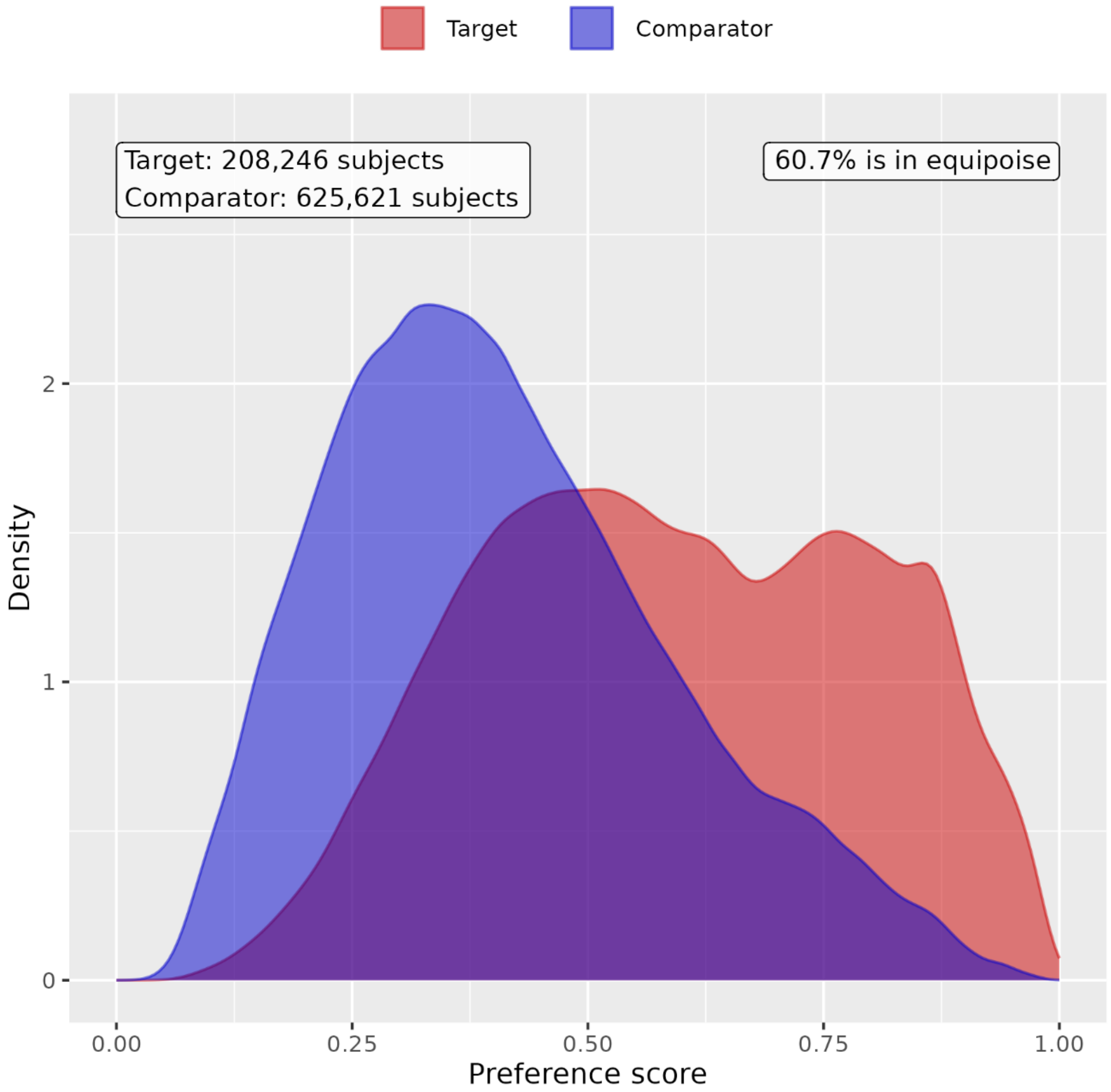

B. Patient characteristic balance

Supplement Figure 49: Patient characteristic balance before and after large-scale PS stratification with 6 strata for children and adolescents with AKI in Asian American and Pacific Islanders group. The upper panel displays the top 20 covariates with the largest standardized difference of means before stratification, while the lower panel displays the top 20 covariates with the largest standardized difference of means after stratification.

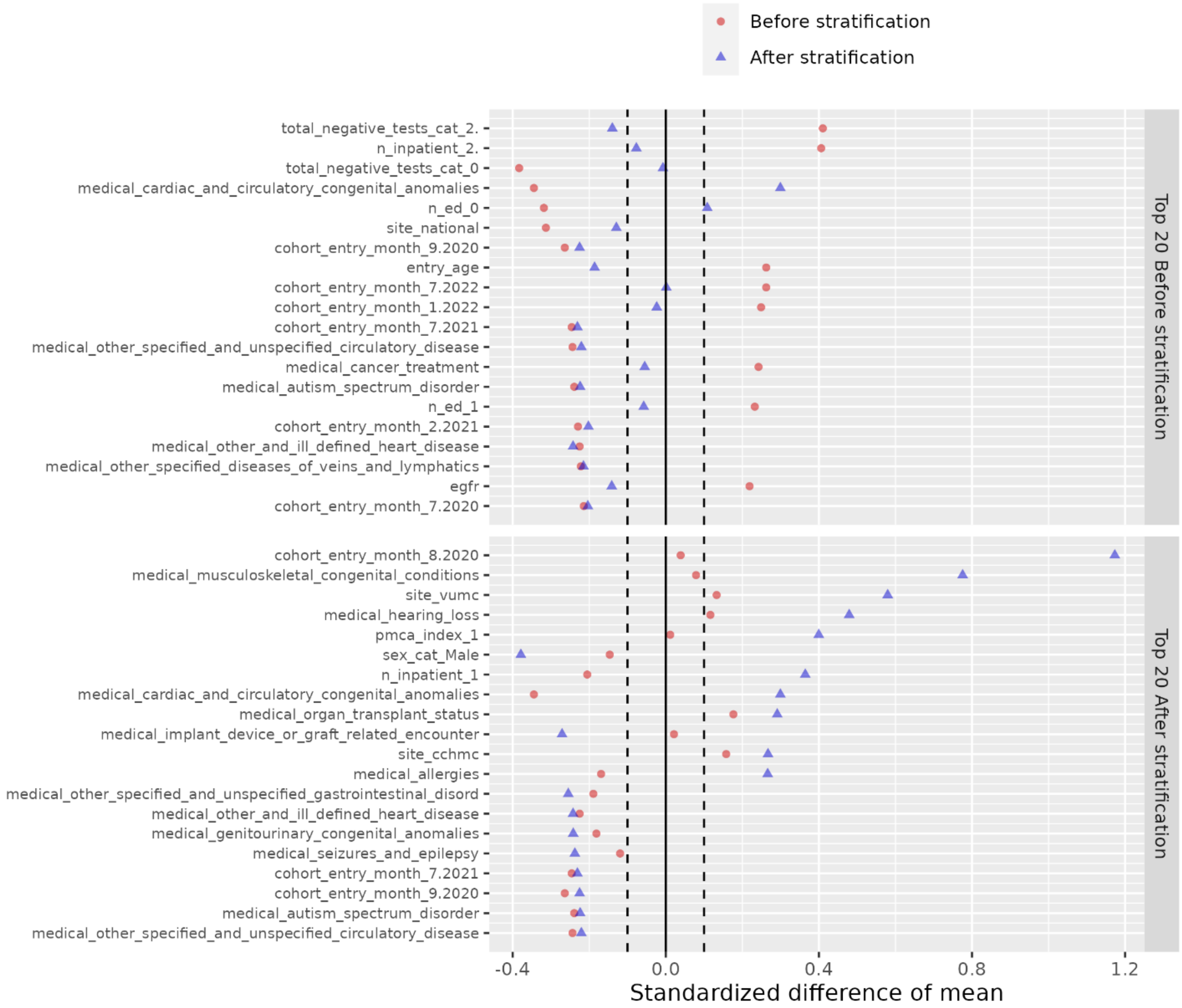

Supplement Figure 50: Patient characteristic balance before and after large-scale PS stratification with 6 strata for children and adolescents with AKI in Black American and African American group. The upper panel displays the top 20 covariates with the largest standardized difference of means before stratification, while the lower panel displays the top 20 covariates with the largest standardized difference of means after stratification.

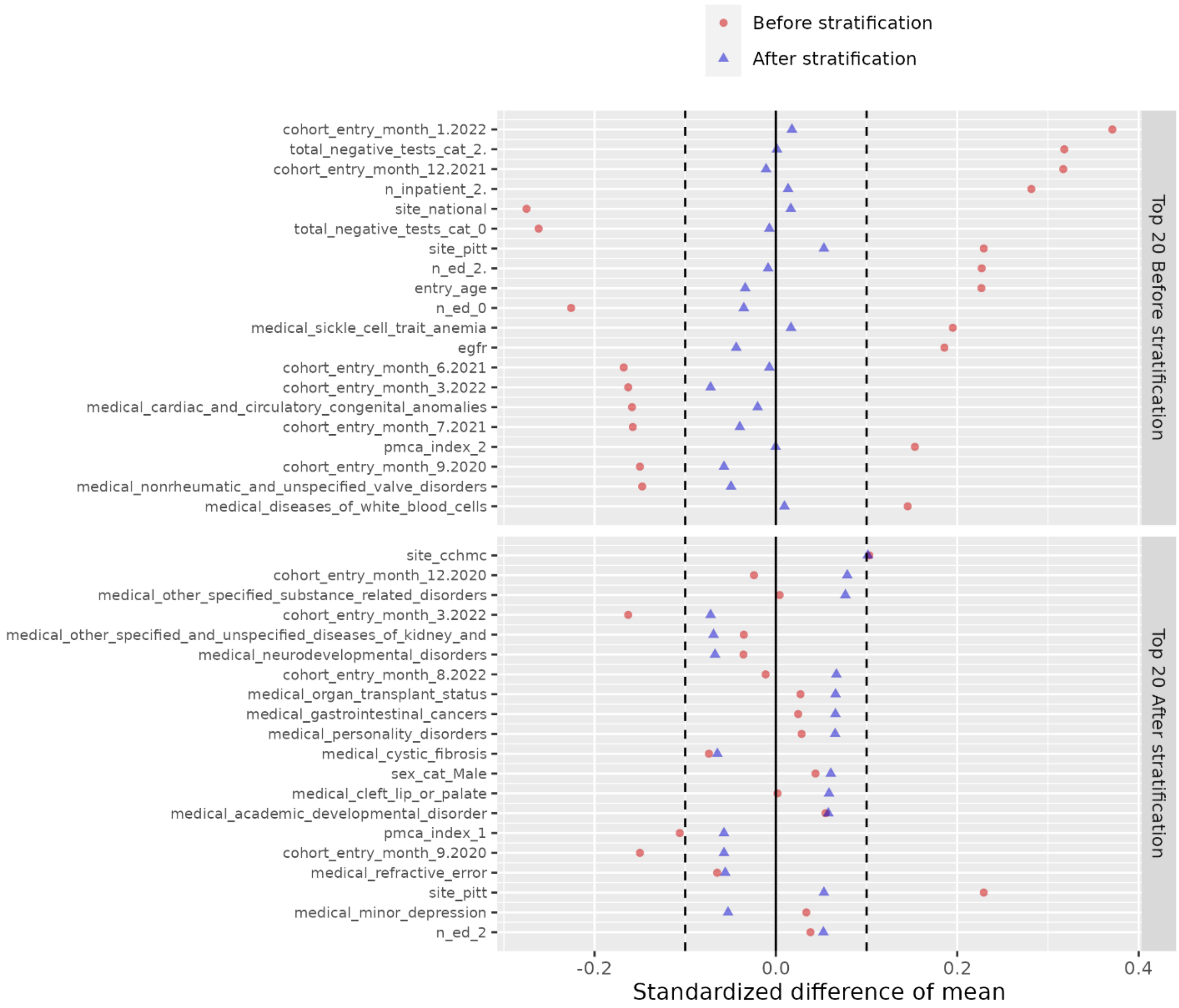

Supplement Figure 51: Patient characteristic balance before and after large-scale PS stratification with 6 strata for children and adolescents with AKI in Hispanic group. The upper panel displays the top 20 covariates with the largest standardized difference of means before stratification, while the lower panel displays the top 20 covariates with the largest standardized difference of means after stratification.

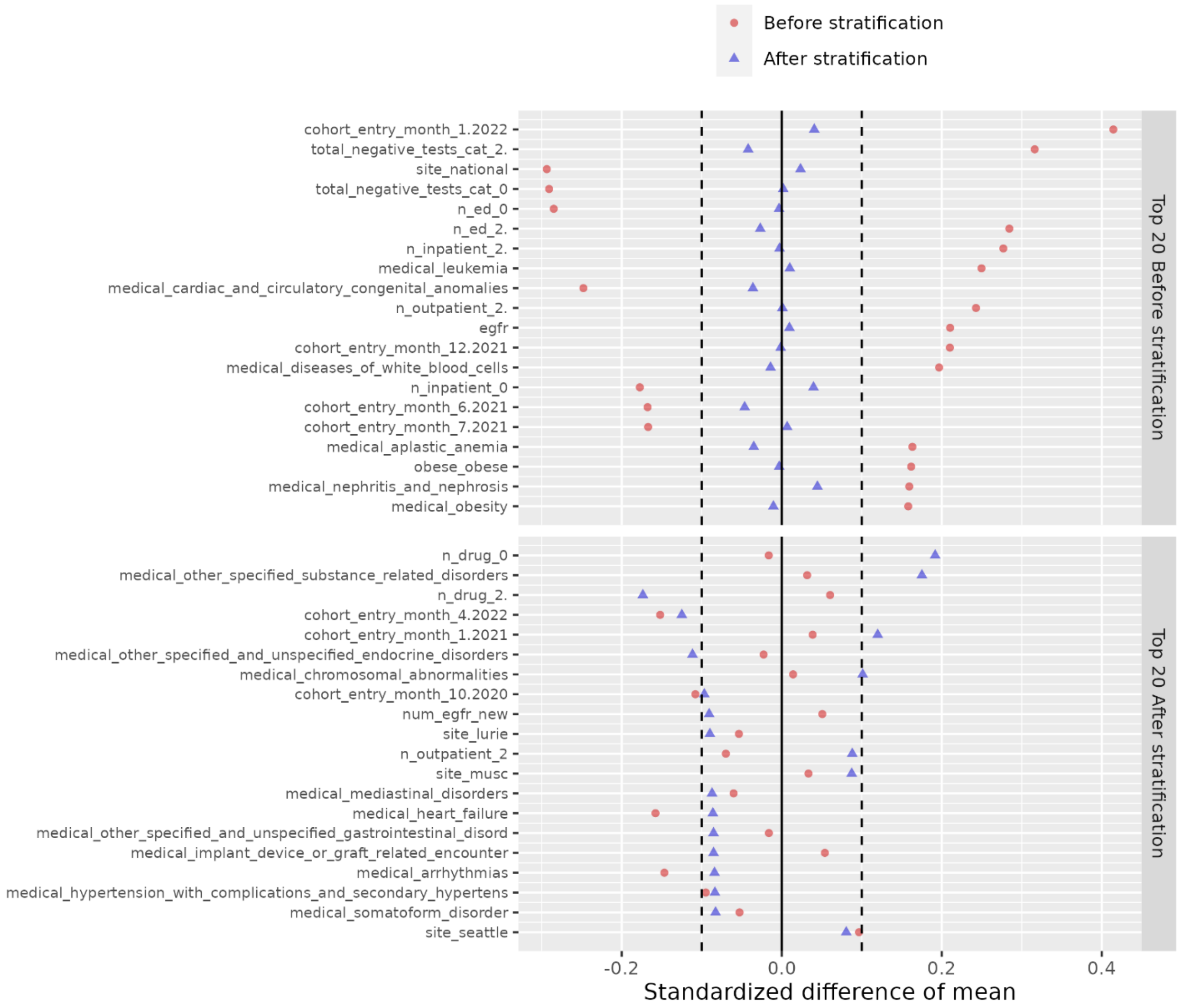

Supplement Figure 52: Patient characteristic balance before and after large-scale PS stratification with 6 strata for children and adolescents with AKI in White group. The upper panel displays the top 20 covariates with the largest standardized difference of means before stratification, while the lower panel displays the top 20 covariates with the largest standardized difference of means after stratification.

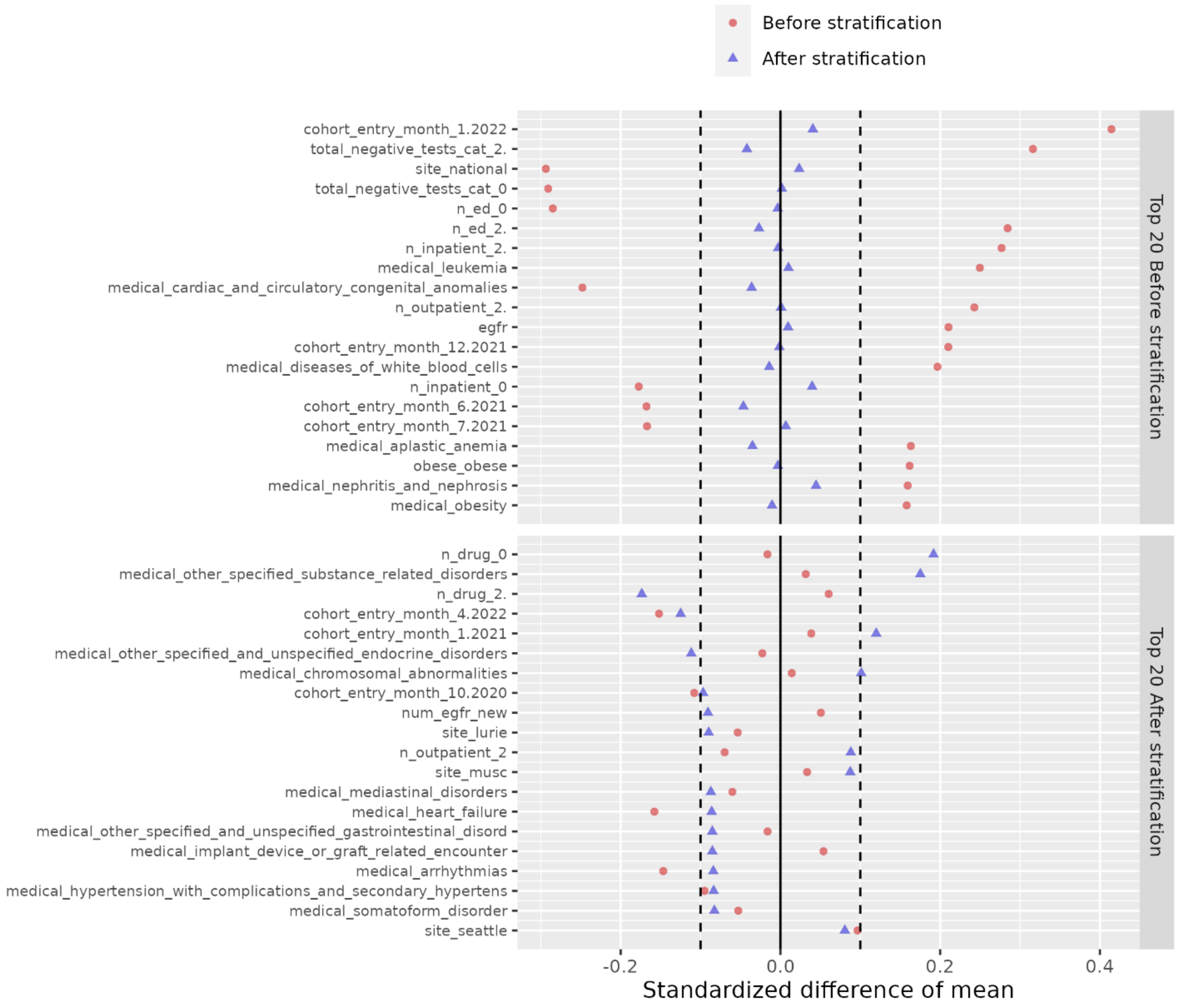

Supplement Figure 53: Patient characteristic balance before and after large-scale PS stratification with 6 strata for children and adolescents with CKD in Asian American and Pacific Islanders group. The upper panel displays the top 20 covariates with the largest standardized difference of means before stratification, while the lower panel displays the top 20 covariates with the largest standardized difference of means after stratification.

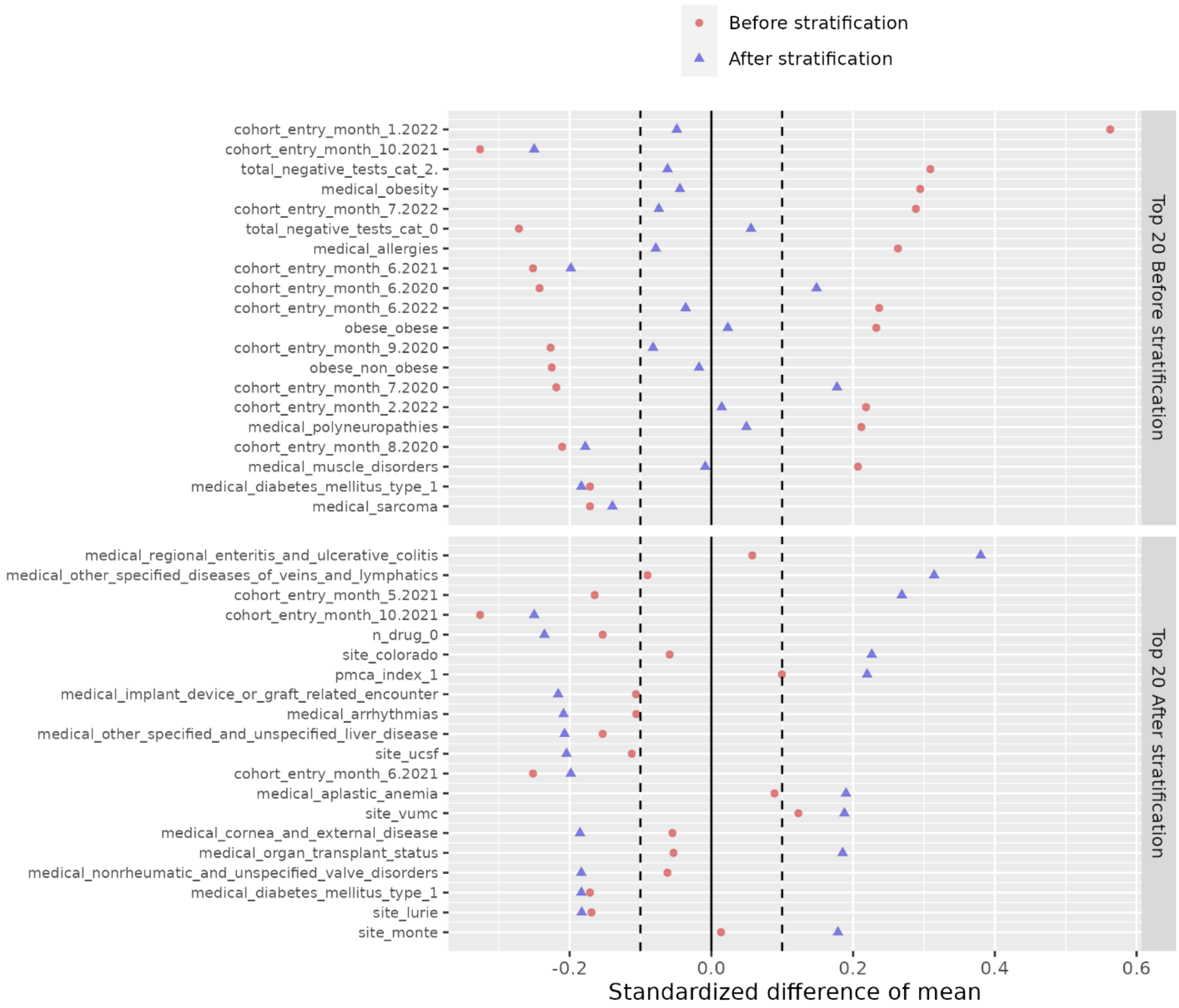

Supplement Figure 54: Patient characteristic balance before and after large-scale PS stratification with 6 strata for children and adolescents with CKD in Black American and African American group. The upper panel displays the top 20 covariates with the largest standardized difference of means before stratification, while the lower panel displays the top 20 covariates with the largest standardized difference of means after stratification.

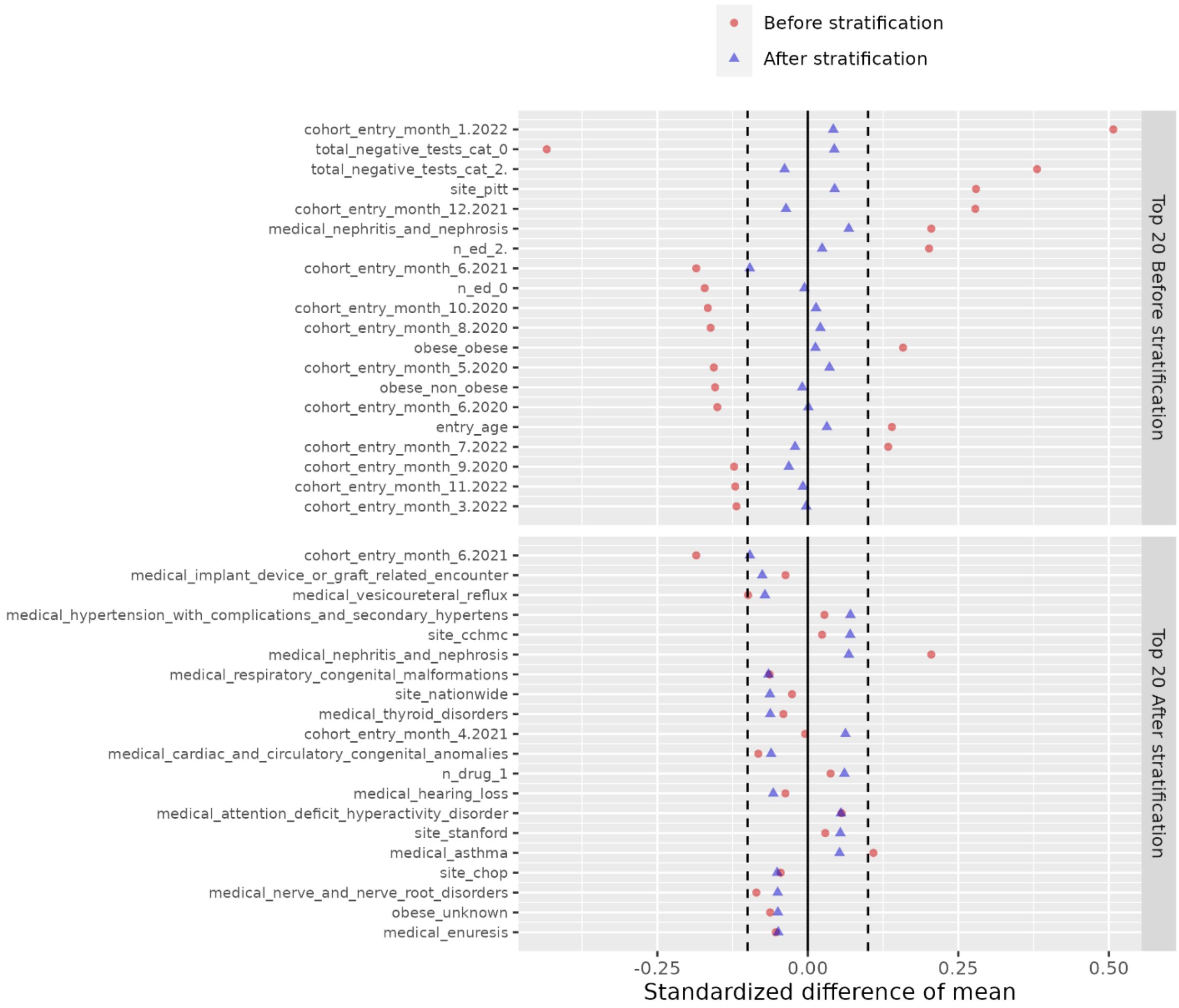

Supplement Figure 55: Patient characteristic balance before and after large-scale PS stratification with 6 strata for children and adolescents with CKD in Hispanic group. The upper panel displays the top 20 covariates with the largest standardized difference of means before stratification, while the lower panel displays the top 20 covariates with the largest standardized difference of means after stratification.

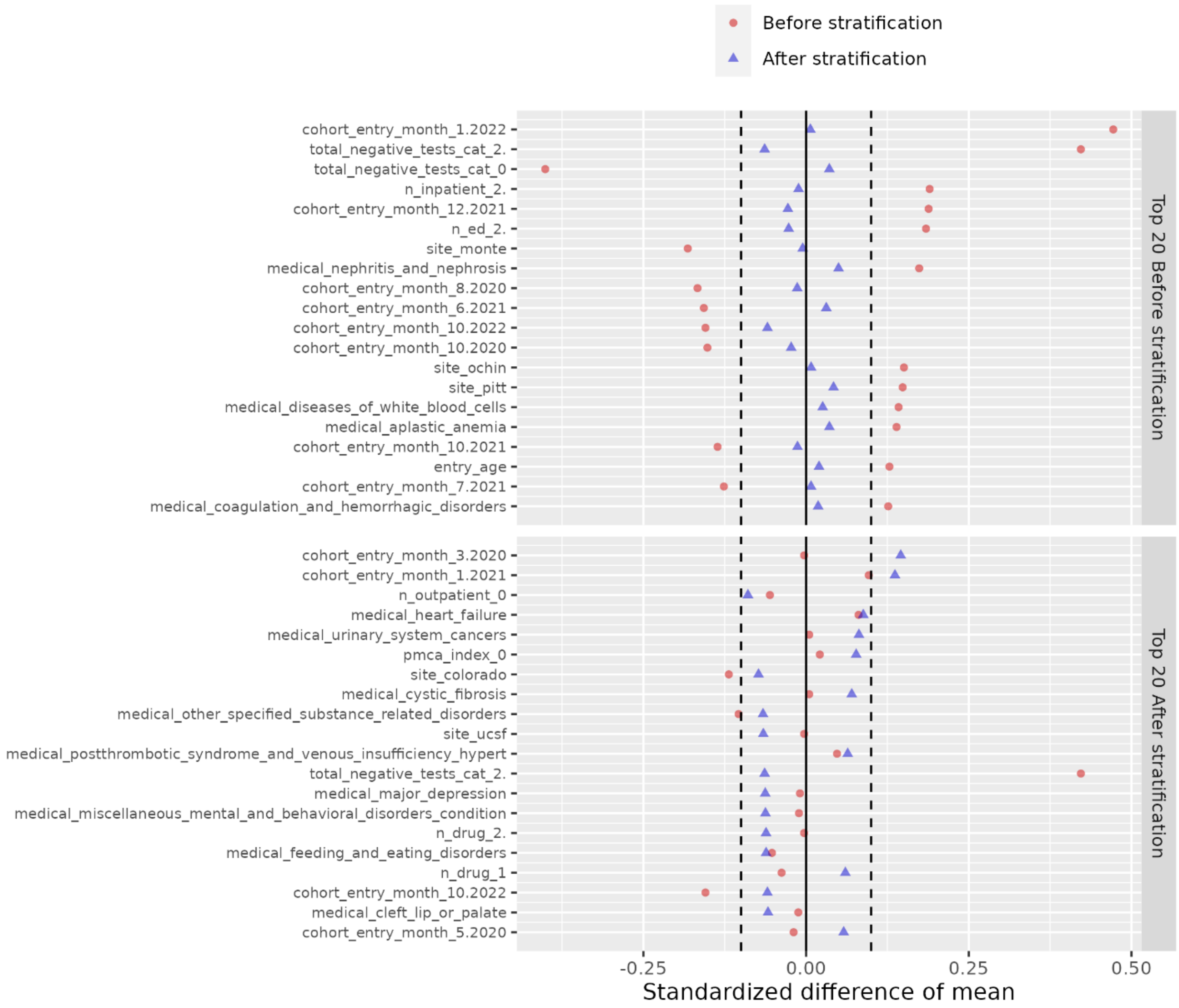

Supplement Figure 56: Patient characteristic balance before and after large-scale PS stratification with 6 strata for children and adolescents with CKD in White group. The upper panel displays the top 20 covariates with the largest standardized difference of means before stratification, while the lower panel displays the top 20 covariates with the largest standardized difference of means after stratification.

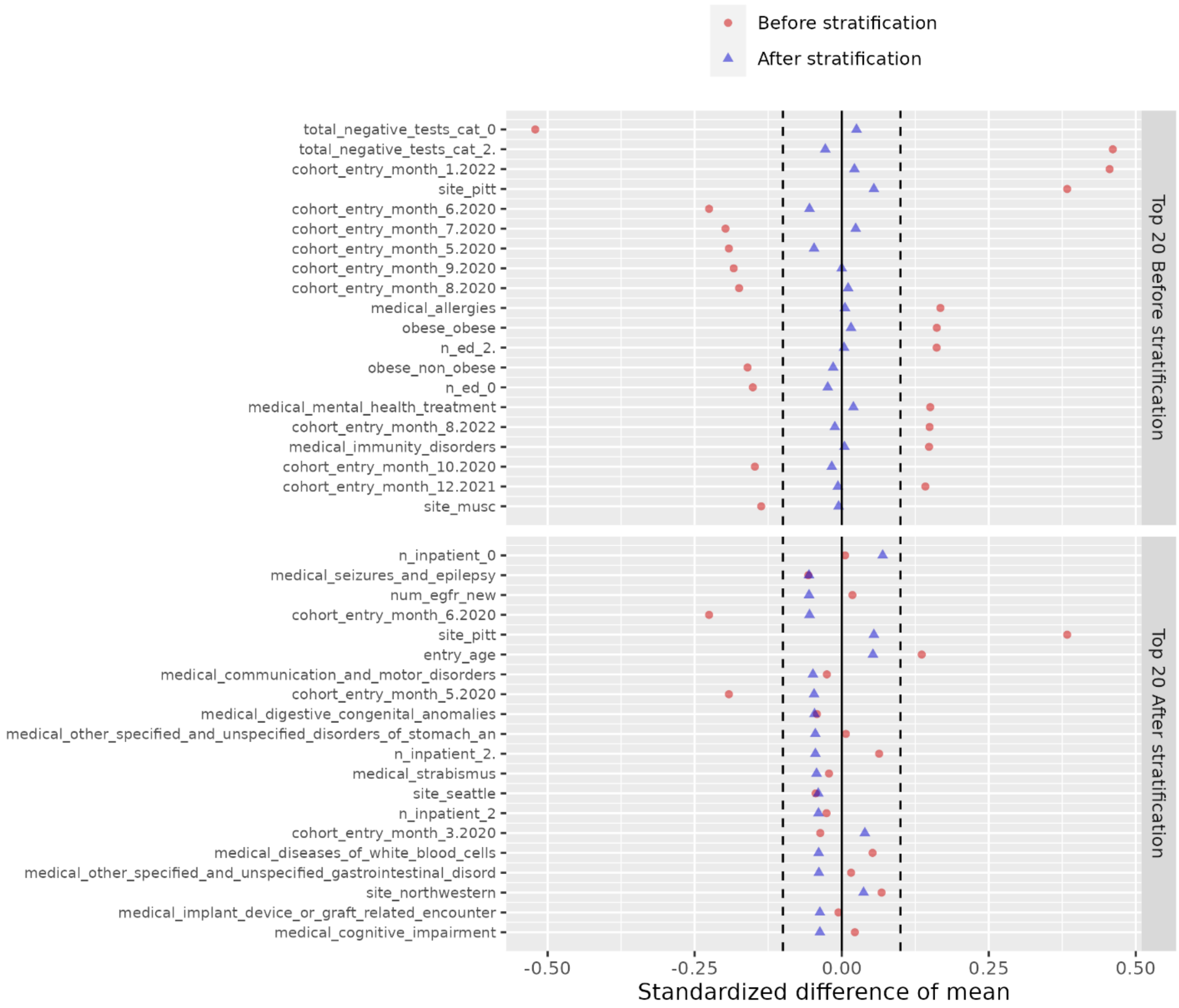

Supplement Figure 57: Patient characteristic balance before and after large-scale PS stratification with 6 strata for children and adolescents with no AKI or CKD in Asian American and Pacific Islanders group. The upper panel displays the top 20 covariates with the largest standardized difference of means before stratification, while the lower panel displays the top 20 covariates with the largest standardized difference of means after stratification.

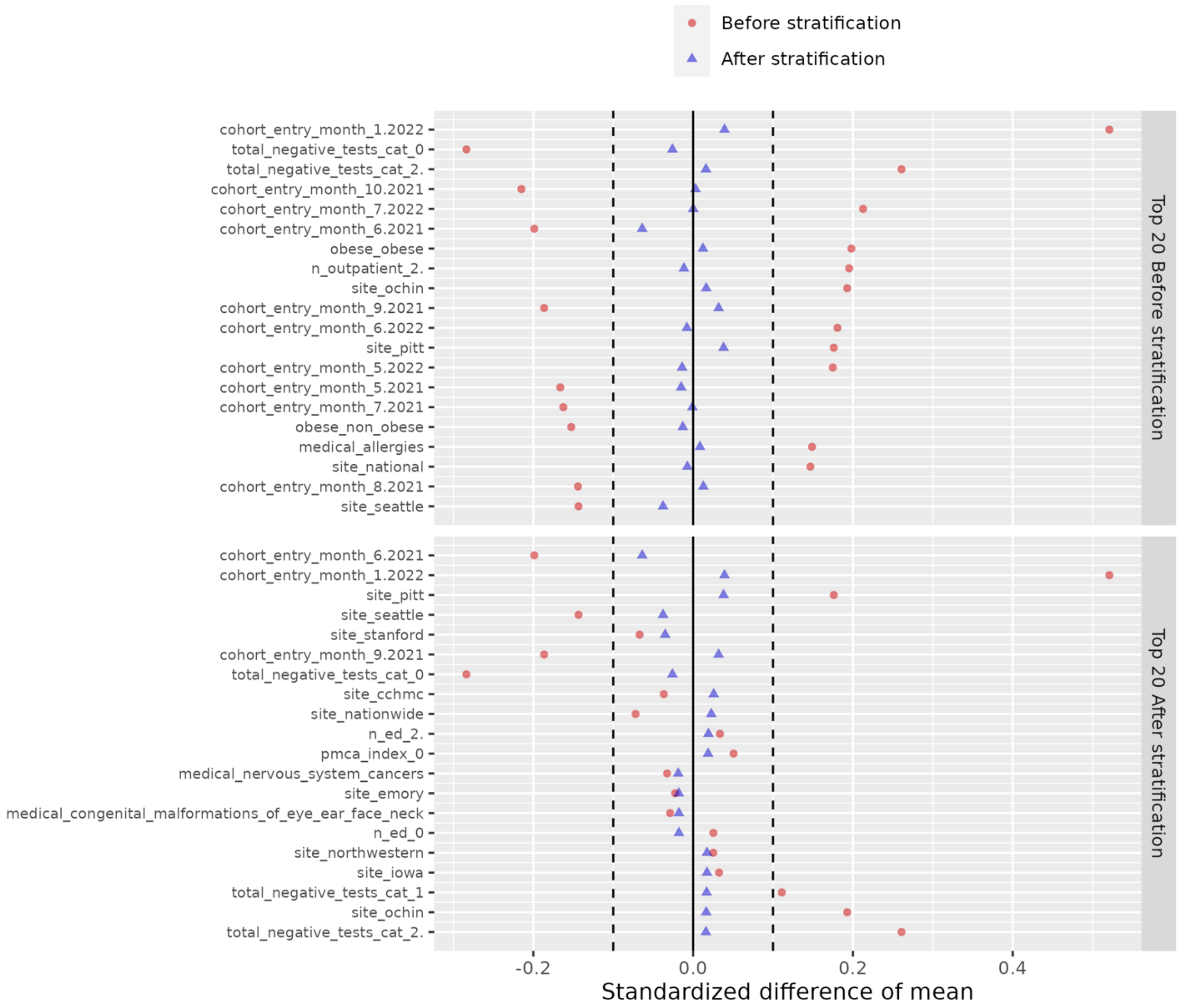

Supplement Figure 58: Patient characteristic balance before and after large-scale PS stratification with 6 strata for children and adolescents with no AKI or CKD in Black American and African American group. The upper panel displays the top 20 covariates with the largest standardized difference of means before stratification, while the lower panel displays the top 20 covariates with the largest standardized difference of means after stratification.

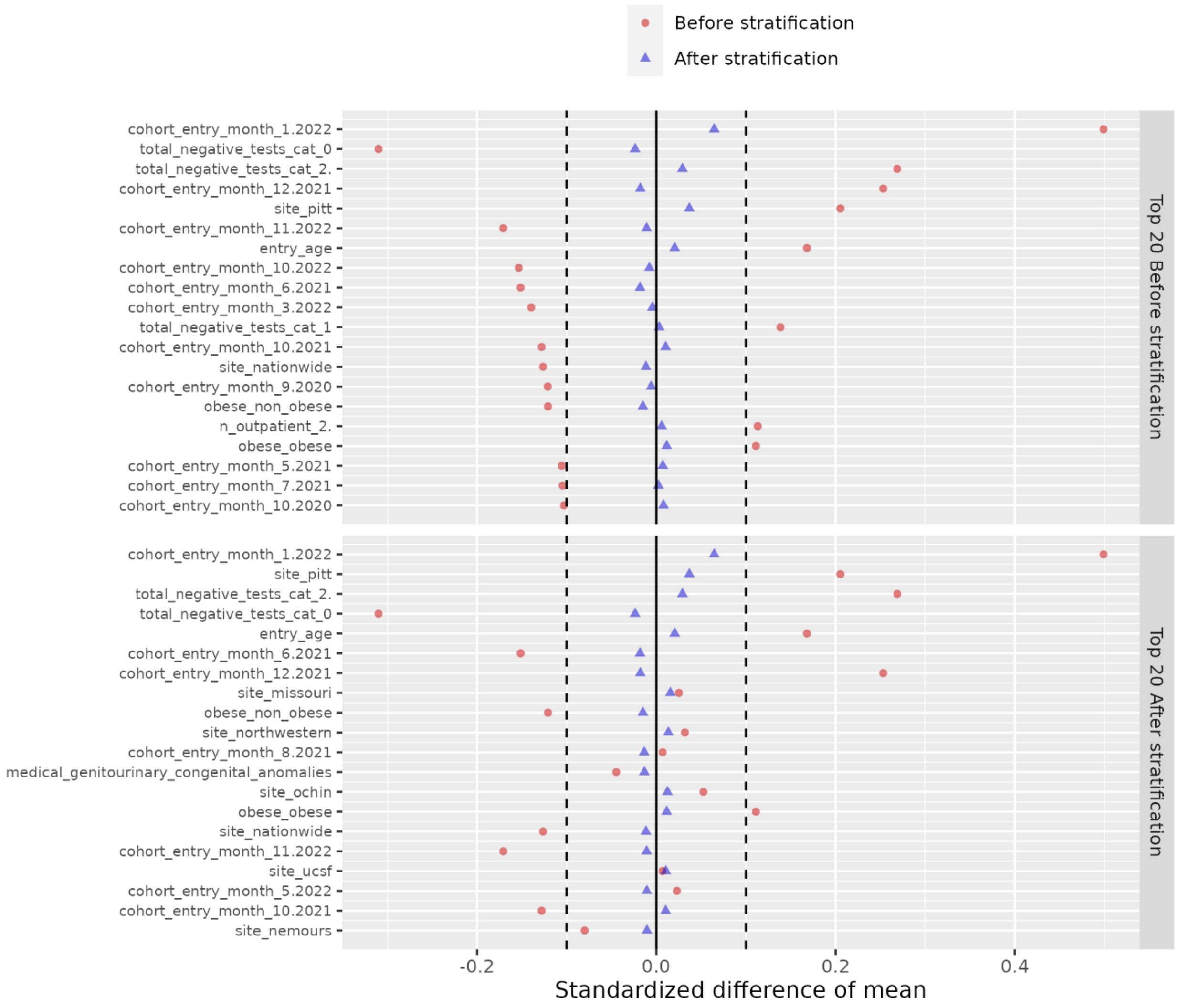

Supplement Figure 59: Patient characteristic balance before and after large-scale PS stratification with 6 strata for children and adolescents with no AKI or CKD in Hispanic group. The upper panel displays the top 20 covariates with the largest standardized difference of means before stratification, while the lower panel displays the top 20 covariates with the largest standardized difference of means after stratification.

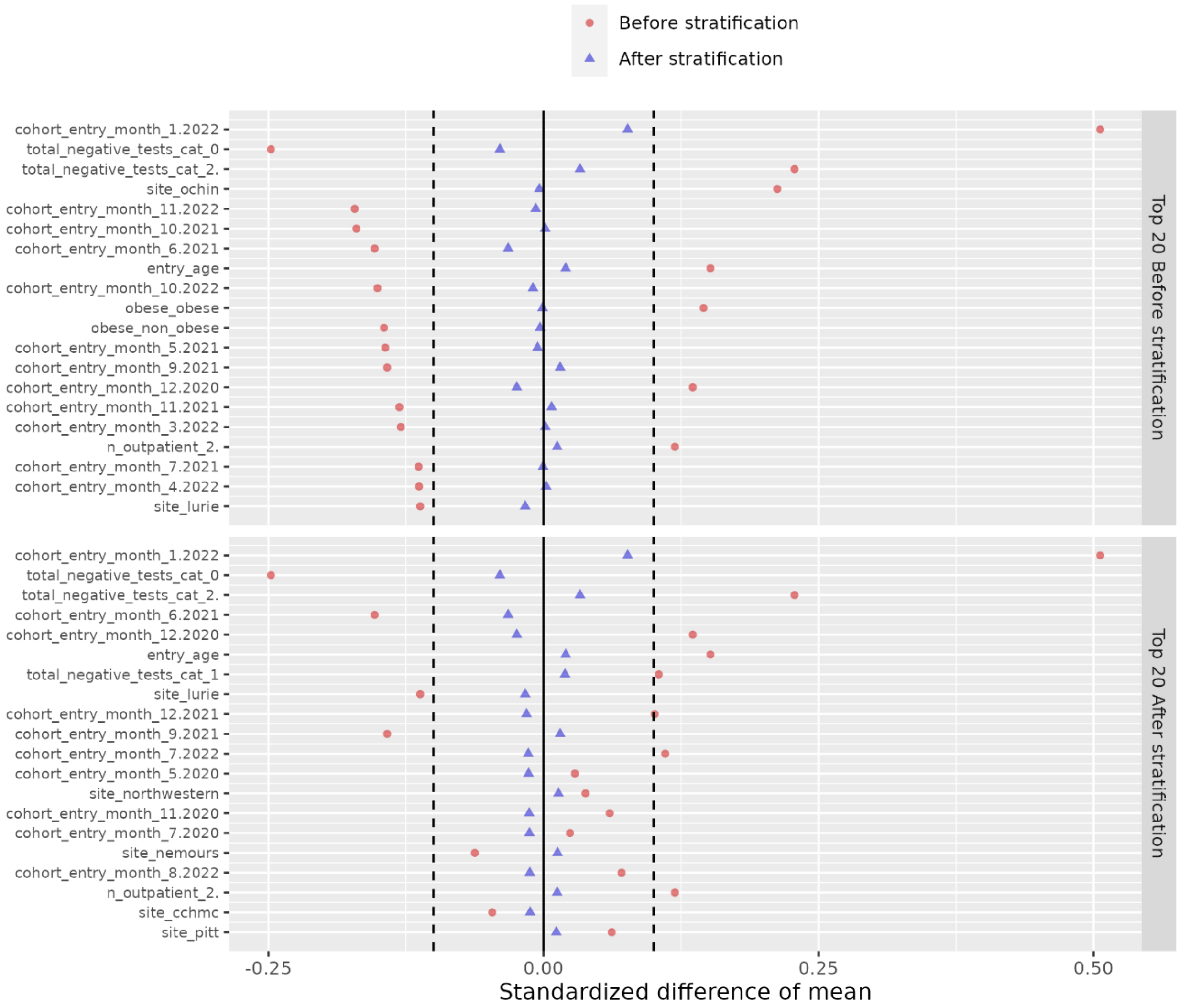

Supplement Figure 60: Patient characteristic balance before and after large-scale PS stratification with 6 strata for children and adolescents with no AKI or CKD in White group. The upper panel displays the top 20 covariates with the largest standardized difference of means before stratification, while the lower panel displays the top 20 covariates with the largest standardized difference of means after stratification.

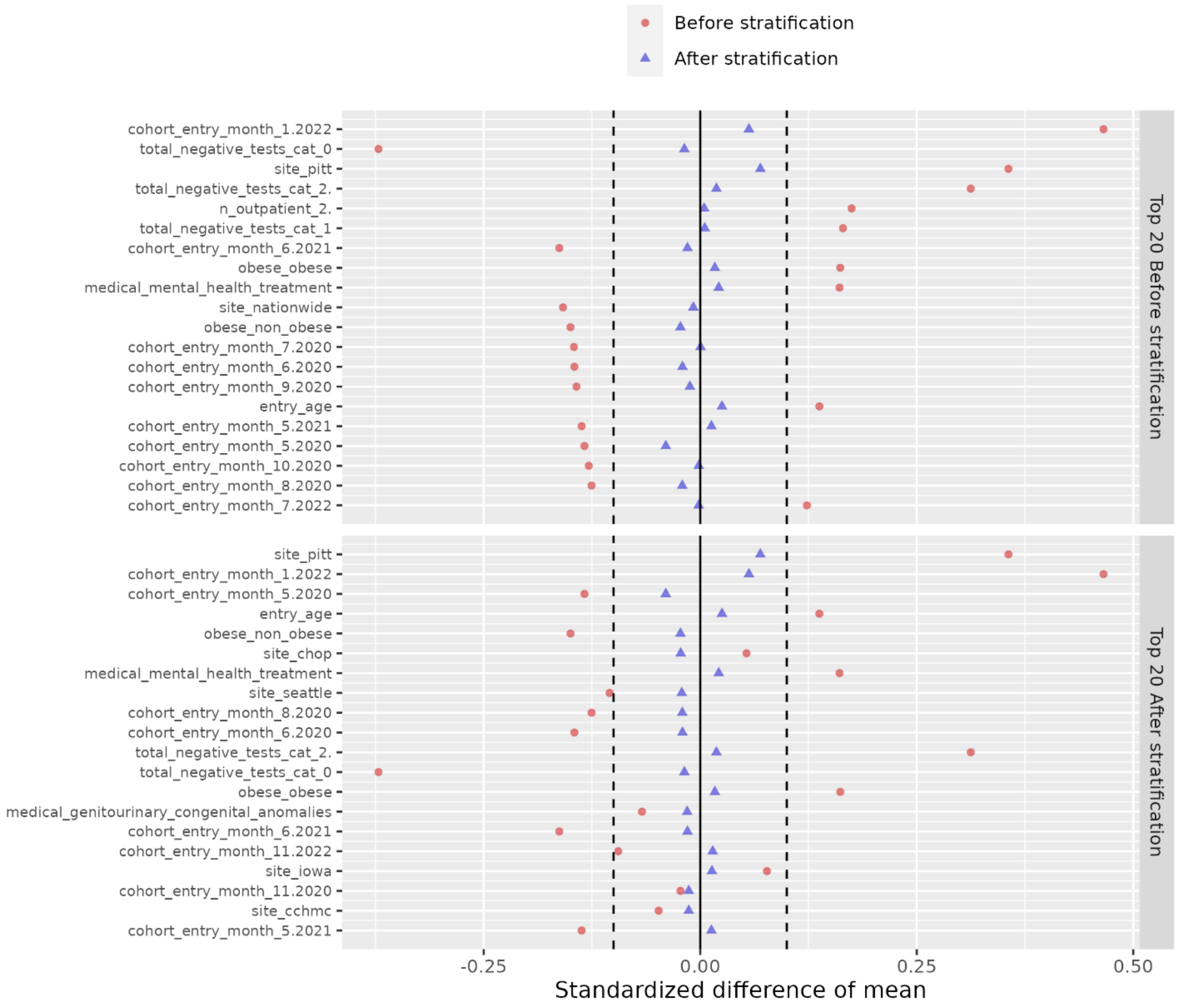

C. Hazard ratio of COVID-19 positive group compared to control group

Supplement Table 7: Estimated hazard ratio in kidney function outcomes between the COVID-19 positive cohort and the control cohort for children and adolescents in Asian American and Pacific Islanders group

Race: Asian Americans and Pacific Islanders	COVID-19 Groups Event (%)	Control Group Event (%)	HR (95% CI)
No CKD or AKI			
CKD 2+ (days 28-729)	48 (0.21%)	165 (0.24%)	1.04 [0.79, 1.38]
CKD 2+ (majority not returned to 90 and above) (days 28-729)	44 (0.19%)	143 (0.21%)	1.06 [0.78, 1.45]
CKD 2+ (not returned to 90 and above) (days 28-729)	43 (0.19%)	126 (0.19%)	1.14 [0.88, 1.48]

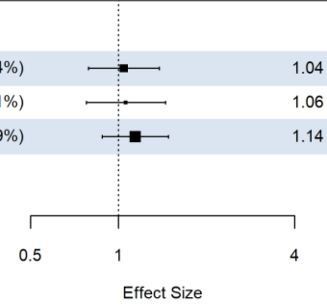

Supplement Table 8: Estimated hazard ratio in kidney function outcomes between the COVID-19 positive cohort and the control cohort for children and adolescents in Black American and African American group

Race: Non-Hispanic Black	COVID-19 Groups Event (%)	Control Group Event (%)	HR (95% CI)
No CKD or AKI			
CKD 2+ (days 28-729)	283 (0.34%)	666 (0.28%)	1.11 [0.94, 1.32]
CKD 2+ (majority not returned to 90 and above) (days 28-729)	234 (0.28%)	569 (0.24%)	1.05 [0.91, 1.21]
CKD 2+ (not returned to 90 and above) (days 28-729)	216 (0.26%)	506 (0.22%)	1.06 [0.91, 1.24]
CKD 3+ (days 28-729)	18 (0.02%)	62 (0.03%)	0.73 [0.49, 1.10]
CKD			
Composite outcome (days 28-179)	227 (15.36%)	369 (12.07%)	1.25 [1.05, 1.49]
Composite outcome (days 180-729)	289 (19.55%)	548 (17.92%)	1.16 [1.05, 1.27]
eGFR decline of 50% or more (days 180-729)	69 (4.67%)	128 (4.19%)	1.32 [0.96, 1.83]
eGFR decline of 40% or more (days 28-179)	131 (8.86%)	210 (6.87%)	1.22 [0.91, 1.64]
eGFR decline of 40% or more (days 180-729)	156 (10.55%)	328 (10.73%)	1.03 [0.88, 1.20]
eGFR decline of 30% or more (days 28-179)	224 (15.16%)	369 (12.07%)	1.23 [1.04, 1.47]
eGFR decline of 30% or more (days 180-729)	288 (19.49%)	548 (17.92%)	1.15 [1.05, 1.27]
AKI			
Composite outcome (days 180-729)	84 (7.25%)	143 (3.54%)	1.37 [0.91, 2.06]
eGFR decline of 50% or more (days 180-729)	42 (3.63%)	58 (1.43%)	1.89 [1.14, 3.12]
eGFR decline of 40% or more (days 180-729)	62 (5.35%)	90 (2.22%)	1.61 [0.95, 2.73]
eGFR decline of 30% or more (days 180-729)	82 (7.08%)	143 (3.54%)	1.33 [0.90, 1.98]

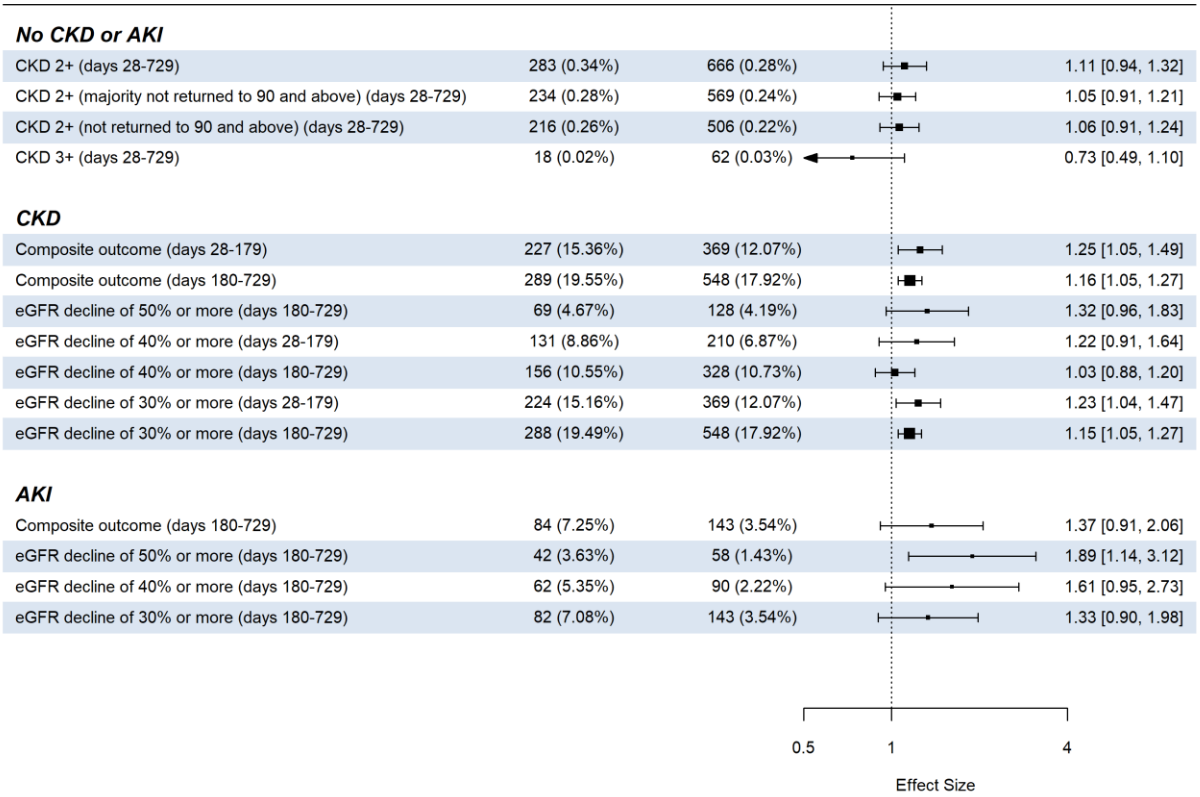

Supplement Table 9: Estimated hazard ratio in kidney function outcomes between the COVID-19 positive cohort and the control cohort for children and adolescents in Hispanic group

Race: Hispanic	COVID-19 Groups Event (%)	Control Group Event (%)	HR (95% CI)
No CKD or AKI			
CKD 2+ (days 28-729)	269 (0.25%)	591 (0.21%)	1.18 [0.96, 1.46]
CKD 2+ (majority not returned to 90 and above) (days 28-729)	232 (0.22%)	495 (0.17%)	1.21 [1.02, 1.45]
CKD 2+ (not returned to 90 and above) (days 28-729)	209 (0.2%)	429 (0.15%)	1.26 [1.02, 1.56]
CKD 3+ (days 28-729)	39 (0.04%)	47 (0.02%)	2.60 [1.82, 3.72]
CKD 3+ (majority not returned to 90 and above) (days 28-729)	28 (0.03%)	36 (0.01%)	2.53 [1.68, 3.81]
CKD 3+ (not returned to 90 and above) (days 28-729)	31 (0.03%)	40 (0.01%)	2.39 [1.52, 3.76]
CKD 3+ (not returned to 60 and above) (days 28-729)	25 (0.02%)	34 (0.01%)	2.37 [1.50, 3.76]
CKD			
Composite outcome (days 28-179)	127 (13.11%)	229 (10.25%)	1.15 [0.94, 1.41]
Composite outcome (days 180-729)	173 (17.85%)	325 (14.55%)	1.22 [1.09, 1.36]
eGFR decline of 50% or more (days 180-729)	36 (3.72%)	92 (4.12%)	1.00 [0.67, 1.48]
eGFR decline of 40% or more (days 28-179)	67 (6.91%)	129 (5.77%)	1.05 [0.69, 1.60]
eGFR decline of 40% or more (days 180-729)	88 (9.08%)	165 (7.39%)	1.34 [0.98, 1.84]
eGFR decline of 30% or more (days 28-179)	126 (13%)	229 (10.25%)	1.14 [0.93, 1.41]
eGFR decline of 30% or more (days 180-729)	170 (17.54%)	325 (14.55%)	1.20 [1.04, 1.37]
AKI			
Composite outcome (days 180-729)	61 (8.39%)	128 (4.79%)	1.24 [0.97, 1.59]
eGFR decline of 50% or more (days 180-729)	27 (3.71%)	53 (1.98%)	1.51 [0.93, 2.46]
eGFR decline of 40% or more (days 180-729)	42 (5.78%)	86 (3.22%)	1.37 [0.84, 2.23]
eGFR decline of 30% or more (days 180-729)	61 (8.39%)	128 (4.79%)	1.24 [0.97, 1.59]

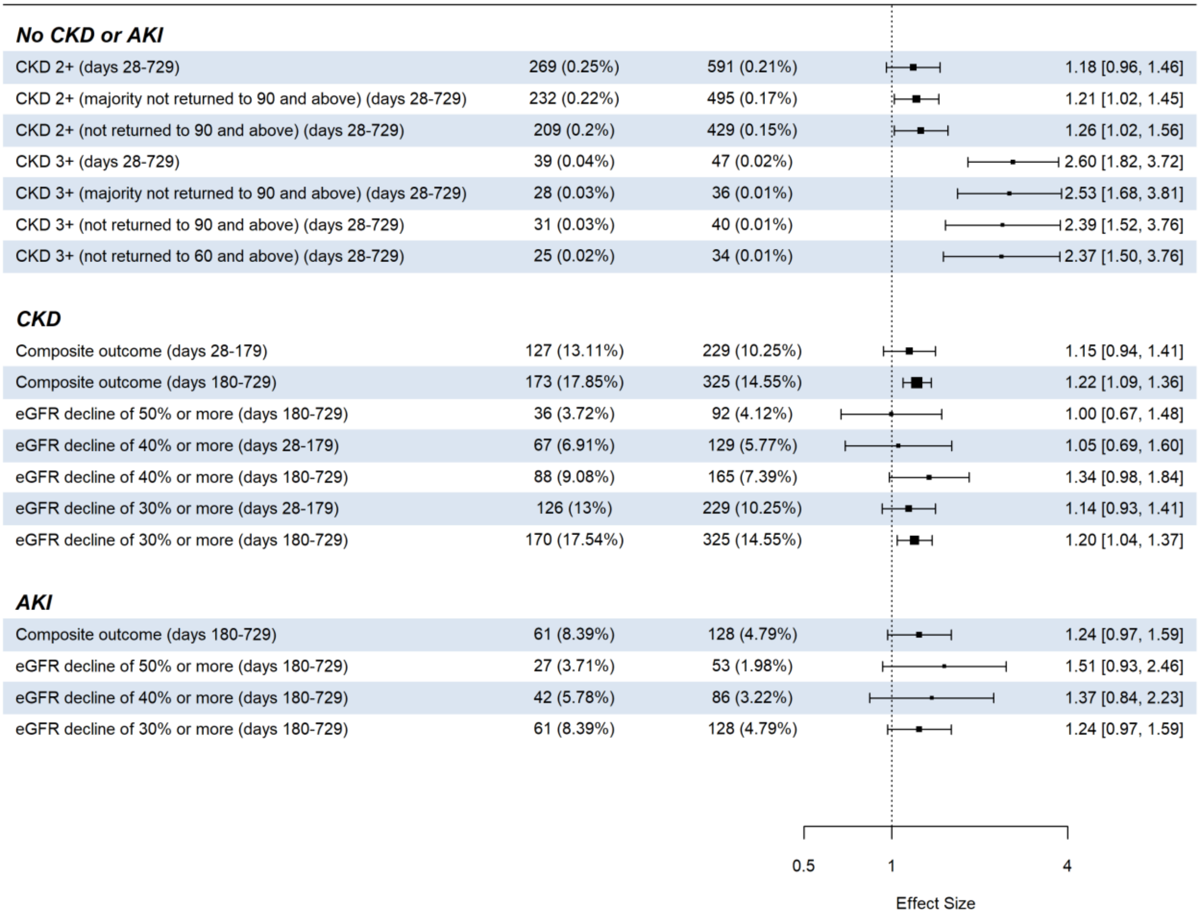

Supplement Table 10: Estimated hazard ratio in kidney function outcomes between the COVID-19 positive cohort and the control cohort for children and adolescents in White group

Race: Non-Hispanic White	COVID-19 Groups Event (%)	Control Group Event (%)	HR (95% CI)
No CKD or AKI			
CKD 2+ (days 28-729)	863 (0.41%)	2147 (0.34%)	1.23 [1.14, 1.33]
CKD 2+ (majority not returned to 90 and above) (days 28-729)	753 (0.36%)	1775 (0.28%)	1.27 [1.18, 1.36]
CKD 2+ (not returned to 90 and above) (days 28-729)	701 (0.34%)	1599 (0.26%)	1.27 [1.17, 1.38]
CKD 3+ (days 28-729)	56 (0.03%)	149 (0.02%)	1.38 [1.17, 1.62]
CKD 3+ (majority not returned to 90 and above) (days 28-729)	44 (0.02%)	110 (0.02%)	1.47 [1.34, 1.60]
CKD 3+ (not returned to 90 and above) (days 28-729)	48 (0.02%)	130 (0.02%)	1.30 [1.20, 1.42]
CKD			
Composite outcome (days 28-179)	482 (13.86%)	957 (11.83%)	1.11 [1.00, 1.23]
Composite outcome (days 180-729)	626 (18%)	1366 (16.88%)	1.16 [1.05, 1.28]
eGFR decline of 50% or more (days 28-179)	96 (2.76%)	217 (2.68%)	0.86 [0.69, 1.07]
eGFR decline of 50% or more (days 180-729)	120 (3.45%)	299 (3.7%)	0.97 [0.79, 1.18]
eGFR decline of 40% or more (days 28-179)	250 (7.19%)	513 (6.34%)	0.99 [0.87, 1.13]
eGFR decline of 40% or more (days 180-729)	329 (9.46%)	715 (8.84%)	1.16 [1.03, 1.30]
eGFR decline of 30% or more (days 28-179)	476 (13.69%)	957 (11.83%)	1.09 [0.99, 1.21]
eGFR decline of 30% or more (days 180-729)	622 (17.88%)	1366 (16.88%)	1.15 [1.04, 1.27]
AKI			
Composite outcome (days 180-729)	185 (11.84%)	425 (7.42%)	1.24 [1.10, 1.39]

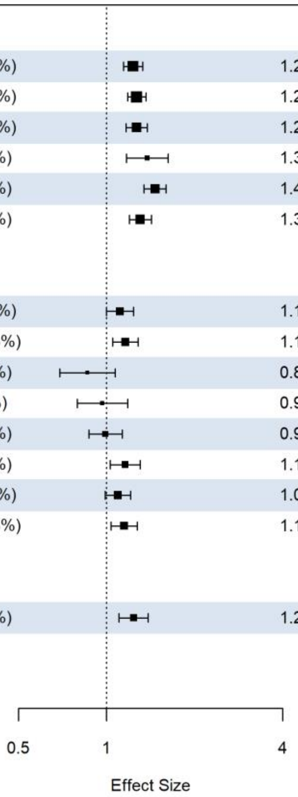

eAppendix 7. Sensitivity Analysis for Subgroups With and Without Obesity

We conducted sensitivity analyses on both cohorts stratified by the obesity status (i.e., obsess or non-obese). We performed the same PS stratification procedure and used Cox proportional hazard model to estimate the hazard ratio.

A. Empirical equipoise assessment

Supplement Figure 61: Preference score distributions of COVID-19 positive and negative groups for non-obese children and adolescents with AKI. A greater convergence of these distributions indicates a higher similarity in the predicted likelihood of being infected between the COVID-19 positive (red) and negative (blue) participants.

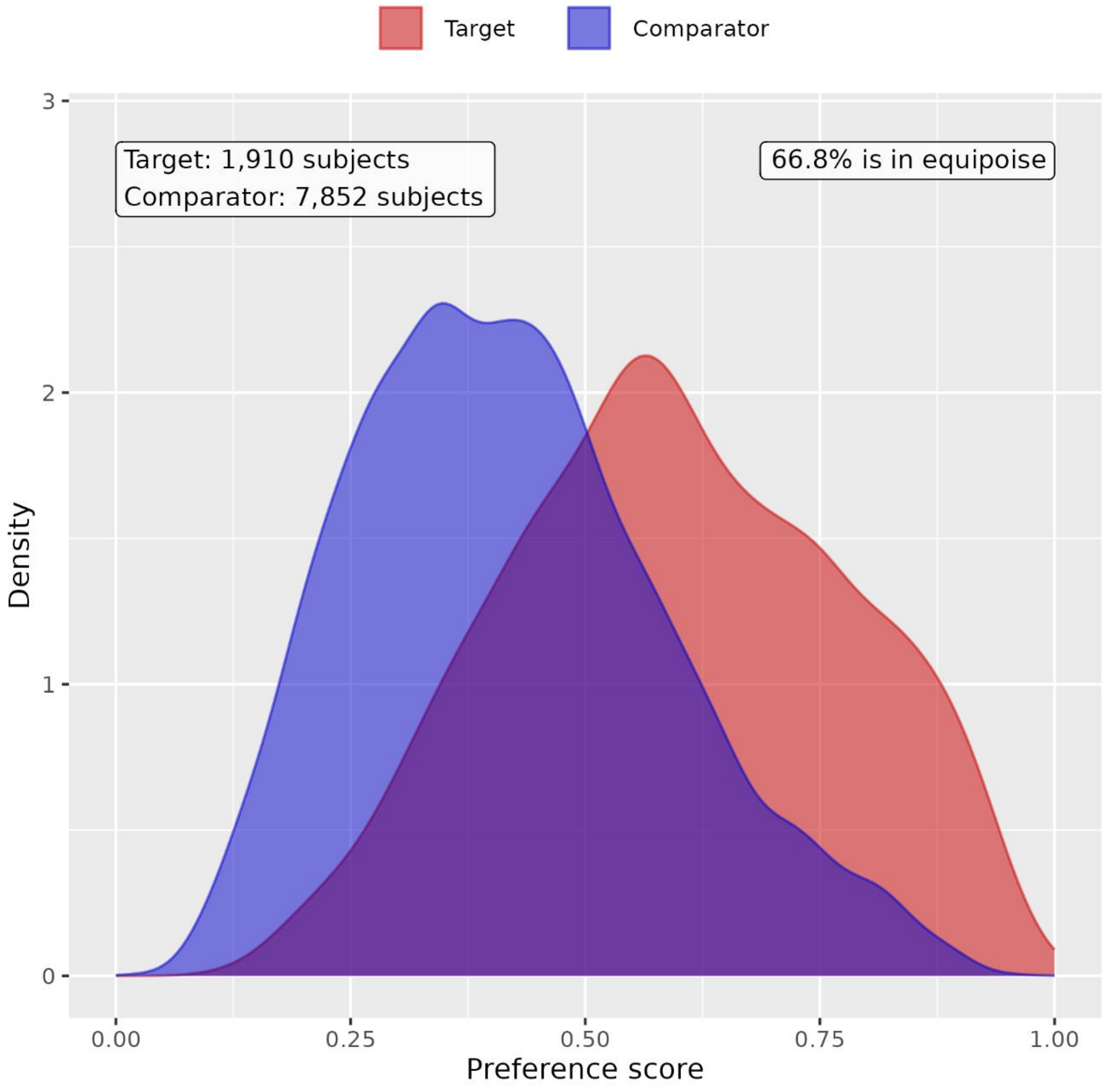

Supplement Figure 62: Preference score distributions of COVID-19 positive and negative groups for obese children and adolescents with AKI. A greater convergence of these

distributions indicates a higher similarity in the predicted likelihood of being infected between the COVID-19 positive (red) and negative (blue) participants.

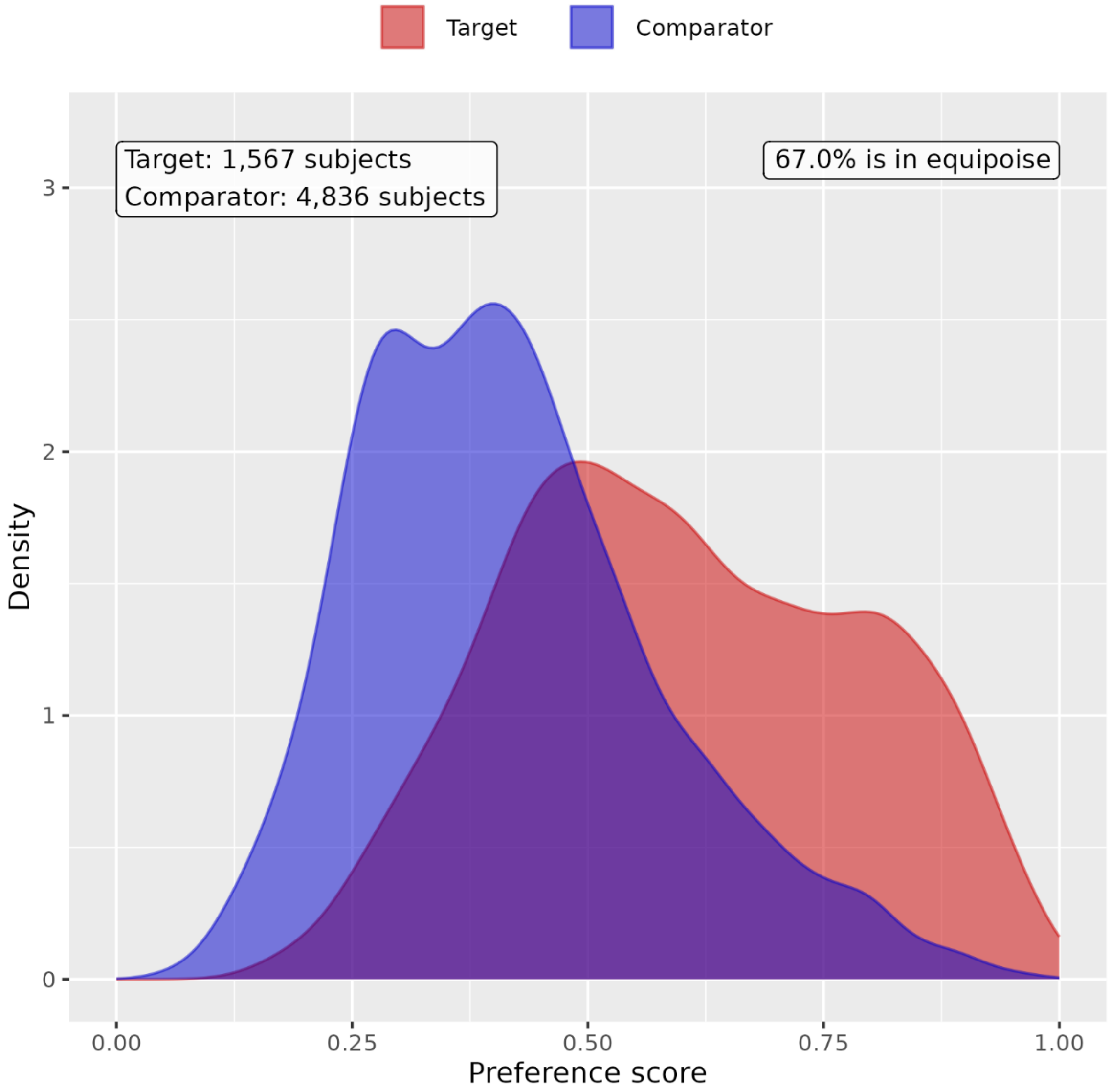

Supplement Figure 63: Preference score distributions of COVID-19 positive and negative groups for non-obese children and adolescents with CKD. A greater convergence of these distributions indicates a higher similarity in the predicted likelihood of being infected between the COVID-19 positive (red) and negative (blue) participants.

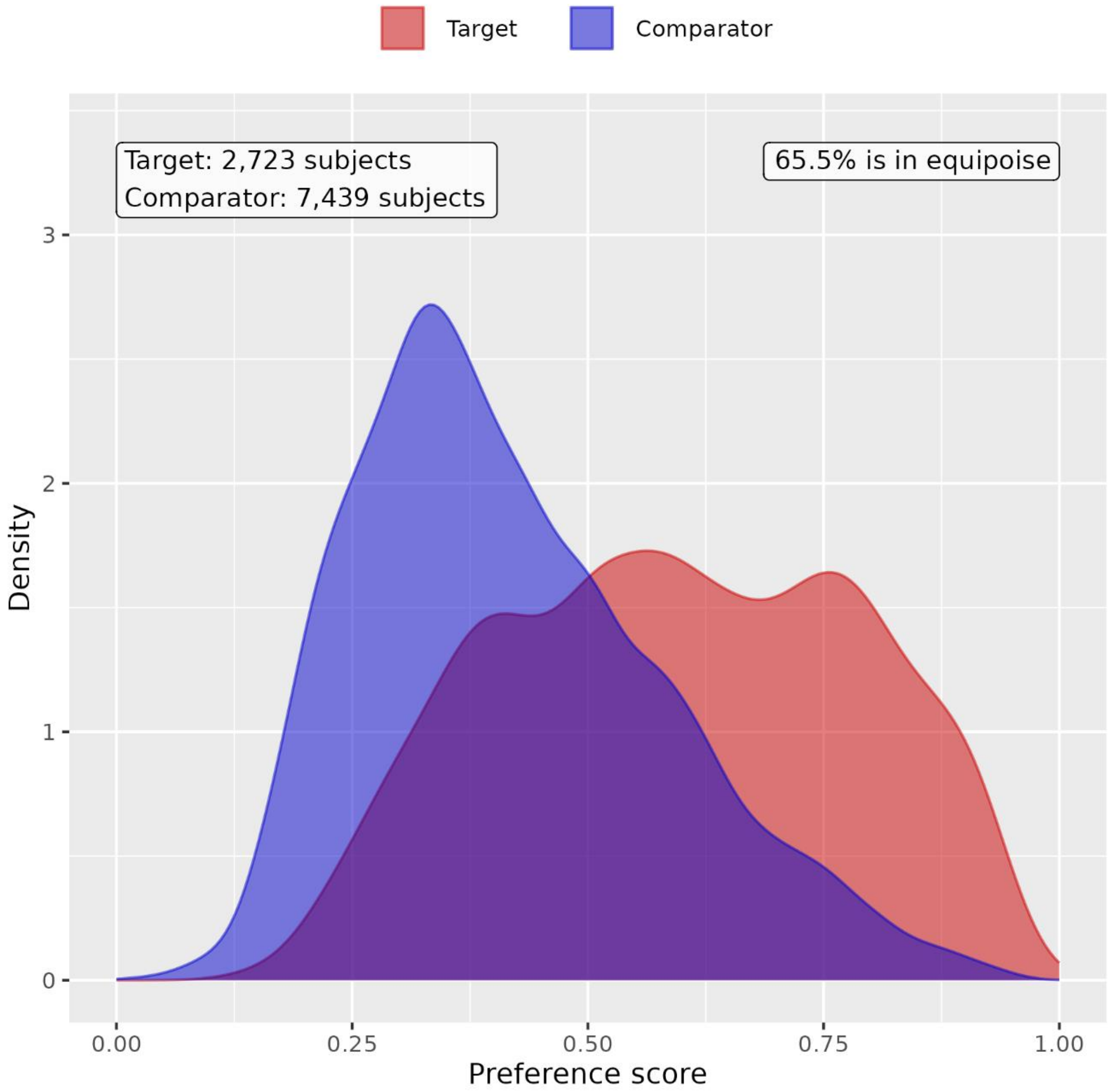

Supplement Figure 64: Preference score distributions of COVID-19 positive and negative groups for obese children and adolescents with CKD. A greater convergence of these distributions indicates a higher similarity in the predicted likelihood of being infected between the COVID-19 positive (red) and negative (blue) participants.

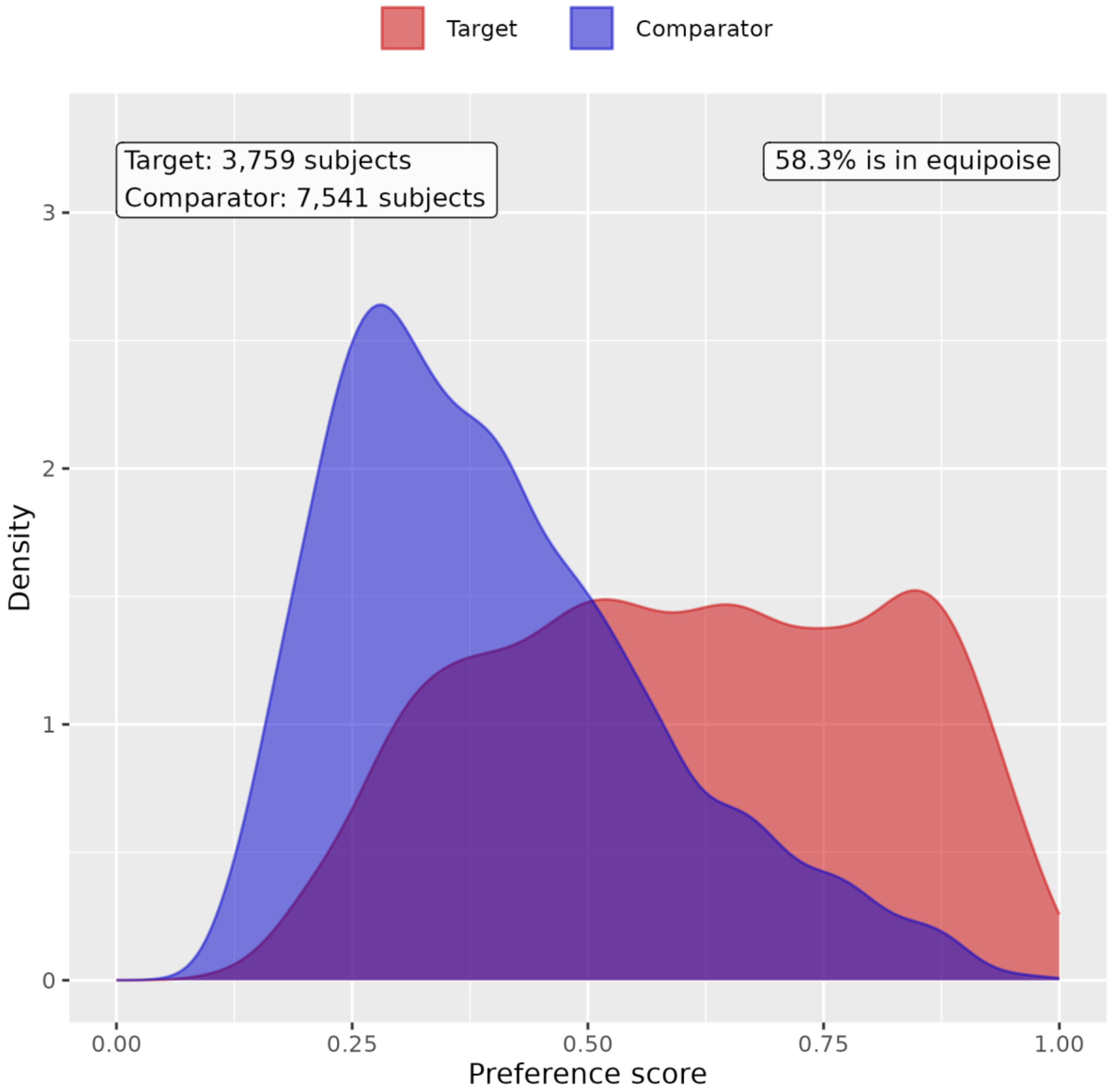

Supplement Figure 65: Preference score distributions of COVID-19 positive and negative groups for non-obese children and adolescents with no AKI or CKD. A greater convergence of these distributions indicates a higher similarity in the predicted likelihood of being infected between the COVID-19 positive (red) and negative (blue) participants.

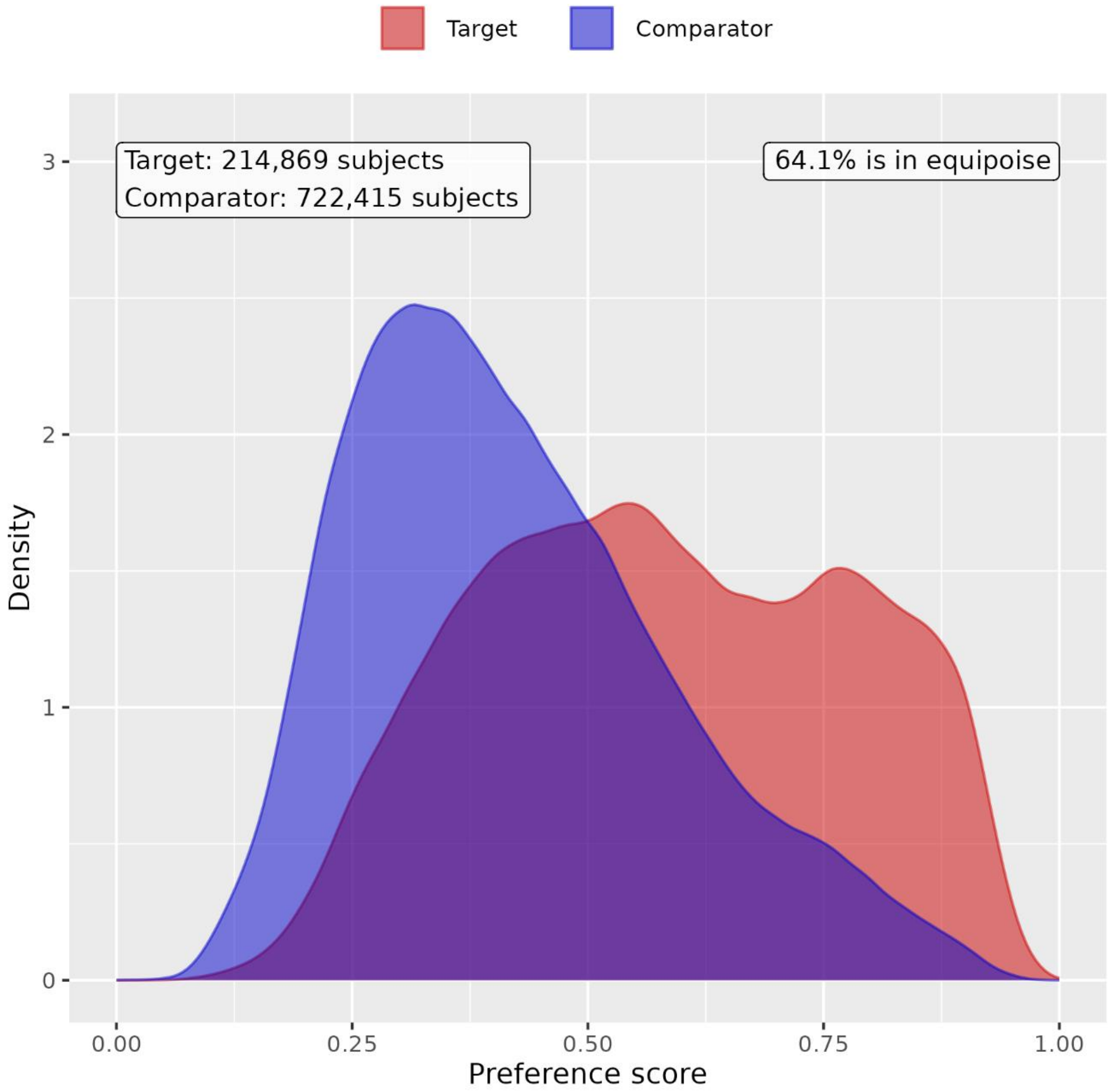

Supplement Figure 66: Preference score distributions of COVID-19 positive and negative groups for obese children and adolescents with no AKI or CKD. A greater convergence of these distributions indicates a higher similarity in the predicted likelihood of being infected between the COVID-19 positive (red) and negative (blue) participants.

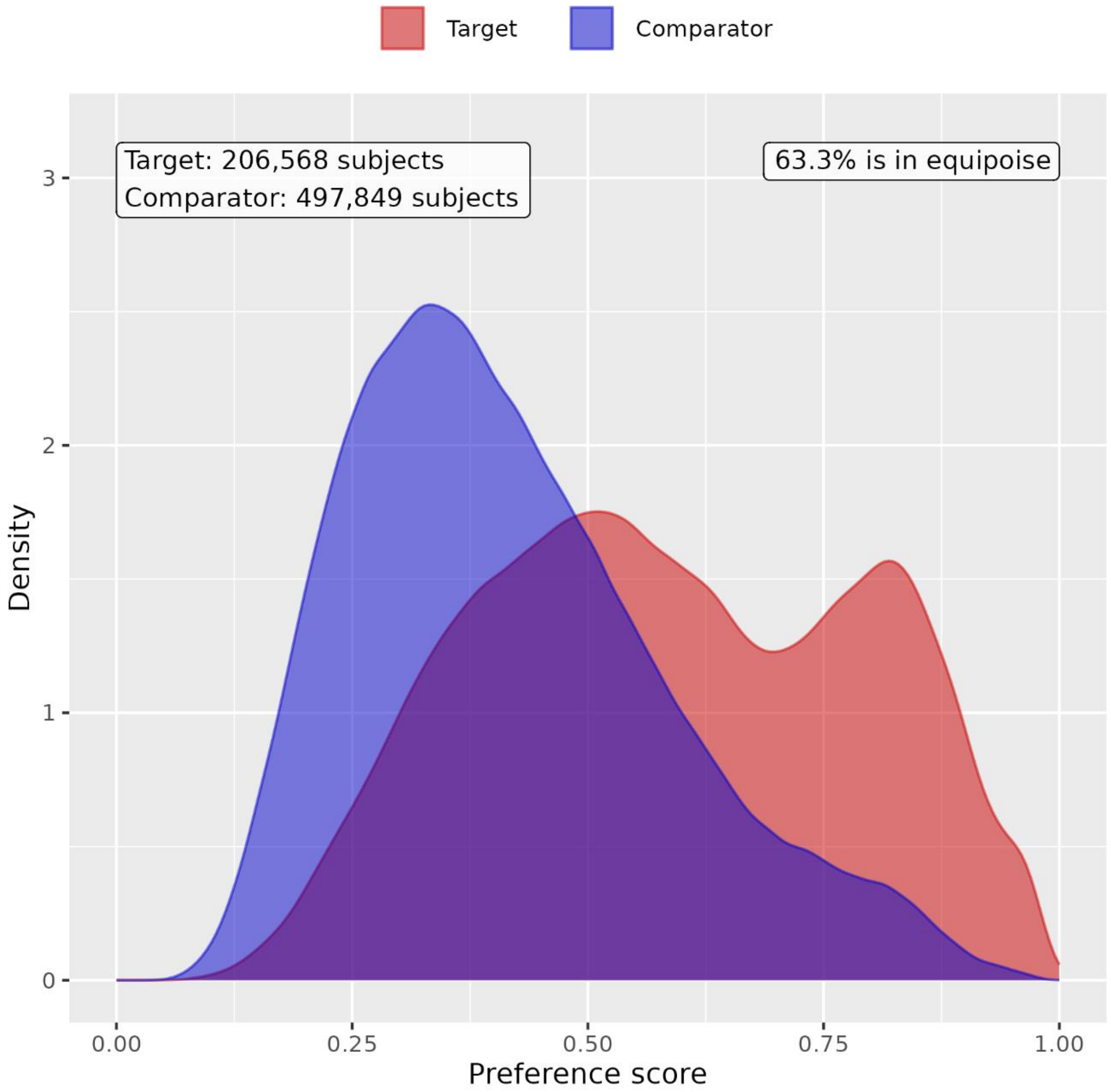

B. Patient characteristic balance

Supplement Figure 67: Patient characteristic balance before and after large-scale PS stratification with 6 strata for non-obese children and adolescents with AKI. The upper panel displays the top 20 covariates with the largest standardized difference of means before stratification, while the lower panel displays the top 20 covariates with the largest standardized difference of means after stratification.

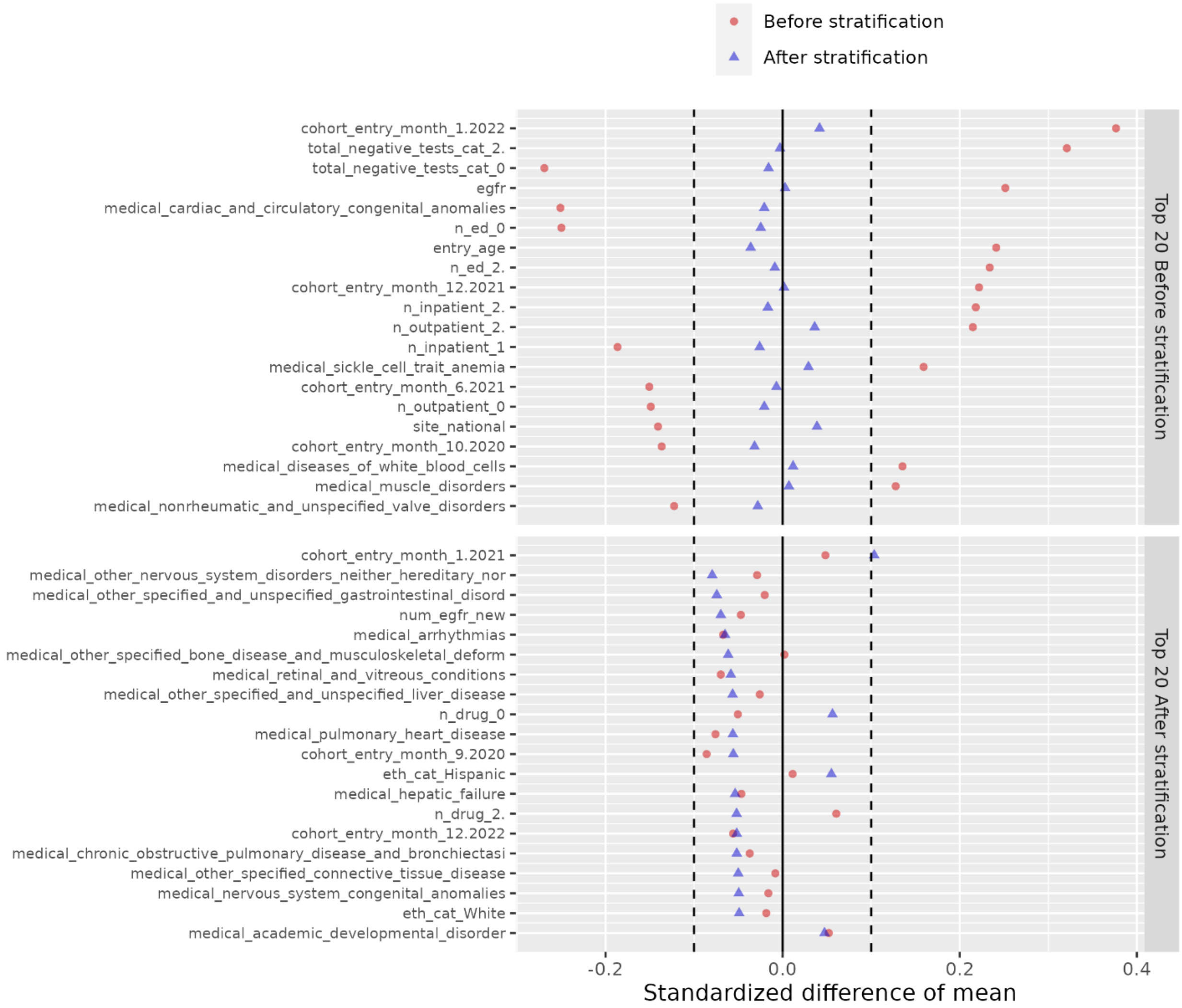

Supplement Figure 68: Patient characteristic balance before and after large-scale PS stratification with 6 strata for obese children and adolescents with AKI. The upper panel displays the top 20 covariates with the largest standardized difference of means before stratification, while the lower panel displays the top 20 covariates with the largest standardized difference of means after stratification.

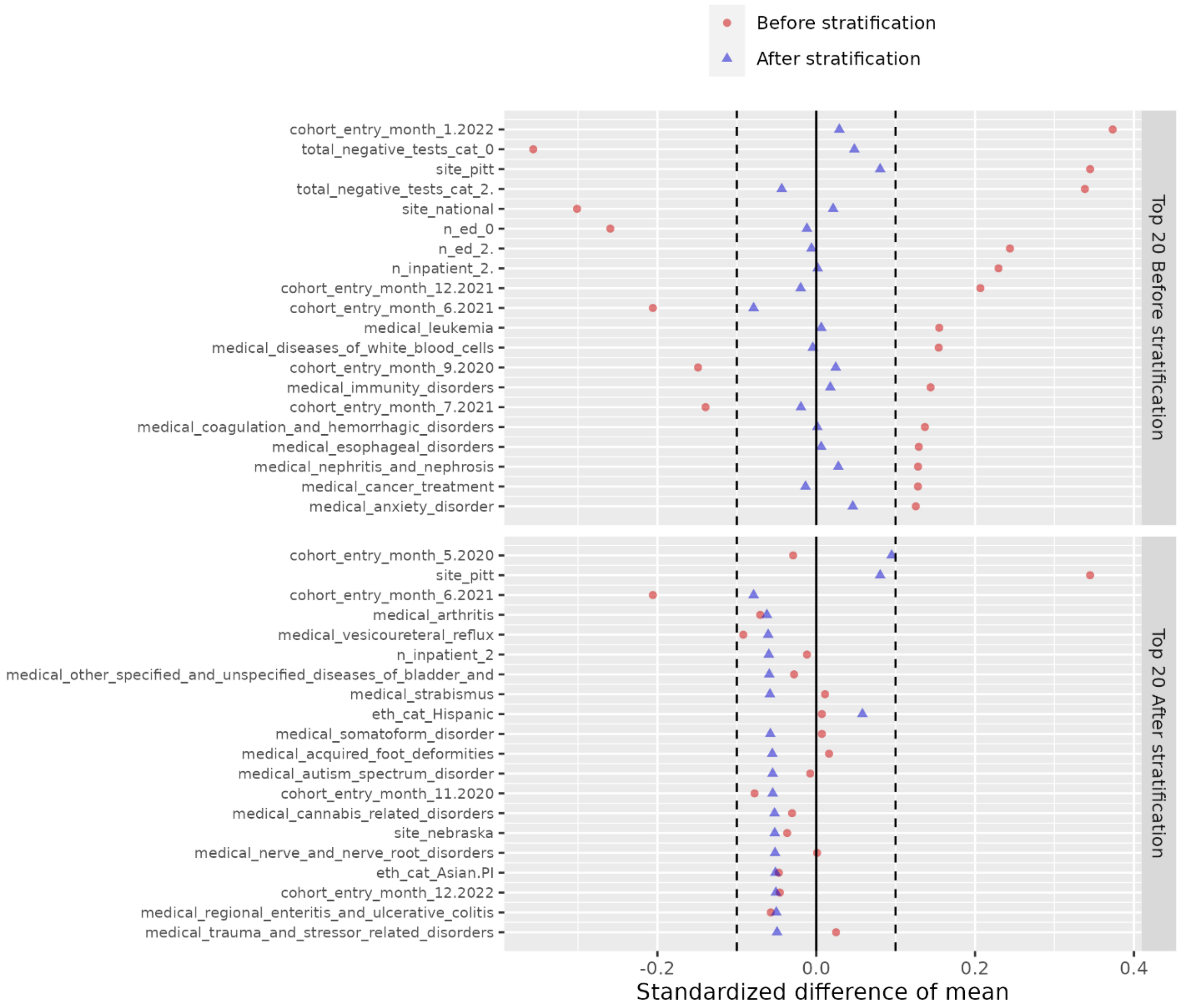

Supplement Figure 69: Patient characteristic balance before and after large-scale PS stratification with 6 strata for non-obese children and adolescents with CKD. The upper panel displays the top 20 covariates with the largest standardized difference of means before stratification, while the lower panel displays the top 20 covariates with the largest standardized difference of means after stratification.

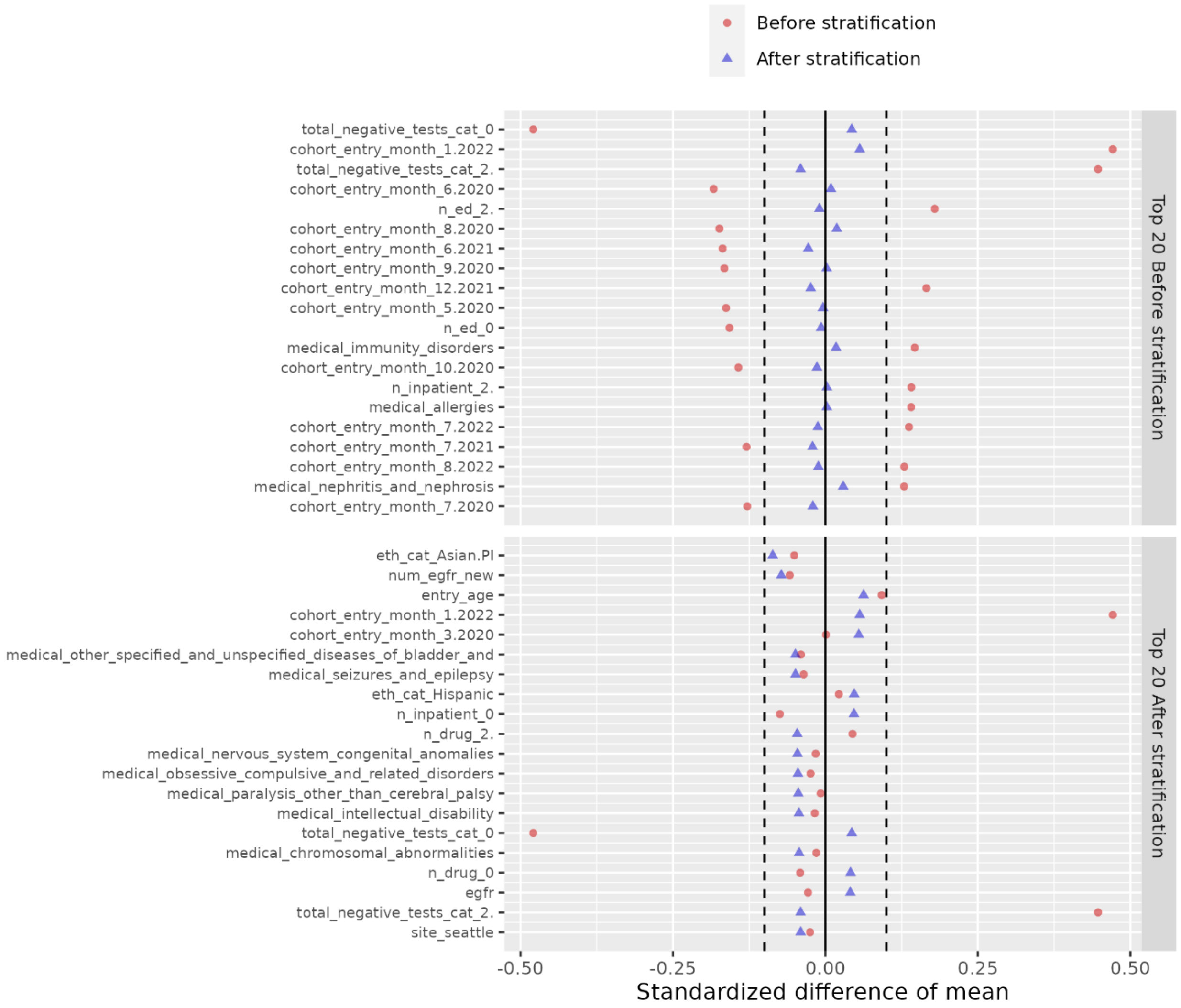

Supplement Figure 70: Patient characteristic balance before and after large-scale PS stratification with 6 strata for obese children and adolescents with CKD. The upper panel displays the top 20 covariates with the largest standardized difference of means before stratification, while the lower panel displays the top 20 covariates with the largest standardized difference of means after stratification.

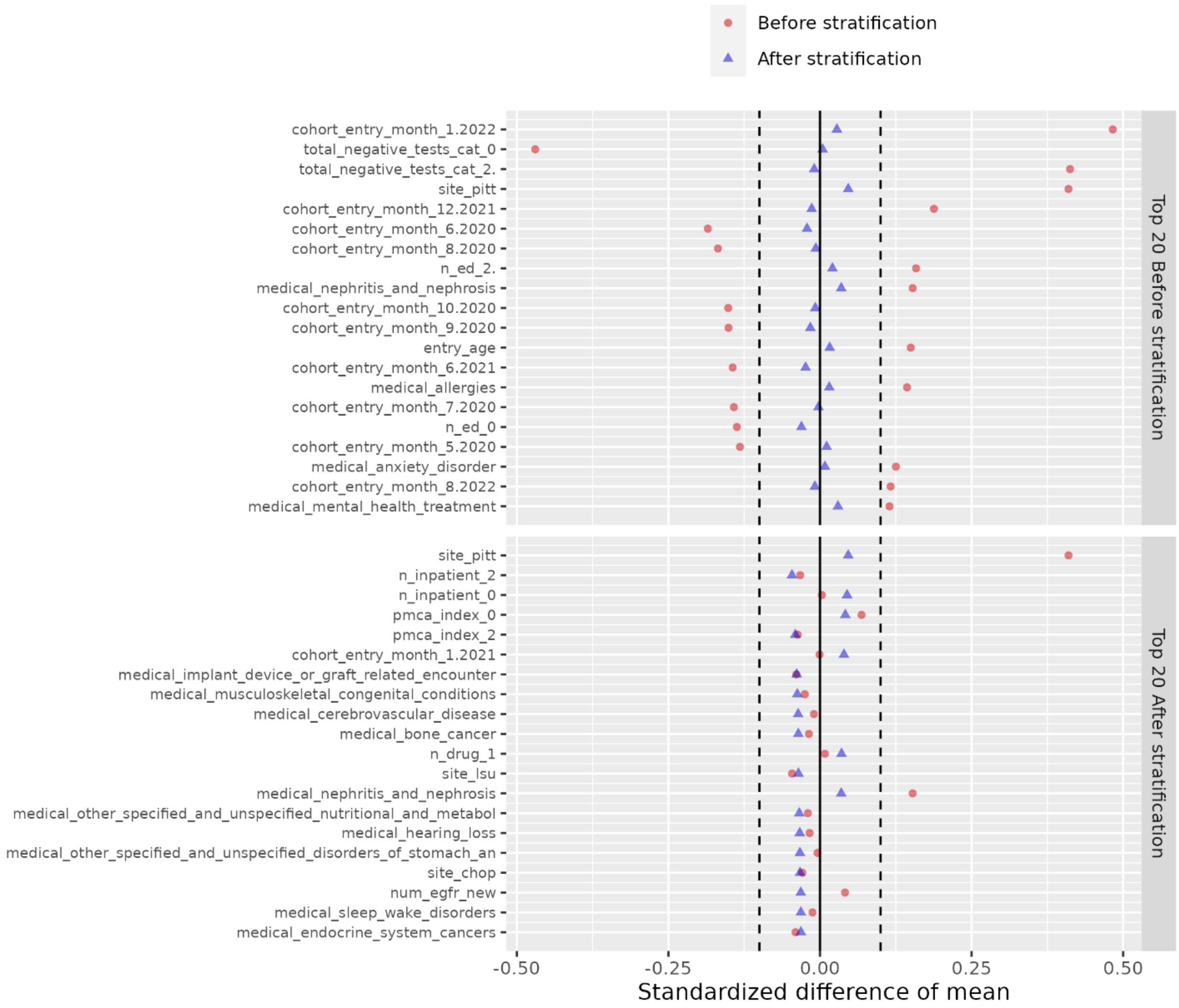

Supplement Figure 71: Patient characteristic balance before and after large-scale PS stratification with 6 strata for non-obese children and adolescents with no AKI or CKD. The upper panel displays the top 20 covariates with the largest standardized difference of means before stratification, while the lower panel displays the top 20 covariates with the largest standardized difference of means after stratification.

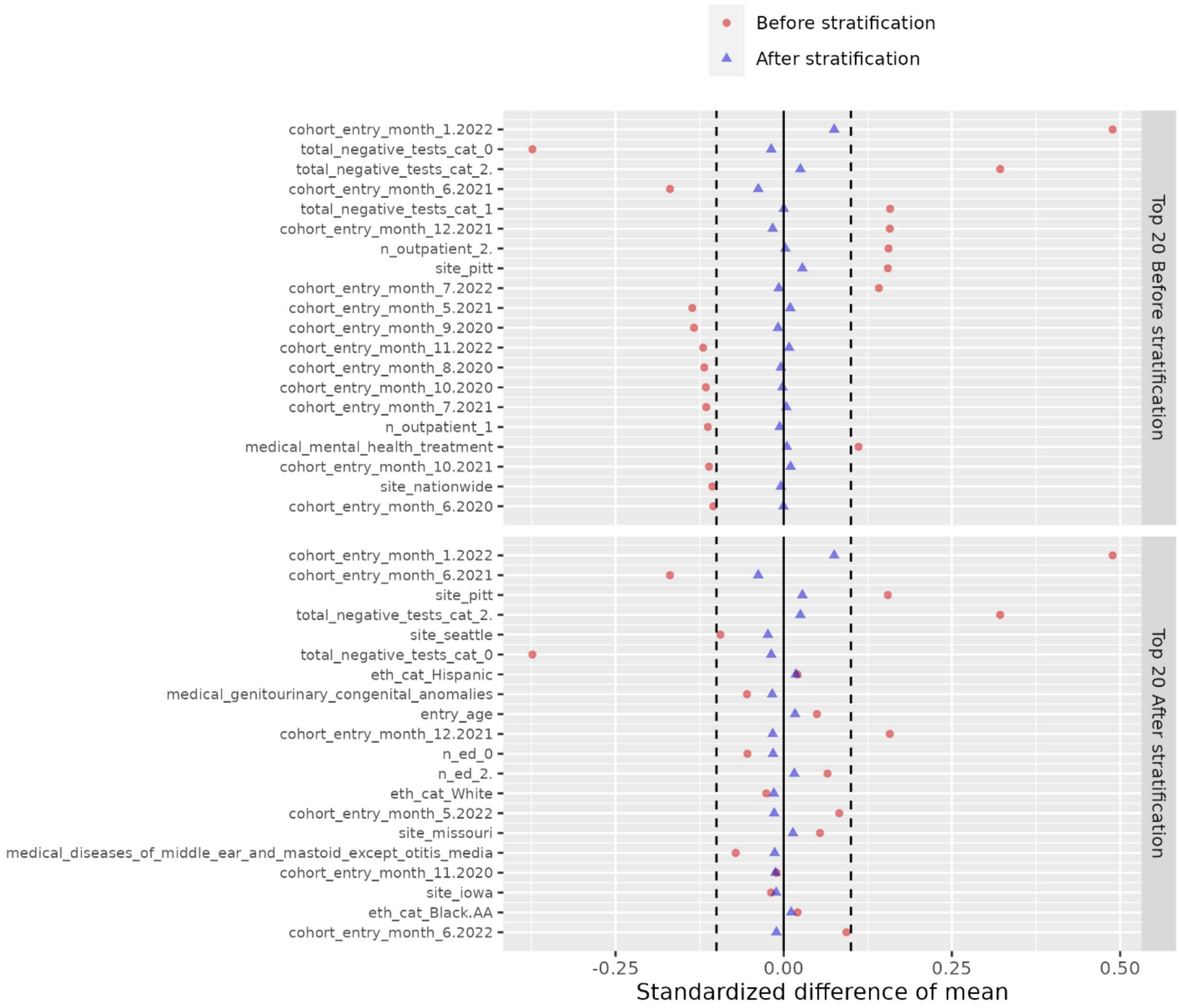

Supplement Figure 72: Patient characteristic balance before and after large-scale PS stratification with 6 strata for obese children and adolescents with no AKI or CKD. The upper panel displays the top 20 covariates with the largest standardized difference of means before stratification, while the lower panel displays the top 20 covariates with the largest standardized difference of means after stratification.

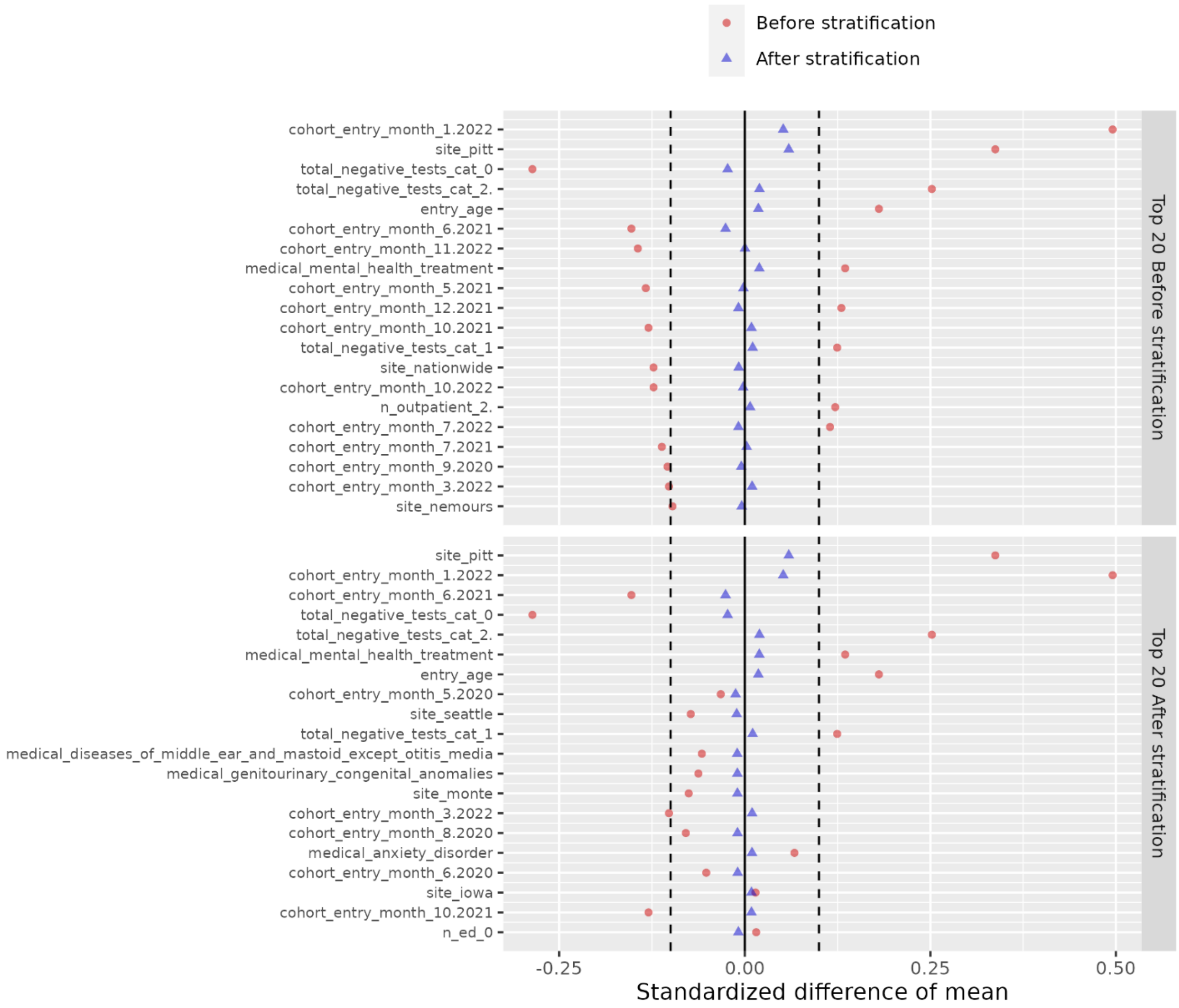

C. Hazard ratio of COVID-19 positive group compared to control group

Supplement Table 11: Estimated hazard ratio in kidney function outcomes between the COVID-19 positive cohort and the control cohort for non-obese children and adolescents

Obesity: Non-obese	COVID-19 Groups Event (%)	Control Group Event (%)	HR (95% CI)
No CKD or AKI			
CKD 2+ (days 28-729)	726 (0.34%)	2097 (0.29%)	1.18 [1.06, 1.31]
CKD 2+ (majority not returned to 90 and above) (days 28-729)	632 (0.29%)	1758 (0.24%)	1.20 [1.07, 1.35]
CKD 2+ (not returned to 90 and above) (days 28-729)	576 (0.27%)	1571 (0.22%)	1.20 [1.03, 1.41]
CKD 3+ (days 28-729)	69 (0.03%)	178 (0.02%)	1.36 [1.05, 1.77]
CKD 3+ (majority not returned to 90 and above) (days 28-729)	51 (0.02%)	136 (0.02%)	1.35 [0.95, 1.91]
CKD			
Composite outcome (days 28-179)	372 (13.66%)	787 (10.58%)	1.29 [1.18, 1.40]
Composite outcome (days 180-729)	474 (17.41%)	1109 (14.91%)	1.28 [1.19, 1.37]
eGFR decline of 50% or more (days 28-179)	82 (3.01%)	160 (2.15%)	1.23 [1.03, 1.47]
eGFR decline of 50% or more (days 180-729)	102 (3.75%)	211 (2.84%)	1.36 [1.17, 1.59]
eGFR decline of 40% or more (days 28-179)	212 (7.79%)	405 (5.44%)	1.32 [1.23, 1.41]
eGFR decline of 40% or more (days 180-729)	258 (9.47%)	556 (7.47%)	1.31 [1.10, 1.56]
eGFR decline of 30% or more (days 28-179)	364 (13.37%)	787 (10.58%)	1.25 [1.16, 1.35]
eGFR decline of 30% or more (days 180-729)	470 (17.26%)	1109 (14.91%)	1.26 [1.17, 1.36]
AKI			
Composite outcome (days 90-179)	107 (5.6%)	285 (3.63%)	1.38 [1.20, 1.58]
Composite outcome (days 180-729)	188 (9.84%)	439 (5.59%)	1.46 [1.27, 1.68]
eGFR decline of 50% or more (days 90-179)	50 (2.62%)	130 (1.66%)	1.46 [1.10, 1.93]
eGFR decline of 50% or more (days 180-729)	92 (4.82%)	189 (2.41%)	1.56 [1.26, 1.93]
eGFR decline of 40% or more (days 90-179)	69 (3.61%)	198 (2.52%)	1.26 [1.00, 1.60]
eGFR decline of 40% or more (days 180-729)	134 (7.02%)	302 (3.85%)	1.50 [1.28, 1.75]
eGFR decline of 30% or more (days 90-179)	105 (5.5%)	285 (3.63%)	1.35 [1.17, 1.55]
eGFR decline of 30% or more (days 180-729)	186 (9.74%)	439 (5.59%)	1.44 [1.24, 1.68]
CKD 2+ (days 28-729)	93 (4.87%)	262 (3.34%)	1.22 [1.04, 1.43]

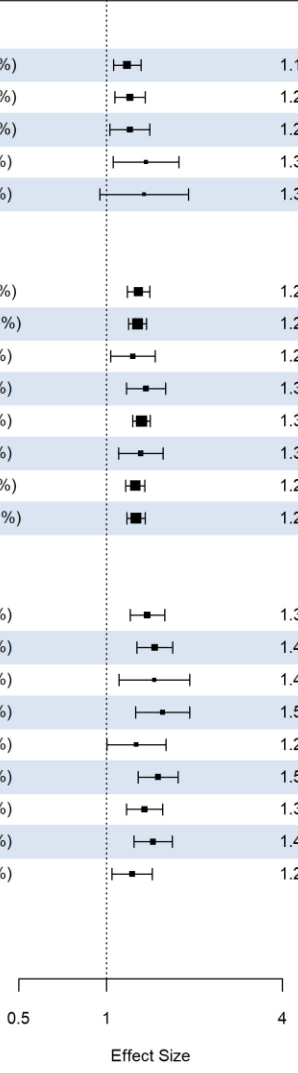

Supplement Table 12: Estimated hazard ratio in kidney function outcomes between the COVID-19 positive cohort and the control cohort for obese children and adolescents

Obesity: Obese	COVID-19 Groups Event (%)	Control Group Event (%)	HR (95% CI)
No CKD or AKI			
CKD 2+ (days 28-729)	764 (0.37%)	1609 (0.32%)	1.18 [1.07, 1.30]
CKD 2+ (majority not returned to 90 and above) (days 28-729)	646 (0.31%)	1334 (0.27%)	1.17 [1.04, 1.30]
CKD 2+ (not returned to 90 and above) (days 28-729)	604 (0.29%)	1183 (0.24%)	1.21 [1.03, 1.41]
CKD 3+ (days 28-729)	55 (0.03%)	113 (0.02%)	1.28 [0.94, 1.75]
CKD 3+ (majority not returned to 90 and above) (days 28-729)	42 (0.02%)	78 (0.02%)	1.41 [0.96, 2.06]
CKD 3+ (not returned to 90 and above) (days 28-729)	43 (0.02%)	94 (0.02%)	1.17 [0.77, 1.79]
CKD 3+ (not returned to 60 and above) (days 28-729)	37 (0.02%)	69 (0.01%)	1.36 [0.90, 2.05]
CKD			
Composite outcome (days 28-179)	533 (14.18%)	957 (12.69%)	1.10 [0.97, 1.24]
Composite outcome (days 180-729)	693 (18.44%)	1407 (18.66%)	1.06 [0.96, 1.16]
eGFR decline of 50% or more (days 28-179)	109 (2.9%)	245 (3.25%)	0.92 [0.78, 1.08]
eGFR decline of 50% or more (days 180-729)	144 (3.83%)	364 (4.83%)	0.95 [0.79, 1.14]
eGFR decline of 40% or more (days 28-179)	272 (7.24%)	552 (7.32%)	0.94 [0.79, 1.10]
eGFR decline of 40% or more (days 180-729)	357 (9.5%)	789 (10.46%)	1.01 [0.83, 1.24]
eGFR decline of 30% or more (days 28-179)	530 (14.1%)	957 (12.69%)	1.09 [0.97, 1.24]
eGFR decline of 30% or more (days 180-729)	689 (18.33%)	1407 (18.66%)	1.05 [0.95, 1.16]
AKI			
Composite outcome (days 90-179)	84 (5.36%)	171 (3.54%)	1.05 [0.77, 1.42]
Composite outcome (days 180-729)	171 (10.91%)	325 (6.72%)	1.14 [0.94, 1.38]
eGFR decline of 40% or more (days 90-179)	49 (3.13%)	100 (2.07%)	1.04 [0.63, 1.72]
eGFR decline of 40% or more (days 180-729)	116 (7.4%)	196 (4.05%)	1.26 [1.03, 1.54]
eGFR decline of 30% or more (days 90-179)	83 (5.3%)	171 (3.54%)	1.04 [0.76, 1.40]
eGFR decline of 30% or more (days 180-729)	169 (10.78%)	325 (6.72%)	1.12 [0.93, 1.34]
CKD 2+ (days 28-729)	90 (5.74%)	215 (4.45%)	1.09 [0.83, 1.44]
CKD 2+ (majority not returned to 90 and above) (days 28-729)	84 (5.36%)	197 (4.07%)	1.12 [0.83, 1.51]
CKD 2+ (not returned to 90 and above) (days 28-729)	76 (4.85%)	170 (3.52%)	1.21 [0.89, 1.64]

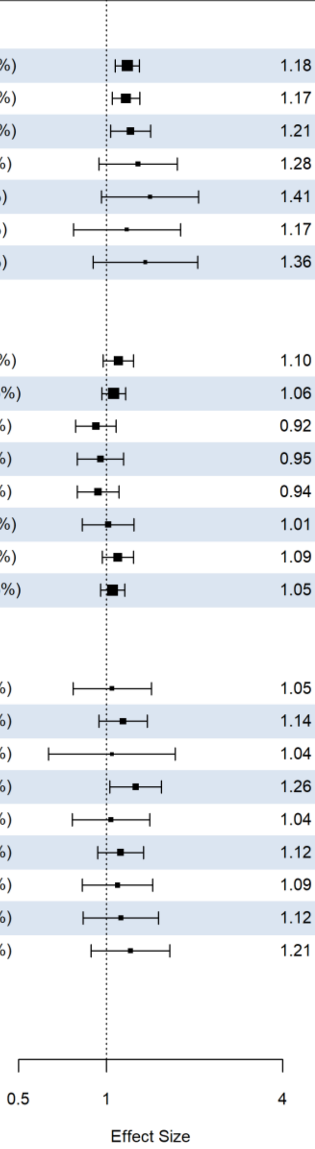

eAppendix 8. Sensitivity Analysis for Subgroups With Different Hospitalization Status

We conducted sensitivity analyses on both cohorts stratified by the hospitalization status (i.e, non-hospitalized, hospitalized, admitted to ICU). We performed the same PS stratification procedure and used Cox proportional hazard model to estimate the hazard ratio.

A. Empirical equipoise assessment

Supplement Figure 73: Preference score distributions of COVID-19 positive and negative groups for non-hospitalized children and adolescent with AKI. A greater convergence of these distributions indicates a higher similarity in the predicted likelihood of being infected between the COVID-19 positive (red) and negative (blue) participants.

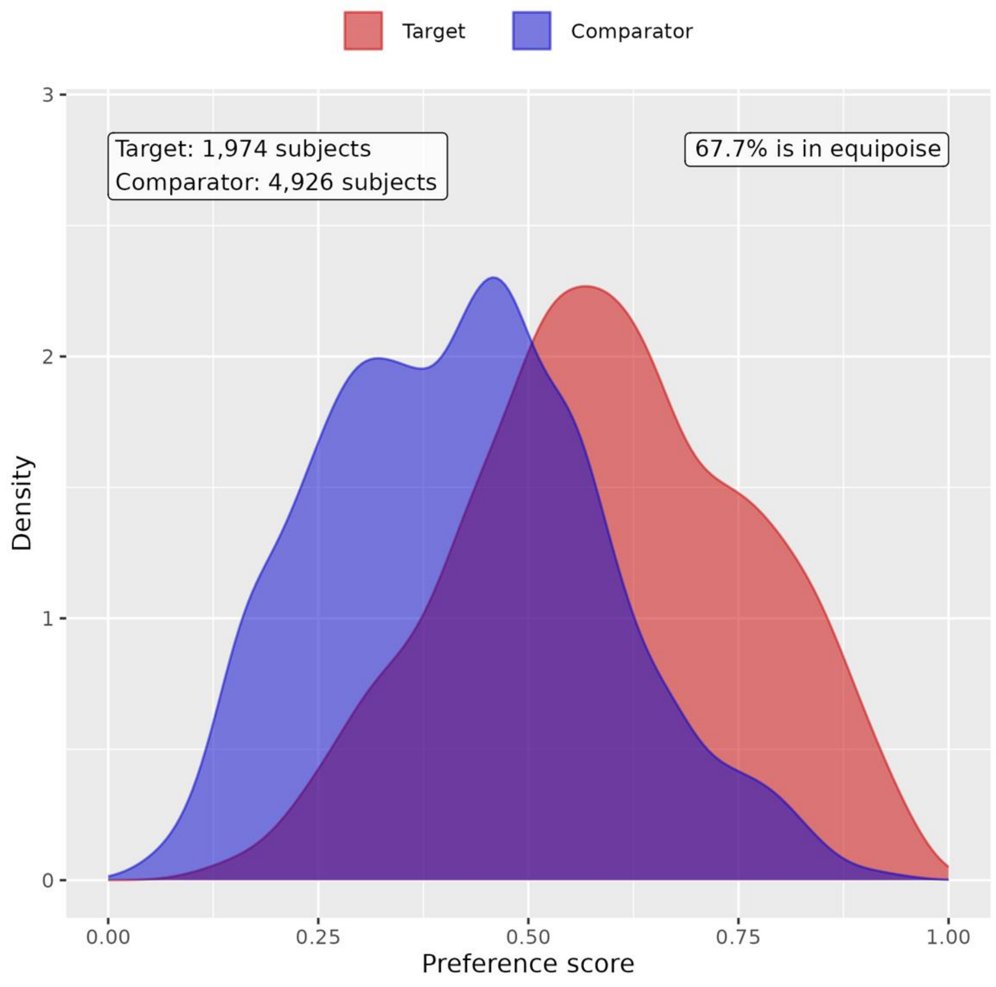

Supplement Figure 74: Preference score distributions of COVID-19 positive and negative groups for hospitalized children and adolescent with AKI. A greater convergence of these

distributions indicates a higher similarity in the predicted likelihood of being infected between the COVID-19 positive (red) and negative (blue) participants.

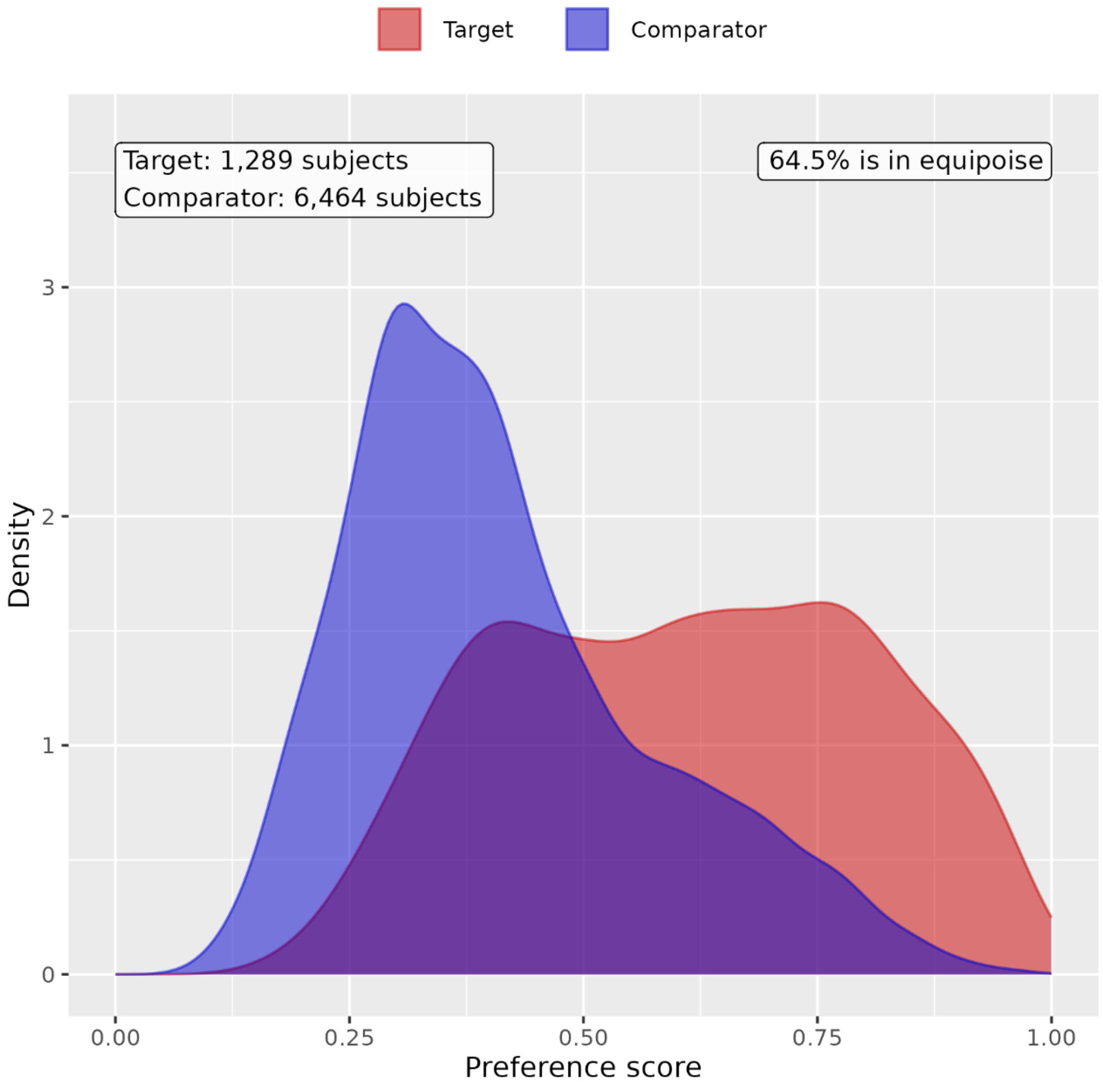

Supplement Figure 75: Preference score distributions of COVID-19 positive and negative groups for children and adolescent admitted to ICU with AKI. A greater convergence of these distributions indicates a higher similarity in the predicted likelihood of being infected between the COVID-19 positive (red) and negative (blue) participants.

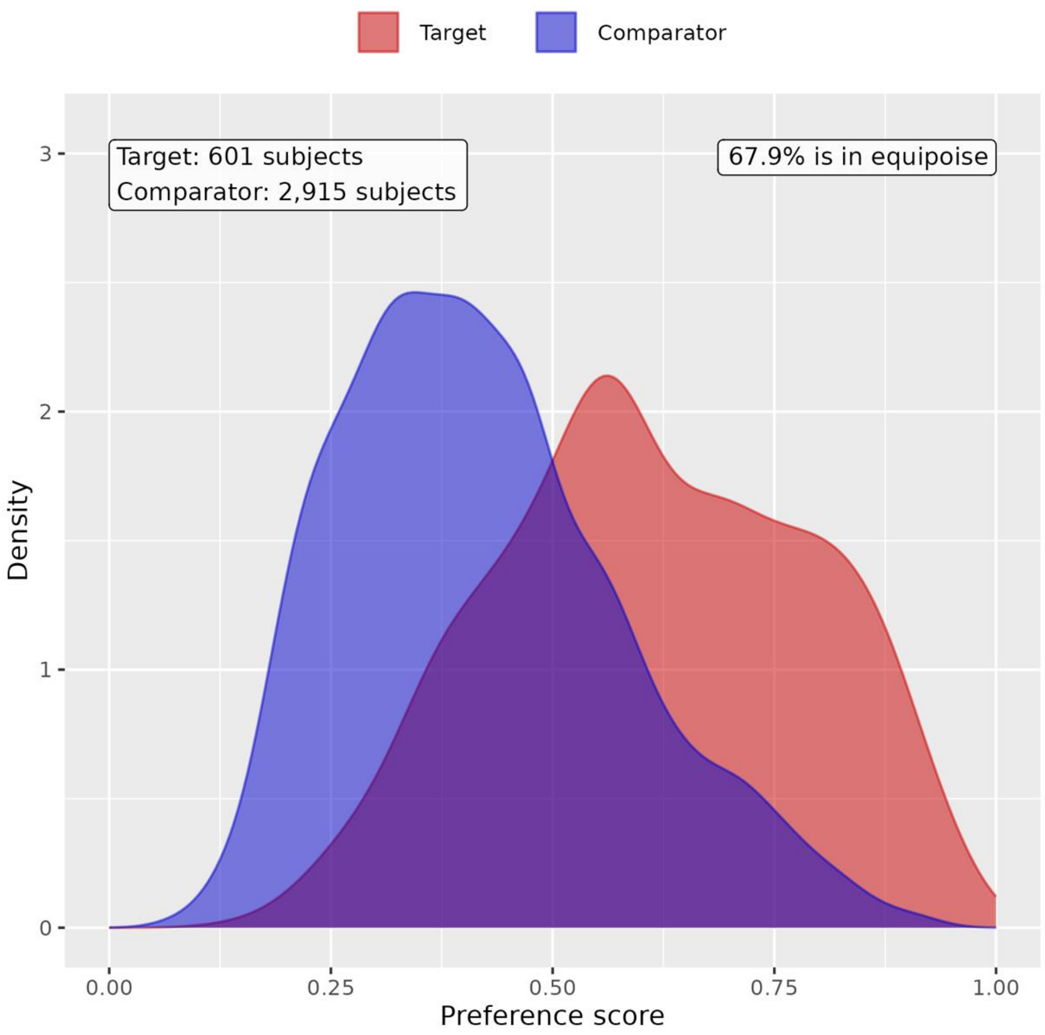

Supplement Figure 76: Preference score distributions of COVID-19 positive and negative groups for non-hospitalized children and adolescent with CKD. A greater convergence of these distributions indicates a higher similarity in the predicted likelihood of being infected between the COVID-19 positive (red) and negative (blue) participants.

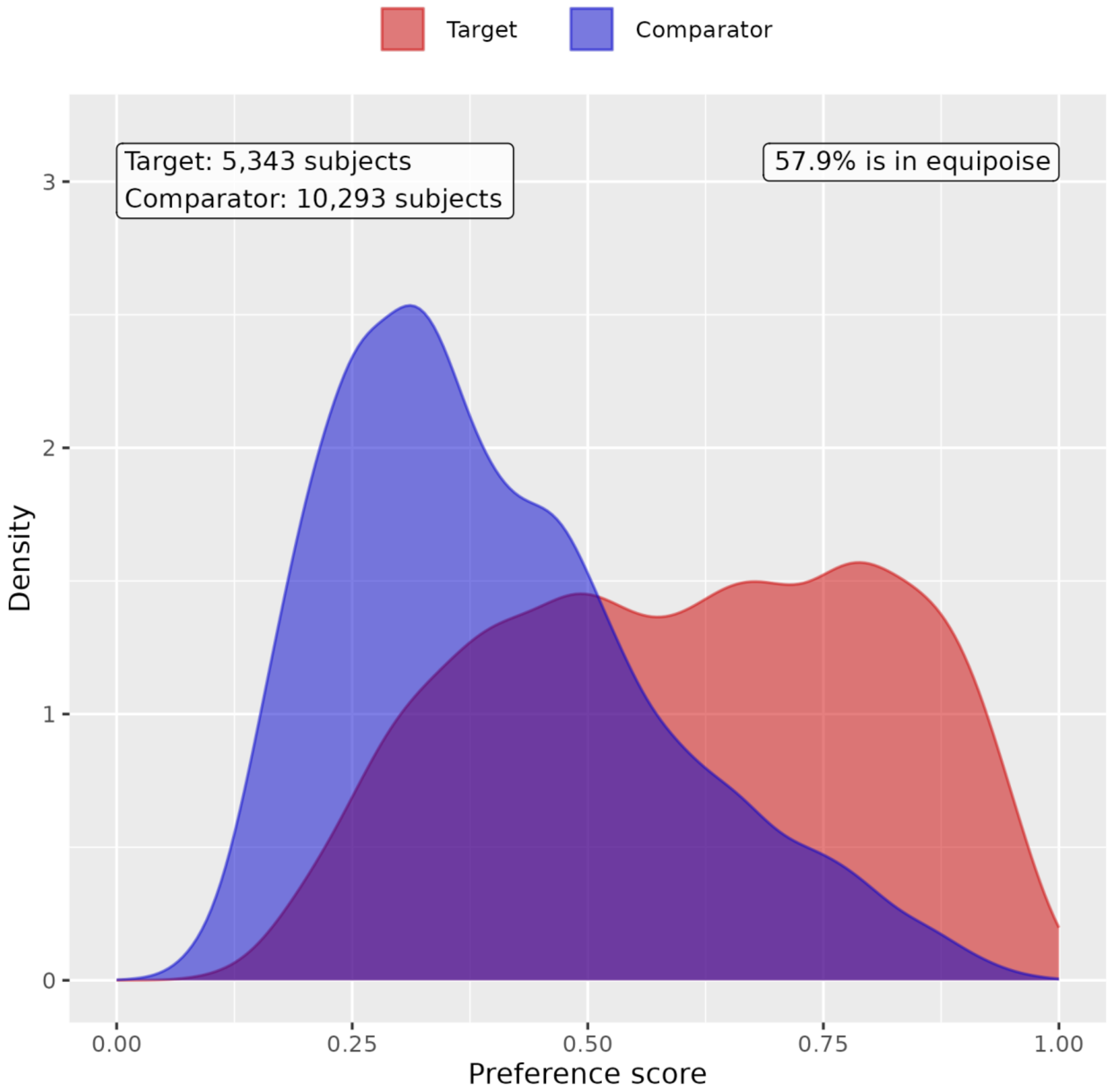

Supplement Figure 77: Preference score distributions of COVID-19 positive and negative groups for hospitalized children and adolescent with CKD. A greater convergence of these distributions indicates a higher similarity in the predicted likelihood of being infected between the COVID-19 positive (red) and negative (blue) participants.

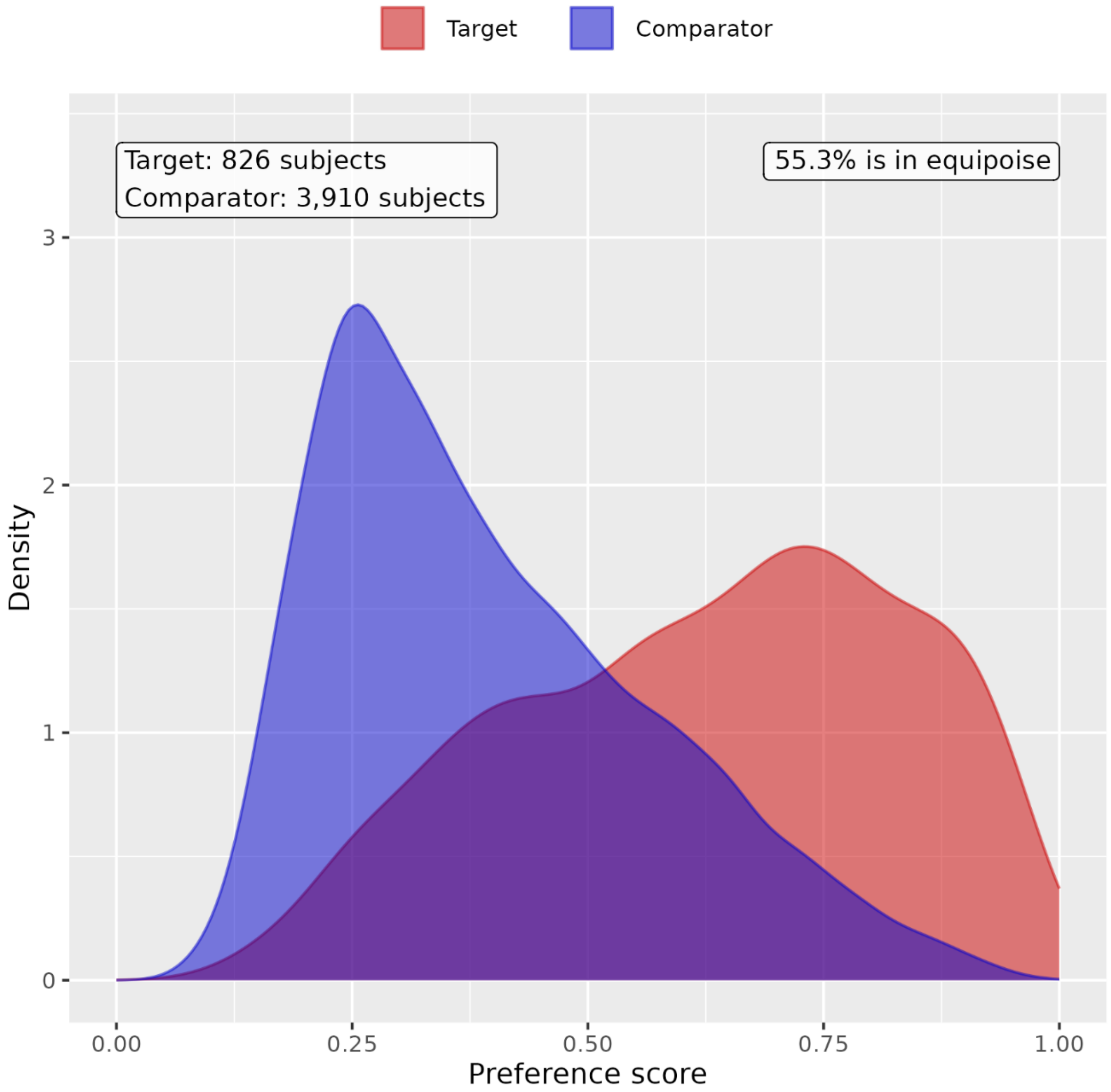

Supplement Figure 78: Preference score distributions of COVID-19 positive and control groups for children and adolescent with CKD admitted to ICU. A greater convergence of these distributions indicates a higher similarity in the predicted likelihood of being infected between the COVID-19 positive (red) and negative (blue) participants.

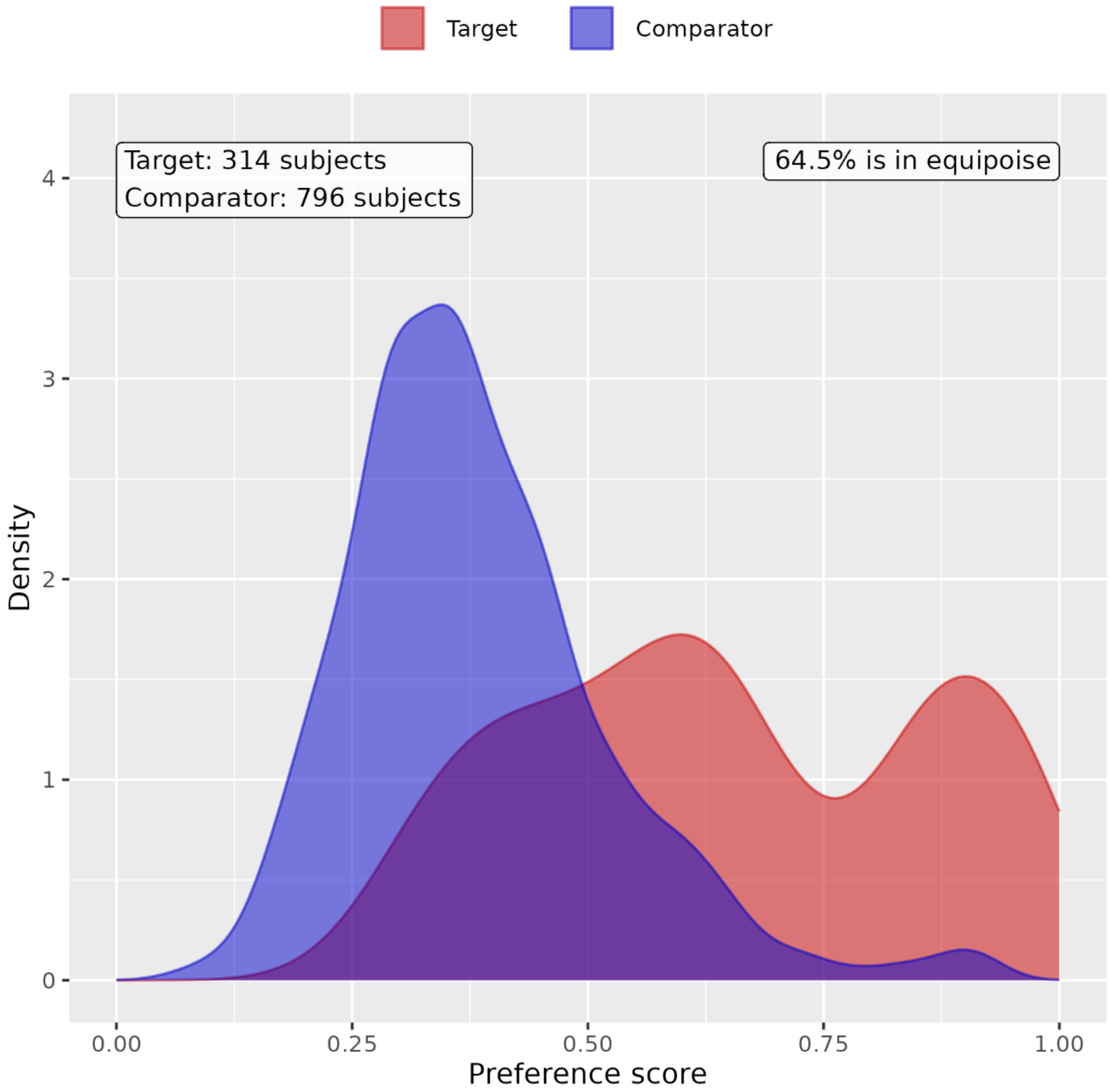

Supplement Figure 79: Preference score distributions of COVID-19 positive and negative groups for non-hospitalized children and adolescent with no AKI or CKD. A greater convergence of these distributions indicates a higher similarity in the predicted likelihood of being infected between the COVID-19 positive (red) and negative (blue) participants.

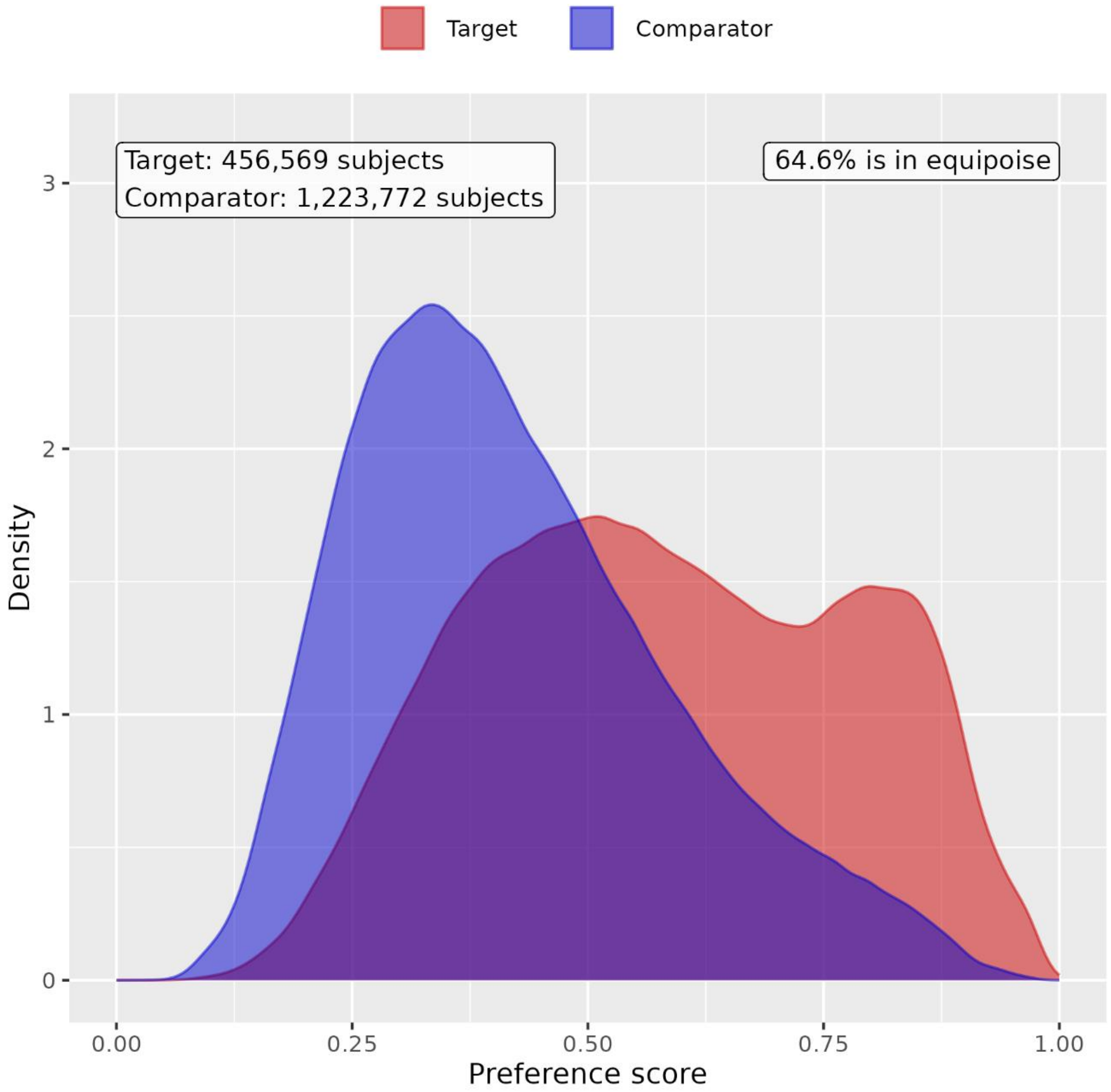

Supplement Figure 80: Preference score distributions of COVID-19 positive and negative groups for hospitalized children and adolescent with no AKI or CKD. A greater convergence of these distributions indicates a higher similarity in the predicted likelihood of being infected between the COVID-19 positive (red) and negative (blue) participants.

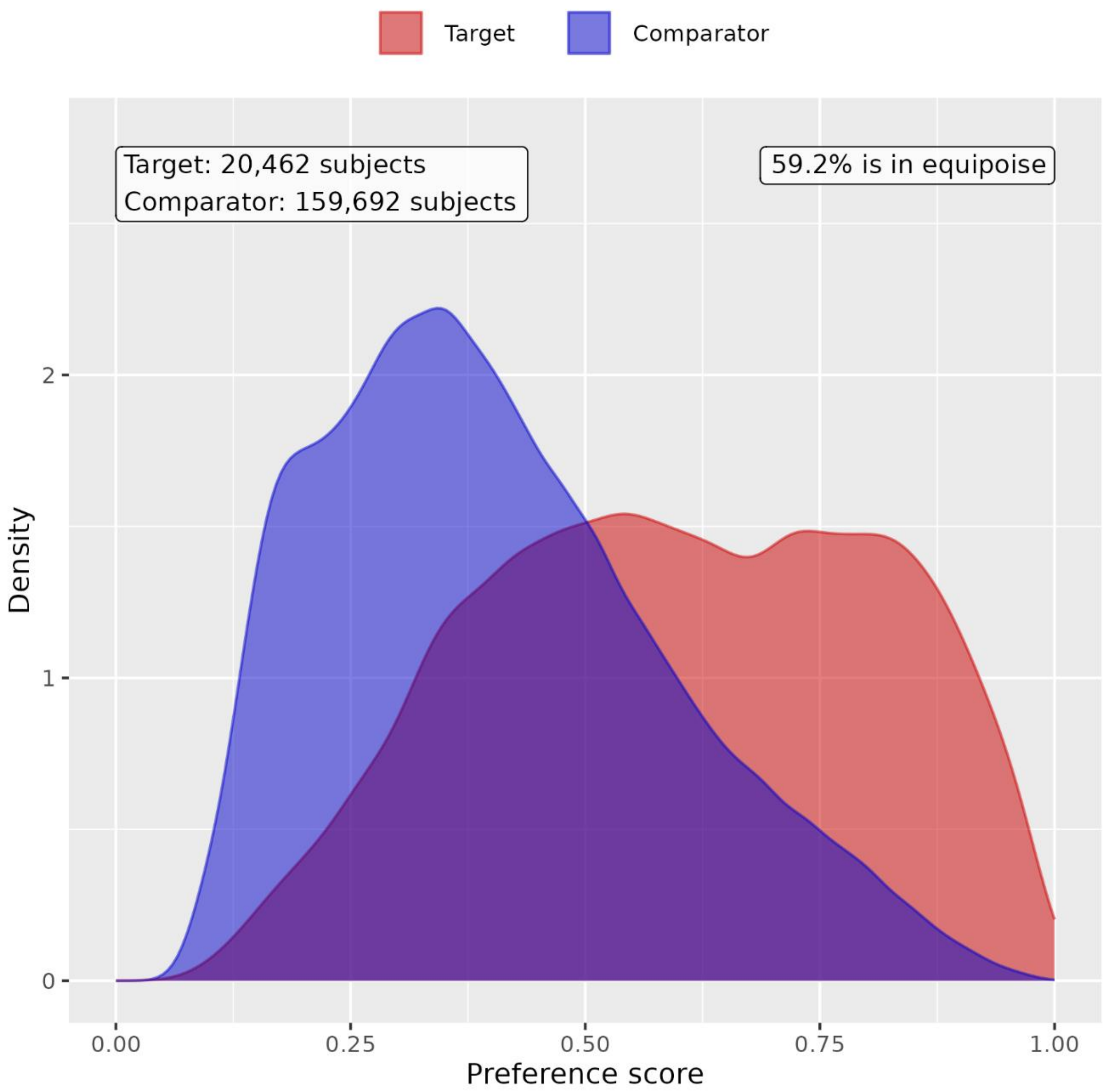

Supplement Figure 81: Preference score distributions of COVID-19 positive and control groups for children and adolescent with no AKI or CKD admitted to ICU. A greater convergence of these distributions indicates a higher similarity in the predicted likelihood of being infected between the COVID-19 positive (red) and negative (blue) participants.

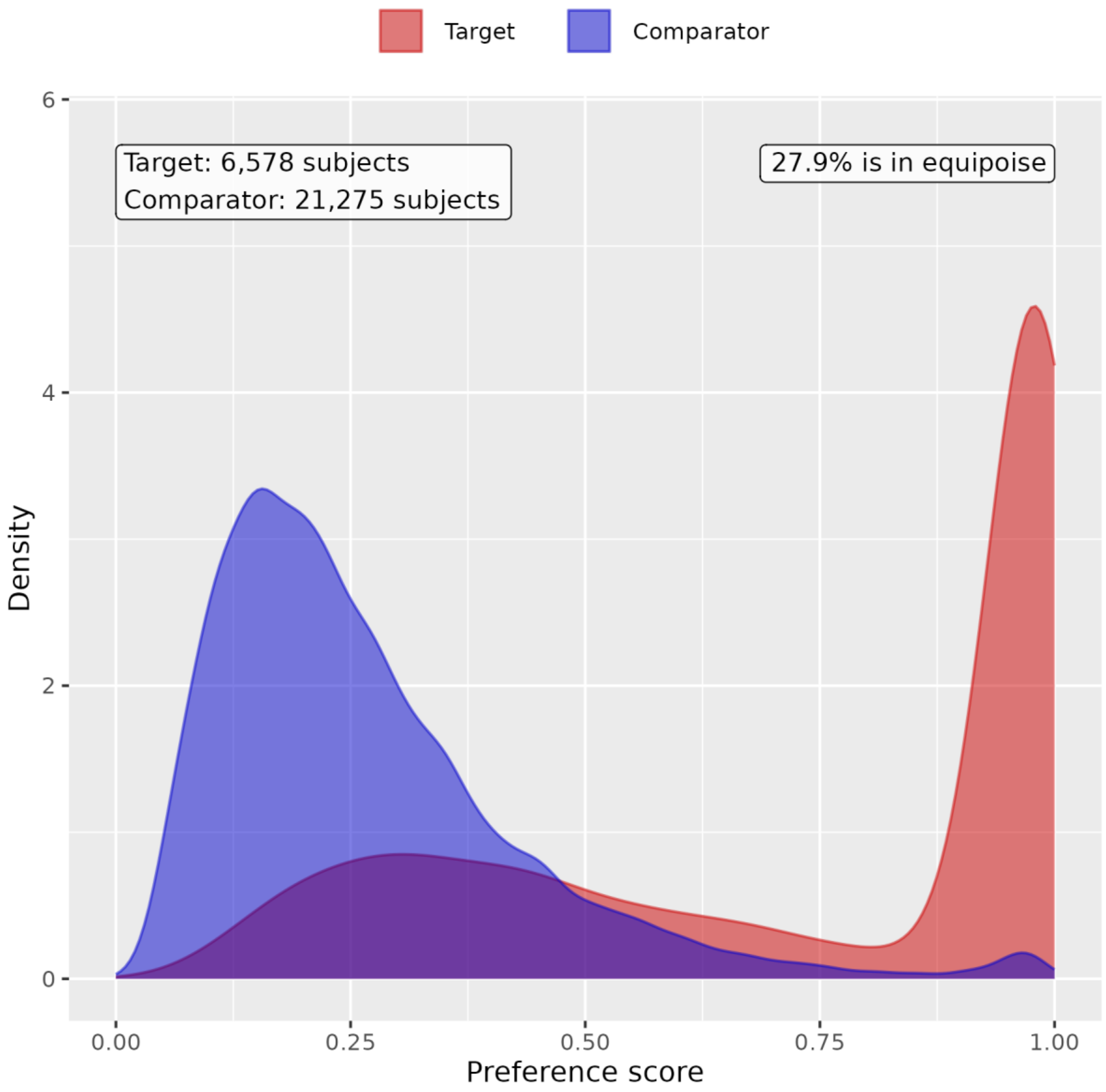

B. Patient characteristic balance

Supplement Figure 82: Patient characteristic balance before and after large-scale PS stratification with 6 strata for non-hospitalized children and adolescent with AKI. The upper panel displays the top 20 covariates with the largest standardized difference of means before stratification, while the lower panel displays the top 20 covariates with the largest standardized difference of means after stratification.

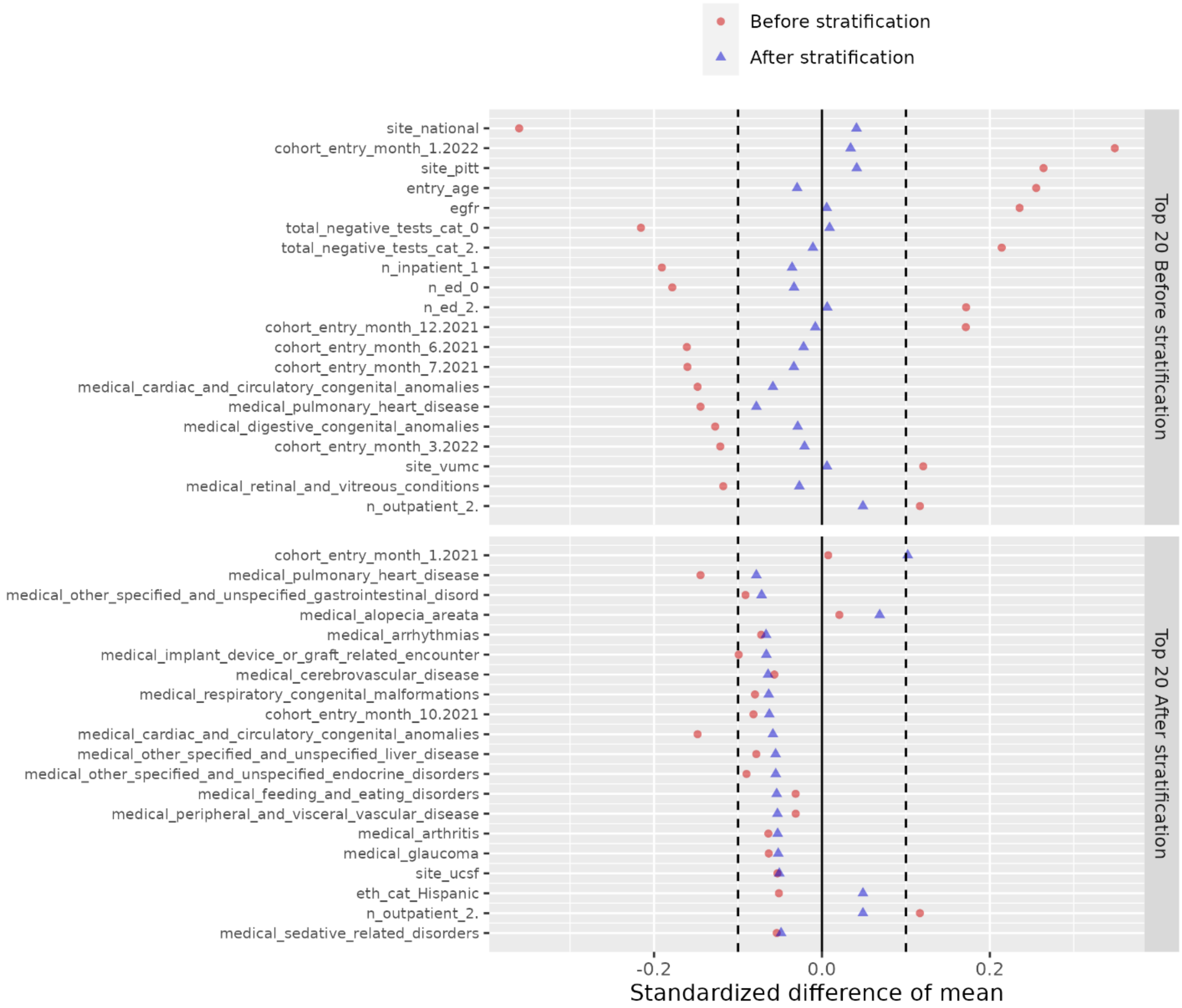

Supplement Figure 83: Patient characteristic balance before and after large-scale PS stratification with 6 strata for hospitalized children and adolescent with AKI. The upper panel displays the top 20 covariates with the largest standardized difference of means before stratification, while the lower panel displays the top 20 covariates with the largest standardized difference of means after stratification.

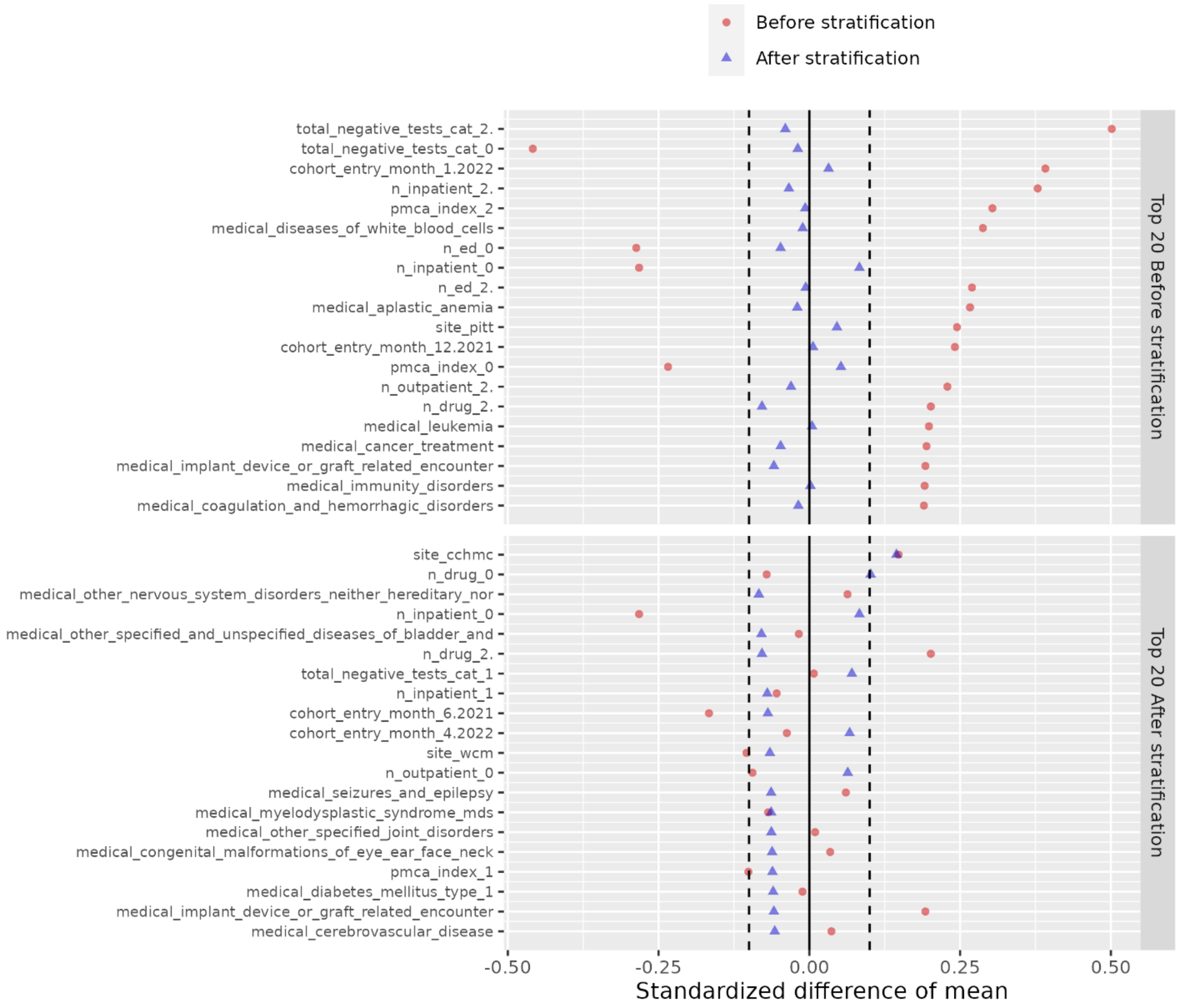

Supplement Figure 84: Patient characteristic balance before and after large-scale PS stratification with 6 strata for children and adolescent with AKI admitted to ICU. The upper panel displays the top 20 covariates with the largest standardized difference of means before stratification, while the lower panel displays the top 20 covariates with the largest standardized difference of means after stratification.

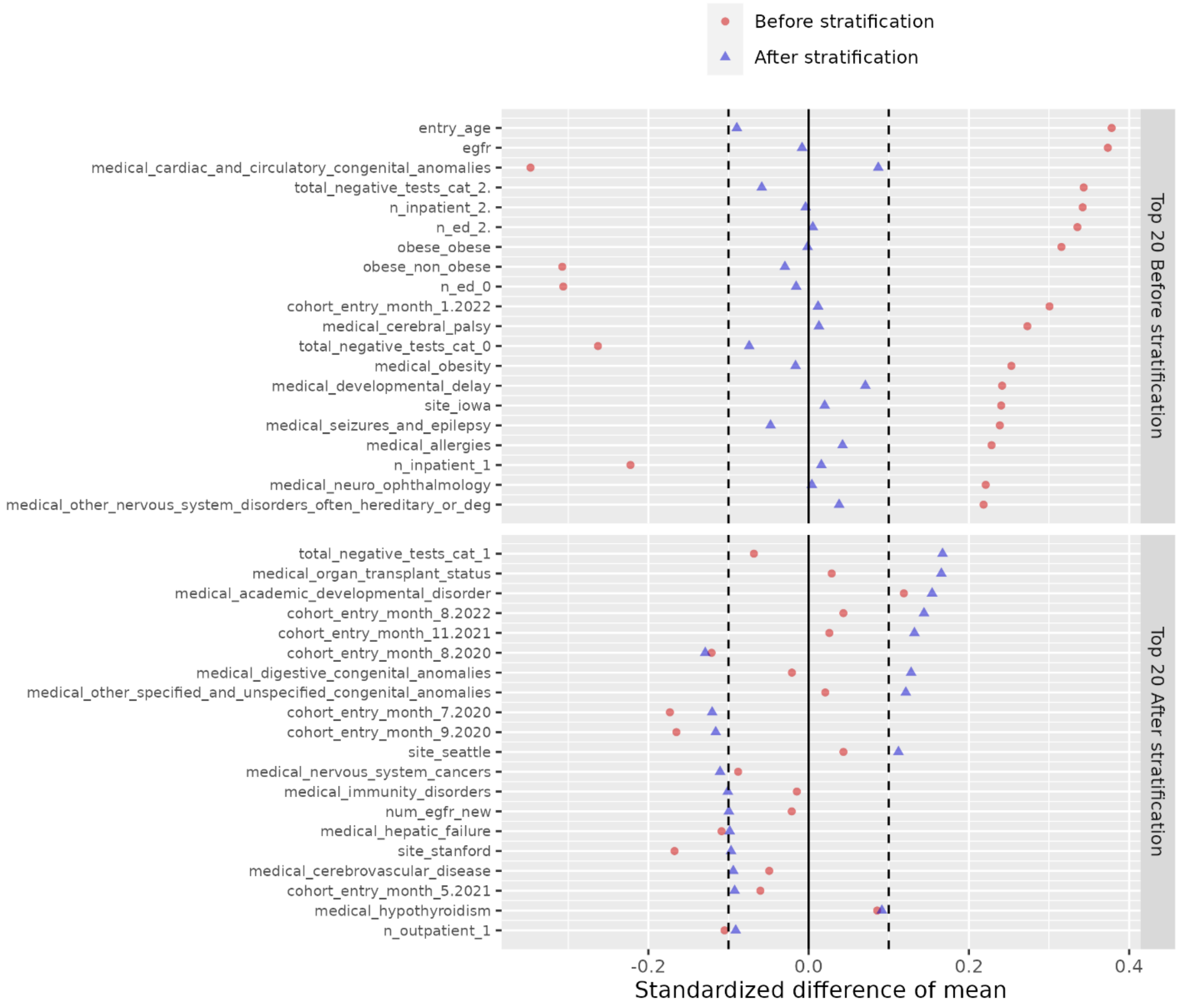

Supplement Figure 85: Patient characteristic balance before and after large-scale PS stratification with 6 strata for non-hospitalized children and adolescent with CKD. The upper panel displays the top 20 covariates with the largest standardized difference of means before stratification, while the lower panel displays the top 20 covariates with the largest standardized difference of means after stratification.

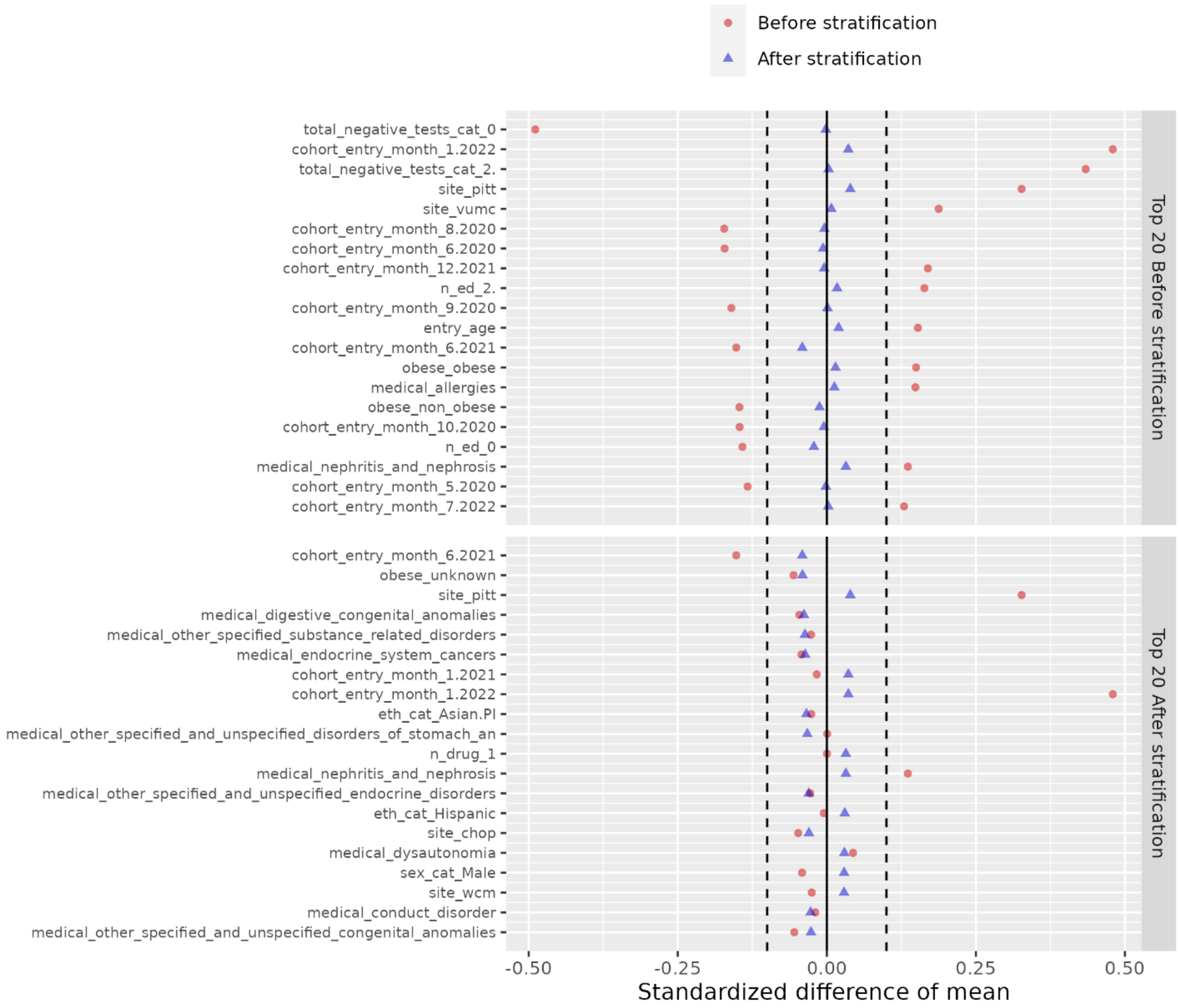

Supplement Figure 86: Patient characteristic balance before and after large-scale PS stratification with 6 strata for hospitalized children and adolescent with CKD. The upper panel displays the top 20 covariates with the largest standardized difference of means before stratification, while the lower panel displays the top 20 covariates with the largest standardized difference of means after stratification.

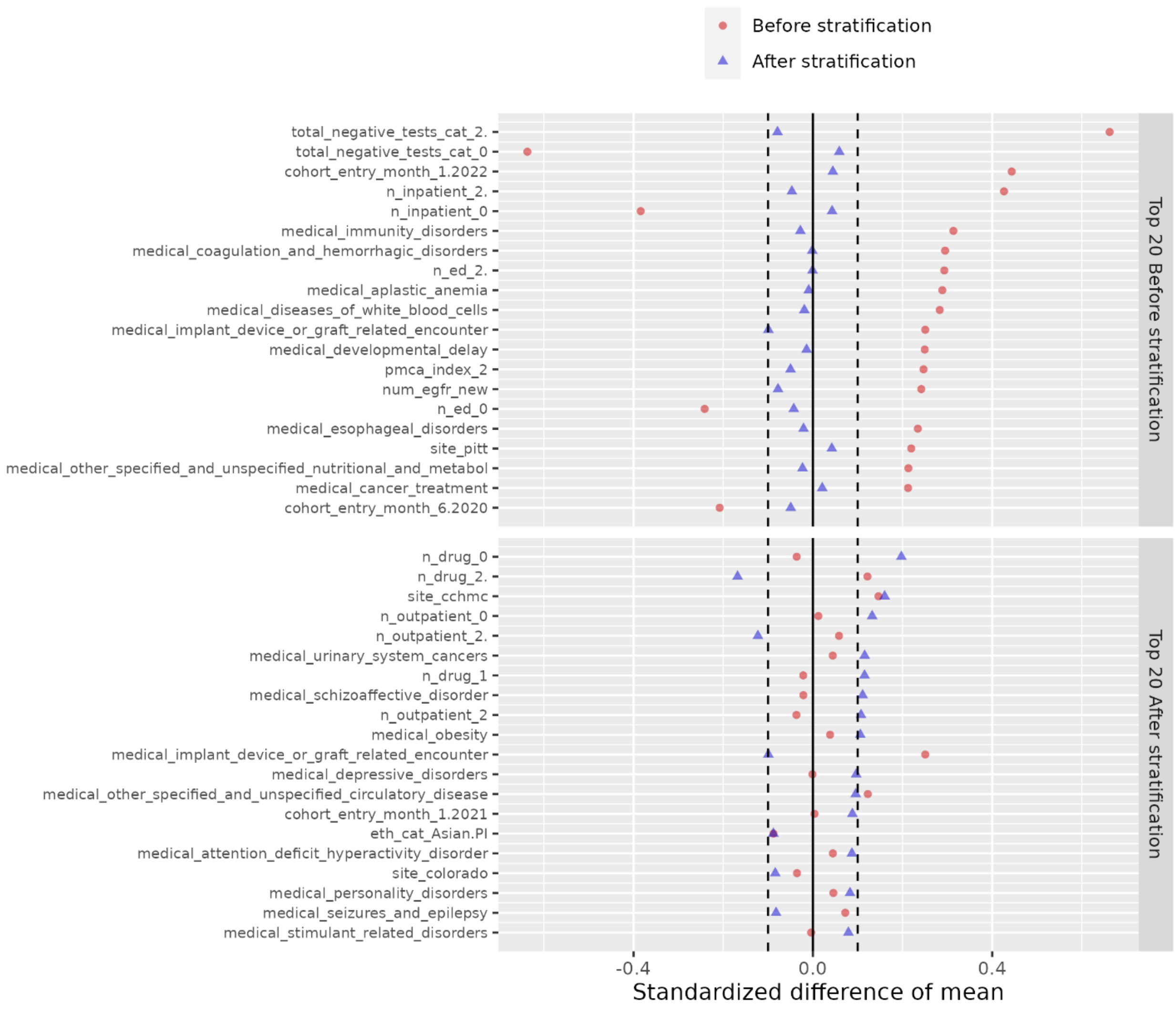

Supplement Figure 87: Patient characteristic balance before and after large-scale PS stratification with 6 strata for children and adolescent with CKD admitted to ICU. The upper panel displays the top 20 covariates with the largest standardized difference of means before stratification, while the lower panel displays the top 20 covariates with the largest standardized difference of means after stratification.

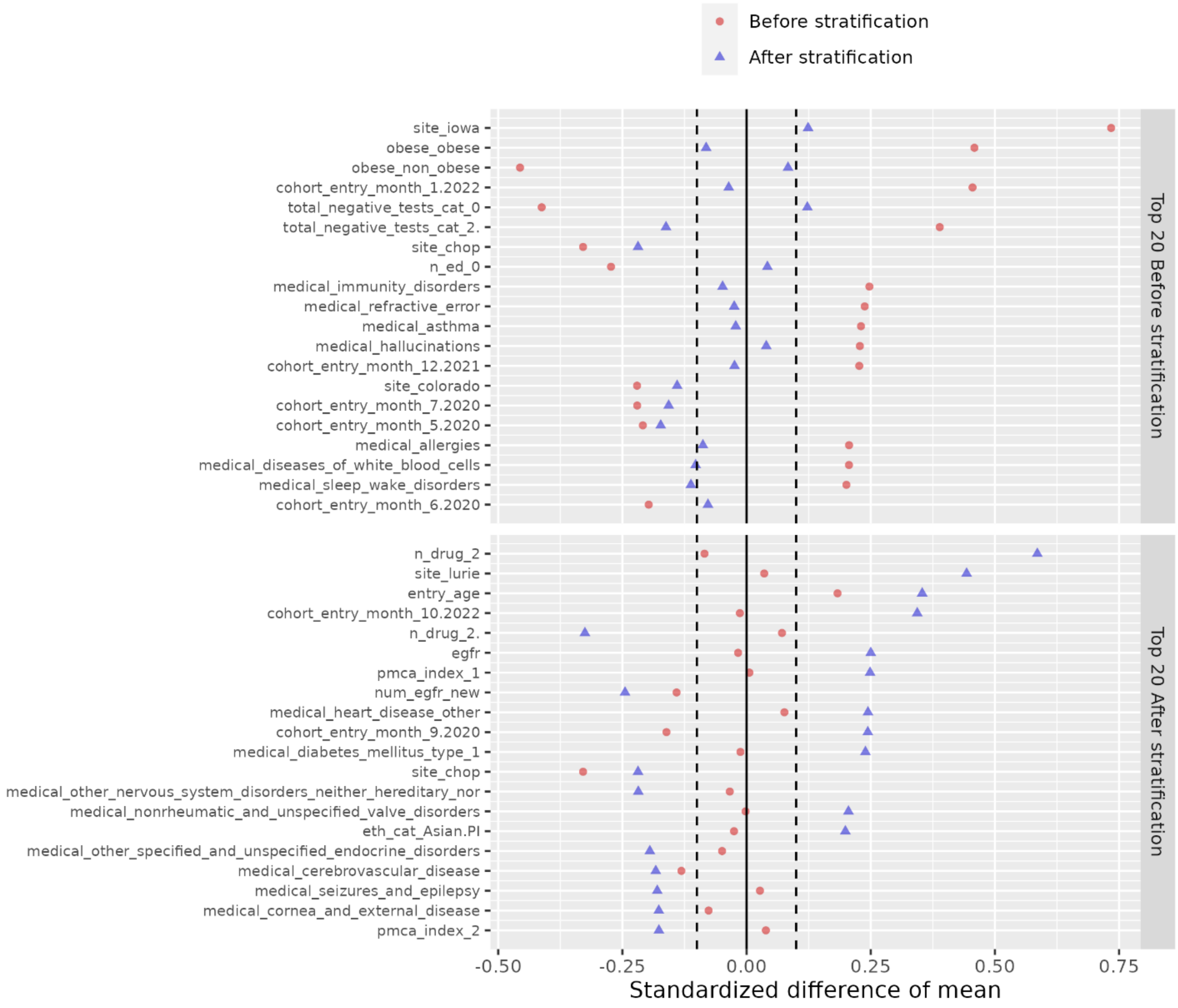

Supplement Figure 88: Patient characteristic balance before and after large-scale PS stratification with 6 strata for non-hospitalized children and adolescent with no AKI or CKD. The upper panel displays the top 20 covariates with the largest standardized difference of means before stratification, while the lower panel displays the top 20 covariates with the largest standardized difference of means after stratification.

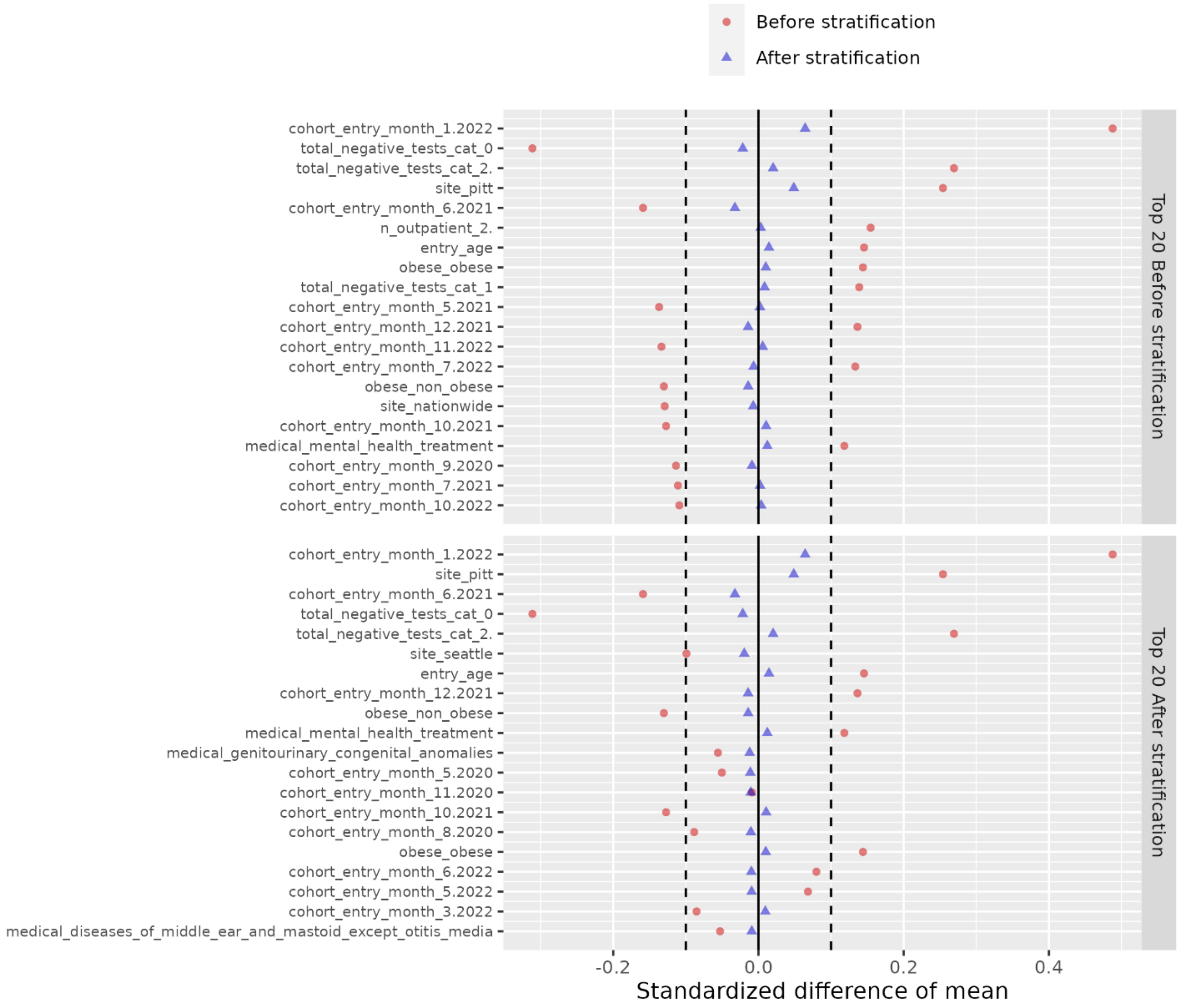

Supplement Figure 89: Patient characteristic balance before and after large-scale PS stratification with 6 strata for hospitalized children and adolescent with no AKI or CKD. The upper panel displays the top 20 covariates with the largest standardized difference of means before stratification, while the lower panel displays the top 20 covariates with the largest standardized difference of means after stratification.

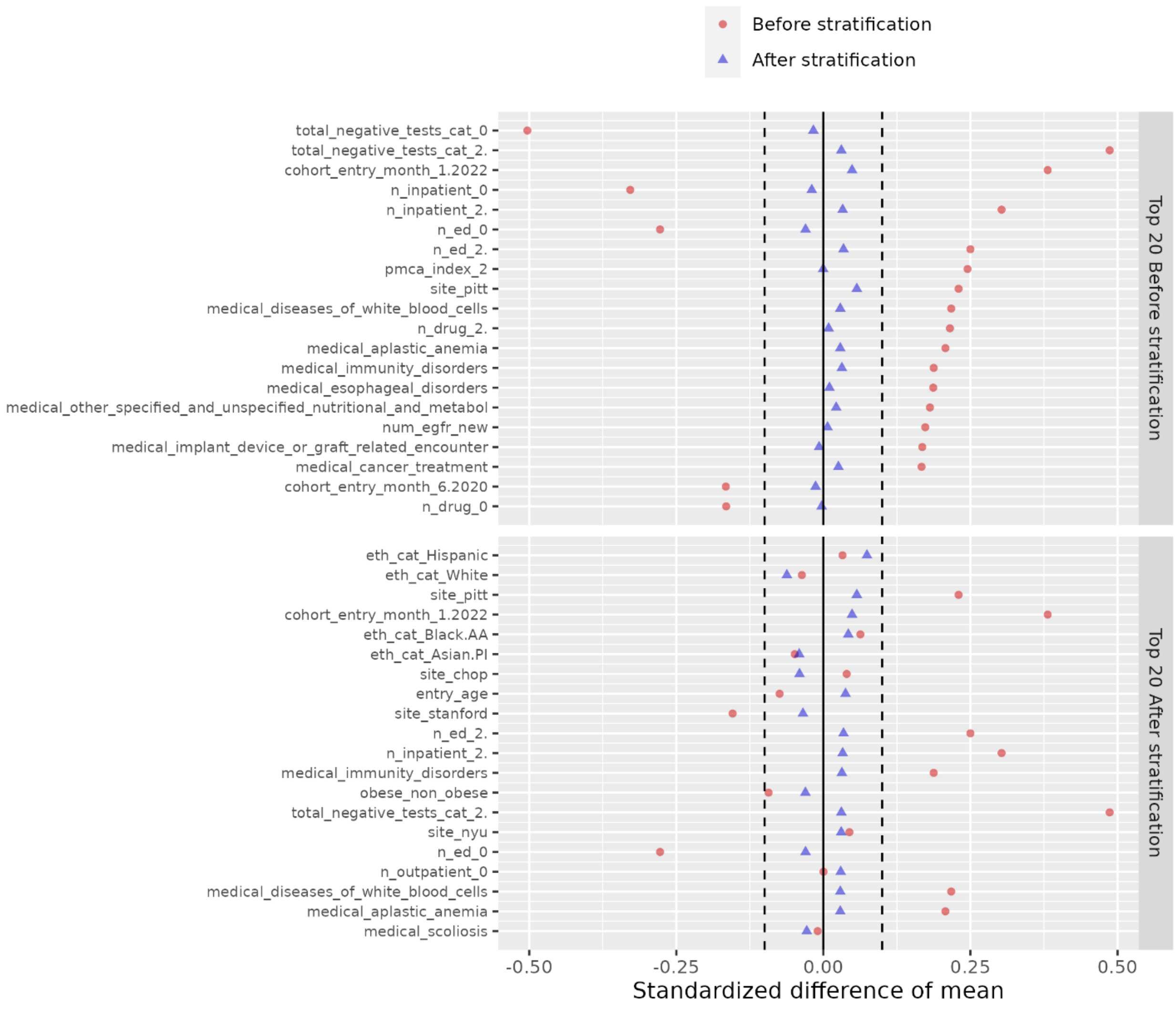

Supplement Figure 90: Patient characteristic balance before and after large-scale PS stratification with 6 strata for children and adolescents with no AKI or CKD admitted to ICU. The upper panel displays the top 20 covariates with the largest standardized difference of means before stratification, while the lower panel displays the top 20 covariates with the largest standardized difference of means after stratification.

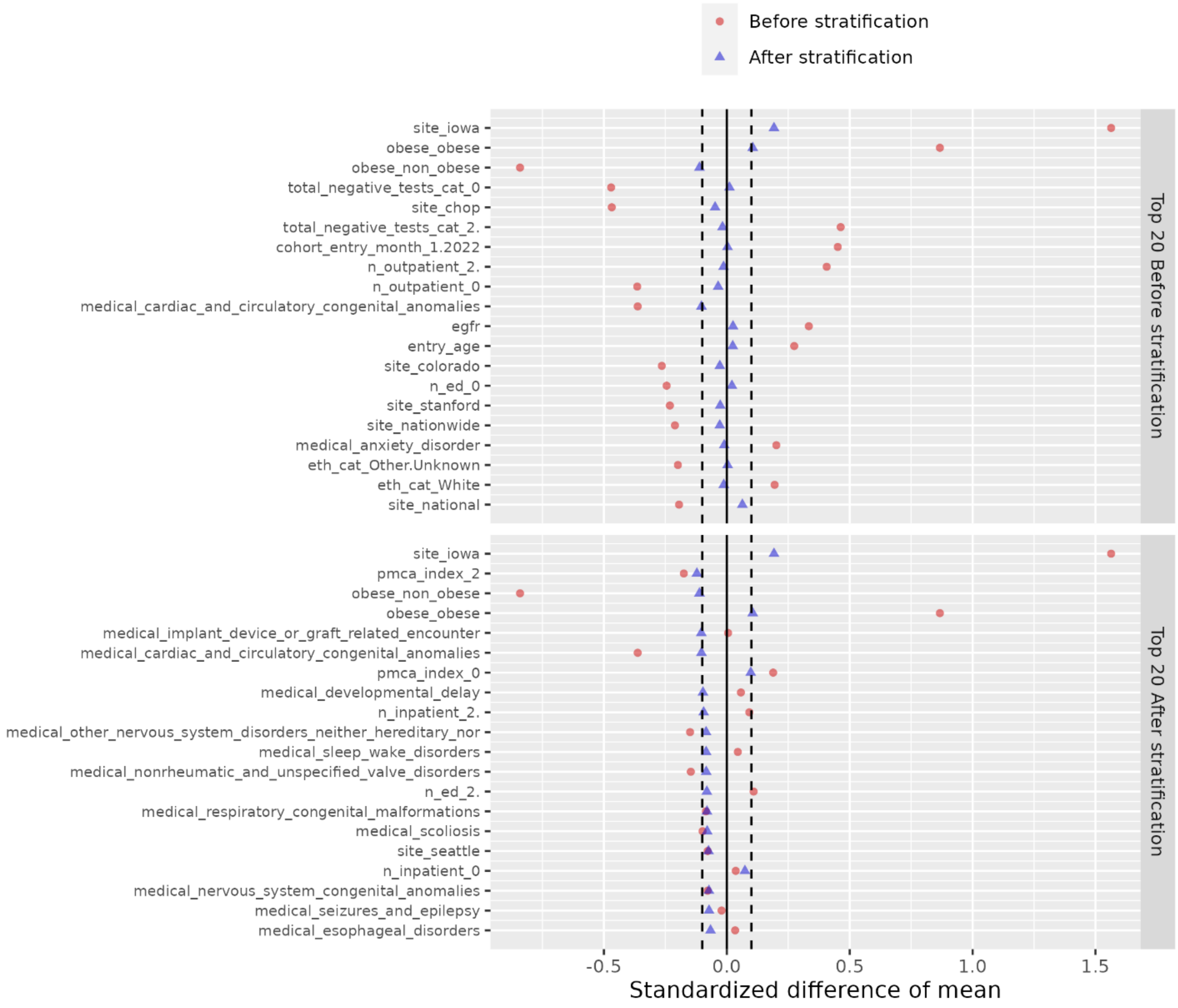

C. Hazard ratio of COVID-19 positive group compared to control group

Supplement Table 13: Estimated hazard ratio in kidney function outcomes between the COVID-19 positive cohort and the control cohort for non-hospitalized children and adolescents.

Hospitalization: nonhospitalized	COVID-19 Groups Event (%)	Control Group Event (%)	HR (95% CI)
No CKD or AKI			
CKD 2+ (days 28-729)	1283 (0.28%)	2774 (0.23%)	1.19 [1.12, 1.26]
CKD 2+ (majority not returned to 90 and above) (days 28-729)	1136 (0.25%)	2363 (0.19%)	1.22 [1.13, 1.31]
CKD 2+ (not returned to 90 and above) (days 28-729)	1075 (0.24%)	2134 (0.17%)	1.24 [1.15, 1.35]
CKD 3+ (days 28-729)	83 (0.02%)	165 (0.01%)	1.41 [1.16, 1.71]
CKD 3+ (majority not returned to 90 and above) (days 28-729)	65 (0.01%)	122 (0.01%)	1.48 [1.13, 1.95]
CKD 3+ (not returned to 90 and above) (days 28-729)	75 (0.02%)	146 (0.01%)	1.39 [1.07, 1.82]
CKD 3+ (not returned to 60 and above) (days 28-729)	63 (0.01%)	109 (0.01%)	1.52 [1.15, 2.01]
CKD			
Composite outcome (days 28-179)	668 (12.5%)	1058 (10.28%)	1.14 [1.04, 1.26]
Composite outcome (days 180-729)	910 (17.03%)	1670 (16.22%)	1.11 [1.04, 1.19]
eGFR decline of 50% or more (days 28-179)	120 (2.25%)	182 (1.77%)	1.13 [0.76, 1.68]
eGFR decline of 50% or more (days 180-729)	167 (3.13%)	283 (2.75%)	1.16 [0.92, 1.46]
eGFR decline of 40% or more (days 28-179)	332 (6.21%)	508 (4.94%)	1.05 [0.89, 1.24]
eGFR decline of 40% or more (days 180-729)	457 (8.55%)	780 (7.58%)	1.14 [1.06, 1.23]
eGFR decline of 30% or more (days 28-179)	661 (12.37%)	1058 (10.28%)	1.13 [1.02, 1.24]
eGFR decline of 30% or more (days 180-729)	908 (16.99%)	1670 (16.22%)	1.11 [1.04, 1.19]
AKI			
Composite outcome (days 90-179)	86 (4.36%)	149 (3.02%)	1.21 [0.85, 1.73]
Composite outcome (days 180-729)	172 (8.71%)	303 (6.15%)	1.12 [0.87, 1.44]
eGFR decline of 50% or more (days 180-729)	62 (3.14%)	83 (1.68%)	1.54 [1.28, 1.85]
eGFR decline of 40% or more (days 180-729)	103 (5.22%)	166 (3.37%)	1.22 [0.98, 1.51]
eGFR decline of 30% or more (days 90-179)	86 (4.36%)	149 (3.02%)	1.21 [0.85, 1.73]
eGFR decline of 30% or more (days 180-729)	169 (8.56%)	303 (6.15%)	1.09 [0.86, 1.40]
CKD 2+ (days 28-729)	116 (5.88%)	226 (4.59%)	1.08 [0.90, 1.31]
CKD 2+ (majority not returned to 90 and above) (days 28-729)	104 (5.27%)	200 (4.06%)	1.07 [0.88, 1.30]
CKD 2+ (not returned to 90 and above) (days 28-729)	94 (4.76%)	186 (3.78%)	1.02 [0.86, 1.21]

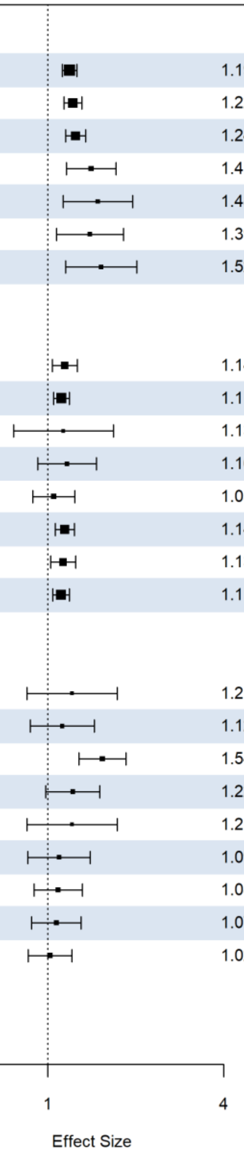

Supplement Table 14: Estimated hazard ratio in kidney function outcomes between the COVID-19 positive cohort and the control cohort for hospitalized children and adolescents.

Hospitalization: hospitalized	COVID-19 Groups Event (%)	Control Group Event (%)	HR (95% CI)
No CKD or AKI			
CKD 2+ (days 28-729)	286 (1.4%)	1126 (0.71%)	1.64 [1.35, 2.00]
CKD 2+ (majority not returned to 90 and above) (days 28-729)	218 (1.07%)	902 (0.56%)	1.59 [1.24, 2.05]
CKD 2+ (not returned to 90 and above) (days 28-729)	175 (0.86%)	775 (0.49%)	1.54 [1.21, 1.95]
CKD 3+ (days 28-729)	49 (0.24%)	144 (0.09%)	1.90 [1.51, 2.39]
CKD 3+ (majority not returned to 90 and above) (days 28-729)	33 (0.16%)	102 (0.06%)	1.92 [1.41, 2.62]
CKD 3+ (not returned to 90 and above) (days 28-729)	36 (0.18%)	125 (0.08%)	1.65 [1.20, 2.27]
CKD 3+ (not returned to 60 and above) (days 28-729)	25 (0.12%)	91 (0.06%)	1.69 [1.04, 2.76]
CKD			
Composite outcome (days 28-179)	164 (19.85%)	558 (14.27%)	1.34 [1.04, 1.73]
Composite outcome (days 180-729)	186 (22.52%)	707 (18.08%)	1.34 [1.24, 1.45]
eGFR decline of 50% or more (days 180-729)	61 (7.38%)	238 (6.09%)	1.19 [0.83, 1.71]
eGFR decline of 40% or more (days 28-179)	101 (12.23%)	361 (9.23%)	1.16 [0.85, 1.58]
eGFR decline of 40% or more (days 180-729)	122 (14.77%)	464 (11.87%)	1.31 [1.09, 1.58]
eGFR decline of 30% or more (days 28-179)	161 (19.49%)	558 (14.27%)	1.30 [1.00, 1.69]
eGFR decline of 30% or more (days 180-729)	183 (22.15%)	707 (18.08%)	1.31 [1.20, 1.44]
AKI			
Composite outcome (days 180-729)	133 (10.32%)	361 (5.58%)	1.41 [1.02, 1.94]
eGFR decline of 40% or more (days 180-729)	104 (8.07%)	259 (4.01%)	1.50 [1.06, 2.11]
eGFR decline of 30% or more (days 180-729)	131 (10.16%)	361 (5.58%)	1.38 [1.03, 1.85]

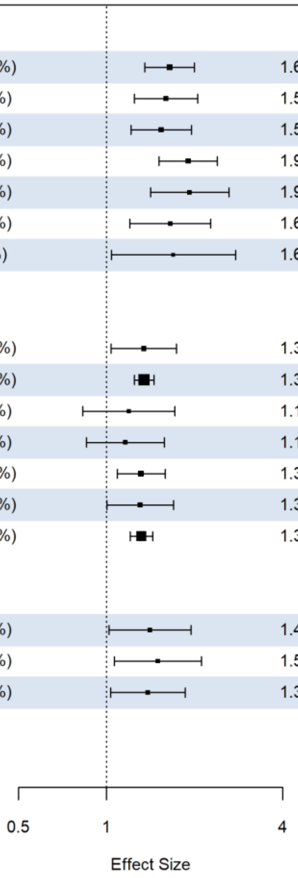

Supplement Table 15: Estimated hazard ratio in kidney function outcomes between the COVID-19 positive cohort and the control cohort for children and adolescents admitted to ICU.

Hospitalization: ICU	COVID-19 Groups Event (%)	Control Group Event (%)	HR (95% CI)
No CKD or AKI			
CKD 2+ (days 28-729)	100 (1.52%)	214 (1.01%)	2.38 [1.55, 3.65]
CKD 2+ (majority not returned to 90 and above) (days 28-729)	79 (1.2%)	169 (0.79%)	2.33 [1.45, 3.74]
AKI			
Composite outcome (days 90-179)	39 (6.49%)	98 (3.36%)	1.56 [0.96, 2.55]
Composite outcome (days 180-729)	65 (10.82%)	132 (4.53%)	1.97 [1.31, 2.96]
eGFR decline of 50% or more (days 90-179)	17 (2.83%)	38 (1.3%)	2.08 [1.00, 4.31]
eGFR decline of 50% or more (days 180-729)	27 (4.49%)	65 (2.23%)	1.32 [0.90, 1.94]
eGFR decline of 40% or more (days 90-179)	26 (4.33%)	67 (2.3%)	1.61 [0.80, 3.24]
eGFR decline of 40% or more (days 180-729)	49 (8.15%)	91 (3.12%)	2.01 [1.27, 3.18]
eGFR decline of 30% or more (days 90-179)	38 (6.32%)	98 (3.36%)	1.52 [0.95, 2.42]
eGFR decline of 30% or more (days 180-729)	65 (10.82%)	132 (4.53%)	1.97 [1.31, 2.96]

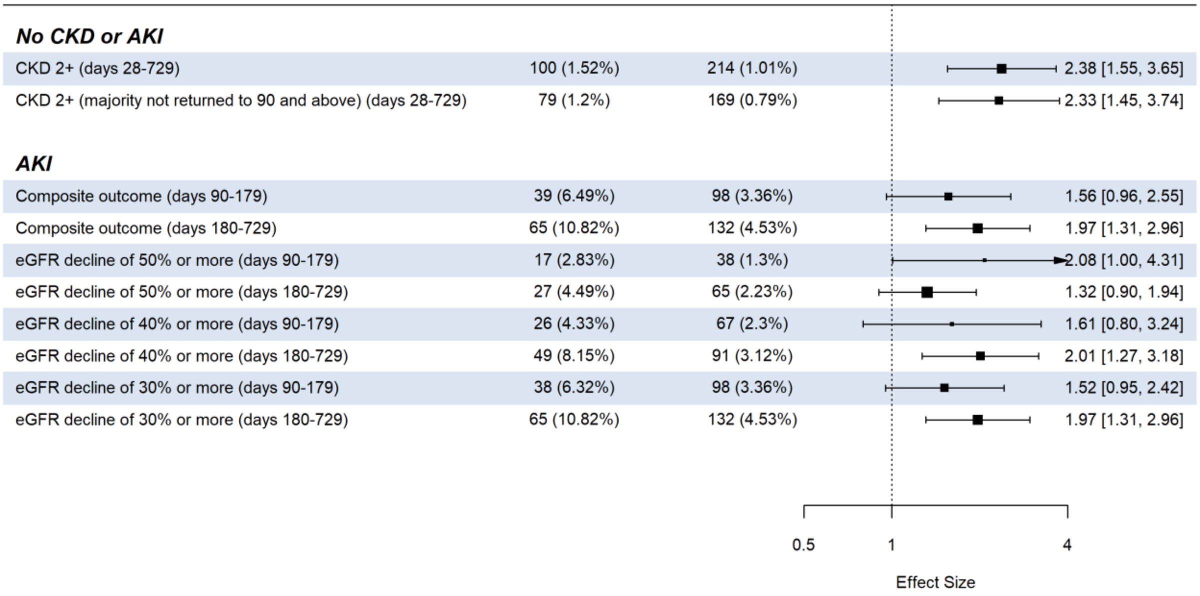

eAppendix 9. Sensitivity Analysis for Subgroups With Different Levels of COVID-19 Severity

We conducted sensitivity analyses on both cohorts stratified by the COVID-19 severity (i.e., asymptomatic, mild, moderate, and severe). We performed the same PS stratification procedure and used Cox proportional hazard model to estimate the hazard ratio.

A. Empirical equipoise assessment

Supplement Figure 91: Preference score distributions of COVID-19 positive and negative groups for children and adolescents with AKI in asymptomatic group. A greater convergence of these distributions indicates a higher similarity in the predicted likelihood of being infected between the COVID-19 positive (red) and negative (blue) participants.

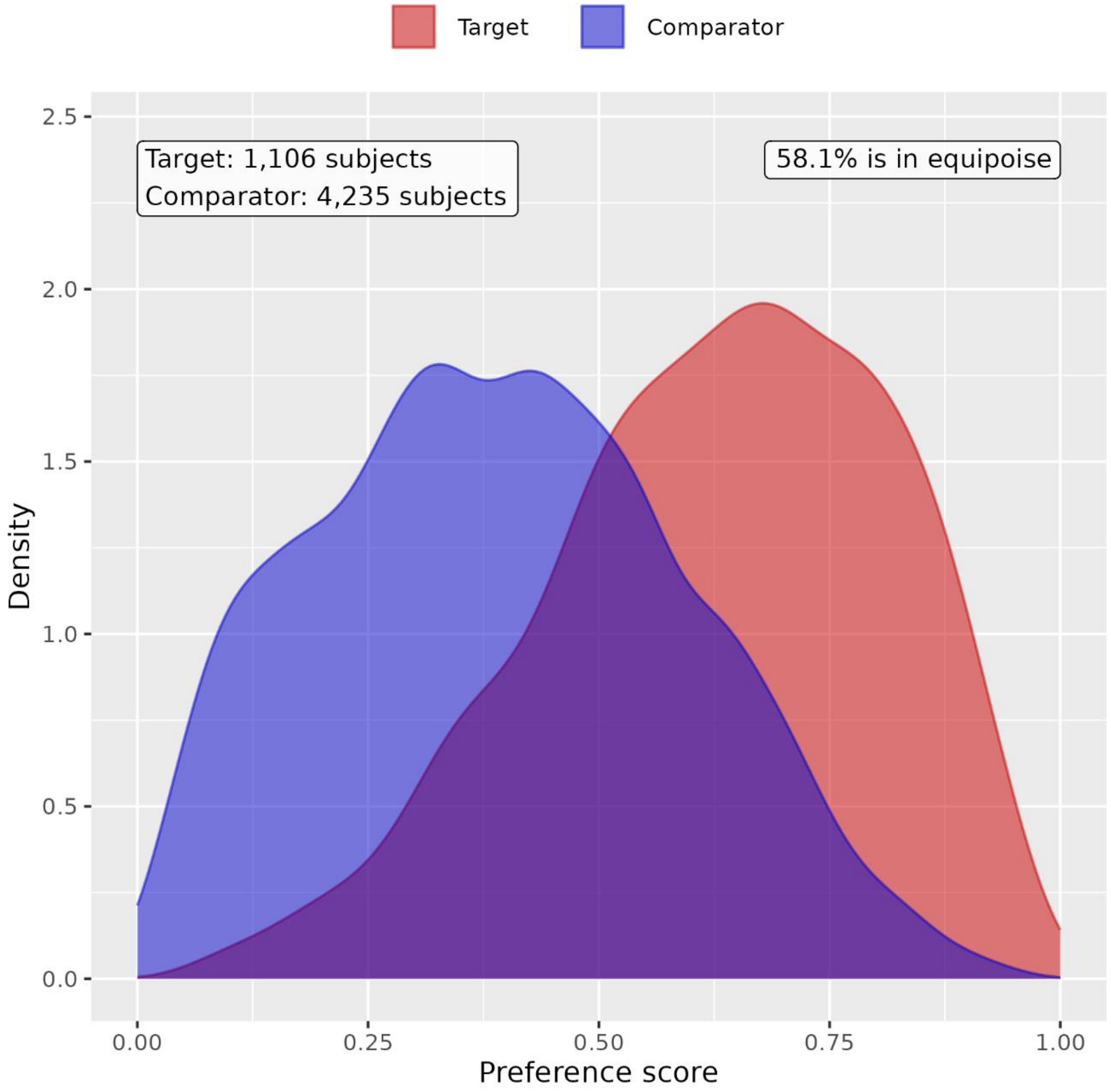

Supplement Figure 92: Preference score distributions of COVID-19 positive and negative groups for children and adolescents with AKI in mild group. A greater convergence of these distributions indicates a higher similarity in the predicted likelihood of being infected between the COVID-19 positive (red) and negative (blue) participants.

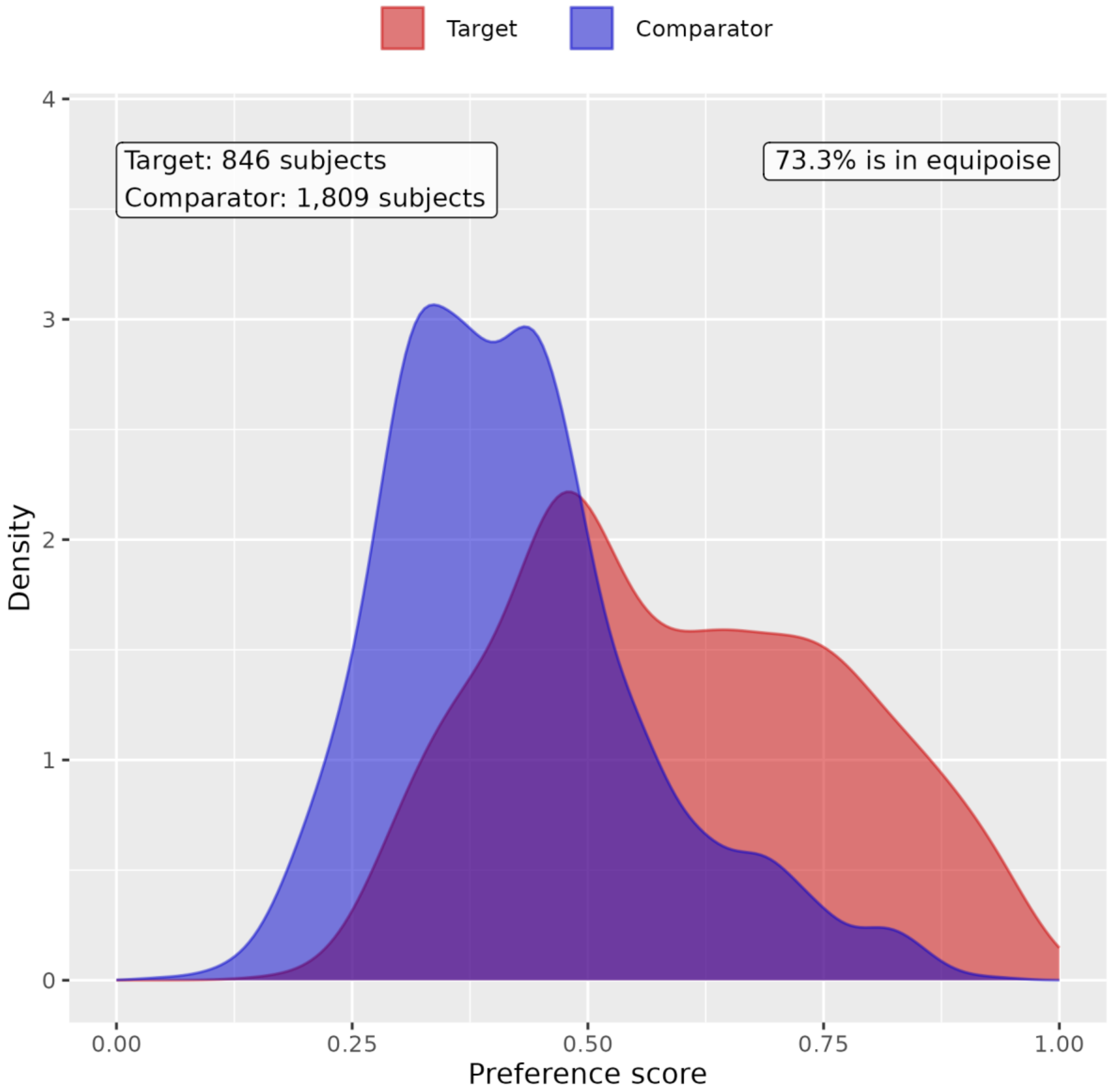

Supplement Figure 93: Preference score distributions of COVID-19 positive and negative groups for children and adolescents with AKI in moderate group. A greater convergence of these distributions indicates a higher similarity in the predicted likelihood of being infected between the COVID-19 positive (red) and negative (blue) participants.

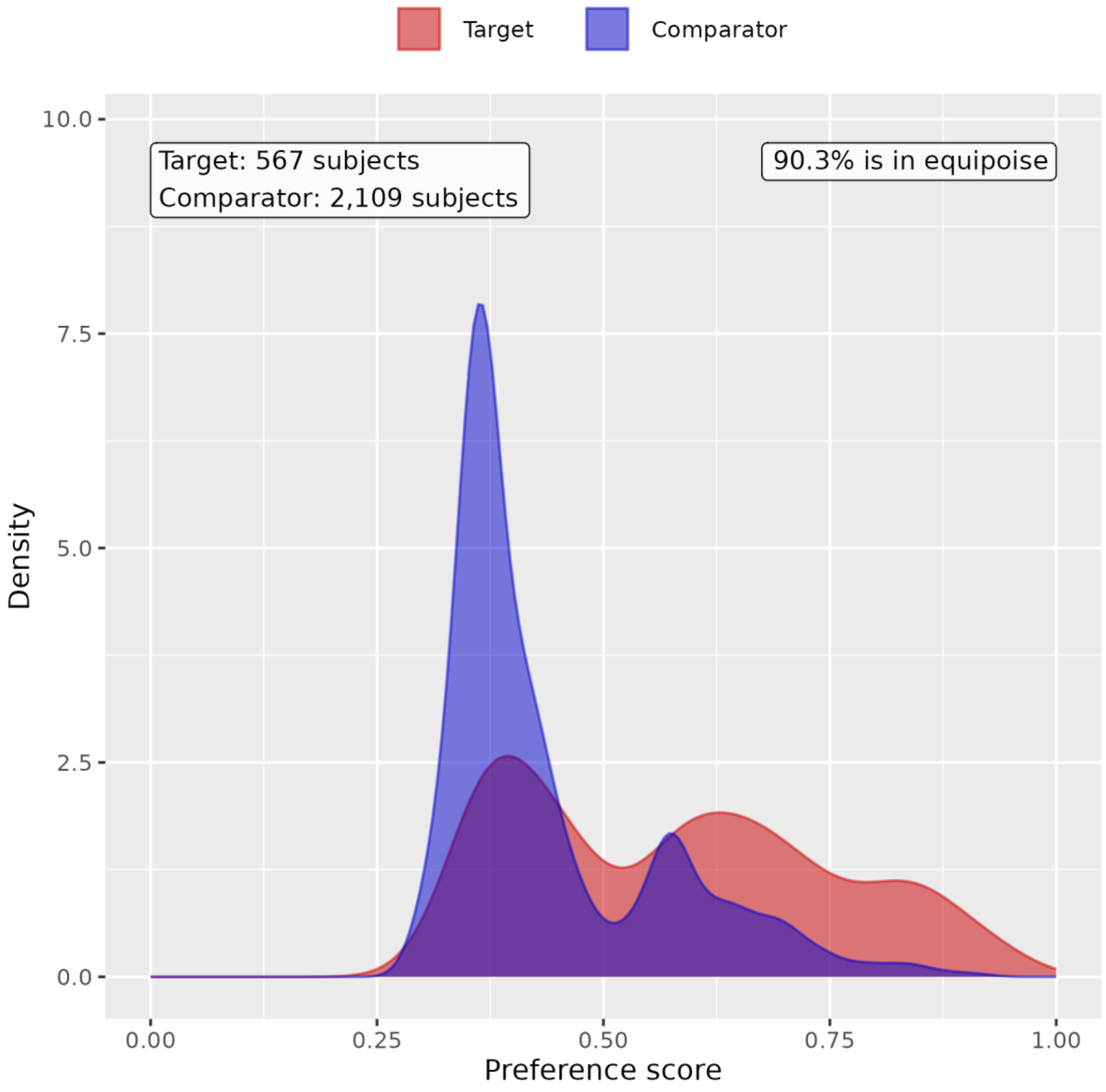

Supplement Figure 94: Preference score distributions of COVID-19 positive and negative groups for children and adolescents with AKI in severe group. A greater convergence of these distributions indicates a higher similarity in the predicted likelihood of being infected between the COVID-19 positive (red) and negative (blue) participants.

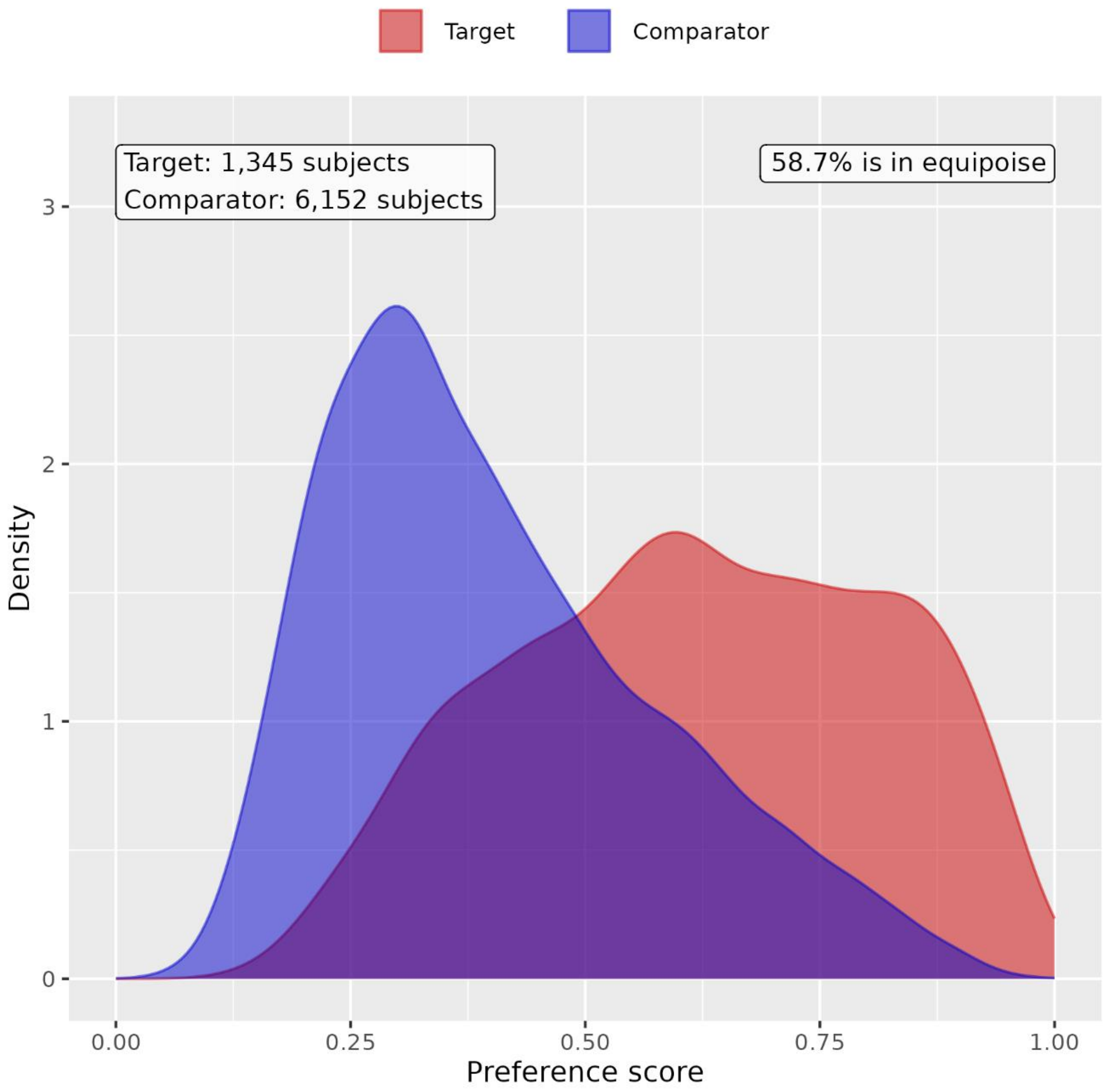

Supplement Figure 95: Preference score distributions of COVID-19 positive and control groups for children and adolescents with CKD in asymptomatic group. A greater convergence of these distributions indicates a higher similarity in the predicted likelihood of being infected between the COVID-19 positive (red) and negative (blue) participants.

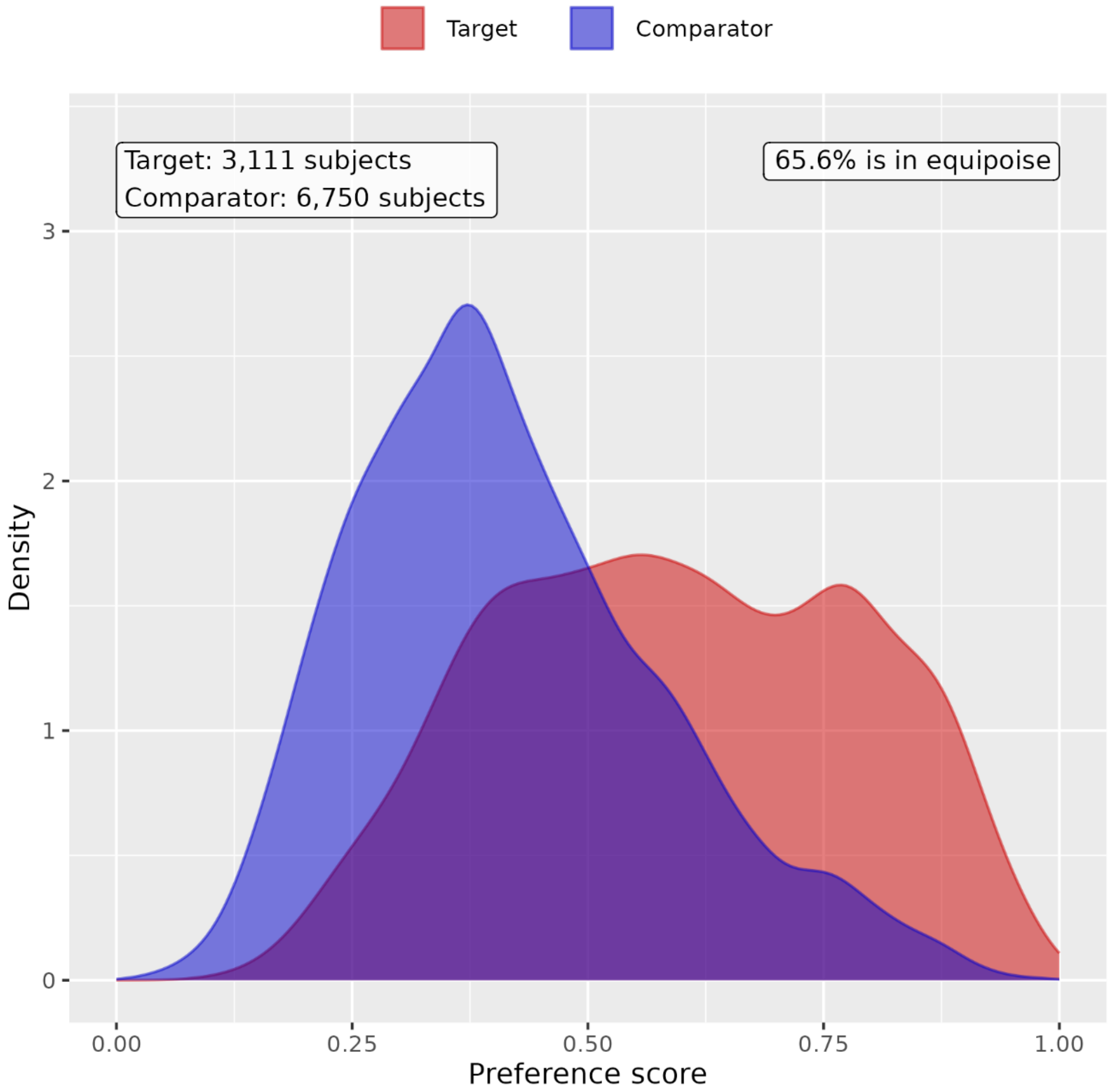

Supplement Figure 96: Preference score distributions of COVID-19 positive and negative groups for children and adolescents with CKD in mild group. A greater convergence of these distributions indicates a higher similarity in the predicted likelihood of being infected between the COVID-19 positive (red) and negative (blue) participants.

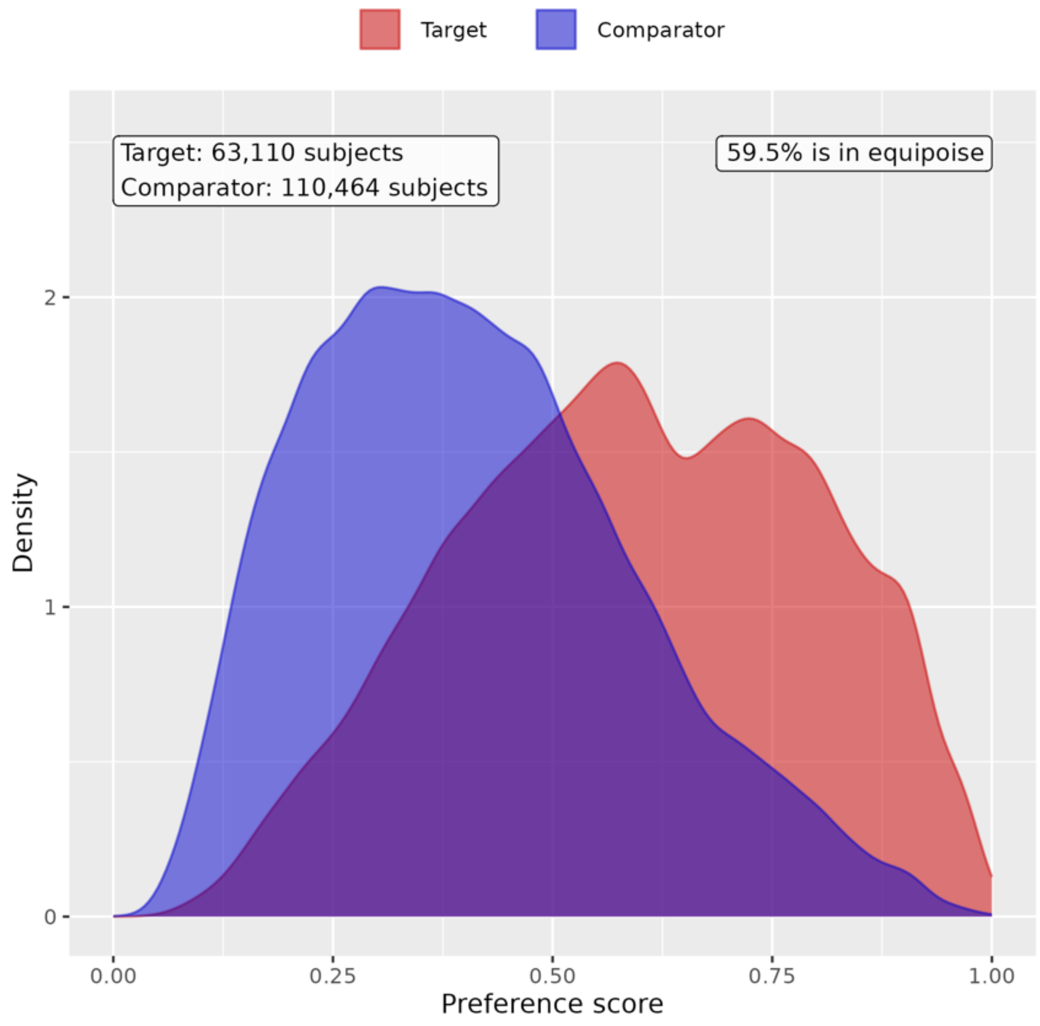

Supplement Figure 97: Preference score distributions of COVID-19 positive and negative groups for children and adolescents with CKD in moderate group. A greater convergence of these distributions indicates a higher similarity in the predicted likelihood of being infected between the COVID-19 positive (red) and negative (blue) participants.

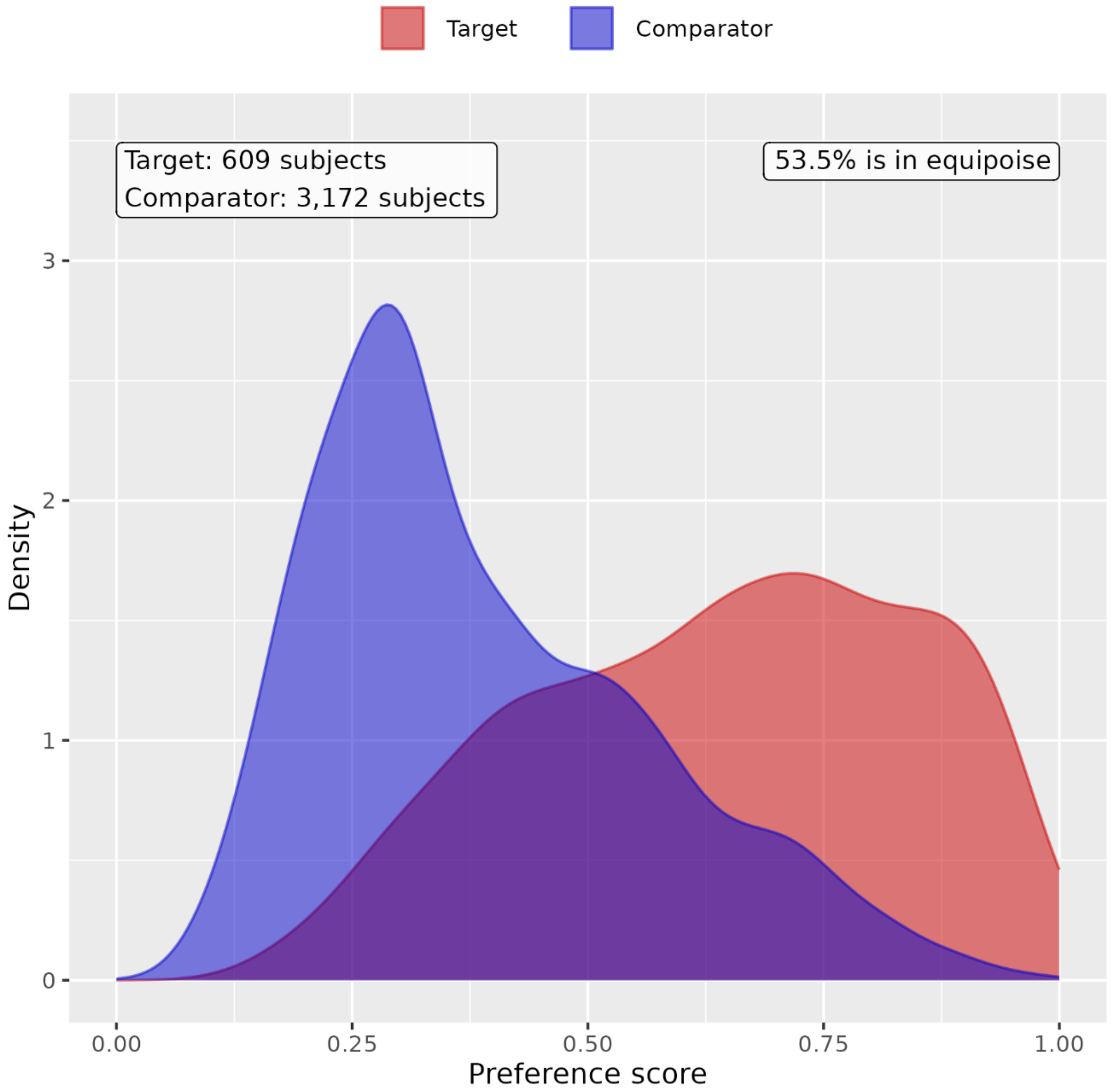

Supplement Figure 98: Preference score distributions of COVID-19 positive and negative groups for children and adolescents with CKD in severe group. A greater convergence of these distributions indicates a higher similarity in the predicted likelihood of being infected between the COVID-19 positive (red) and negative (blue) participants.

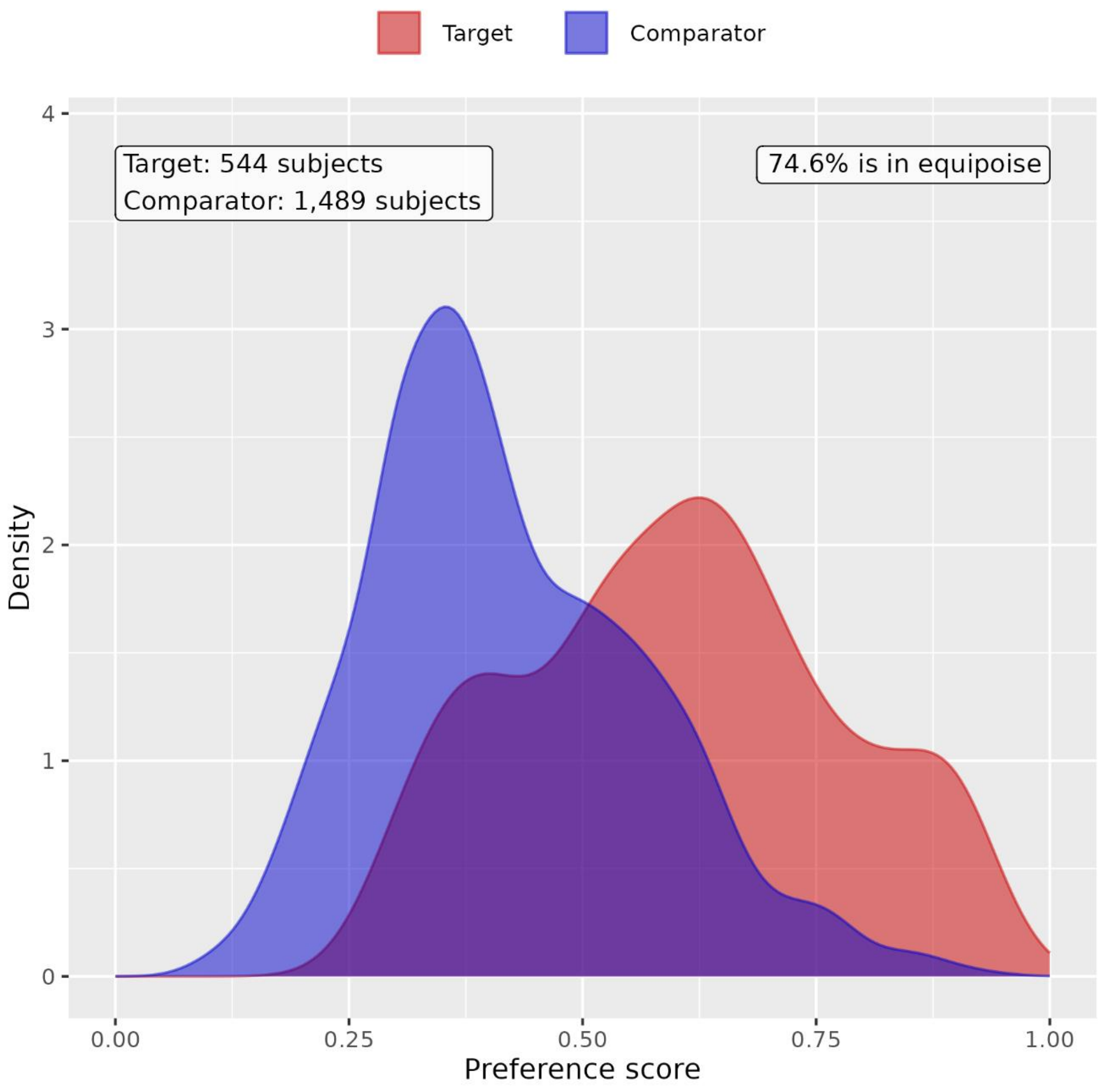

Supplement Figure 99: Preference score distributions of COVID-19 positive and negative groups for children and adolescents with no AKI or CKD in asymptomatic group. A greater convergence of these distributions indicates a higher similarity in the predicted likelihood of being infected between the COVID-19 positive (red) and negative (blue) participants.

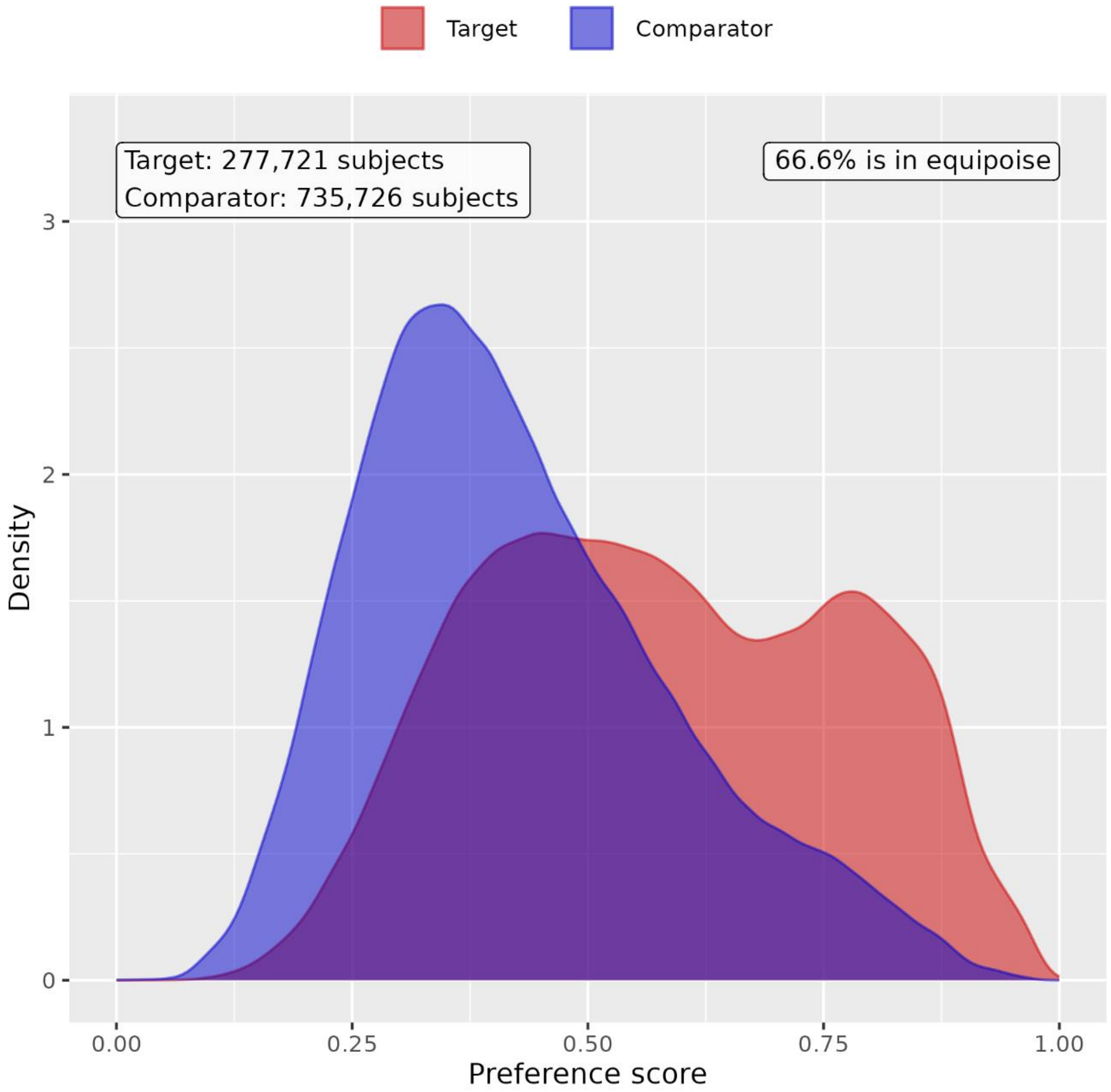

Supplement Figure 100: Preference score distributions of COVID-19 positive and negative groups for children and adolescents with no AKI or CKD in mild group. A greater convergence of these distributions indicates a higher similarity in the predicted likelihood of being infected between the COVID-19 positive (red) and negative (blue) participants.

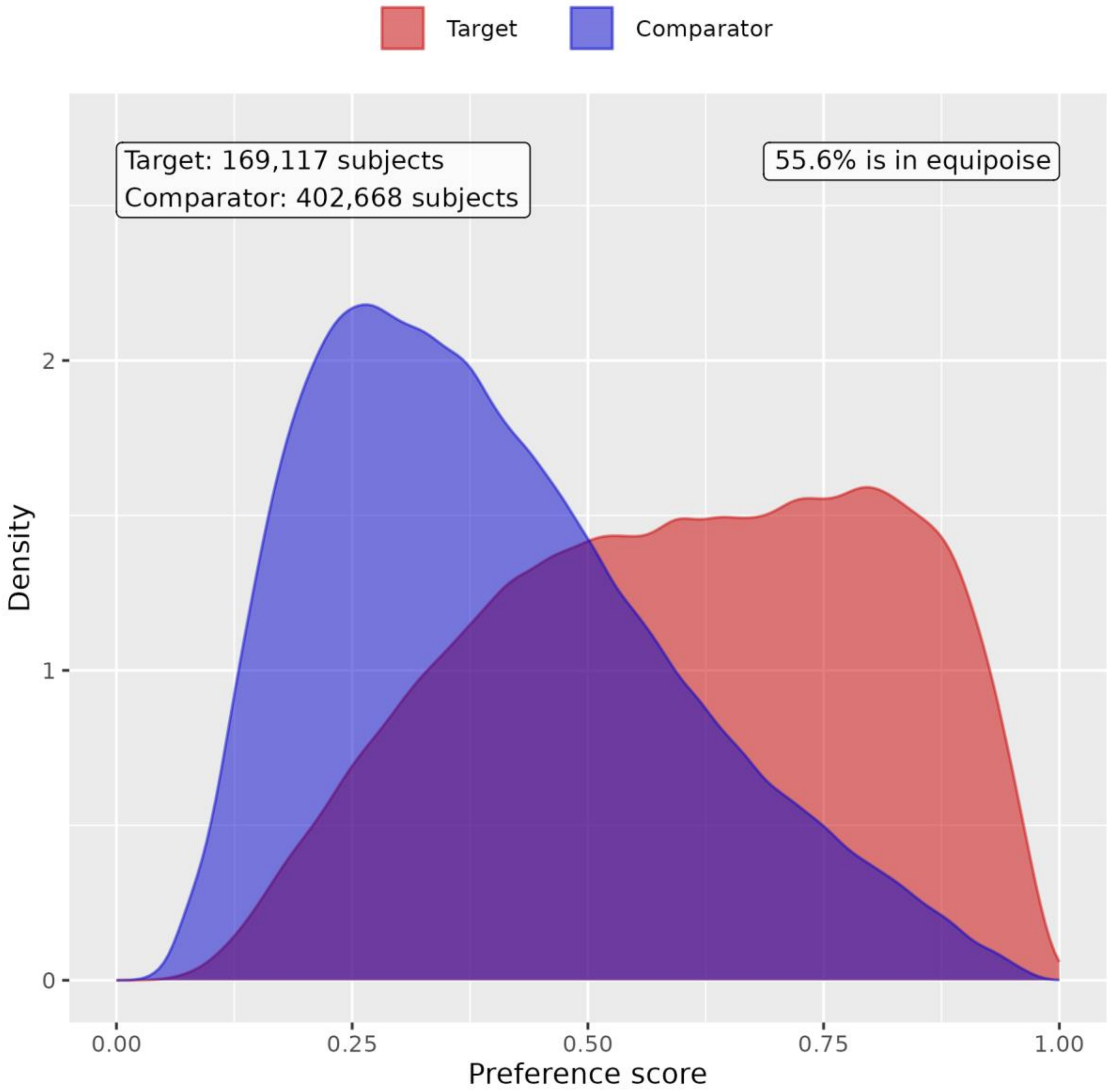

Supplement Figure 101: Preference score distributions of COVID-19 positive and negative groups for children and adolescents with no AKI or CKD in moderate group. A greater convergence of these distributions indicates a higher similarity in the predicted likelihood of being infected between the COVID-19 positive (red) and negative (blue) participants.

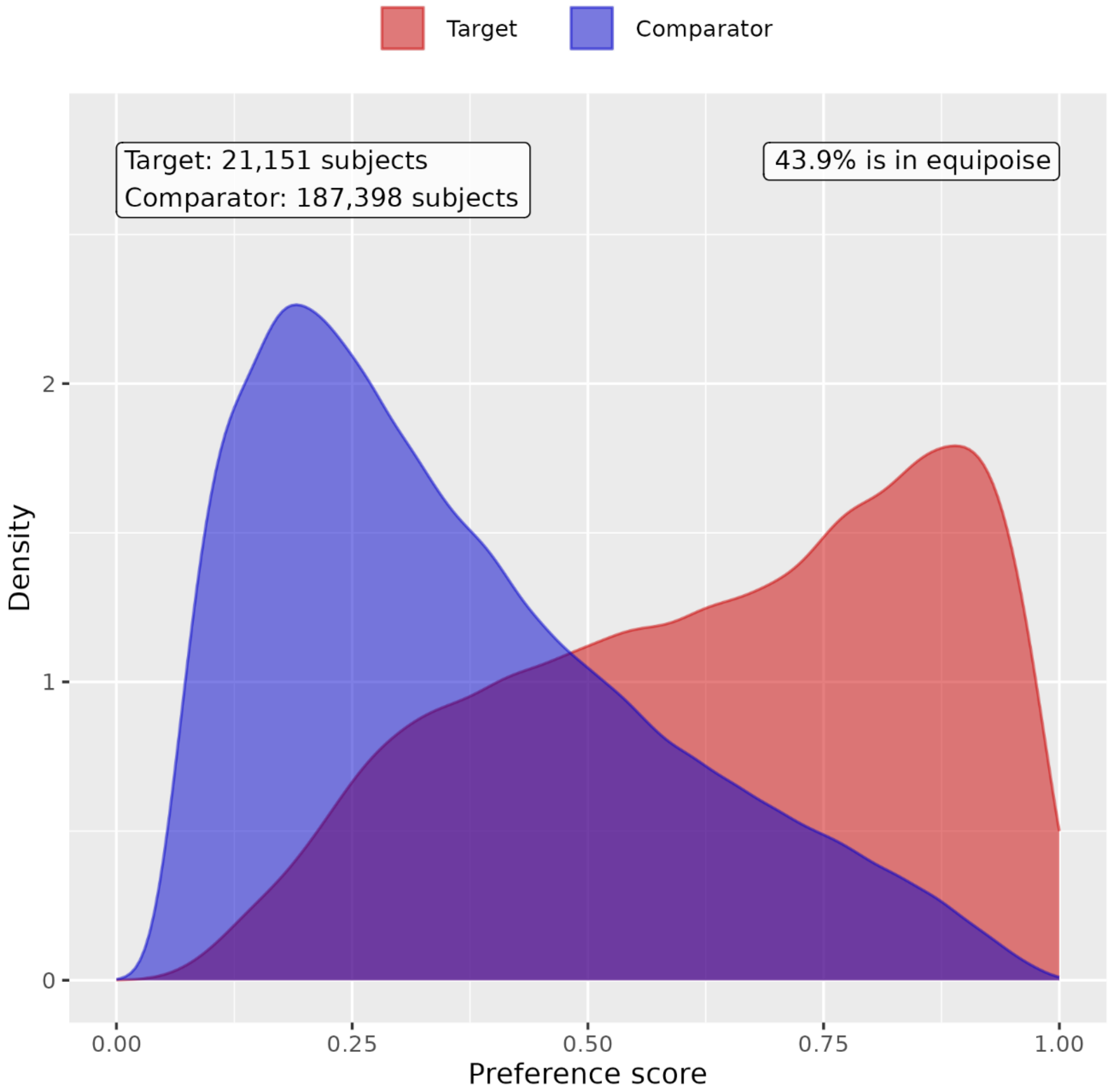

Supplement Figure 102: Preference score distributions of COVID-19 positive and negative groups for children and adolescents with no AKI or CKD in severe group. A greater convergence of these distributions indicates a higher similarity in the predicted likelihood of being infected between the COVID-19 positive (red) and negative (blue) participants.

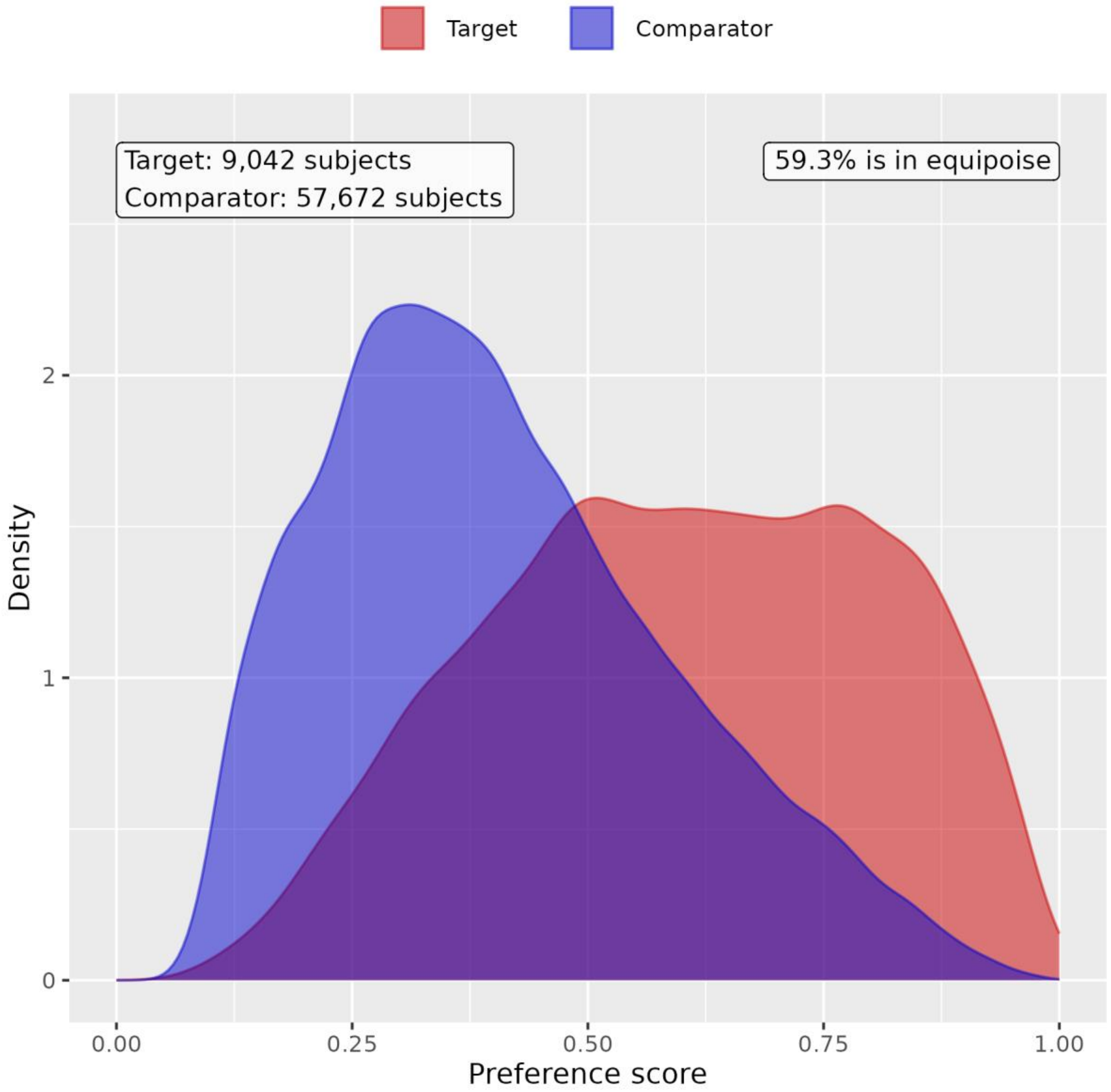

B. Patient characteristic balance

Supplement Figure 103: Patient characteristic balance before and after large-scale PS stratification with 6 strata for children and adolescents with AKI in asymptomatic group. The upper panel displays the top 20 covariates with the largest standardized difference of means before stratification, while the lower panel displays the top 20 covariates with the largest standardized difference of means after stratification.

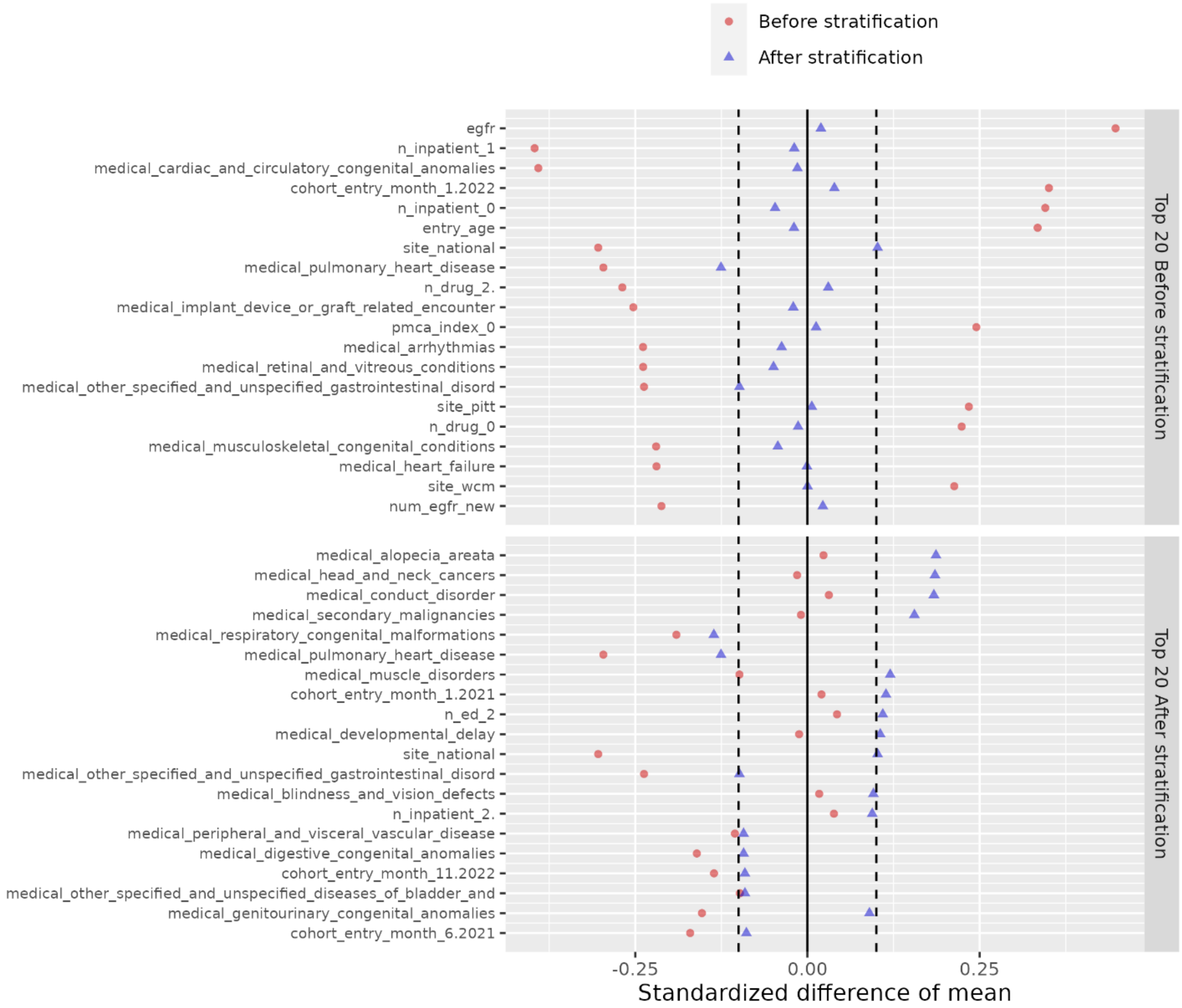

Supplement Figure 104: Patient characteristic balance before and after large-scale PS stratification with 6 strata for children and adolescents with AKI in mild group. The upper panel displays the top 20 covariates with the largest standardized difference of means before stratification, while the lower panel displays the top 20 covariates with the largest standardized difference of means after stratification.

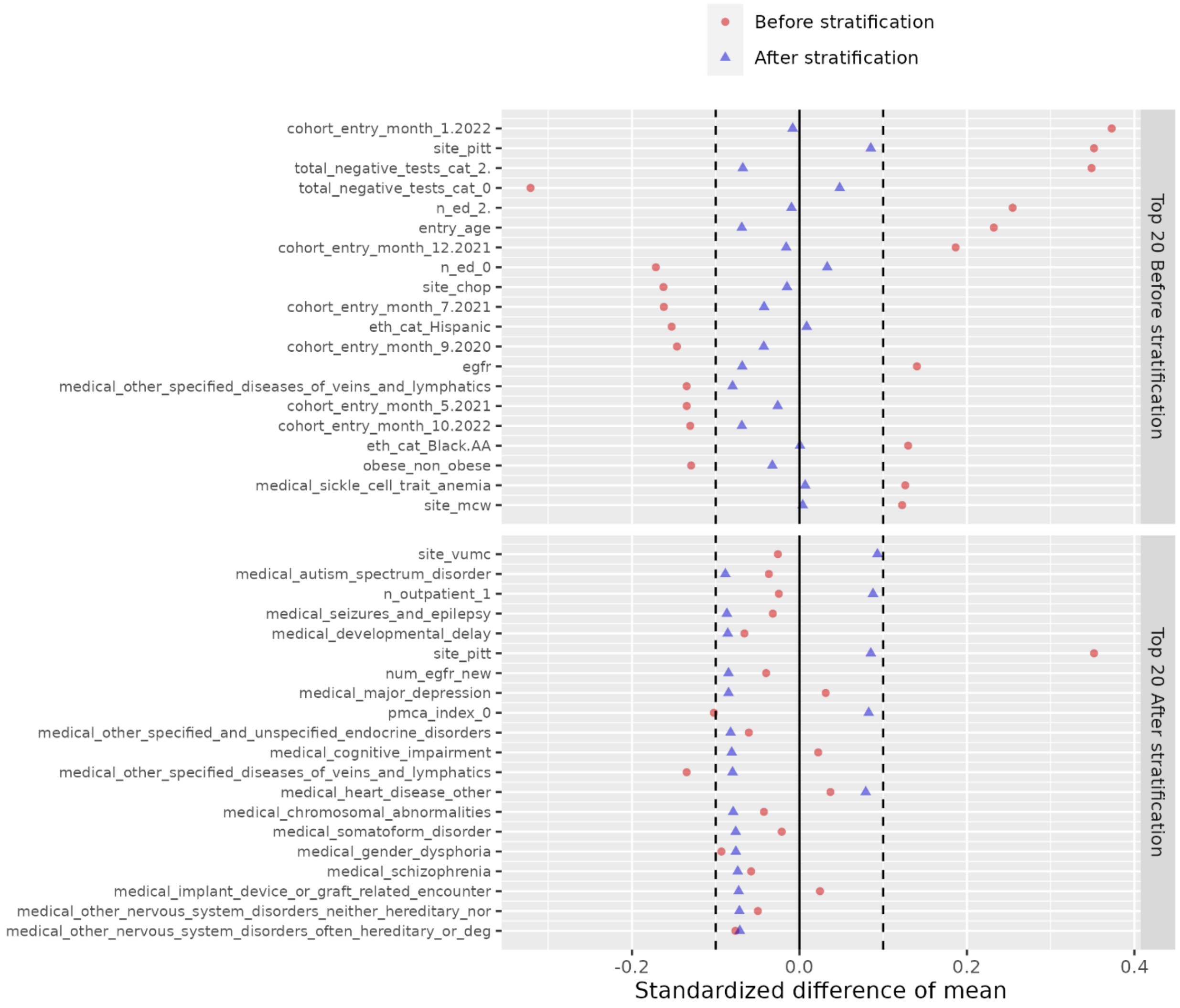

Supplement Figure 105: Patient characteristic balance before and after large-scale PS stratification with 6 strata for children and adolescents with AKI in moderate group. The upper panel displays the top 20 covariates with the largest standardized difference of means before stratification, while the lower panel displays the top 20 covariates with the largest standardized difference of means after stratification.

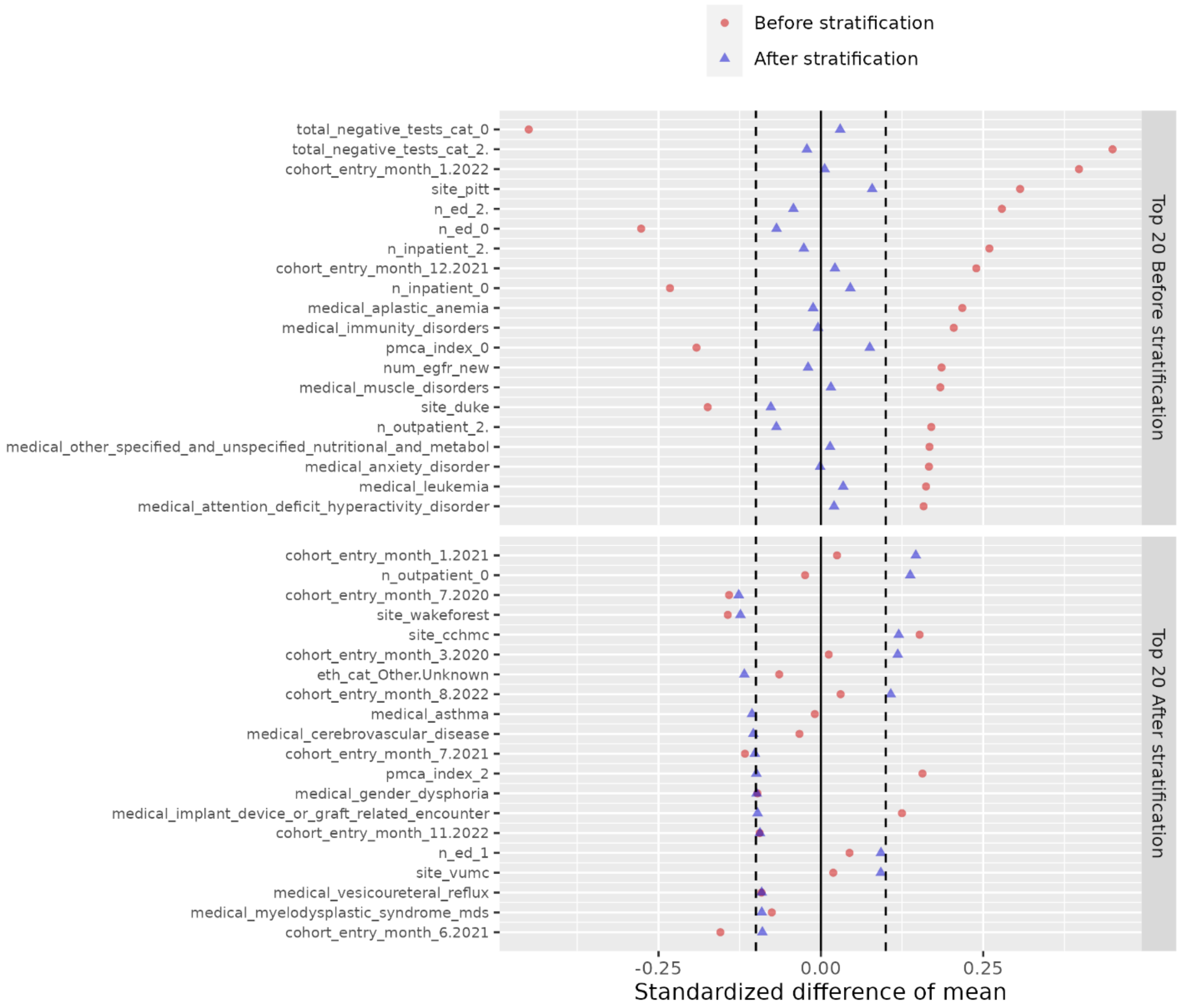

Supplement Figure 106: Patient characteristic balance before and after large-scale PS stratification with 6 strata for children and adolescents with AKI in severe group. The upper panel displays the top 20 covariates with the largest standardized difference of means before stratification, while the lower panel displays the top 20 covariates with the largest standardized difference of means after stratification.

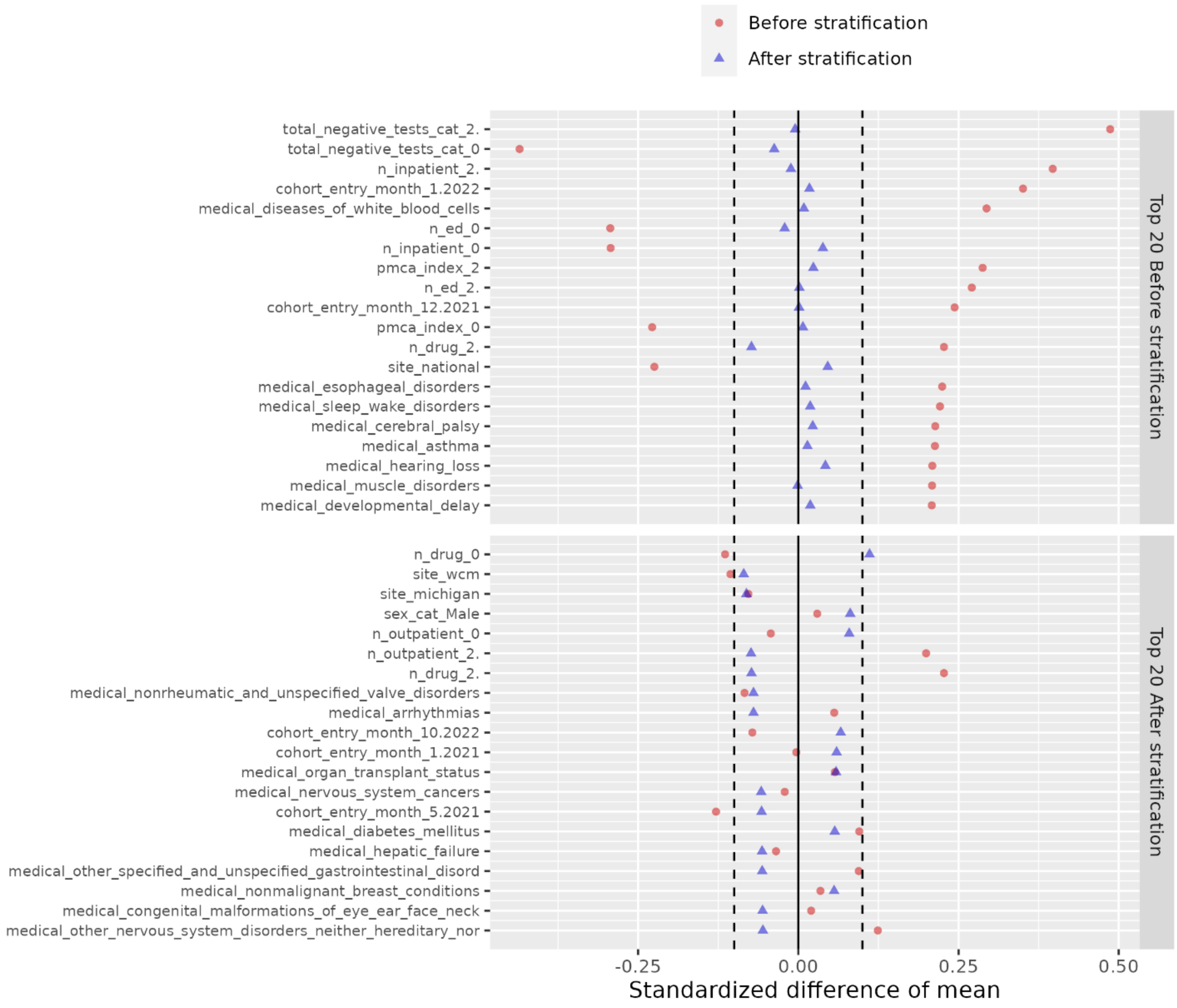

Supplement Figure 107: Patient characteristic balance before and after large-scale PS stratification with 6 strata for children and adolescents with CKD in asymptomatic group. The upper panel displays the top 20 covariates with the largest standardized difference of means before stratification, while the lower panel displays the top 20 covariates with the largest standardized difference of means after stratification.

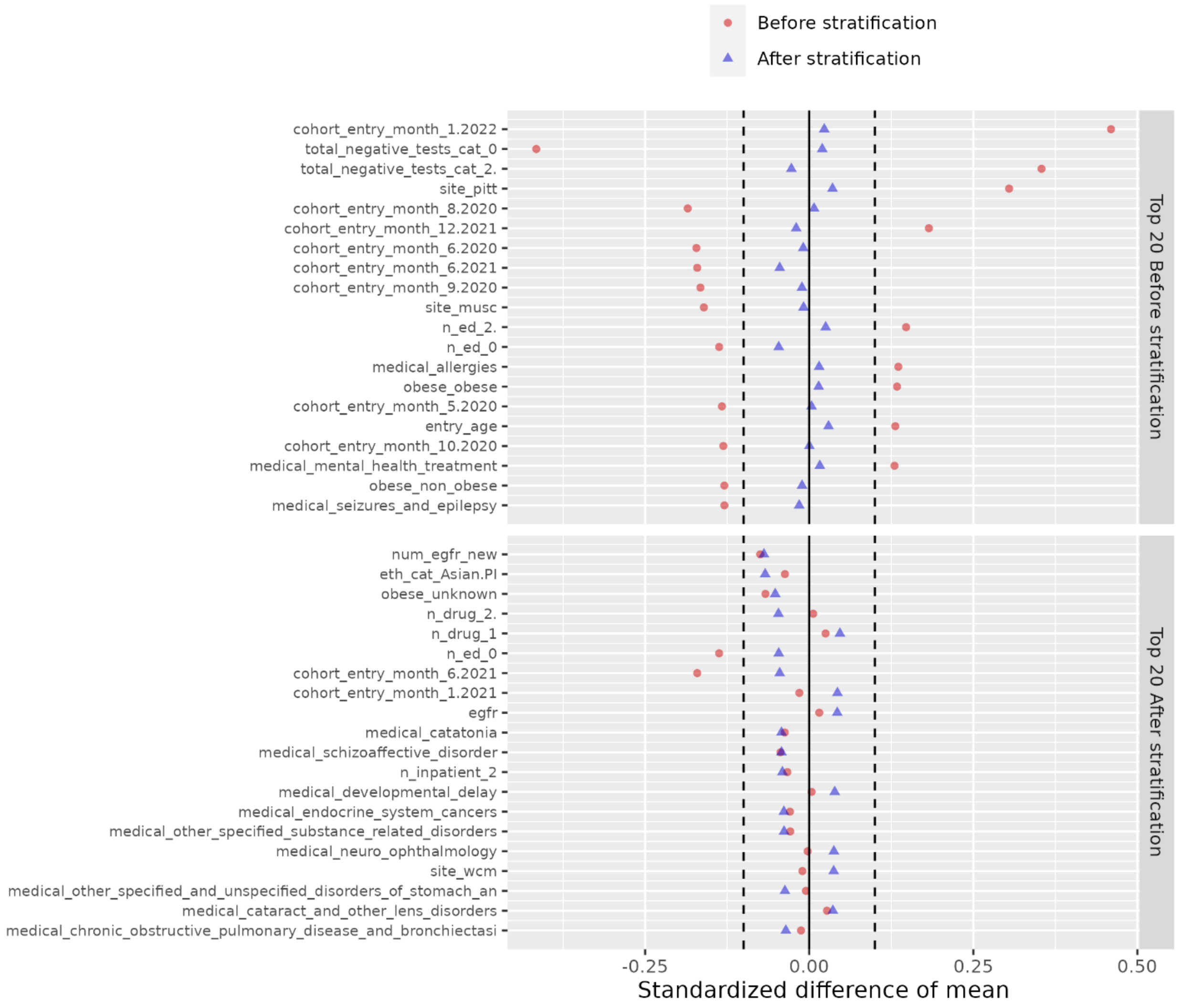

Supplement Figure 108: Patient characteristic balance before and after large-scale PS stratification with 6 strata for children and adolescents with CKD in mild group. The upper panel displays the top 20 covariates with the largest standardized difference of means before stratification, while the lower panel displays the top 20 covariates with the largest standardized difference of means after stratification.

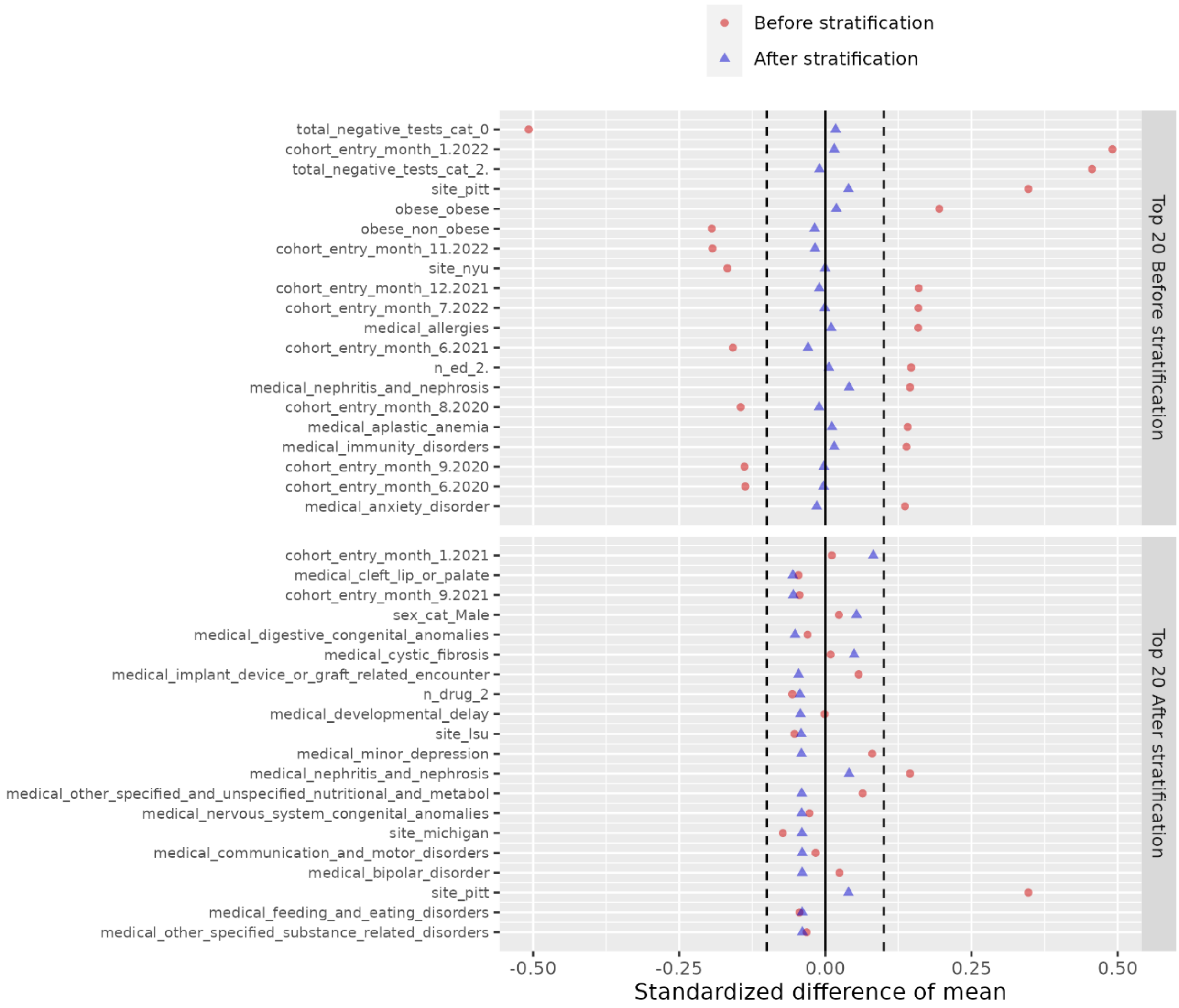

Supplement Figure 109: Patient characteristic balance before and after large-scale PS stratification with 6 strata for children and adolescents with CKD in moderate group. The upper panel displays the top 20 covariates with the largest standardized difference of means before stratification, while the lower panel displays the top 20 covariates with the largest standardized difference of means after stratification.

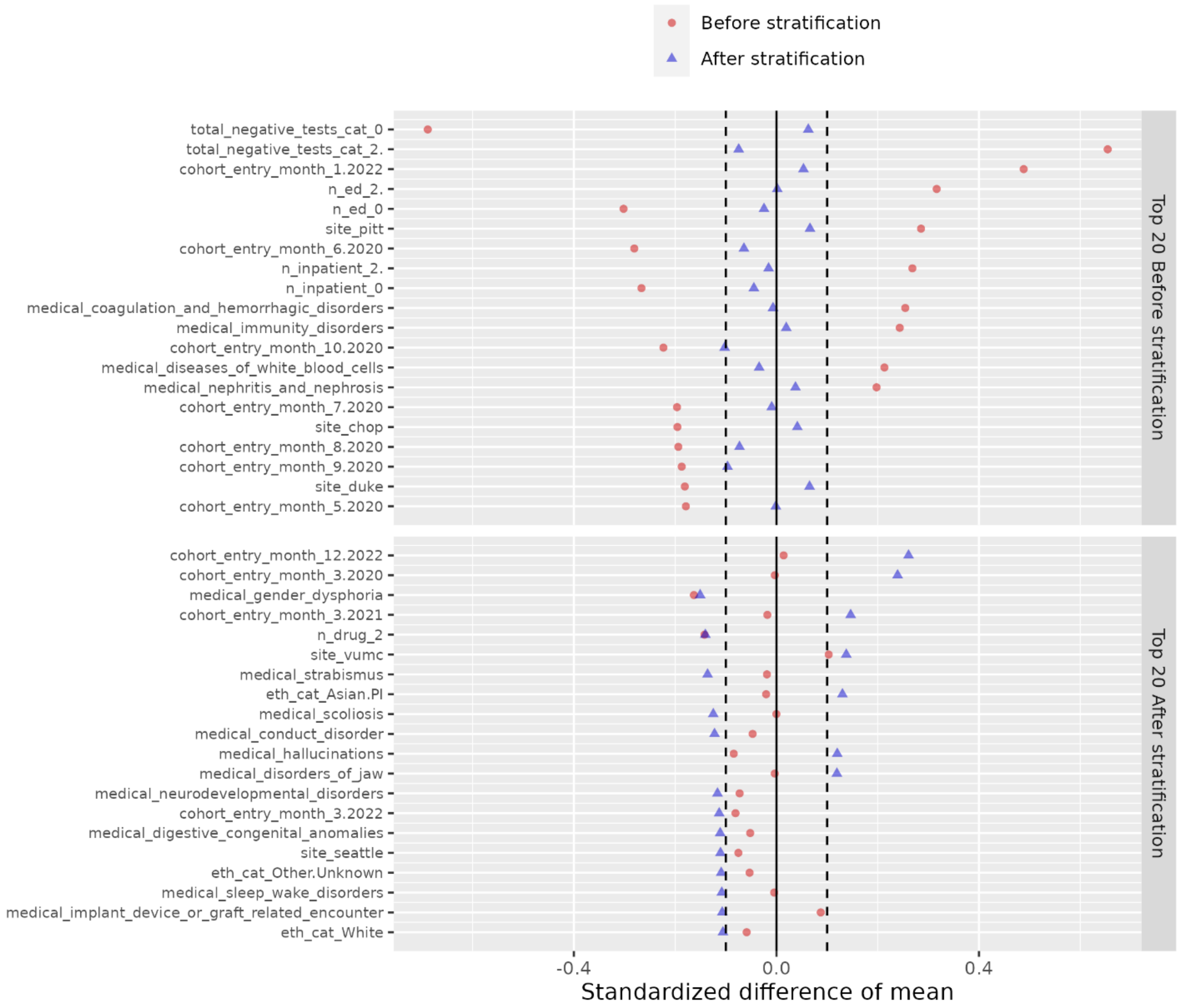

Supplement Figure 110: Patient characteristic balance before and after large-scale PS stratification with 6 strata for children and adolescents with CKD in severe group. The upper panel displays the top 20 covariates with the largest standardized difference of means before stratification, while the lower panel displays the top 20 covariates with the largest standardized difference of means after stratification.

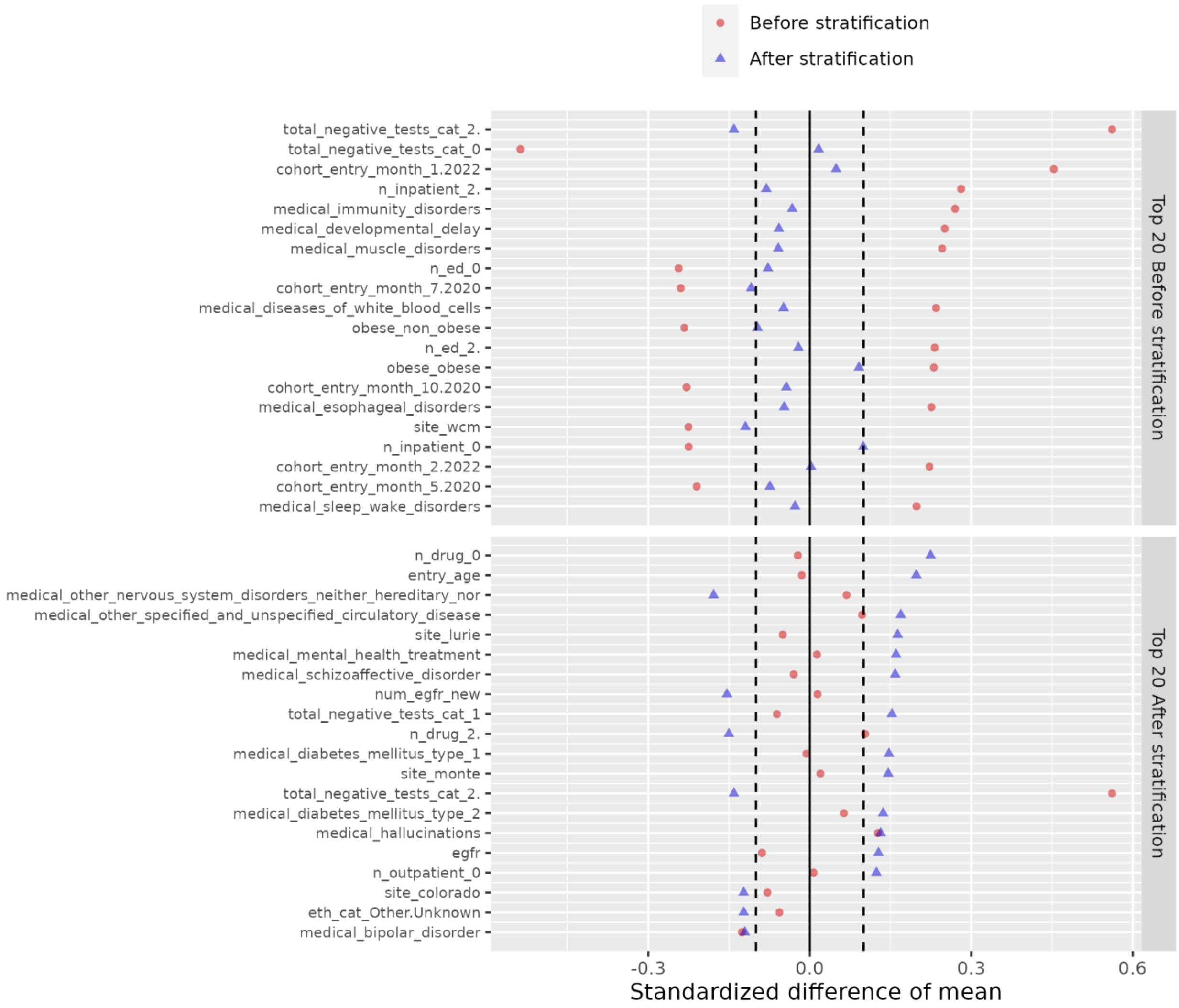

Supplement Figure 111: Patient characteristic balance before and after large-scale PS stratification with 6 strata for children and adolescents with no AKI or CKD in asymptomatic group. The upper panel displays the top 20 covariates with the largest standardized difference of means before stratification, while the lower panel displays the top 20 covariates with the largest standardized difference of means after stratification.

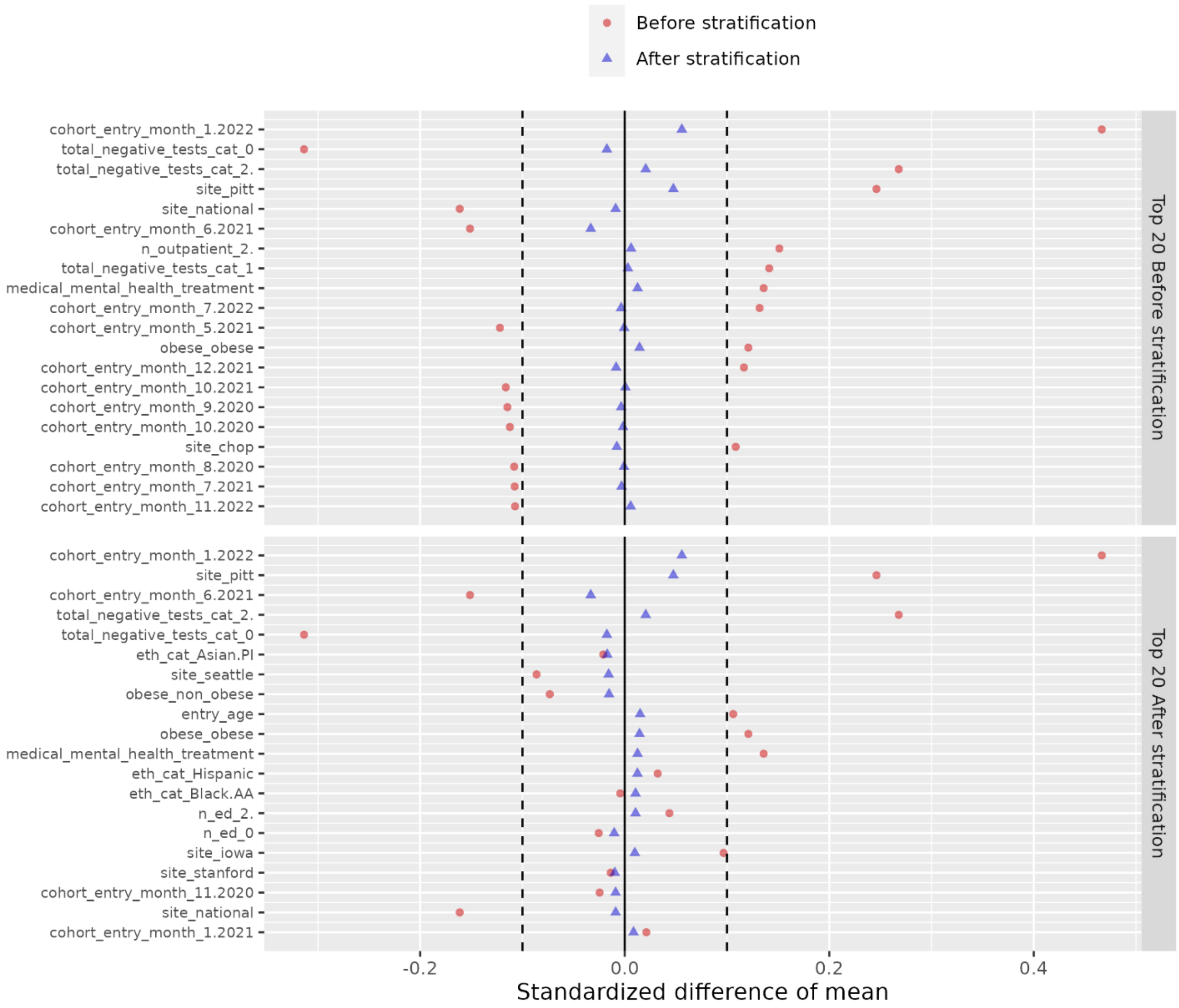

Supplement Figure 112: Patient characteristic balance before and after large-scale PS stratification with 6 strata for children and adolescents with no AKI or CKD in mild group. The upper panel displays the top 20 covariates with the largest standardized difference of means before stratification, while the lower panel displays the top 20 covariates with the largest standardized difference of means after stratification.

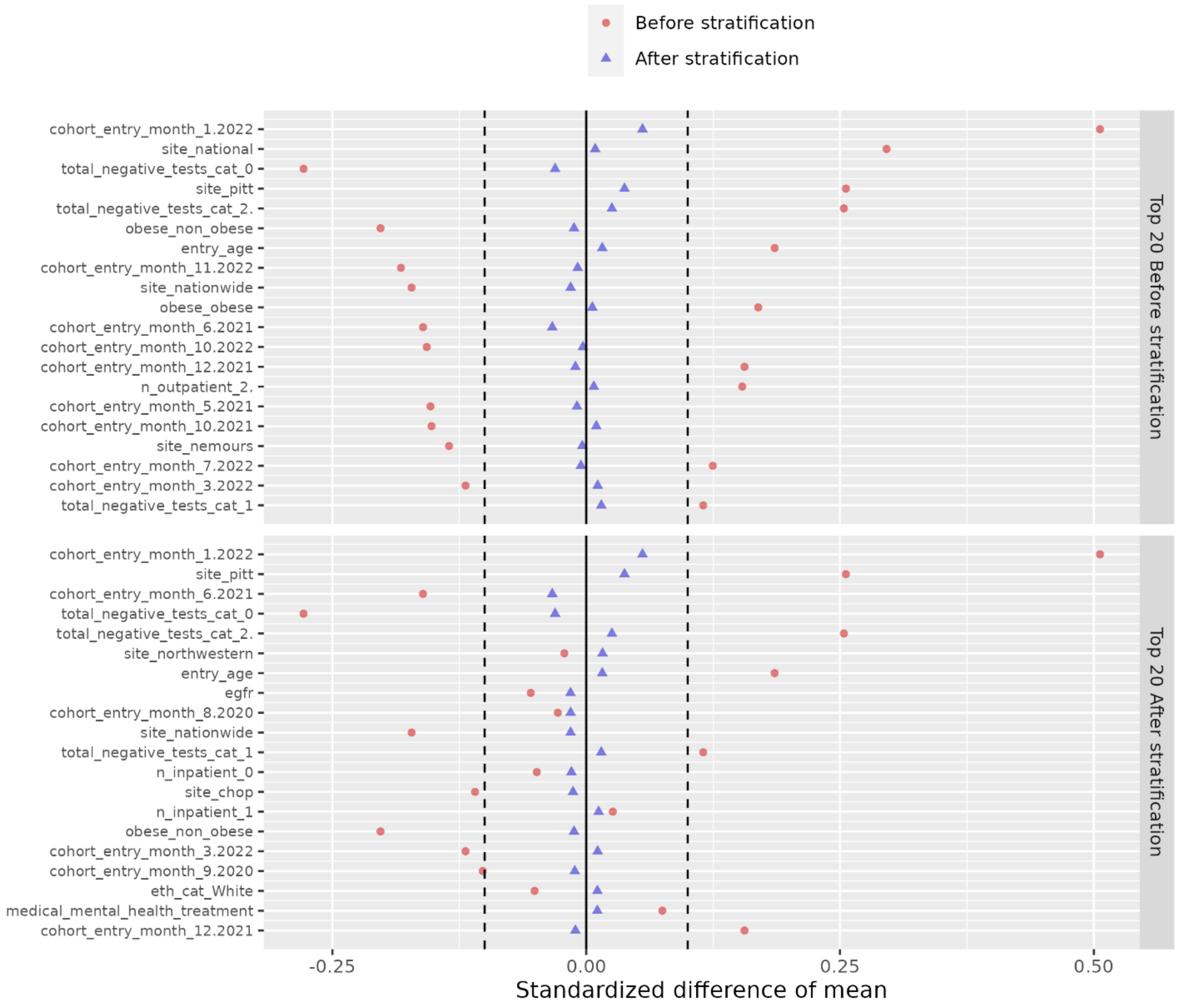

Supplement Figure 113: Patient characteristic balance before and after large-scale PS stratification with 6 strata for children and adolescents with no AKI or CKD in moderate group. The upper panel displays the top 20 covariates with the largest standardized difference of means before stratification, while the lower panel displays the top 20 covariates with the largest standardized difference of means after stratification.

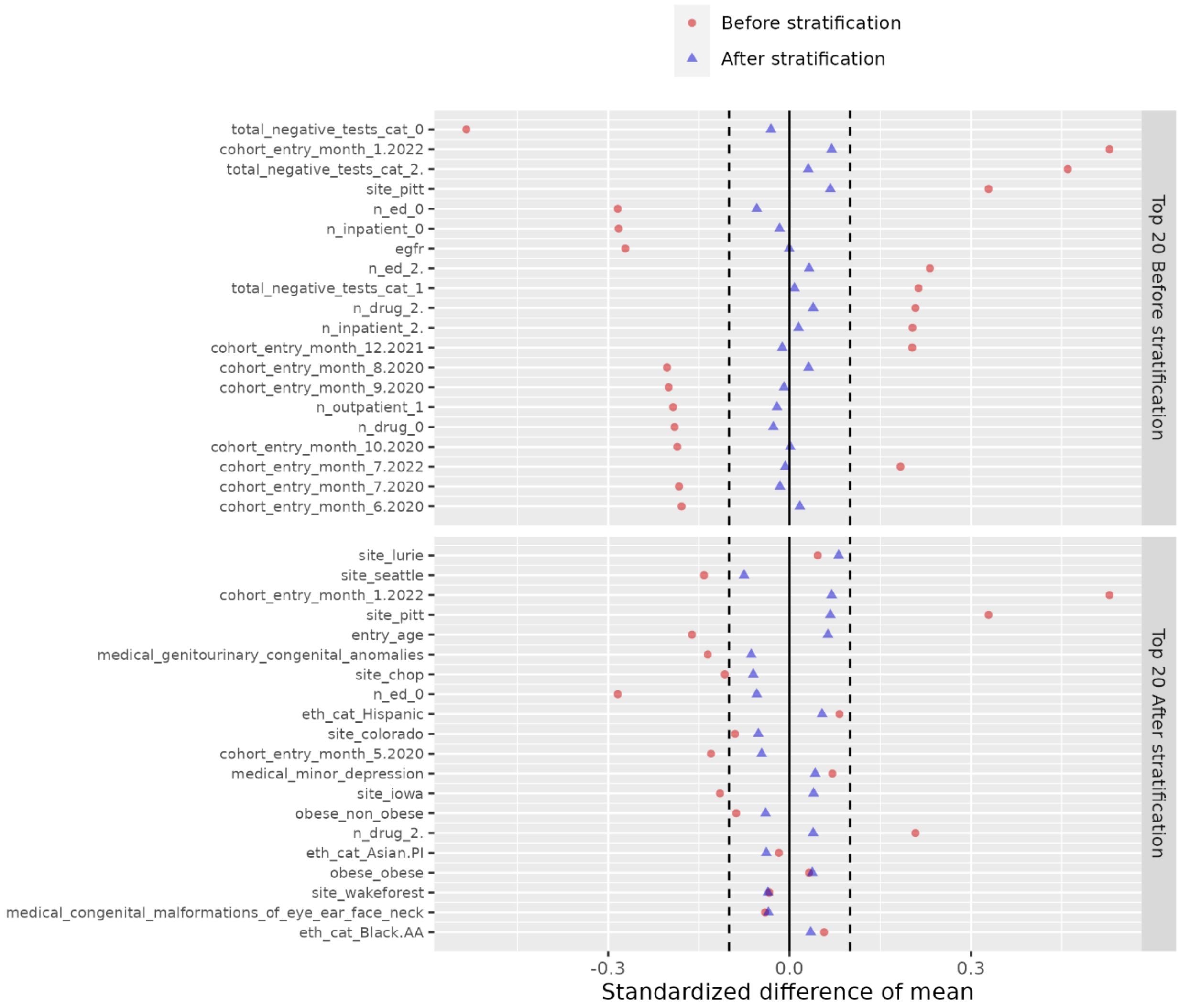

Supplement Figure 114: Patient characteristic balance before and after large-scale PS stratification with 6 strata for children and adolescents with no AKI or CKD in severe group. The upper panel displays the top 20 covariates with the largest standardized difference of means before stratification, while the lower panel displays the top 20 covariates with the largest standardized difference of means after stratification.

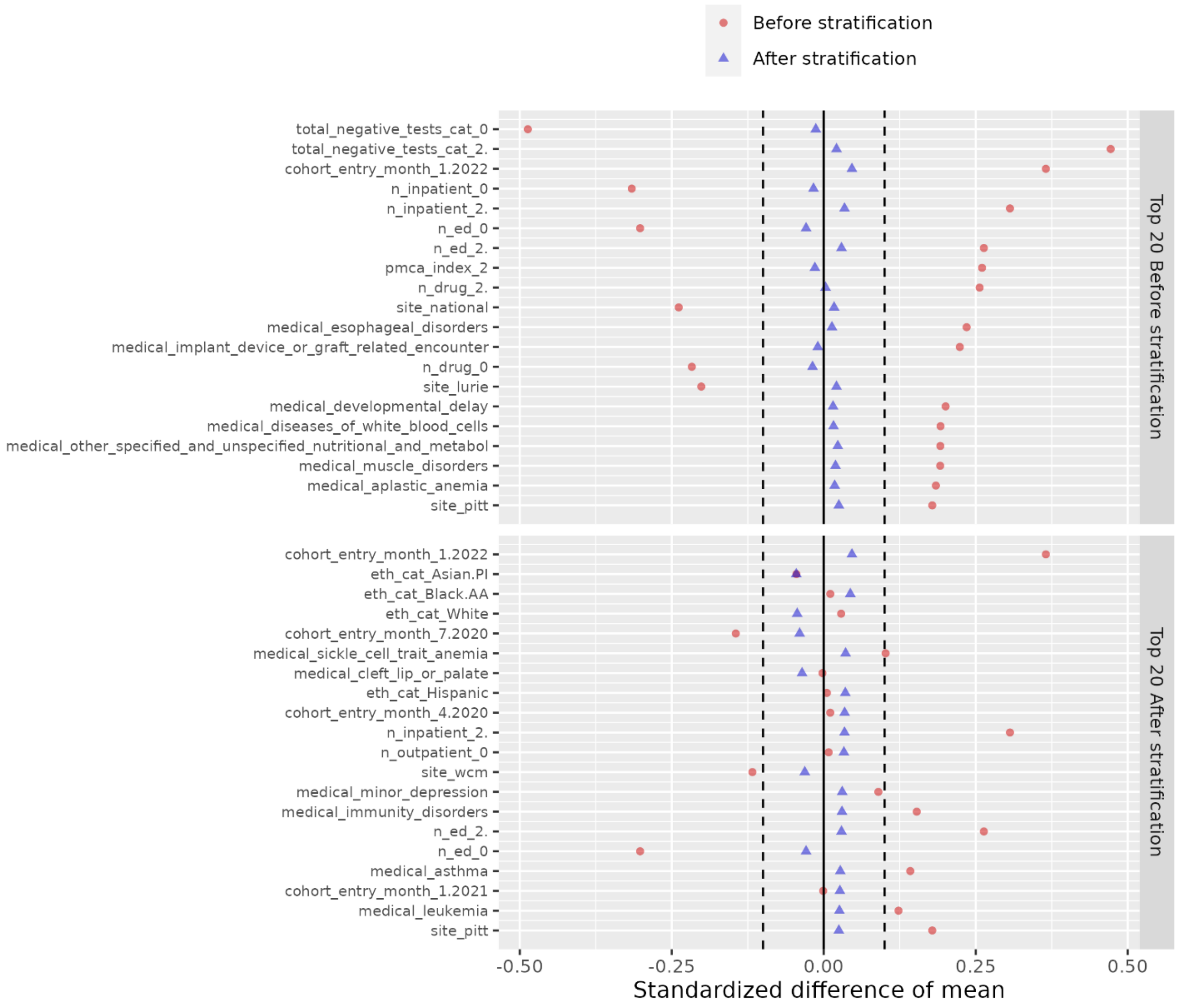

C. Hazard ratio of COVID-19 positive group compared to control group

Supplement Table 16: Estimated hazard ratio in kidney function outcomes between the COVID-19 positive cohort and the control cohort for children and adolescents in asymptomatic group

Severity: Asymptomatic	COVID-19 Groups Event (%)	Control Group Event (%)	HR (95% CI)
No CKD or AKI			
CKD 2+ (days 28-729)	794 (0.29%)	1843 (0.25%)	1.16 [1.08, 1.25]
CKD 2+ (majority not returned to 90 and above) (days 28-729)	704 (0.25%)	1558 (0.21%)	1.20 [1.12, 1.29]
CKD 2+ (not returned to 90 and above) (days 28-729)	665 (0.24%)	1406 (0.19%)	1.23 [1.14, 1.33]
CKD 3+ (days 28-729)	46 (0.02%)	121 (0.02%)	1.35 [1.19, 1.52]
CKD 3+ (majority not returned to 90 and above) (days 28-729)	36 (0.01%)	85 (0.01%)	1.45 [1.06, 1.99]
CKD 3+ (not returned to 90 and above) (days 28-729)	40 (0.01%)	109 (0.01%)	1.25 [0.97, 1.62]
CKD 3+ (not returned to 60 and above) (days 28-729)	34 (0.01%)	75 (0.01%)	1.51 [1.07, 2.14]
CKD			
Composite outcome (days 28-179)	379 (12.18%)	715 (10.59%)	1.15 [1.08, 1.23]
Composite outcome (days 180-729)	548 (17.61%)	1093 (16.19%)	1.18 [1.07, 1.31]
eGFR decline of 50% or more (days 28-179)	67 (2.15%)	130 (1.93%)	1.06 [0.87, 1.29]
eGFR decline of 50% or more (days 180-729)	116 (3.73%)	190 (2.81%)	1.40 [1.23, 1.60]
eGFR decline of 40% or more (days 28-179)	183 (5.88%)	343 (5.08%)	1.09 [0.98, 1.21]
eGFR decline of 40% or more (days 180-729)	276 (8.87%)	526 (7.79%)	1.19 [1.05, 1.34]
eGFR decline of 30% or more (days 28-179)	373 (11.99%)	715 (10.59%)	1.13 [1.06, 1.21]
eGFR decline of 30% or more (days 180-729)	547 (17.58%)	1093 (16.19%)	1.18 [1.06, 1.31]
AKI			
Composite outcome (days 90-179)	50 (4.52%)	165 (3.9%)	1.04 [0.82, 1.32]
Composite outcome (days 180-729)	89 (8.05%)	284 (6.71%)	0.96 [0.71, 1.30]
eGFR decline of 30% or more (days 90-179)	50 (4.52%)	165 (3.9%)	1.04 [0.82, 1.32]
eGFR decline of 30% or more (days 180-729)	87 (7.87%)	284 (6.71%)	0.94 [0.71, 1.24]
CKD 2+ (days 28-729)	59 (5.33%)	212 (5.01%)	0.85 [0.69, 1.05]
CKD 2+ (majority not returned to 90 and above) (days 28-729)	51 (4.61%)	187 (4.42%)	0.80 [0.62, 1.01]
CKD 2+ (not returned to 90 and above) (days 28-729)	47 (4.25%)	165 (3.9%)	0.79 [0.61, 1.03]

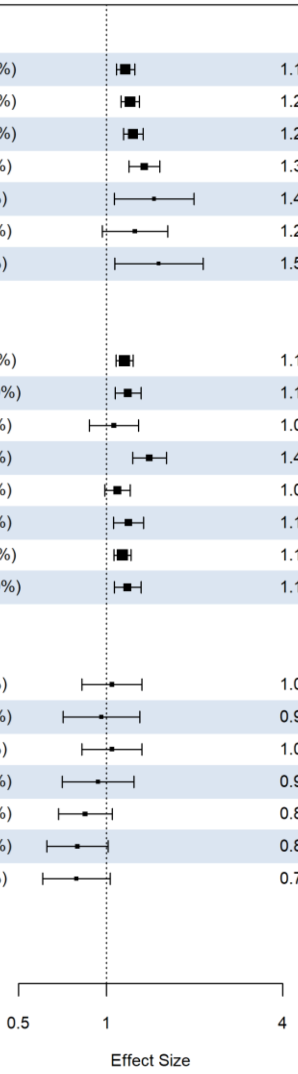

Supplement Table 17: Estimated hazard ratio in kidney function outcomes between the COVID-19 positive cohort and the control cohort for children and adolescents in mild group

Severity: Mild	COVID-19 Groups Event (%)	Control Group Event (%)	HR (95% CI)
No CKD or AKI			
CKD 2+ (days 28-729)	516 (0.31%)	850 (0.21%)	1.26 [1.14, 1.39]
CKD 2+ (majority not returned to 90 and above) (days 28-729)	450 (0.27%)	750 (0.19%)	1.25 [1.11, 1.40]
CKD 2+ (not returned to 90 and above) (days 28-729)	420 (0.25%)	694 (0.17%)	1.26 [1.12, 1.41]
CKD			
Composite outcome (days 28-179)	284 (12.8%)	371 (10.34%)	1.16 [0.94, 1.43]
Composite outcome (days 180-729)	370 (16.67%)	549 (15.3%)	1.10 [0.88, 1.37]
eGFR decline of 50% or more (days 28-179)	48 (2.16%)	78 (2.17%)	0.91 [0.56, 1.46]
eGFR decline of 50% or more (days 180-729)	60 (2.7%)	118 (3.29%)	0.82 [0.63, 1.05]
eGFR decline of 40% or more (days 28-179)	147 (6.62%)	196 (5.46%)	1.12 [0.71, 1.76]
eGFR decline of 40% or more (days 180-729)	196 (8.83%)	286 (7.97%)	1.18 [0.92, 1.52]
eGFR decline of 30% or more (days 28-179)	282 (12.71%)	371 (10.34%)	1.15 [0.93, 1.43]
eGFR decline of 30% or more (days 180-729)	367 (16.54%)	549 (15.3%)	1.09 [0.88, 1.35]
AKI			
Composite outcome (days 90-179)	46 (5.44%)	81 (4.48%)	1.20 [0.89, 1.61]
Composite outcome (days 180-729)	92 (10.87%)	132 (7.3%)	1.37 [1.08, 1.74]
eGFR decline of 50% or more (days 180-729)	30 (3.55%)	53 (2.93%)	1.15 [0.91, 1.46]
eGFR decline of 40% or more (days 90-179)	24 (2.84%)	48 (2.65%)	1.10 [0.68, 1.79]
eGFR decline of 40% or more (days 180-729)	56 (6.62%)	84 (4.64%)	1.36 [1.17, 1.58]
eGFR decline of 30% or more (days 90-179)	46 (5.44%)	81 (4.48%)	1.20 [0.89, 1.61]
eGFR decline of 30% or more (days 180-729)	91 (10.76%)	132 (7.3%)	1.35 [1.07, 1.71]
CKD 2+ (days 28-729)	60 (7.09%)	92 (5.09%)	1.35 [1.02, 1.80]
CKD 2+ (majority not returned to 90 and above) (days 28-729)	59 (6.97%)	85 (4.7%)	1.40 [1.10, 1.79]
CKD 2+ (not returned to 90 and above) (days 28-729)	53 (6.26%)	82 (4.53%)	1.33 [0.98, 1.81]
CKD 3+ (days 28-729)	15 (1.77%)	20 (1.11%)	2.10 [1.35, 3.25]
CKD 3+ (majority not returned to 90 and above) (days 28-729)	13 (1.54%)	19 (1.05%)	1.80 [0.97, 3.35]
CKD 3+ (not returned to 90 and above) (days 28-729)	15 (1.77%)	19 (1.05%)	2.15 [1.37, 3.38]
CKD 3+ (not returned to 60 and above) (days 28-729)	12 (1.42%)	19 (1.05%)	1.74 [0.90, 3.35]

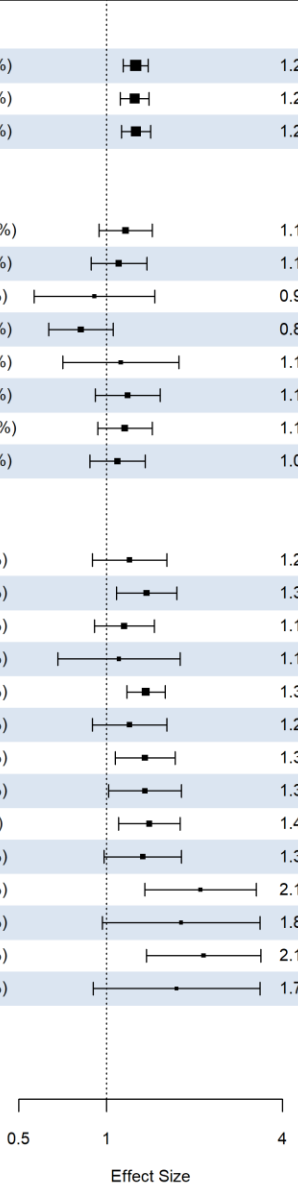

Supplement Table 18: Estimated hazard ratio in kidney function outcomes between the COVID-19 positive cohort and the control cohort for children and adolescents in moderate group

Severity: Moderate	COVID-19 Groups Event (%)	Control Group Event (%)	HR (95% CI)
No CKD or AKI			
CKD 2+ (days 28-729)	116 (0.55%)	847 (0.45%)	1.11 [0.95, 1.29]
CKD 2+ (majority not returned to 90 and above) (days 28-729)	85 (0.4%)	680 (0.36%)	1.02 [0.89, 1.16]
CKD 2+ (not returned to 90 and above) (days 28-729)	77 (0.36%)	586 (0.31%)	1.11 [0.96, 1.28]
CKD			
Composite outcome (days 28-179)	108 (17.73%)	429 (13.52%)	1.27 [1.08, 1.50]
Composite outcome (days 180-729)	124 (20.36%)	622 (19.61%)	1.22 [0.97, 1.52]
eGFR decline of 40% or more (days 28-179)	68 (11.17%)	266 (8.39%)	1.23 [1.05, 1.44]
eGFR decline of 40% or more (days 180-729)	67 (11%)	373 (11.76%)	1.16 [0.76, 1.76]
eGFR decline of 30% or more (days 28-179)	106 (17.41%)	429 (13.52%)	1.25 [1.06, 1.48]
eGFR decline of 30% or more (days 180-729)	123 (20.2%)	622 (19.61%)	1.21 [0.97, 1.51]
AKI			
Composite outcome (days 90-179)	37 (6.53%)	89 (4.22%)	1.36 [0.98, 1.88]
eGFR decline of 30% or more (days 90-179)	37 (6.53%)	89 (4.22%)	1.36 [0.98, 1.88]

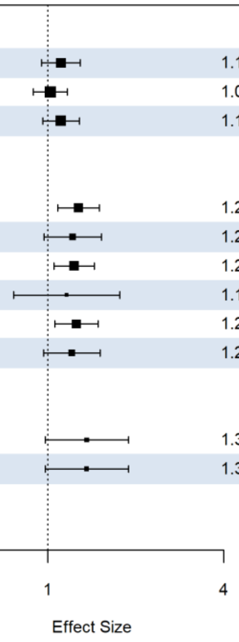

Supplement Table 19: Estimated hazard ratio in kidney function outcomes between the COVID-19 positive cohort and the control cohort for children and adolescents in severe group

Severity: Severe	COVID-19 Groups Event (%)	Control Group Event (%)	HR (95% CI)
No CKD or AKI			
CKD 2+ (days 28-729)	143 (1.58%)	360 (0.62%)	1.84 [1.54, 2.21]
CKD 2+ (majority not returned to 90 and above) (days 28-729)	115 (1.27%)	277 (0.48%)	1.96 [1.64, 2.34]
CKD 2+ (not returned to 90 and above) (days 28-729)	88 (0.97%)	223 (0.39%)	1.76 [1.44, 2.15]
CKD			
Composite outcome (days 28-179)	134 (24.63%)	231 (15.51%)	1.43 [1.05, 1.96]
Composite outcome (days 180-729)	125 (22.98%)	252 (16.92%)	1.33 [1.02, 1.73]
eGFR decline of 50% or more (days 28-179)	47 (8.64%)	76 (5.1%)	1.23 [0.95, 1.59]
eGFR decline of 40% or more (days 28-179)	86 (15.81%)	152 (10.21%)	1.20 [0.98, 1.46]
eGFR decline of 40% or more (days 180-729)	76 (13.97%)	160 (10.75%)	1.15 [0.90, 1.47]
eGFR decline of 30% or more (days 28-179)	133 (24.45%)	231 (15.51%)	1.41 [1.06, 1.88]
eGFR decline of 30% or more (days 180-729)	122 (22.43%)	252 (16.92%)	1.29 [1.01, 1.64]
AKI			
Composite outcome (days 90-179)	73 (5.43%)	142 (2.31%)	1.66 [1.14, 2.40]
Composite outcome (days 180-729)	118 (8.77%)	215 (3.49%)	1.75 [1.55, 1.97]
eGFR decline of 40% or more (days 90-179)	50 (3.72%)	101 (1.64%)	1.63 [1.09, 2.43]
eGFR decline of 40% or more (days 180-729)	92 (6.84%)	148 (2.41%)	1.90 [1.49, 2.41]
eGFR decline of 30% or more (days 90-179)	70 (5.2%)	142 (2.31%)	1.59 [1.11, 2.26]
eGFR decline of 30% or more (days 180-729)	117 (8.7%)	215 (3.49%)	1.72 [1.50, 1.98]
CKD 2+ (days 28-729)	42 (3.12%)	108 (1.76%)	1.54 [1.24, 1.90]

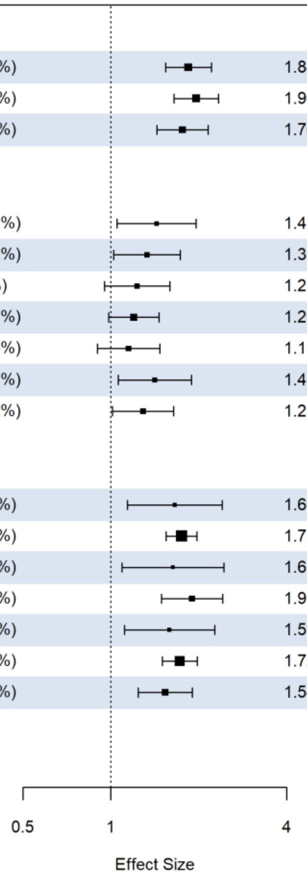

eAppendix 10. Sensitivity Analysis for Subgroups With Different Age Group

We conducted sensitivity analyses on both cohorts stratified by the cohort entry age (i.e, under 5, 5 to 12, and 12-21). We performed the same PS stratification procedure and used Cox proportional hazard model to estimate the hazard ratio.

A. Empirical equipoise assessment

Supplement Figure 115: Preference score distributions of COVID-19 positive and negative groups for children under 5 with AKI. A greater convergence of these distributions indicates a higher similarity in the predicted likelihood of being infected between the COVID-19 positive (red) and negative (blue) participants.

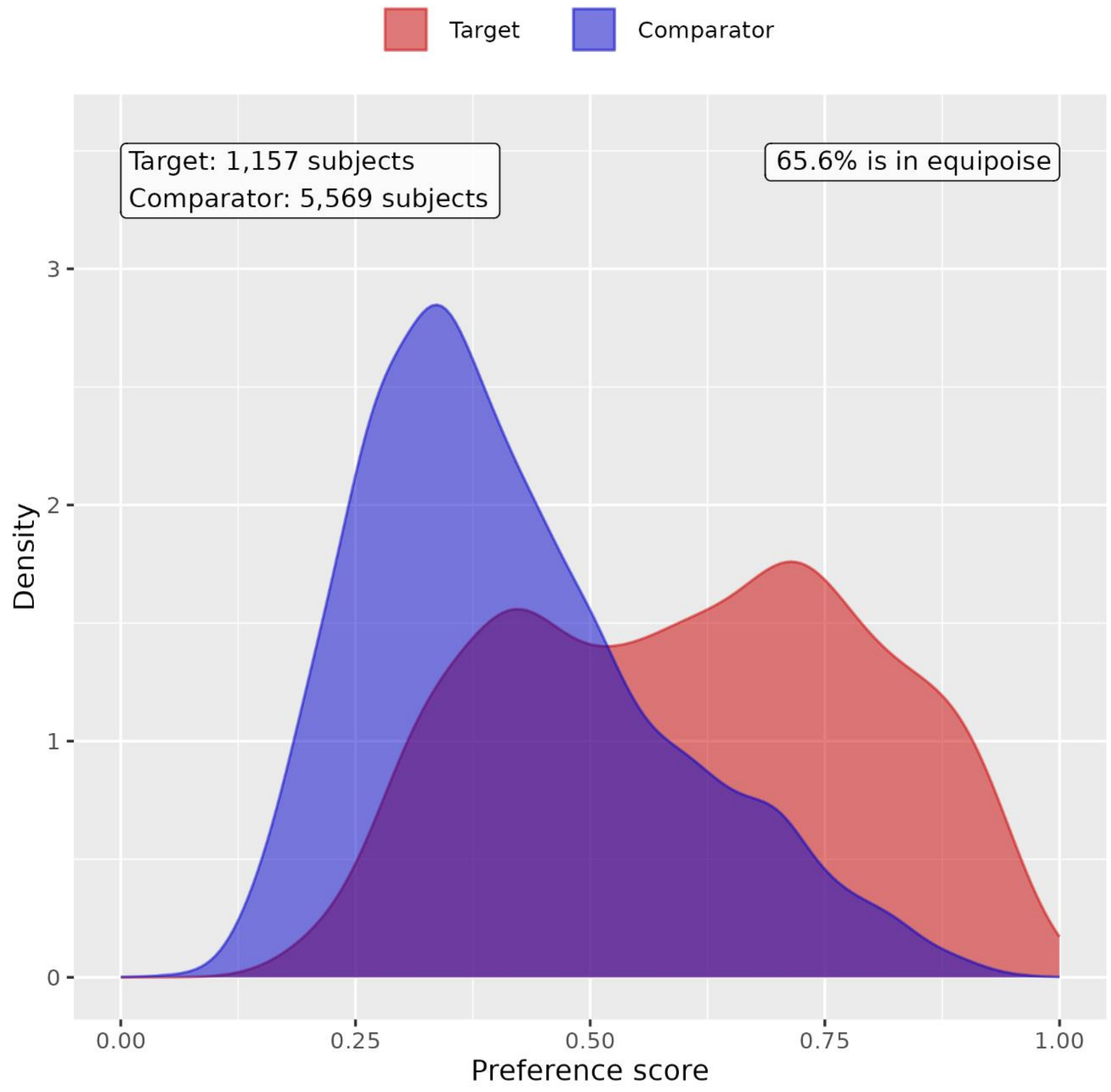

Supplement Figure 116: Preference score distributions of COVID-19 positive and negative groups for children within 5 to 12 with AKI. A greater convergence of these distributions indicates a higher similarity in the predicted likelihood of being infected between the COVID-19 positive (red) and negative (blue) participants.

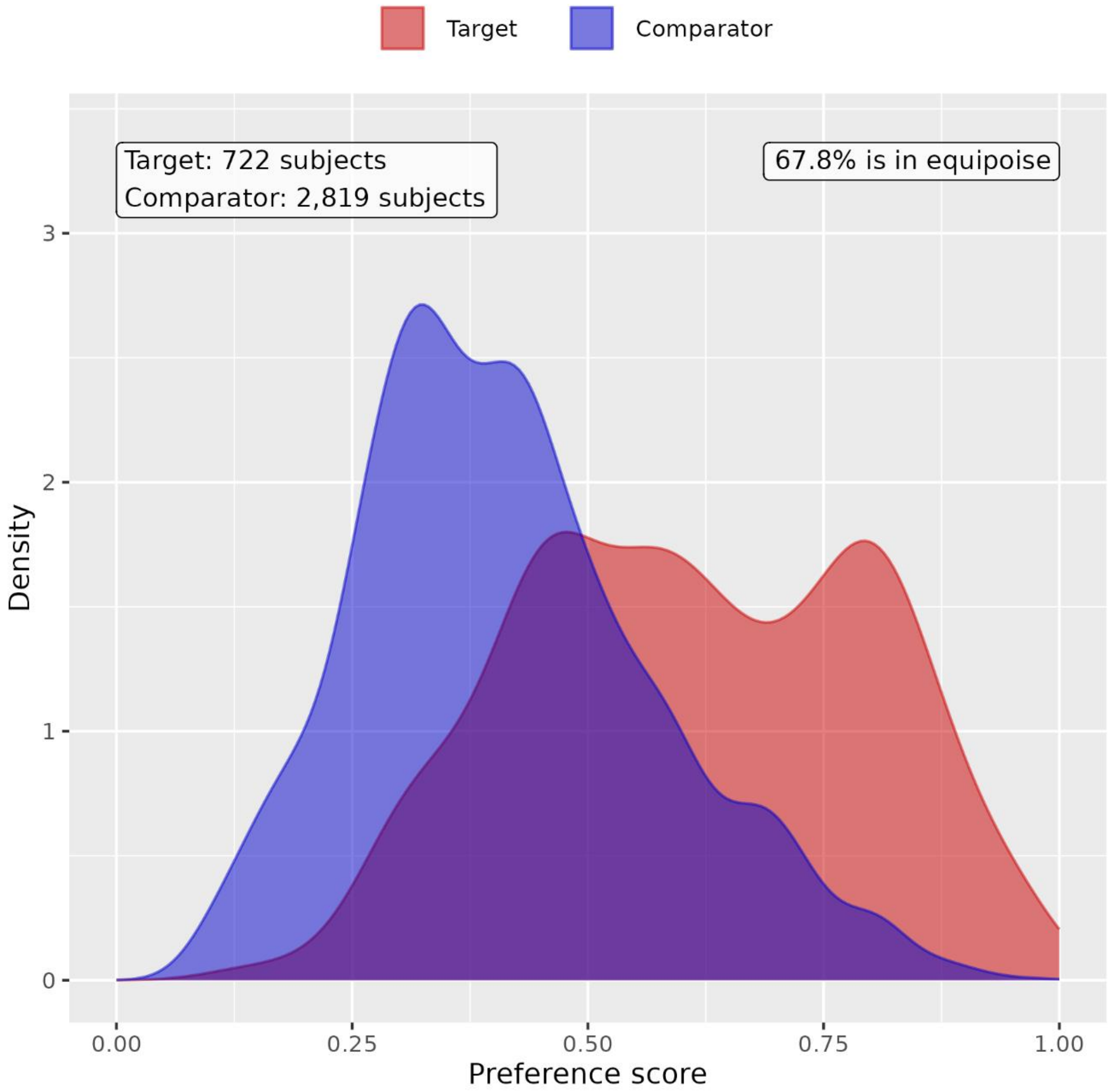

Supplement Figure 117: Preference score distributions of COVID-19 positive and negative groups for adolescents within 12 to 21 with AKI. A greater convergence of these distributions indicates a higher similarity in the predicted likelihood of being infected between the COVID-19 positive (red) and negative (blue) participants.

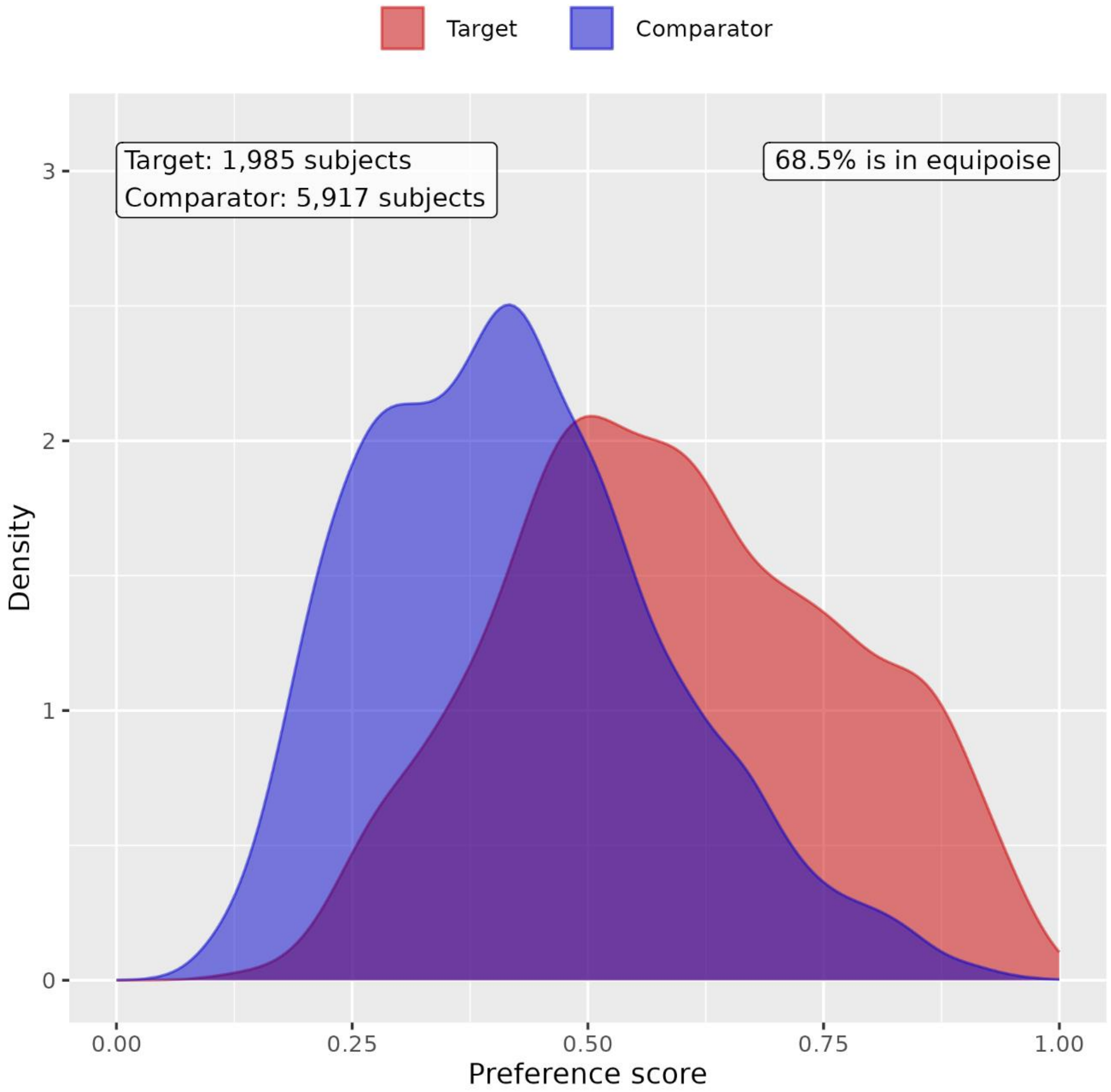

Supplement Figure 118: Preference score distributions of COVID-19 positive and negative groups for children under 5 with CKD. A greater convergence of these distributions indicates a higher similarity in the predicted likelihood of being infected between the COVID-19 positive (red) and negative (blue) participants.

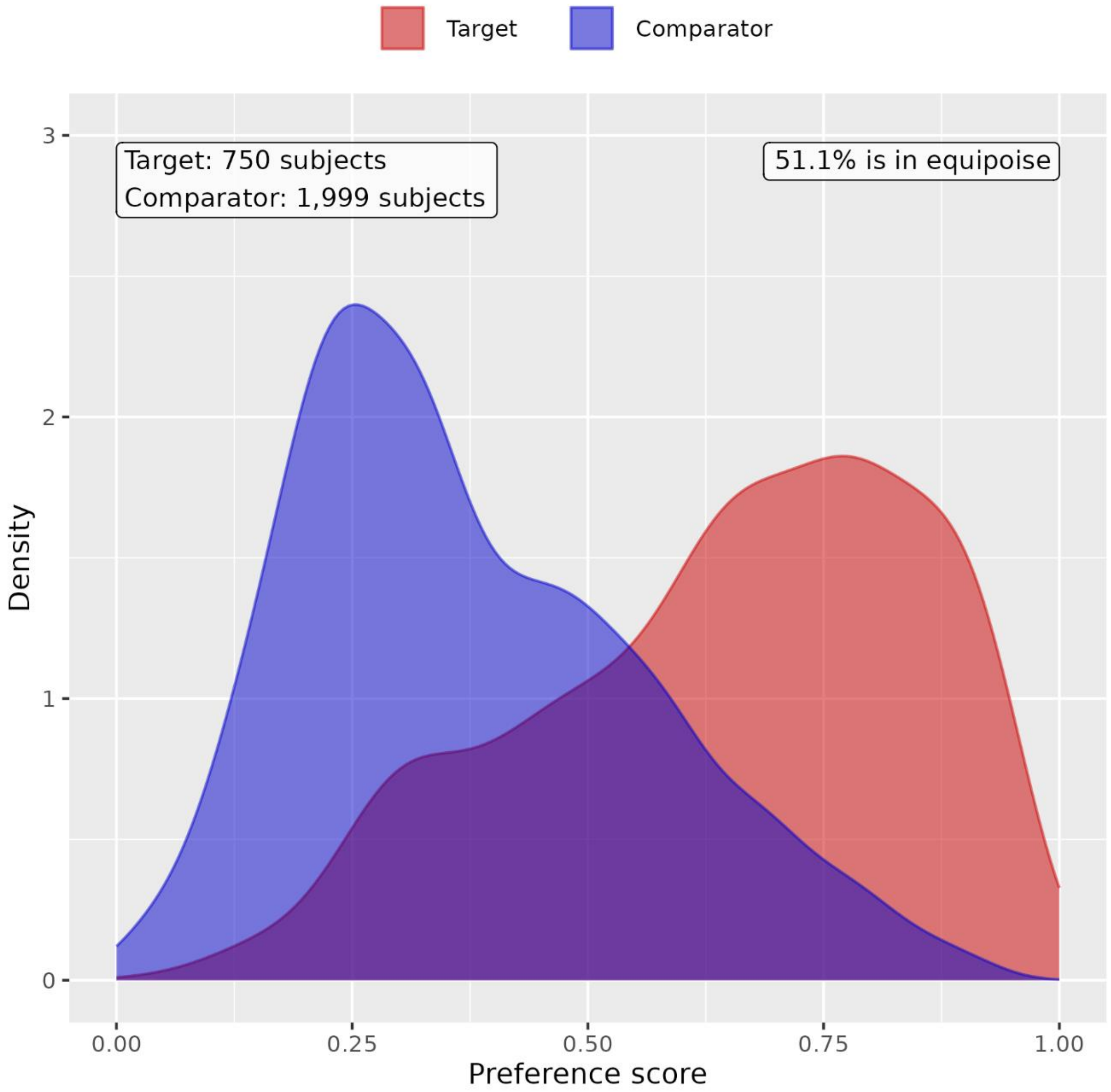

Supplement Figure 119: Preference score distributions of COVID-19 positive and negative groups for children within 5 to 12 with CKD. A greater convergence of these distributions indicates a higher similarity in the predicted likelihood of being infected between the COVID-19 positive (red) and negative (blue) participants.

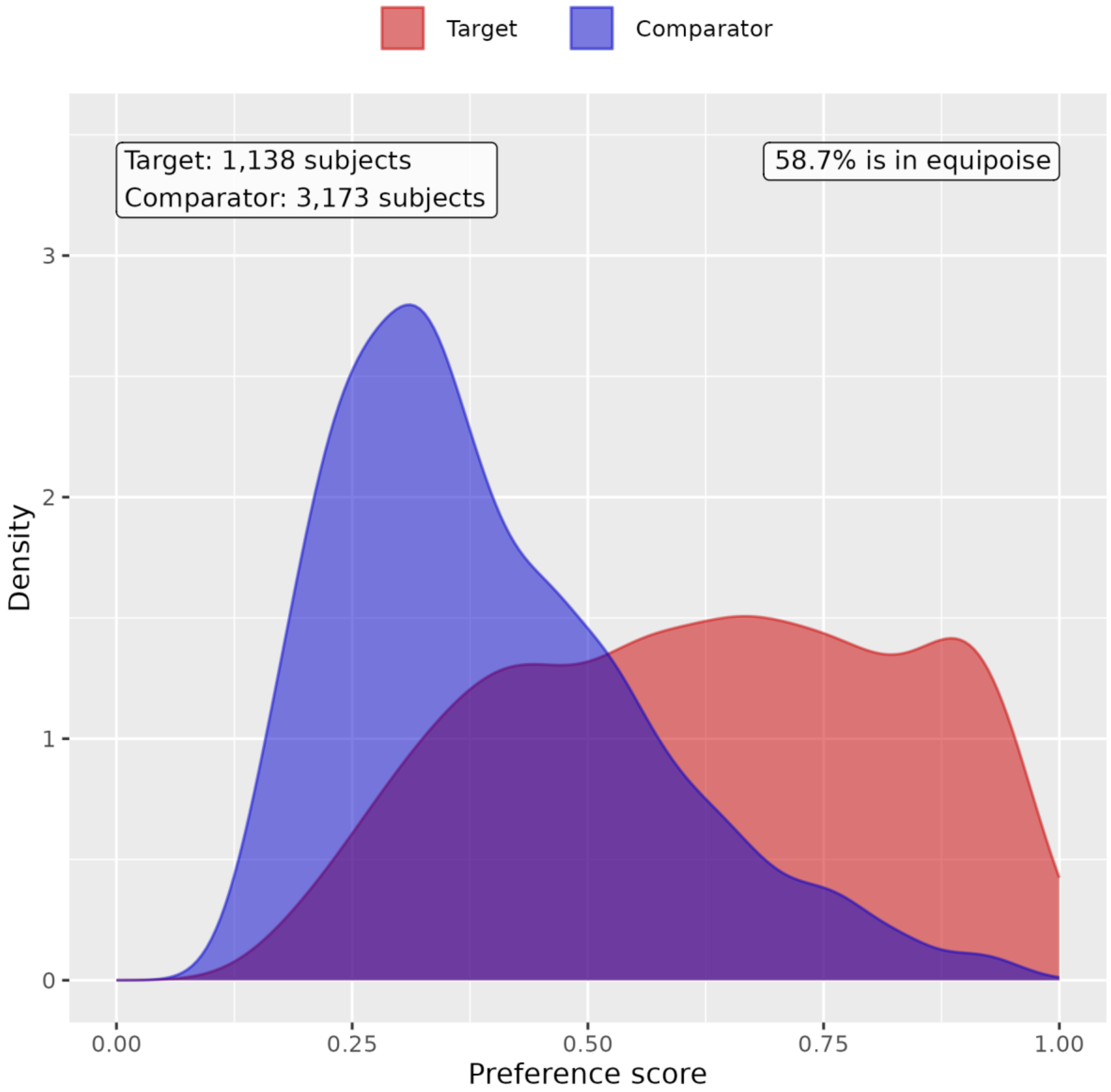

Supplement Figure 120: Preference score distributions of COVID-19 positive and control groups for adolescent within 12 to 21 with CKD. A greater convergence of these distributions indicates a higher similarity in the predicted likelihood of being infected between the COVID-19 positive (red) and negative (blue) participants.

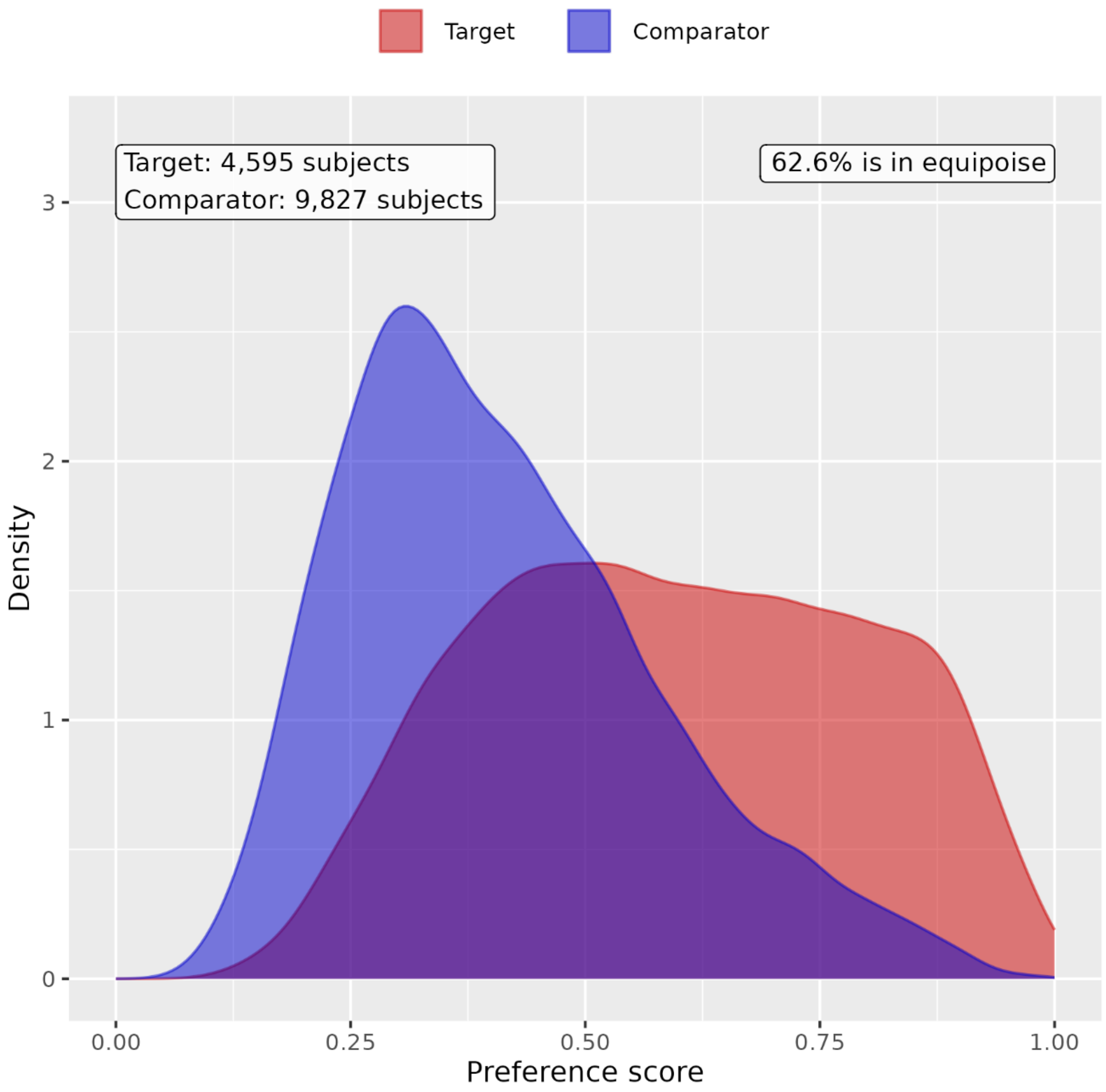

Supplement Figure 121: Preference score distributions of COVID-19 positive and negative groups for children under 5 with no AKI or CKD. A greater convergence of these distributions indicates a higher similarity in the predicted likelihood of being infected between the COVID-19 positive (red) and negative (blue) participants.

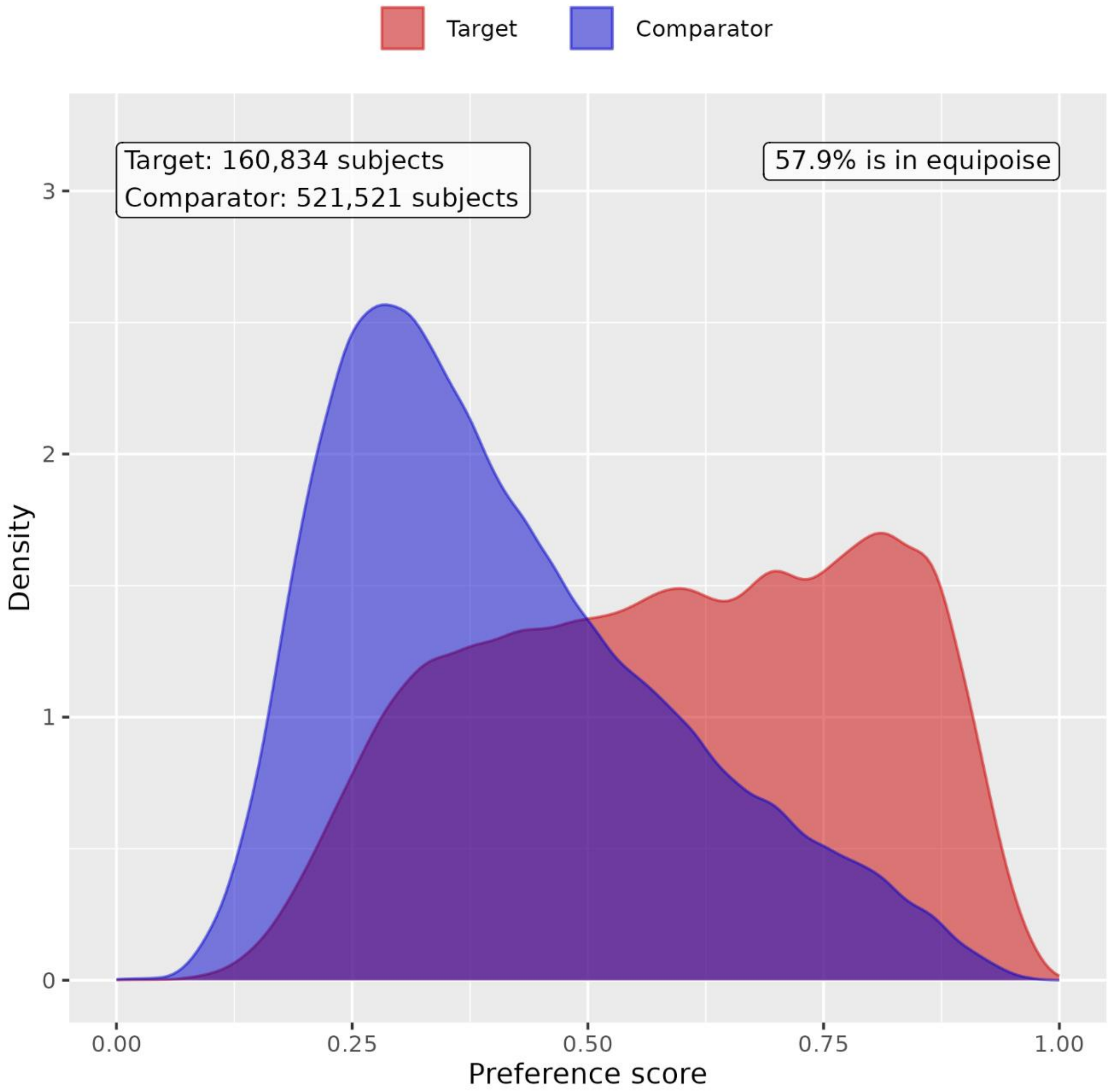

Supplement Figure 122: Preference score distributions of COVID-19 positive and negative groups for children within 5 to 12 with no AKI or CKD. A greater convergence of these distributions indicates a higher similarity in the predicted likelihood of being infected between the COVID-19 positive (red) and negative (blue) participants.

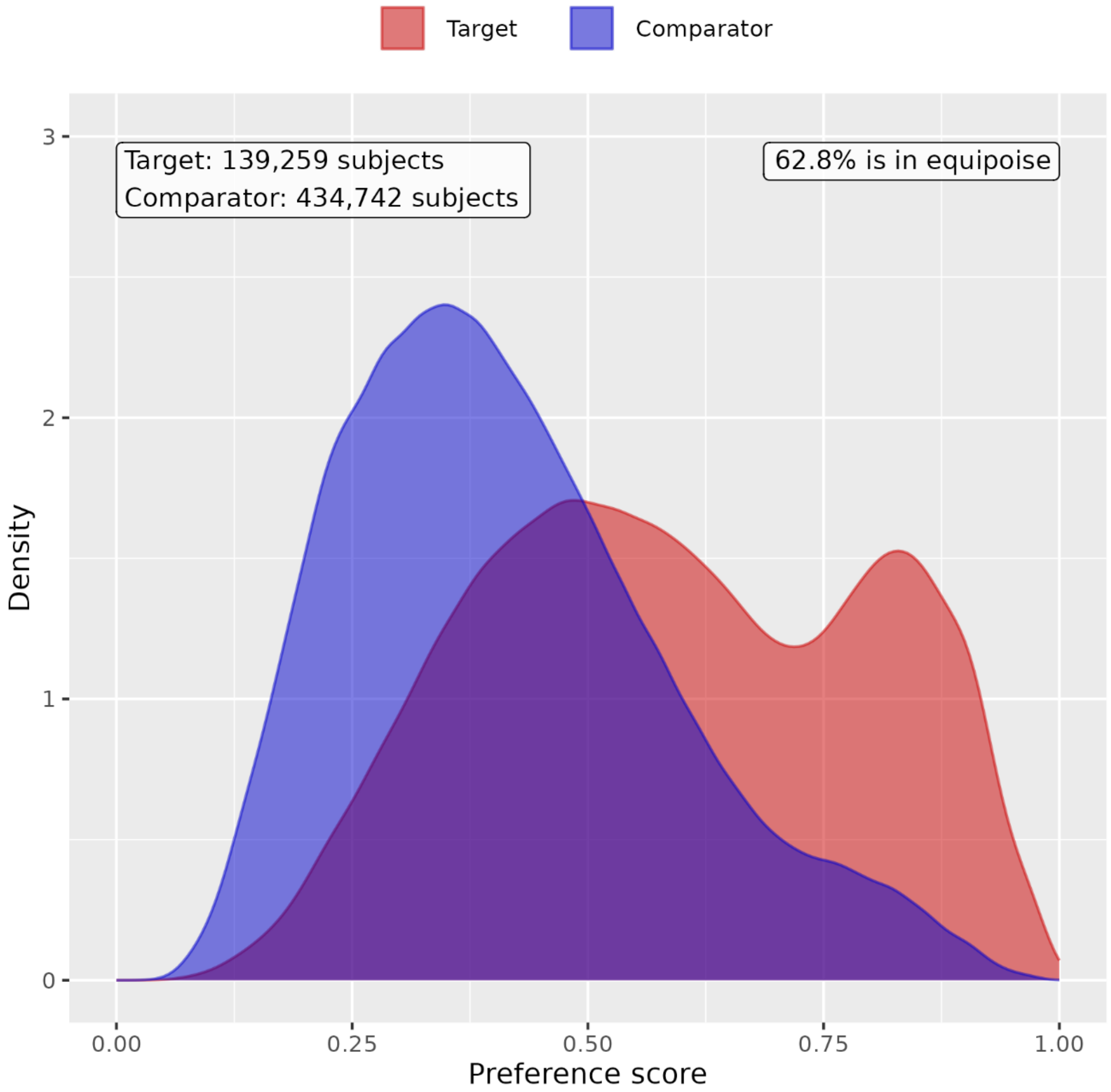

Supplement Figure 123: Preference score distributions of COVID-19 positive and control groups for adolescents within 12 to 21 with no AKI or CKD. A greater convergence of these distributions indicates a higher similarity in the predicted likelihood of being infected between the COVID-19 positive (red) and negative (blue) participants.

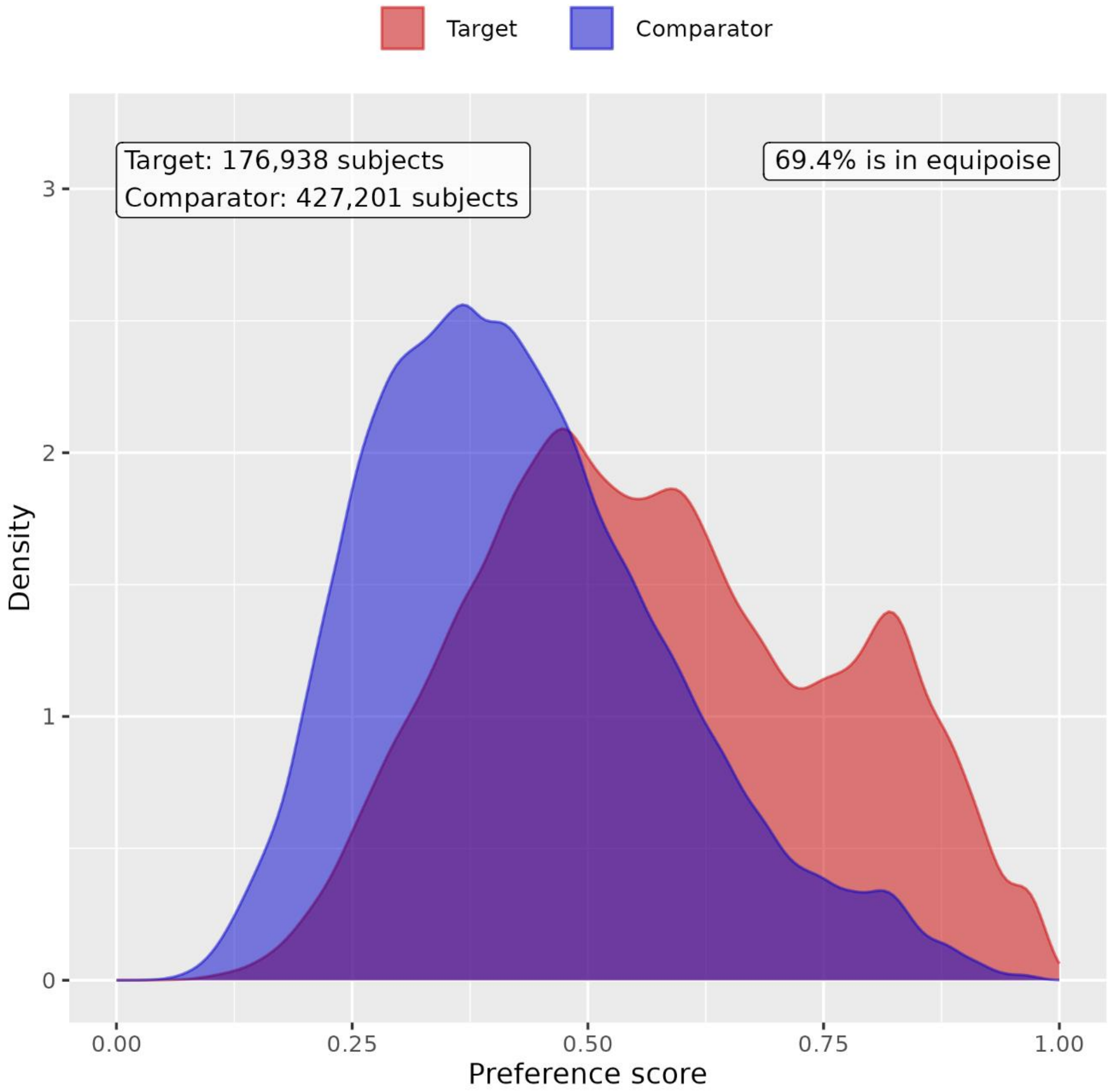

B. Patient characteristic balance

Supplement Figure 124: Patient characteristic balance before and after large-scale PS stratification with 6 strata for children under 5 with AKI. The upper panel displays the top 20 covariates with the largest standardized difference of means before stratification, while the lower panel displays the top 20 covariates with the largest standardized difference of means after stratification.

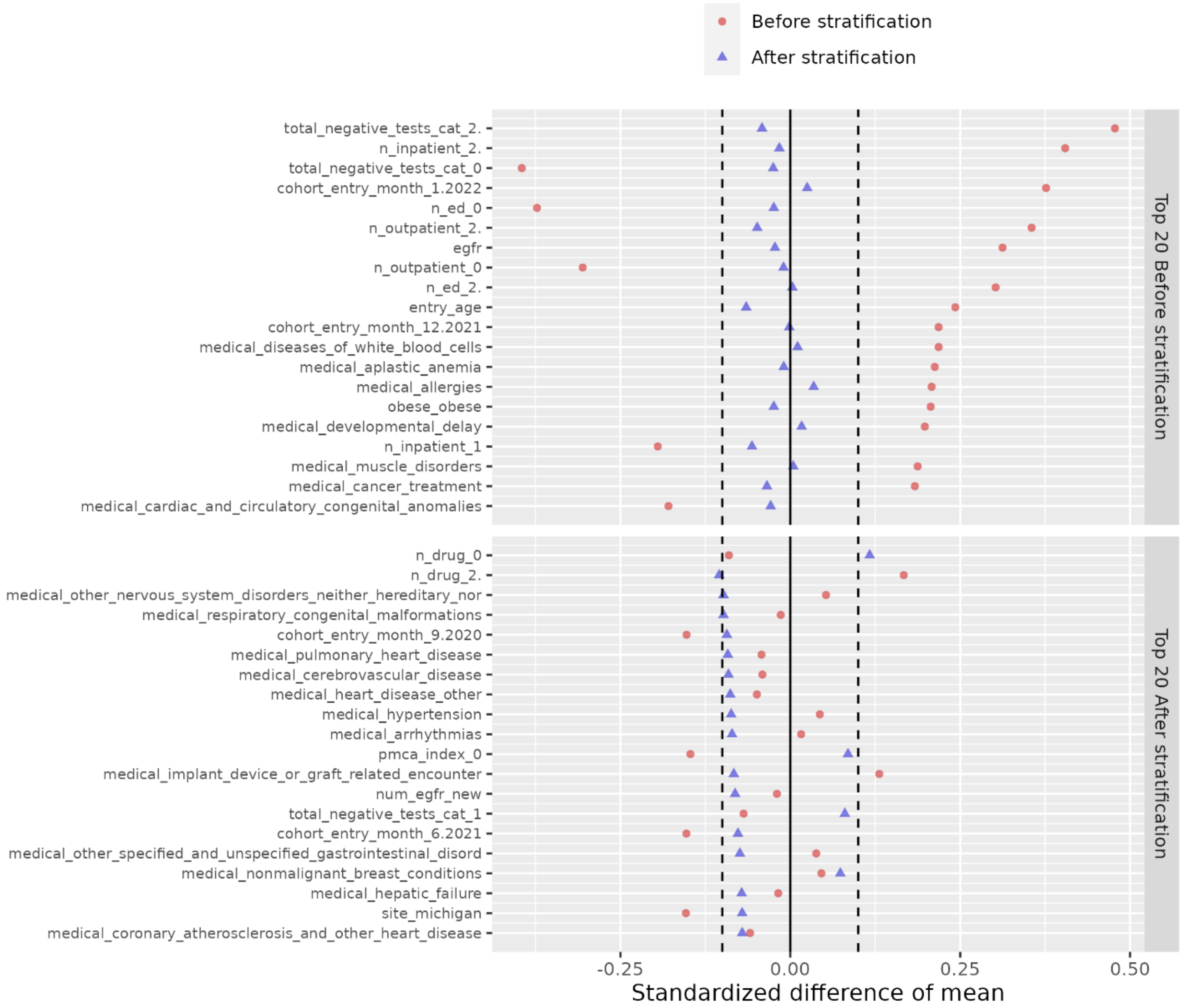

Supplement Figure 125: Patient characteristic balance before and after large-scale PS stratification with 6 strata for children within 5 to 12 with AKI. The upper panel displays the top 20 covariates with the largest standardized difference of means before stratification, while the lower panel displays the top 20 covariates with the largest standardized difference of means after stratification.

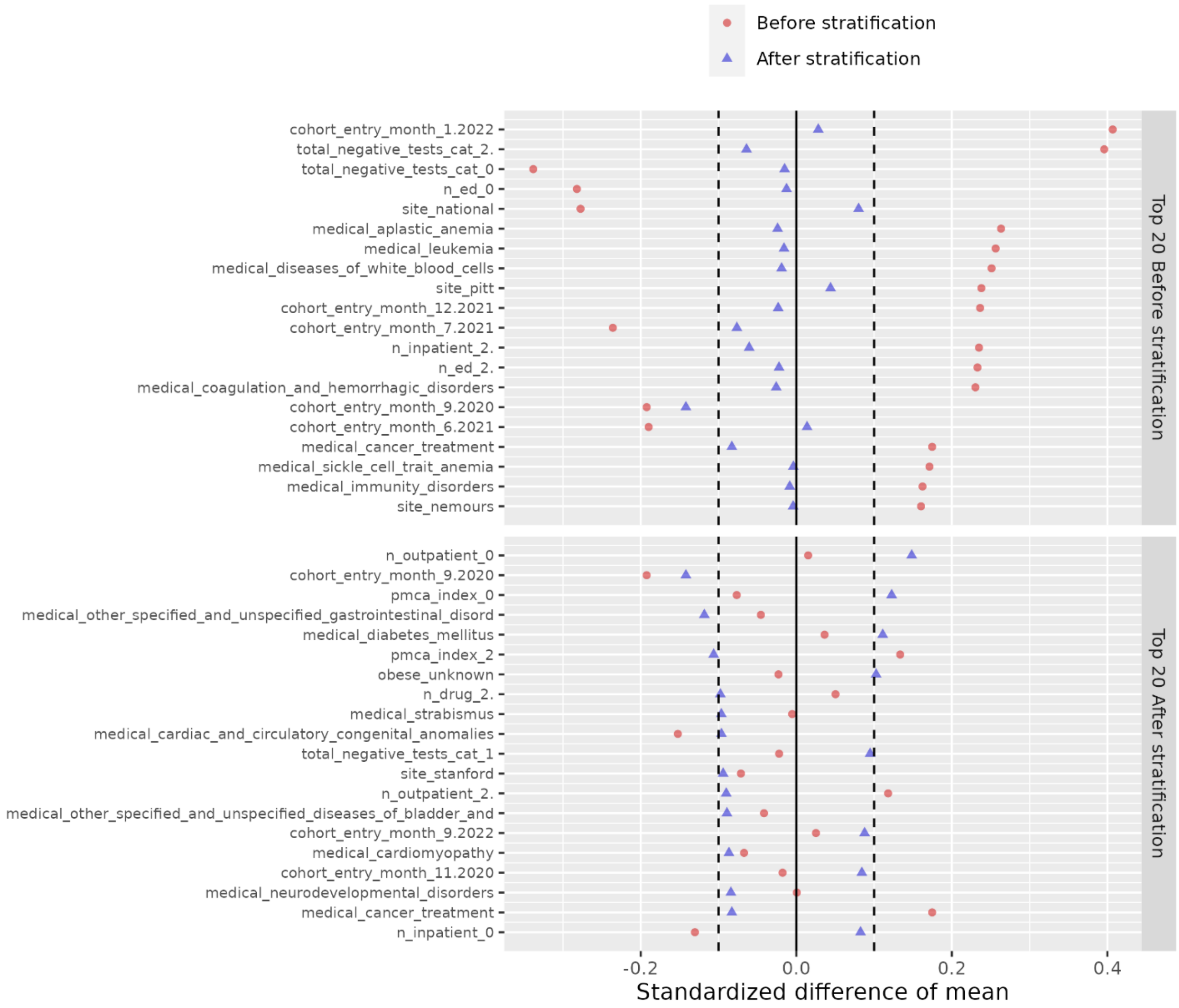

Supplement Figure 126: Patient characteristic balance before and after large-scale PS stratification with 6 strata for adolescent within 12 to 21 with AKI. The upper panel displays the top 20 covariates with the largest standardized difference of means before stratification, while the lower panel displays the top 20 covariates with the largest standardized difference of means after stratification.

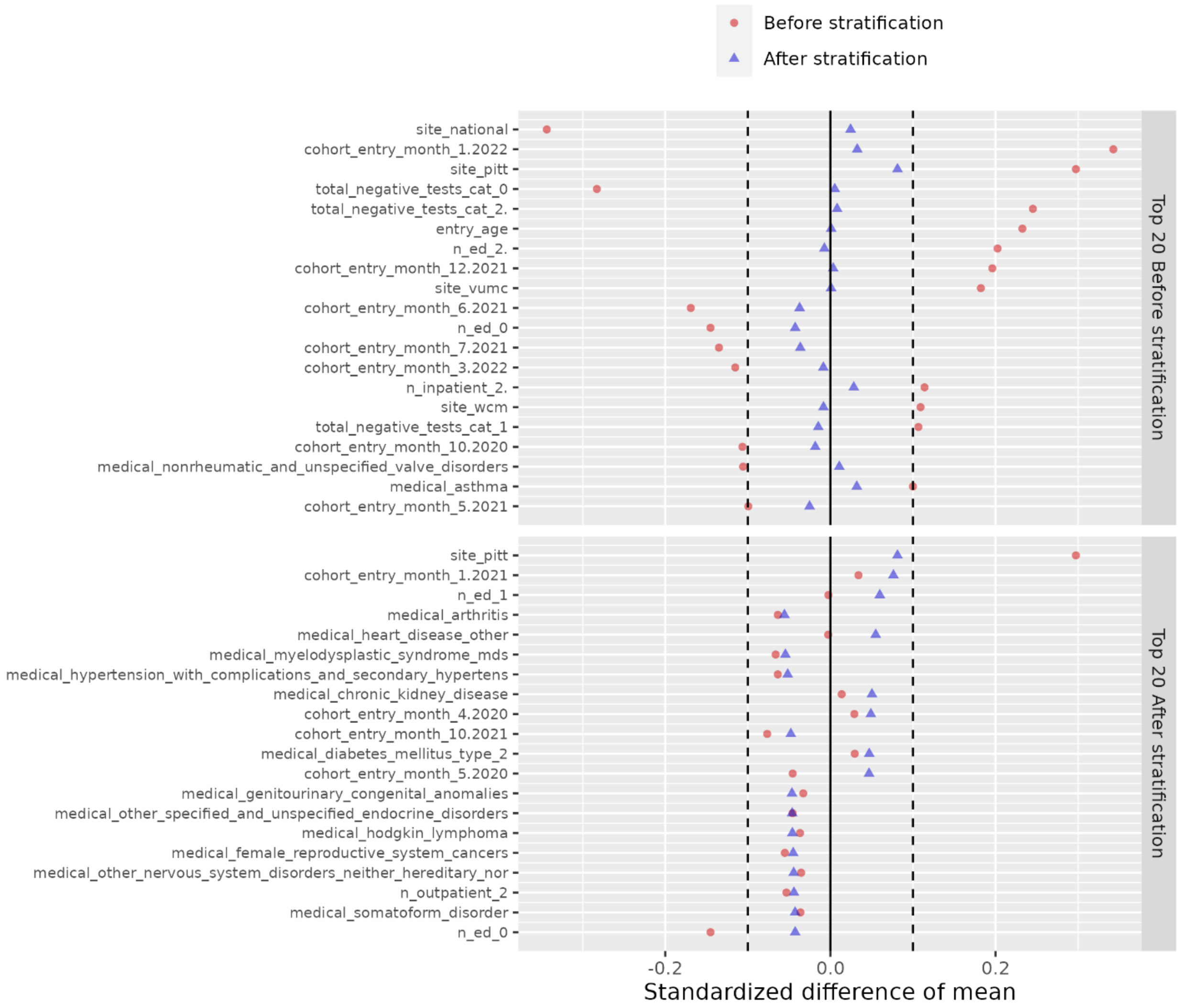

Supplement Figure 127: Patient characteristic balance before and after large-scale PS stratification with 6 strata for children under 5 with CKD. The upper panel displays the top 20 covariates with the largest standardized difference of means before stratification, while the lower panel displays the top 20 covariates with the largest standardized difference of means after stratification.

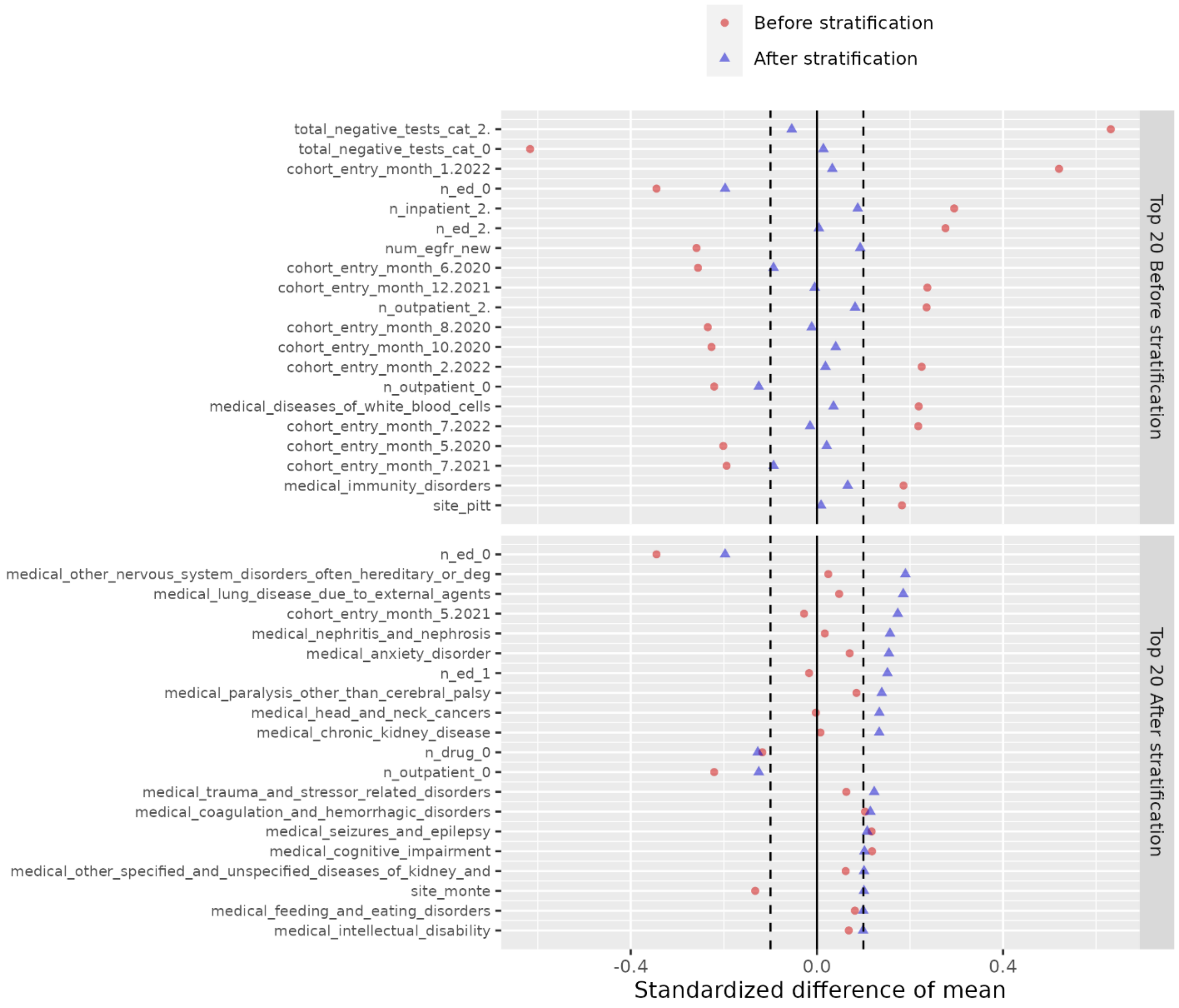

Supplement Figure 128: Patient characteristic balance before and after large-scale PS stratification with 6 strata for children within 5 to 12 with CKD. The upper panel displays the top 20 covariates with the largest standardized difference of means before stratification, while the lower panel displays the top 20 covariates with the largest standardized difference of means after stratification.

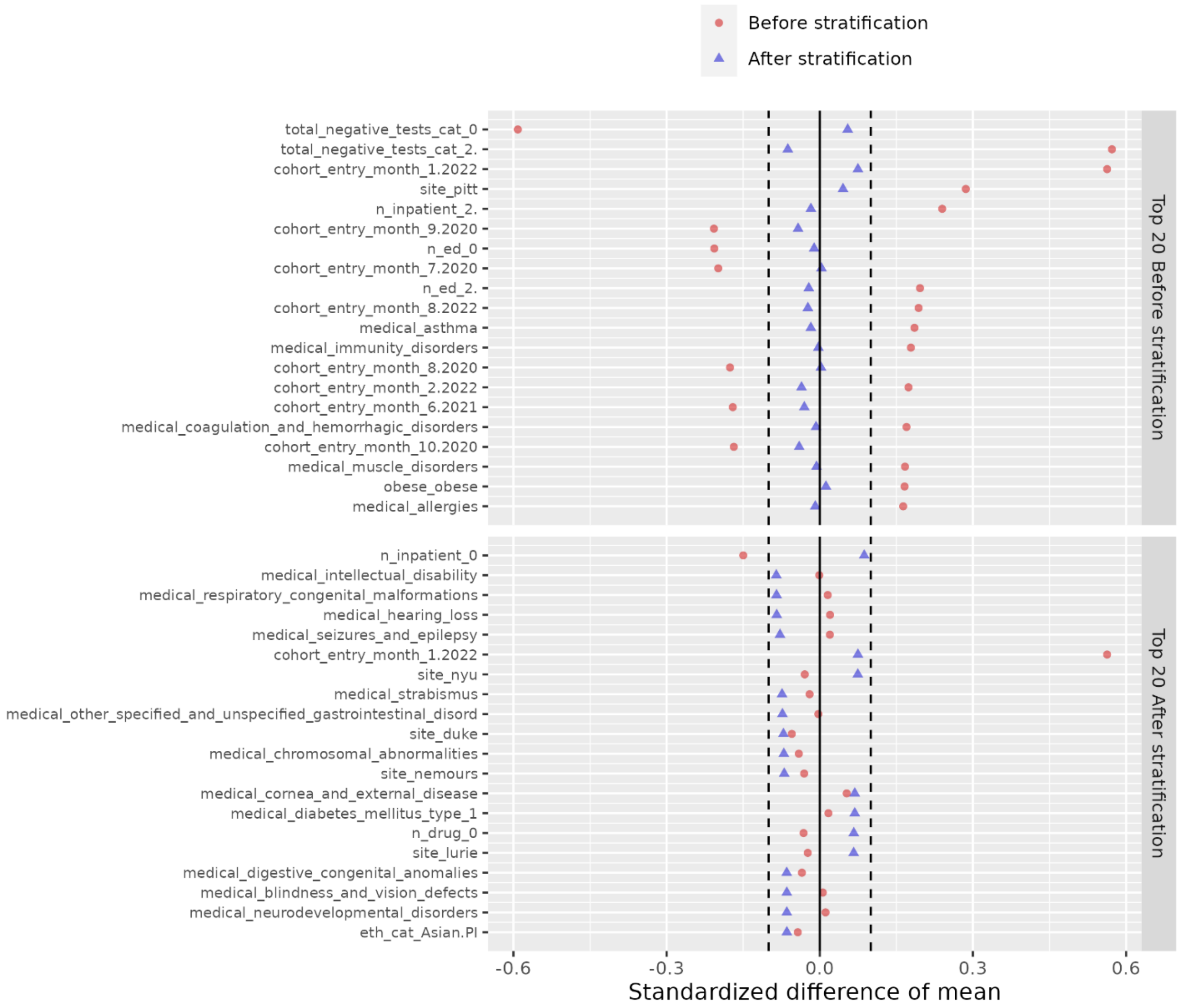

Supplement Figure 129: Patient characteristic balance before and after large-scale PS stratification with 6 strata for adolescent within 12 to 21 with CKD. The upper panel displays the top 20 covariates with the largest standardized difference of means before stratification, while the lower panel displays the top 20 covariates with the largest standardized difference of means after stratification.

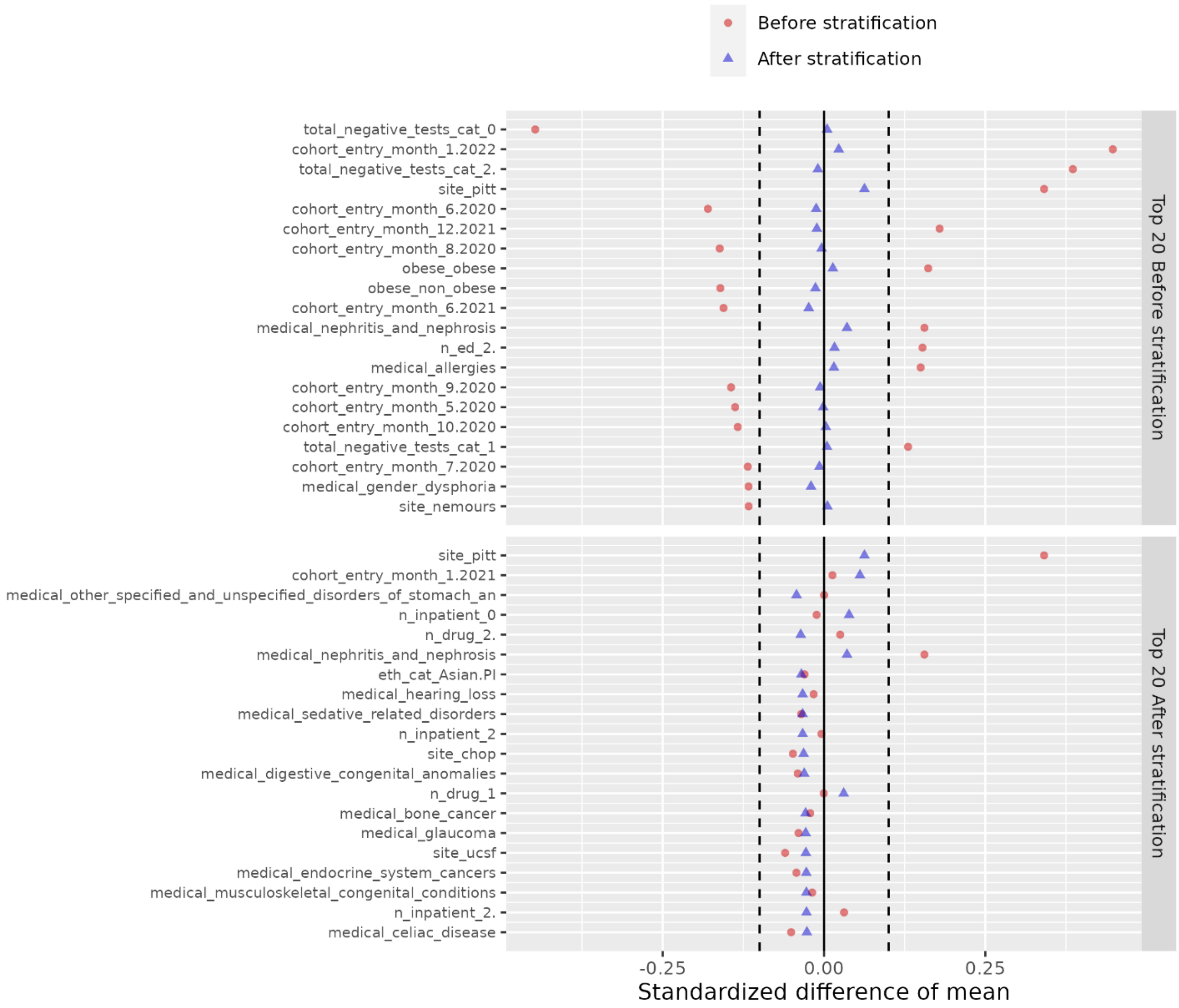

Supplement Figure 130: Patient characteristic balance before and after large-scale PS stratification with 6 strata for children under 5 with no AKI or CKD. The upper panel displays the top 20 covariates with the largest standardized difference of means before stratification, while the lower panel displays the top 20 covariates with the largest standardized difference of means after stratification.

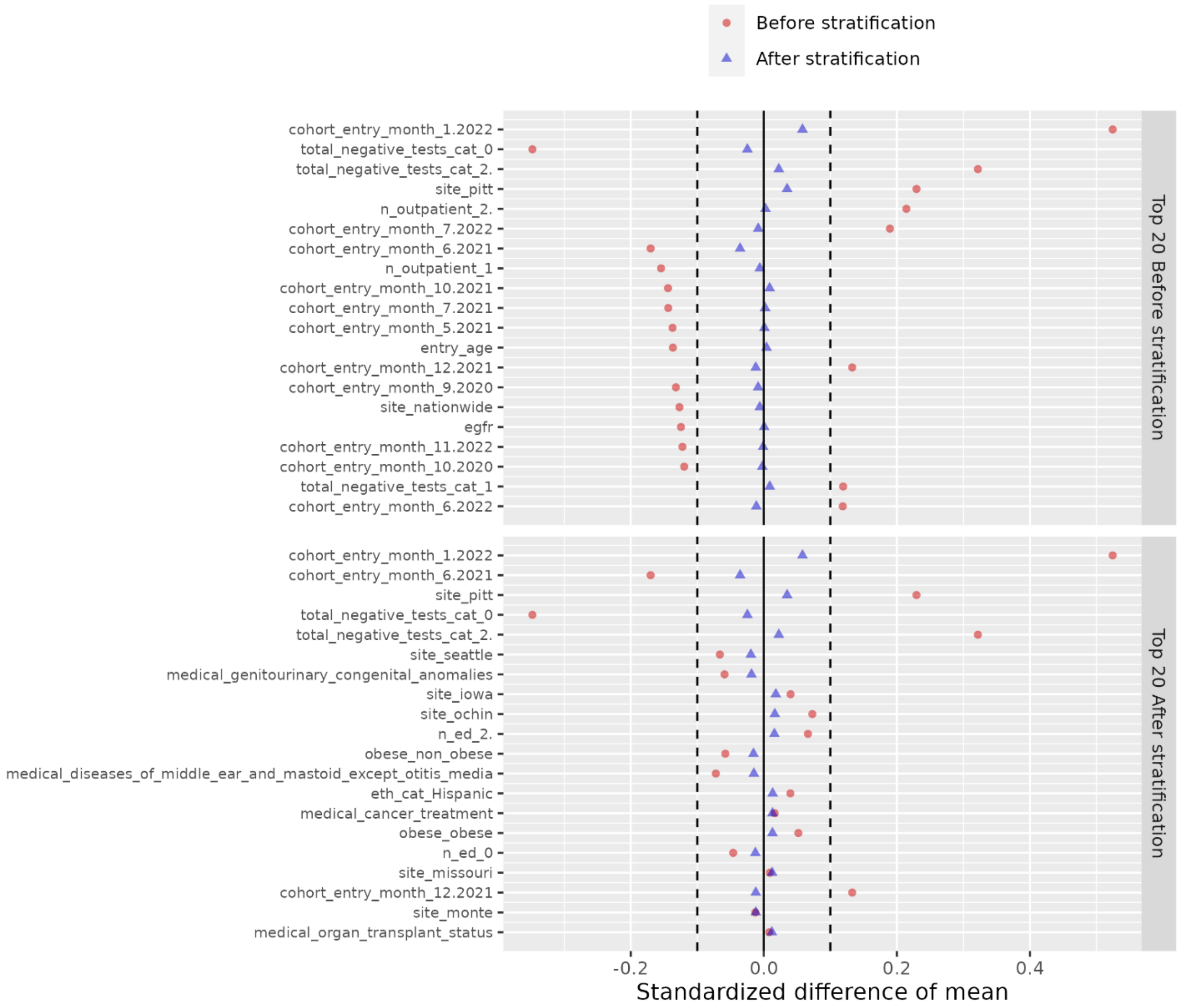

Supplement Figure 131: Patient characteristic balance before and after large-scale PS stratification with 6 strata for hospitalized children within 5 to 12 with no AKI or CKD. The upper panel displays the top 20 covariates with the largest standardized difference of means before stratification, while the lower panel displays the top 20 covariates with the largest standardized difference of means after stratification.

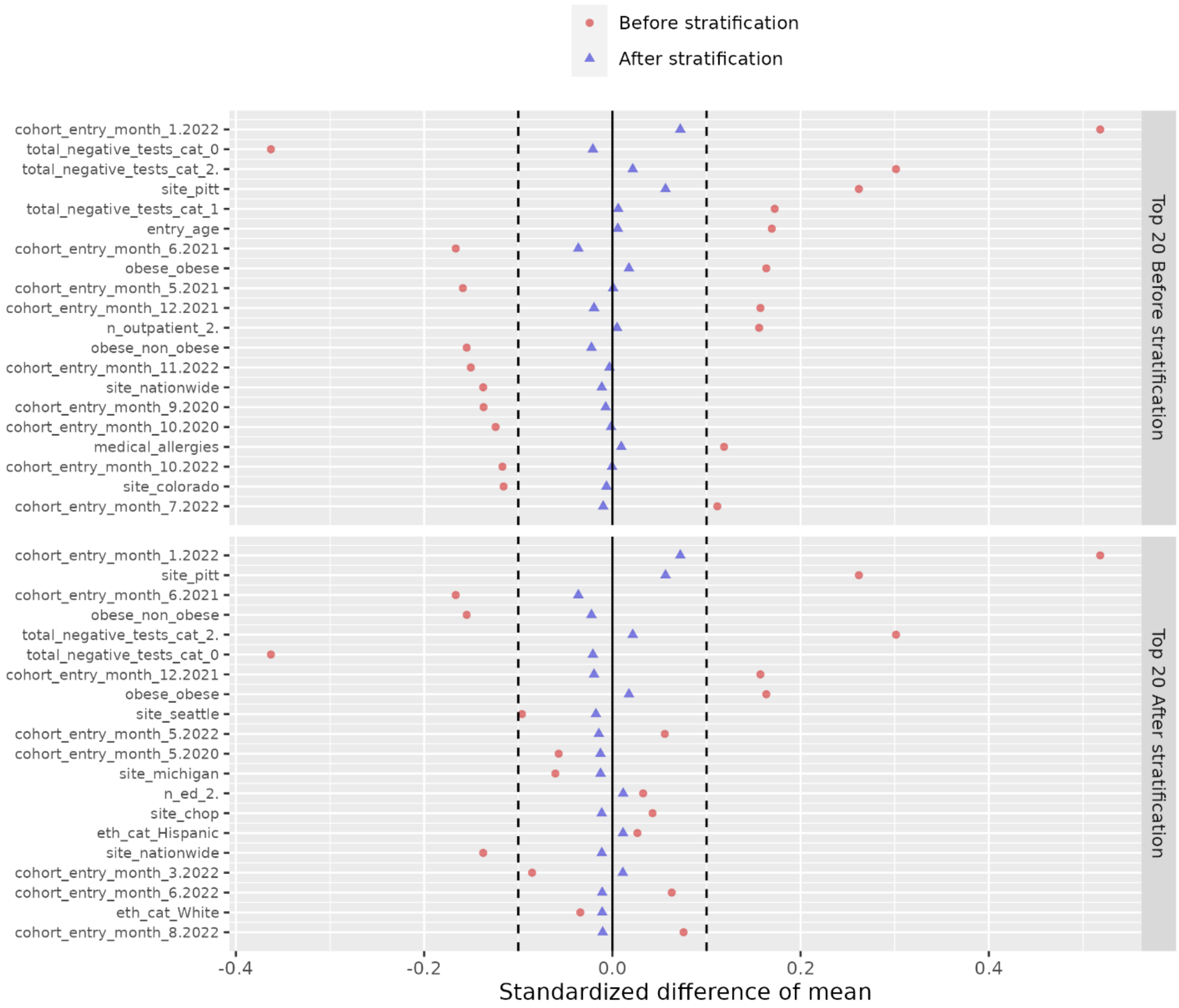

Supplement Figure 132: Patient characteristic balance before and after large-scale PS stratification with 6 strata for adolescents within 12 to 21 with no AKI or CKD. The upper panel displays the top 20 covariates with the largest standardized difference of means before stratification, while the lower panel displays the top 20 covariates with the largest standardized difference of means after stratification.

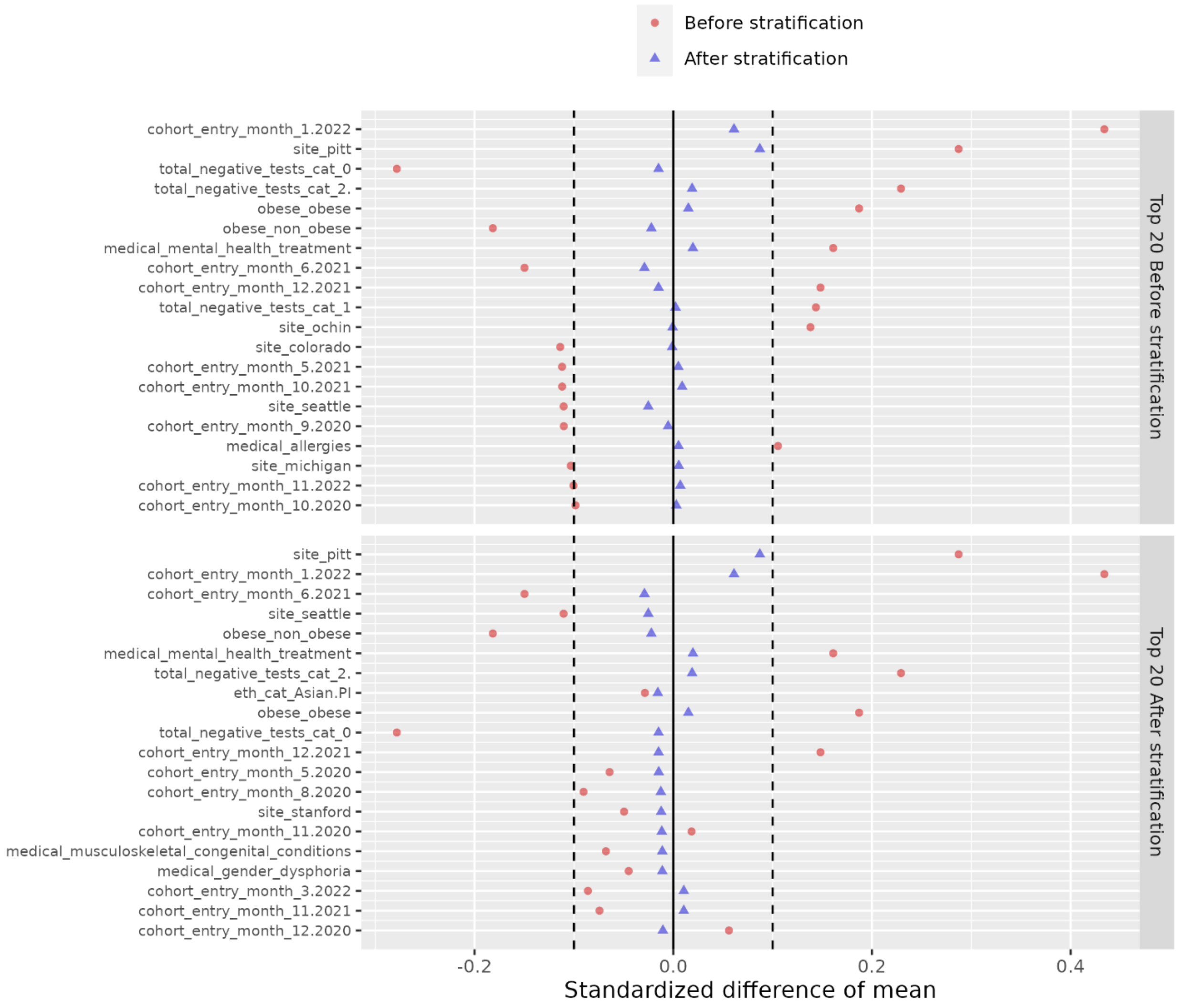

C. Hazard ratio of COVID-19 positive group compared to control group

Supplement Table 20: Estimated hazard ratio in kidney function outcomes between the COVID-19 positive cohort and the control cohort for children under 5.

Age: Below 5	COVID-19 Groups Event (%)	Control Group Event (%)	HR (95% CI)
No CKD or AKI			
CKD 2+ (days 28-729)	271 (0.17%)	745 (0.14%)	1.30 [1.07, 1.58]
CKD 2+ (majority not returned to 90 and above) (days 28-729)	223 (0.14%)	580 (0.11%)	1.36 [1.04, 1.78]
CKD 2+ (not returned to 90 and above) (days 28-729)	195 (0.12%)	505 (0.1%)	1.34 [1.05, 1.71]
CKD 3+ (days 28-729)	66 (0.04%)	148 (0.03%)	1.56 [1.07, 2.28]
CKD 3+ (majority not returned to 90 and above) (days 28-729)	41 (0.03%)	109 (0.02%)	1.32 [0.98, 1.79]
CKD 3+ (not returned to 90 and above) (days 28-729)	51 (0.03%)	129 (0.02%)	1.29 [0.91, 1.83]
CKD 3+ (not returned to 60 and above) (days 28-729)	37 (0.02%)	95 (0.02%)	1.34 [0.93, 1.94]
CKD			
Composite outcome (days 28-179)	108 (14.4%)	211 (10.56%)	1.44 [1.19, 1.75]
Composite outcome (days 180-729)	123 (16.4%)	276 (13.81%)	1.16 [0.99, 1.35]
eGFR decline of 50% or more (days 28-179)	41 (5.47%)	79 (3.95%)	1.41 [1.10, 1.81]
eGFR decline of 50% or more (days 180-729)	43 (5.73%)	92 (4.6%)	1.28 [0.88, 1.87]
eGFR decline of 40% or more (days 28-179)	77 (10.27%)	146 (7.3%)	1.38 [1.10, 1.75]
eGFR decline of 40% or more (days 180-729)	69 (9.2%)	177 (8.85%)	1.11 [0.89, 1.37]
eGFR decline of 30% or more (days 28-179)	108 (14.4%)	211 (10.56%)	1.44 [1.19, 1.75]
eGFR decline of 30% or more (days 180-729)	121 (16.13%)	276 (13.81%)	1.14 [0.97, 1.34]
AKI			
Composite outcome (days 180-729)	122 (10.54%)	238 (4.27%)	1.74 [1.58, 1.91]
eGFR decline of 50% or more (days 180-729)	85 (7.35%)	135 (2.42%)	1.88 [1.70, 2.09]
eGFR decline of 40% or more (days 180-729)	104 (8.99%)	190 (3.41%)	1.84 [1.71, 1.97]
eGFR decline of 30% or more (days 180-729)	122 (10.54%)	238 (4.27%)	1.74 [1.58, 1.91]

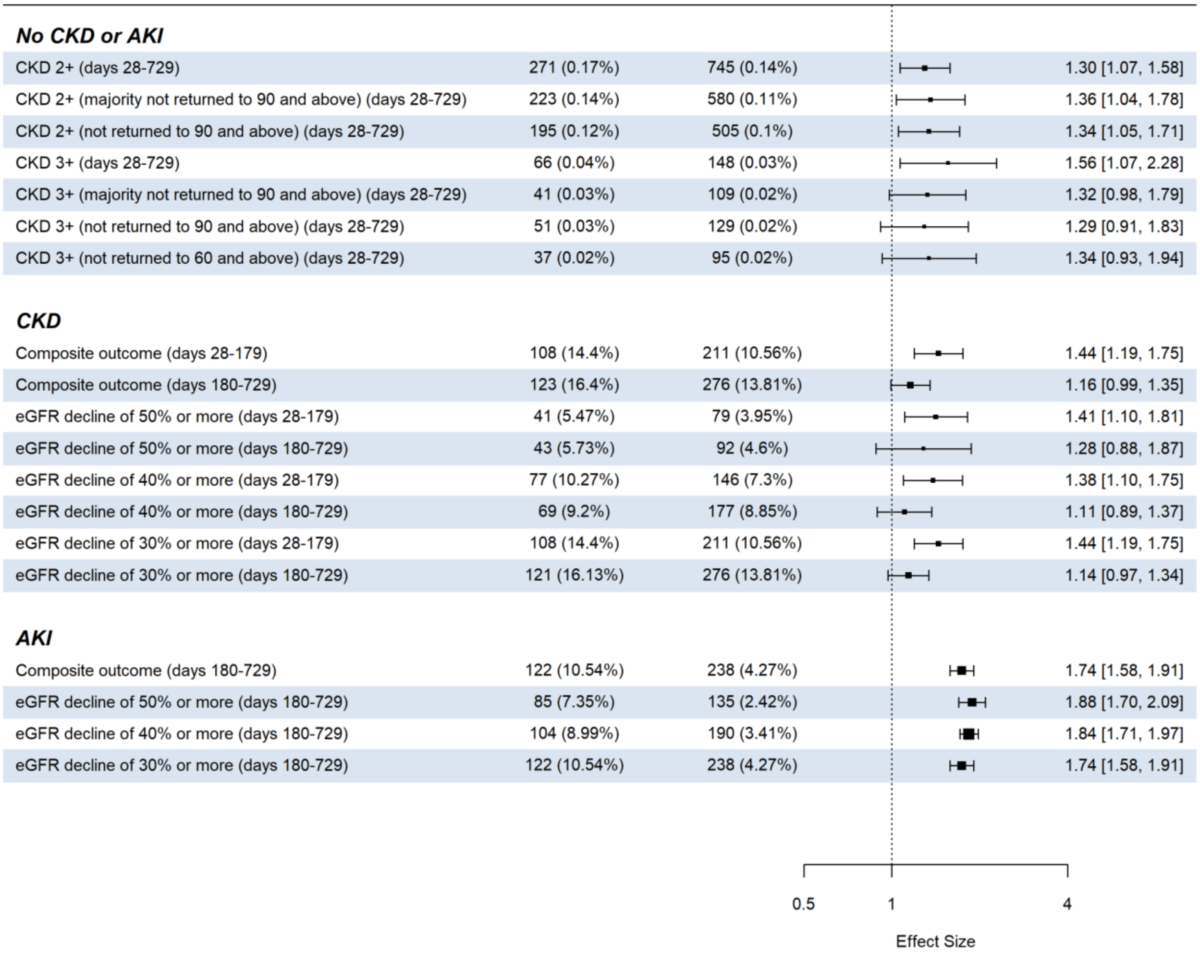

Supplement Table 21: Estimated hazard ratio in kidney function outcomes between the COVID-19 positive cohort and the control cohort for hospitalized children within 5 to 12

Age: 5 - 12	COVID-19 Groups Event (%)	Control Group Event (%)	HR (95% CI)
No CKD or AKI			
CKD 2+ (days 28-729)	260 (0.19%)	806 (0.19%)	1.05 [0.93, 1.18]
CKD 2+ (majority not returned to 90 and above) (days 28-729)	217 (0.16%)	656 (0.15%)	1.06 [0.88, 1.27]
CKD 2+ (not returned to 90 and above) (days 28-729)	204 (0.15%)	569 (0.13%)	1.13 [0.94, 1.36]
CKD 3+ (days 28-729)	42 (0.03%)	94 (0.02%)	1.53 [1.08, 2.17]
CKD 3+ (majority not returned to 90 and above) (days 28-729)	36 (0.03%)	76 (0.02%)	1.60 [1.00, 2.54]
CKD 3+ (not returned to 90 and above) (days 28-729)	38 (0.03%)	84 (0.02%)	1.52 [0.97, 2.38]
CKD 3+ (not returned to 60 and above) (days 28-729)	34 (0.02%)	69 (0.02%)	1.60 [0.92, 2.75]
CKD			
Composite outcome (days 28-179)	194 (17.05%)	409 (12.89%)	1.26 [1.06, 1.48]
Composite outcome (days 180-729)	272 (23.9%)	674 (21.24%)	1.29 [1.16, 1.44]
eGFR decline of 50% or more (days 28-179)	70 (6.15%)	175 (5.52%)	0.99 [0.86, 1.15]
eGFR decline of 50% or more (days 180-729)	107 (9.4%)	286 (9.01%)	1.07 [0.90, 1.28]
eGFR decline of 40% or more (days 28-179)	118 (10.37%)	263 (8.29%)	1.08 [0.89, 1.32]
eGFR decline of 40% or more (days 180-729)	166 (14.59%)	427 (13.46%)	1.22 [1.03, 1.43]
eGFR decline of 30% or more (days 28-179)	189 (16.61%)	409 (12.89%)	1.23 [1.03, 1.48]
eGFR decline of 30% or more (days 180-729)	269 (23.64%)	674 (21.24%)	1.28 [1.15, 1.41]

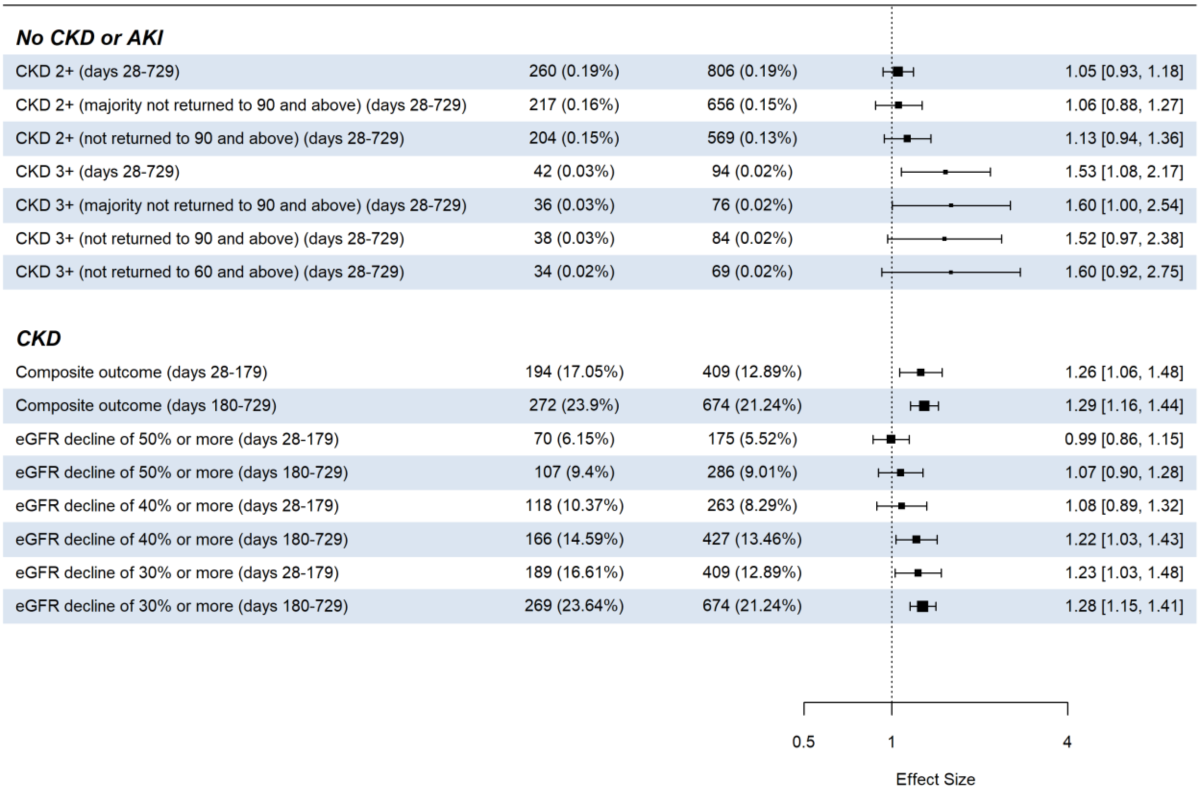

Supplement Table 22: Estimated hazard ratio in kidney function outcomes between the COVID-19 positive cohort and the control cohort for adolescents within 12 to 21

Age: Above 12	COVID-19 Groups Event (%)	Control Group Event (%)	HR (95% CI)
No CKD or AKI			
CKD 2+ (days 28-729)	1038 (0.59%)	2349 (0.55%)	1.20 [1.12, 1.28]
CKD 2+ (majority not returned to 90 and above) (days 28-729)	914 (0.52%)	2029 (0.47%)	1.19 [1.10, 1.29]
CKD 2+ (not returned to 90 and above) (days 28-729)	851 (0.48%)	1835 (0.43%)	1.20 [1.09, 1.32]
CKD 3+ (days 28-729)	24 (0.01%)	67 (0.02%)	1.00 [0.76, 1.32]
CKD 3+ (majority not returned to 90 and above) (days 28-729)	21 (0.01%)	39 (0.01%)	1.41 [1.01, 1.97]
CKD 3+ (not returned to 90 and above) (days 28-729)	22 (0.01%)	58 (0.01%)	1.05 [0.81, 1.36]
CKD 3+ (not returned to 60 and above) (days 28-729)	17 (0.01%)	36 (0.01%)	1.23 [0.90, 1.67]
CKD			
Composite outcome (days 28-179)	603 (13.12%)	1126 (11.46%)	1.11 [1.00, 1.23]
Composite outcome (days 180-729)	772 (16.8%)	1566 (15.94%)	1.09 [0.96, 1.24]
eGFR decline of 50% or more (days 28-179)	80 (1.74%)	151 (1.54%)	1.04 [0.80, 1.35]
eGFR decline of 50% or more (days 180-729)	96 (2.09%)	197 (2%)	1.22 [1.01, 1.47]
eGFR decline of 40% or more (days 28-179)	289 (6.29%)	548 (5.58%)	1.02 [0.87, 1.20]
eGFR decline of 40% or more (days 180-729)	380 (8.27%)	741 (7.54%)	1.11 [0.92, 1.34]
eGFR decline of 30% or more (days 28-179)	597 (12.99%)	1126 (11.46%)	1.09 [0.98, 1.22]
eGFR decline of 30% or more (days 180-729)	769 (16.74%)	1566 (15.94%)	1.09 [0.96, 1.24]
AKI			
Composite outcome (days 90-179)	95 (4.79%)	205 (3.46%)	1.25 [1.01, 1.56]
Composite outcome (days 180-729)	188 (9.47%)	388 (6.56%)	1.25 [0.98, 1.58]
eGFR decline of 40% or more (days 180-729)	104 (5.24%)	209 (3.53%)	1.25 [0.98, 1.59]
eGFR decline of 30% or more (days 90-179)	93 (4.69%)	205 (3.46%)	1.22 [0.97, 1.53]
eGFR decline of 30% or more (days 180-729)	186 (9.37%)	388 (6.56%)	1.23 [0.98, 1.55]
CKD 2+ (days 28-729)	109 (5.49%)	279 (4.72%)	0.99 [0.87, 1.14]
CKD 2+ (majority not returned to 90 and above) (days 28-729)	97 (4.89%)	252 (4.26%)	0.95 [0.84, 1.08]
CKD 2+ (not returned to 90 and above) (days 28-729)	88 (4.43%)	236 (3.99%)	0.90 [0.87, 0.94]

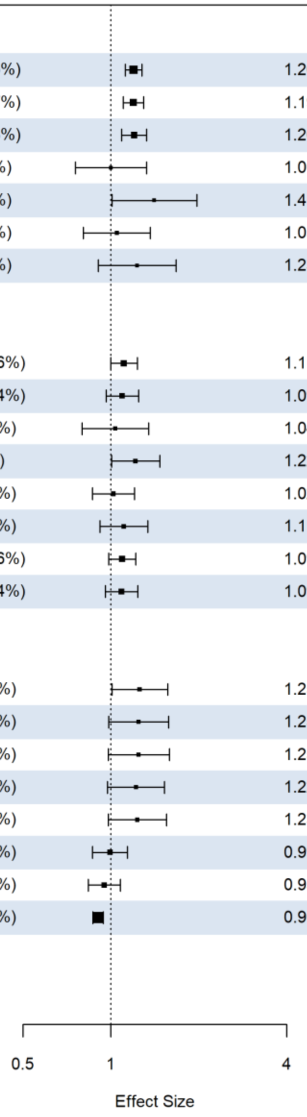

eAppendix 11. Sensitivity Analysis for AKI Subgroup Without Excluding Patients With Dialysis During Acute Phase

We conducted sensitivity analyses for the AKI subgroup without excluding the patients who had dialysis during the acute phase of COVID-19 infection. We performed the same PS stratification procedure and used Cox proportional hazard model to estimate the hazard ratio.

A. Empirical equipoise assessment

Supplement Figure 133: Preference score distributions of COVID-19 positive and negative groups within AKI subgroup. A greater convergence of these distributions indicates a higher similarity in the predicted likelihood of being infected between the COVID-19 positive (red) and negative (blue) participants.

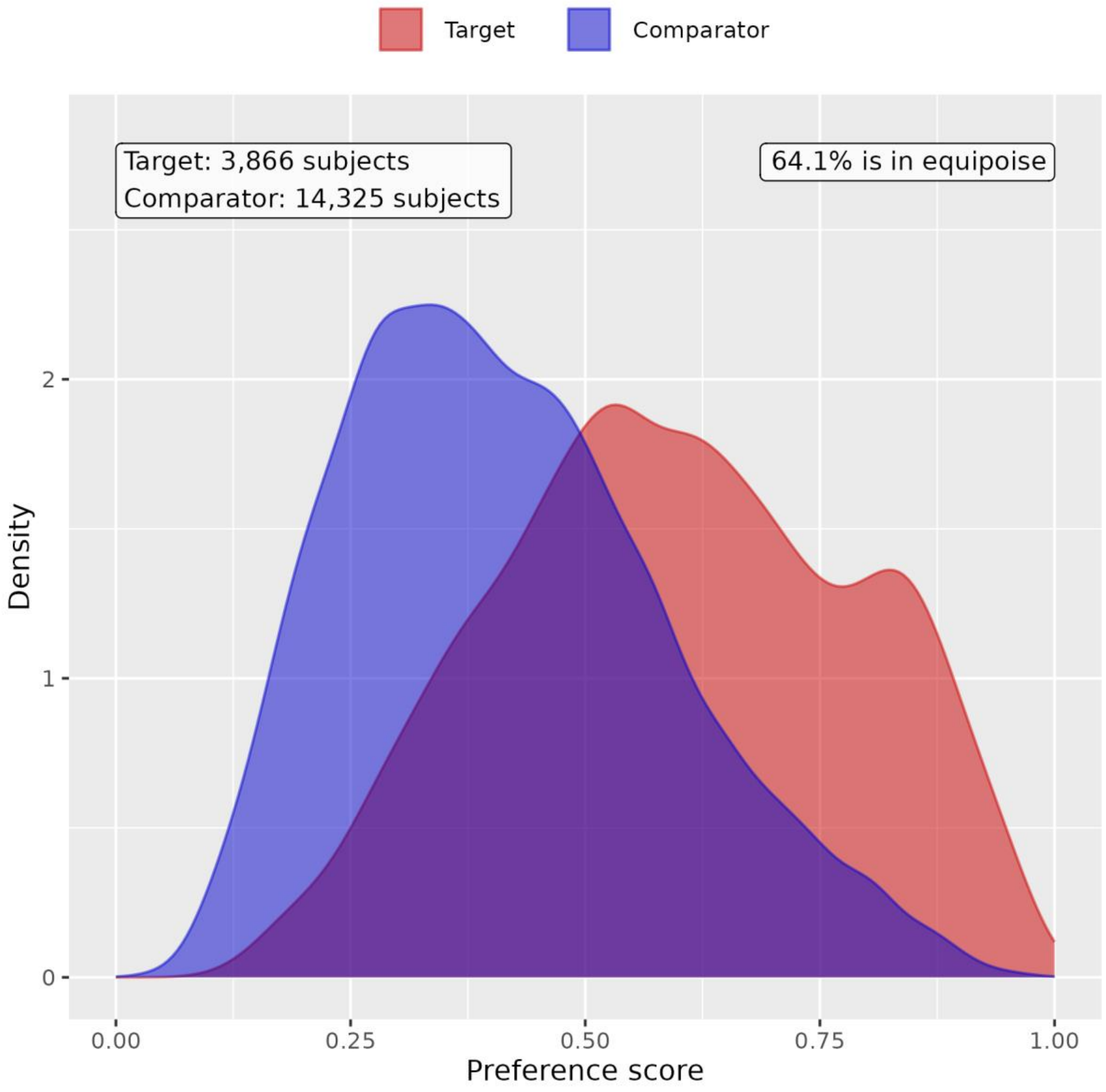

B. Patient characteristic balance

Supplement Figure 134: Patient characteristic balance before and after large-scale PS stratification with 6 strata with AKI group. The upper panel displays the top 20 covariates with the largest standardized difference of means before stratification, while the lower panel displays the top 20 covariates with the largest standardized difference of means after stratification.

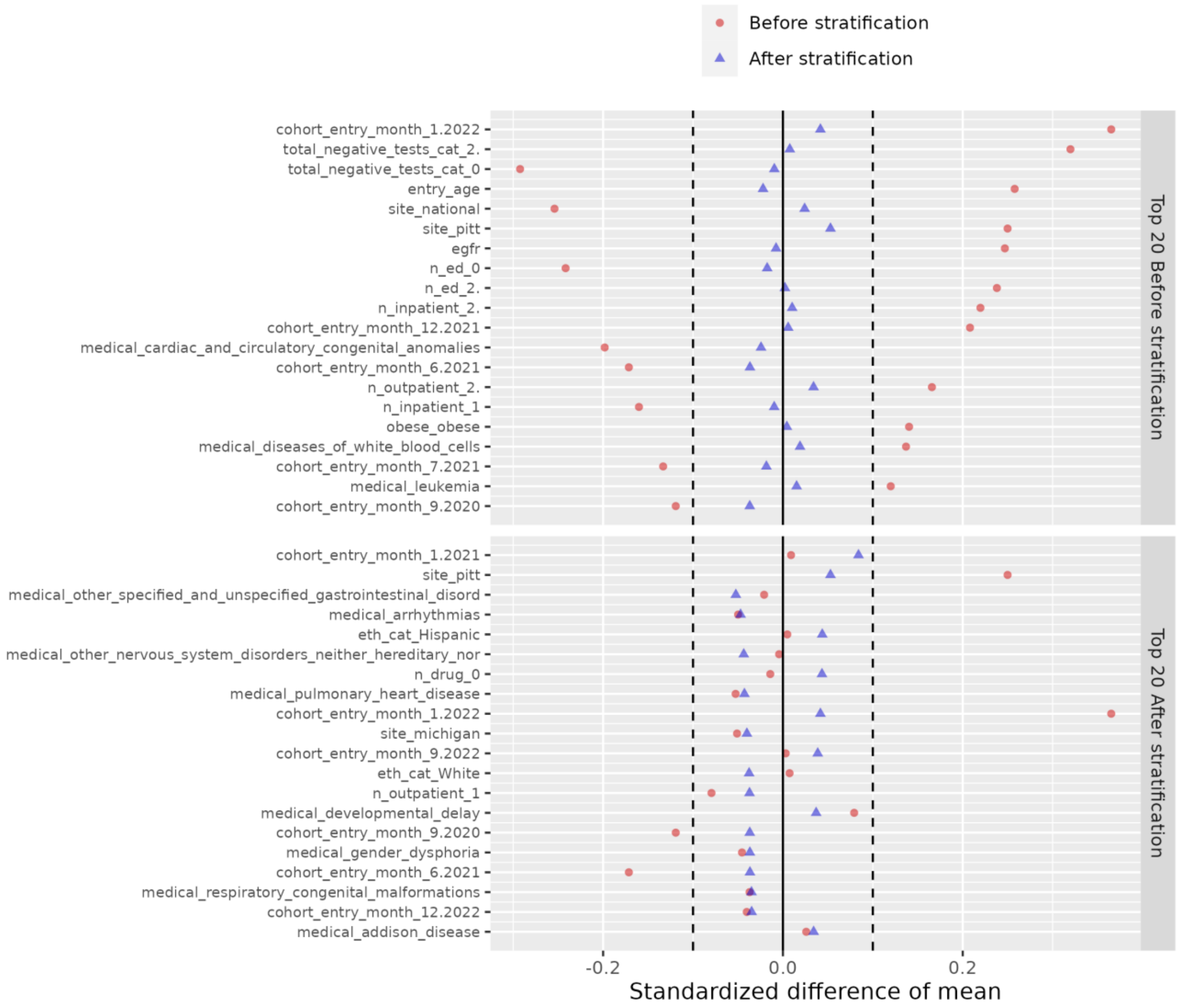

C. Hazard ratio of COVID-19 positive group compared to control group

Supplement Table 23: Estimated hazard ratio in kidney function outcomes between the COVID-19 positive cohort and the control cohort for AKI group.

AKI (not excluding patients with dialysis during acute phase)	COVID-19 Groups Event (%)	Control Group Event (%)	HR (95% CI)
AKI			
Composite outcome (days 90-179)	206 (5.33%)	484 (3.38%)	1.27 [1.15, 1.40]
Composite outcome (days 180-729)	371 (9.6%)	804 (5.61%)	1.32 [1.19, 1.46]
eGFR decline of 50% or more (days 90-179)	88 (2.28%)	195 (1.36%)	1.44 [1.09, 1.92]
eGFR decline of 50% or more (days 180-729)	162 (4.19%)	315 (2.2%)	1.40 [1.29, 1.51]
eGFR decline of 40% or more (days 90-179)	129 (3.34%)	317 (2.21%)	1.22 [1.06, 1.41]
eGFR decline of 40% or more (days 180-729)	256 (6.62%)	523 (3.65%)	1.38 [1.20, 1.59]
eGFR decline of 30% or more (days 90-179)	203 (5.25%)	484 (3.38%)	1.25 [1.14, 1.37]
eGFR decline of 30% or more (days 180-729)	366 (9.47%)	804 (5.61%)	1.30 [1.18, 1.42]
CKD 2+ (days 28-729)	194 (5.02%)	513 (3.58%)	1.15 [1.04, 1.27]
CKD 2+ (majority not returned to 90 and above) (days 28-729)	175 (4.53%)	450 (3.14%)	1.14 [1.01, 1.28]
CKD 2+ (not returned to 90 and above) (days 28-729)	159 (4.11%)	408 (2.85%)	1.12 [0.97, 1.30]
CKD 3+ (days 28-729)	62 (1.6%)	124 (0.87%)	1.54 [1.01, 2.35]
CKD 3+ (majority not returned to 90 and above) (days 28-729)	53 (1.37%)	96 (0.67%)	1.64 [1.01, 2.65]
CKD 3+ (not returned to 90 and above) (days 28-729)	59 (1.53%)	111 (0.77%)	1.59 [1.06, 2.40]
CKD 3+ (not returned to 60 and above) (days 28-729)	50 (1.29%)	90 (0.63%)	1.61 [0.98, 2.65]

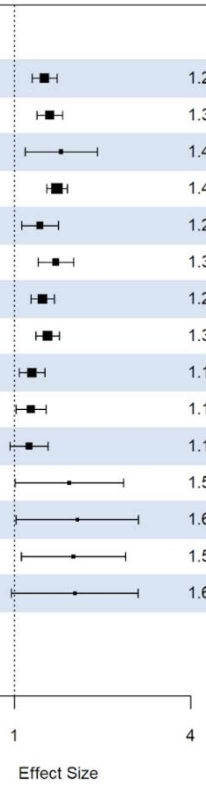

eAppendix 12. Sensitivity Analysis for AKI Subgroup Using the Lowest Creatinine Measurement Within 90 Days Before the Index Date as the Baseline

We conducted sensitivity analyses for the AKI subgroup using the lowest creatinine measurement within 90 days before the index date as the baseline. We performed the same PS stratification procedure and used Cox proportional hazard model to estimate the hazard ratio.

A. Empirical equipoise assessment

Supplement Figure 135: Preference score distributions of COVID-19 positive and negative groups within AKI subgroup. A greater convergence of these distributions indicates a higher similarity in the predicted likelihood of being infected between the COVID-19 positive (red) and negative (blue) participants.

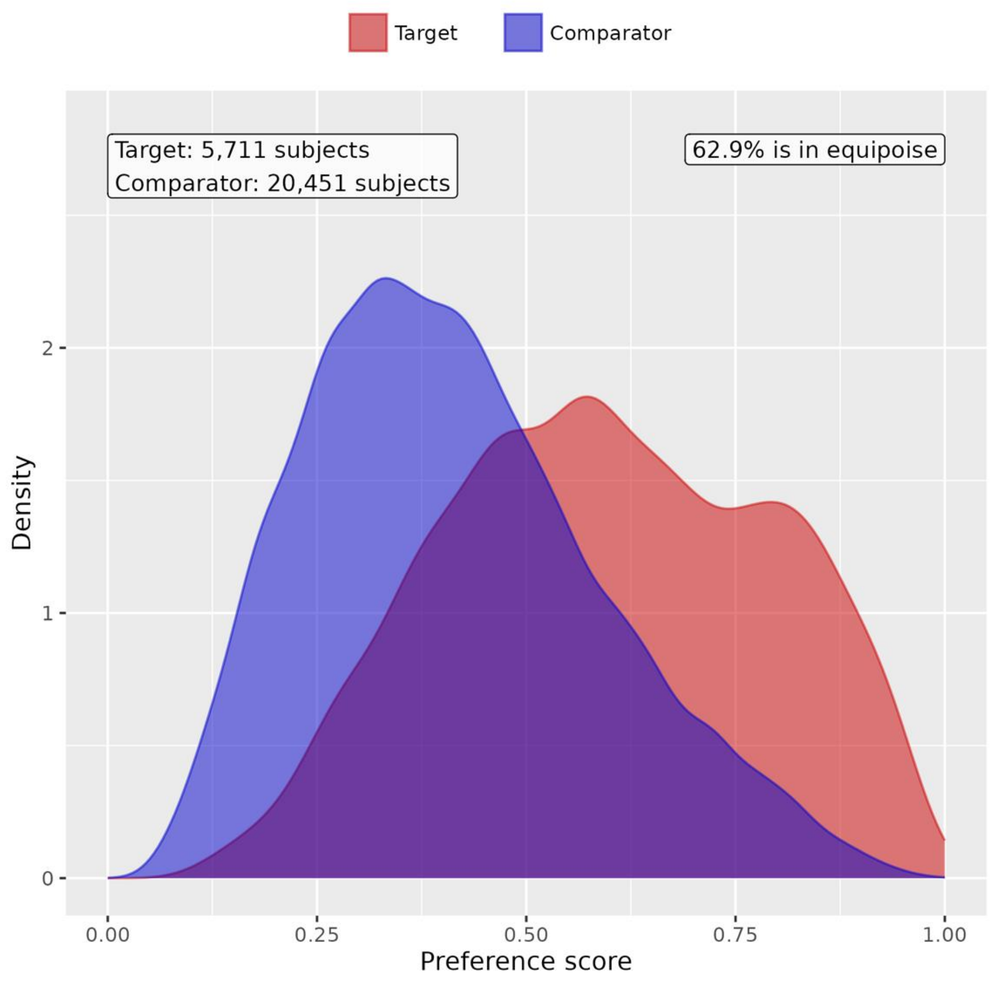

B. Patient characteristic balance

Supplement Figure 136: Patient characteristic balance before and after large-scale PS stratification with 6 strata with AKI group. The upper panel displays the top 20 covariates with the largest standardized difference of means before stratification, while the lower panel displays the top 20 covariates with the largest standardized difference of means after stratification.

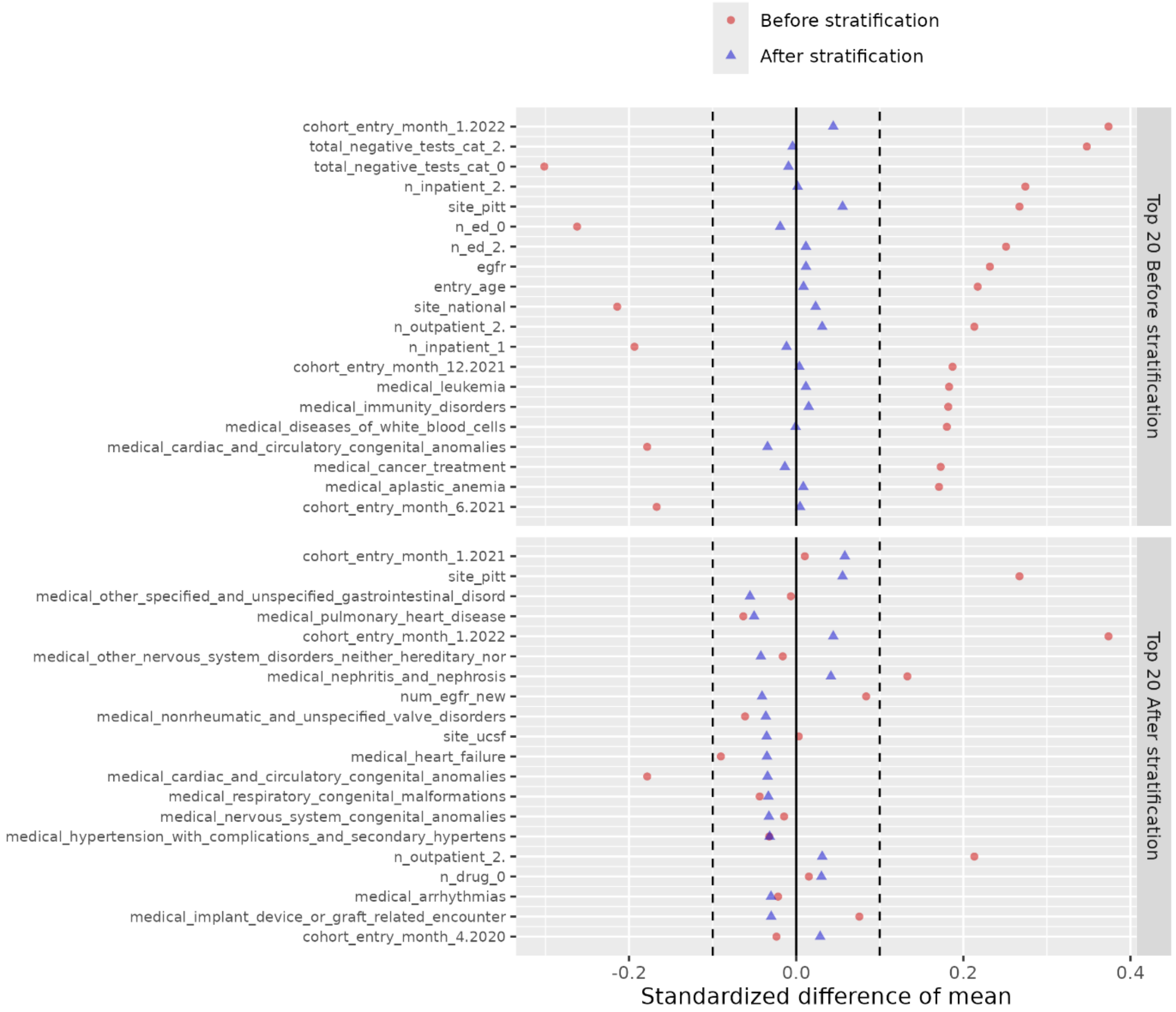

C. Hazard ratio of COVID-19 positive group compared to control group

Supplement Table 24: Estimated hazard ratio in kidney function outcomes between the COVID-19 positive cohort and the control cohort for AKI group.

AKI using lowest creatinine as baseline	COVID-19 Groups Event (%)	Control Group Event (%)	HR (95% CI)
AKI			
Composite outcome (days 90-179)	326 (5.71%)	812 (3.97%)	1.13 [1.05, 1.22]
Composite outcome (days 180-729)	582 (10.19%)	1248 (6.1%)	1.37 [1.26, 1.50]
eGFR decline of 50% or more (days 90-179)	121 (2.12%)	290 (1.42%)	1.23 [0.99, 1.52]
eGFR decline of 50% or more (days 180-729)	224 (3.92%)	435 (2.13%)	1.41 [1.28, 1.56]
eGFR decline of 40% or more (days 90-179)	189 (3.31%)	480 (2.35%)	1.10 [0.98, 1.24]
eGFR decline of 40% or more (days 180-729)	367 (6.43%)	732 (3.58%)	1.42 [1.33, 1.51]
eGFR decline of 30% or more (days 90-179)	316 (5.53%)	812 (3.97%)	1.10 [1.01, 1.20]
eGFR decline of 30% or more (days 180-729)	572 (10.02%)	1248 (6.1%)	1.35 [1.25, 1.46]
CKD 2+ (days 28-729)	262 (4.59%)	772 (3.77%)	1.09 [1.00, 1.20]
CKD 2+ (majority not returned to 90 and above) (days 28-729)	225 (3.94%)	634 (3.1%)	1.11 [1.02, 1.21]
CKD 2+ (not returned to 90 and above) (days 28-729)	196 (3.43%)	534 (2.61%)	1.14 [1.04, 1.24]
CKD 3+ (days 28-729)	79 (1.38%)	162 (0.79%)	1.59 [1.32, 1.93]
CKD 3+ (majority not returned to 90 and above) (days 28-729)	67 (1.17%)	115 (0.56%)	1.86 [1.50, 2.30]
CKD 3+ (not returned to 90 and above) (days 28-729)	71 (1.24%)	135 (0.66%)	1.72 [1.37, 2.15]
CKD 3+ (not returned to 60 and above) (days 28-729)	60 (1.05%)	104 (0.51%)	1.81 [1.44, 2.27]

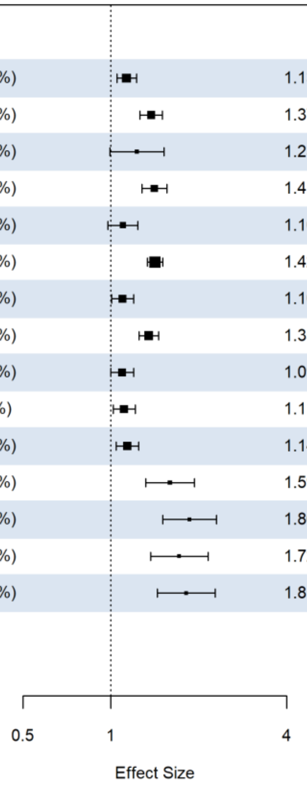

eAppendix 13. Sensitivity Analysis for CKD Subgroup Without Excluding Patients With ESKD During Baseline

We conducted sensitivity analyses for the CKD subgroup without excluding the patients who had diagnosis of ESKD during baseline. We performed the same PS stratification procedure and used Cox proportional hazard model to estimate the hazard ratio.

A. Empirical equipoise assessment

Supplement Figure 137: Preference score distributions of COVID-19 positive and negative groups within AKI subgroup. A greater convergence of these distributions indicates a higher similarity in the predicted likelihood of being infected between the COVID-19 positive (red) and negative (blue) participants.

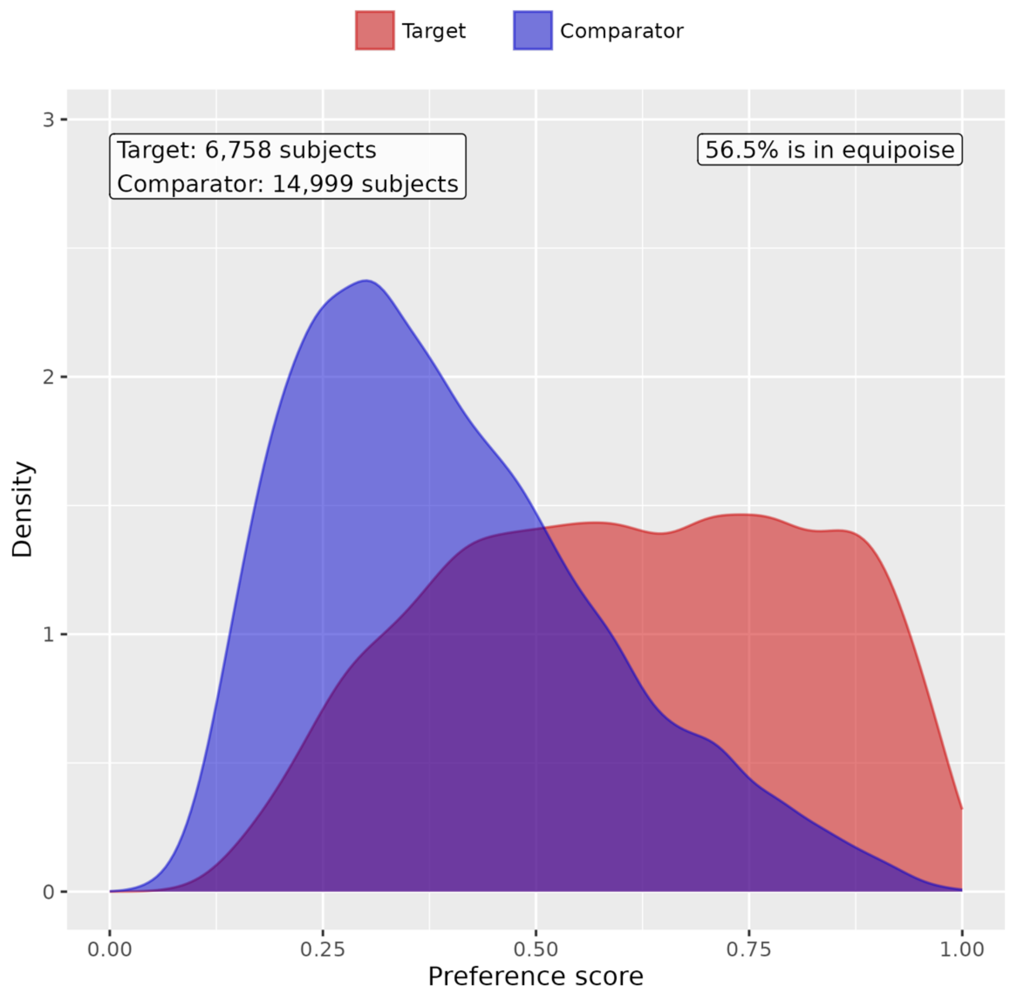

B. Patient characteristic balance

Supplement Figure 138: Patient characteristic balance before and after large-scale PS stratification with 6 strata with AKI group. The upper panel displays the top 20 covariates with the largest standardized difference of means before stratification, while the lower panel displays the top 20 covariates with the largest standardized difference of means after stratification.

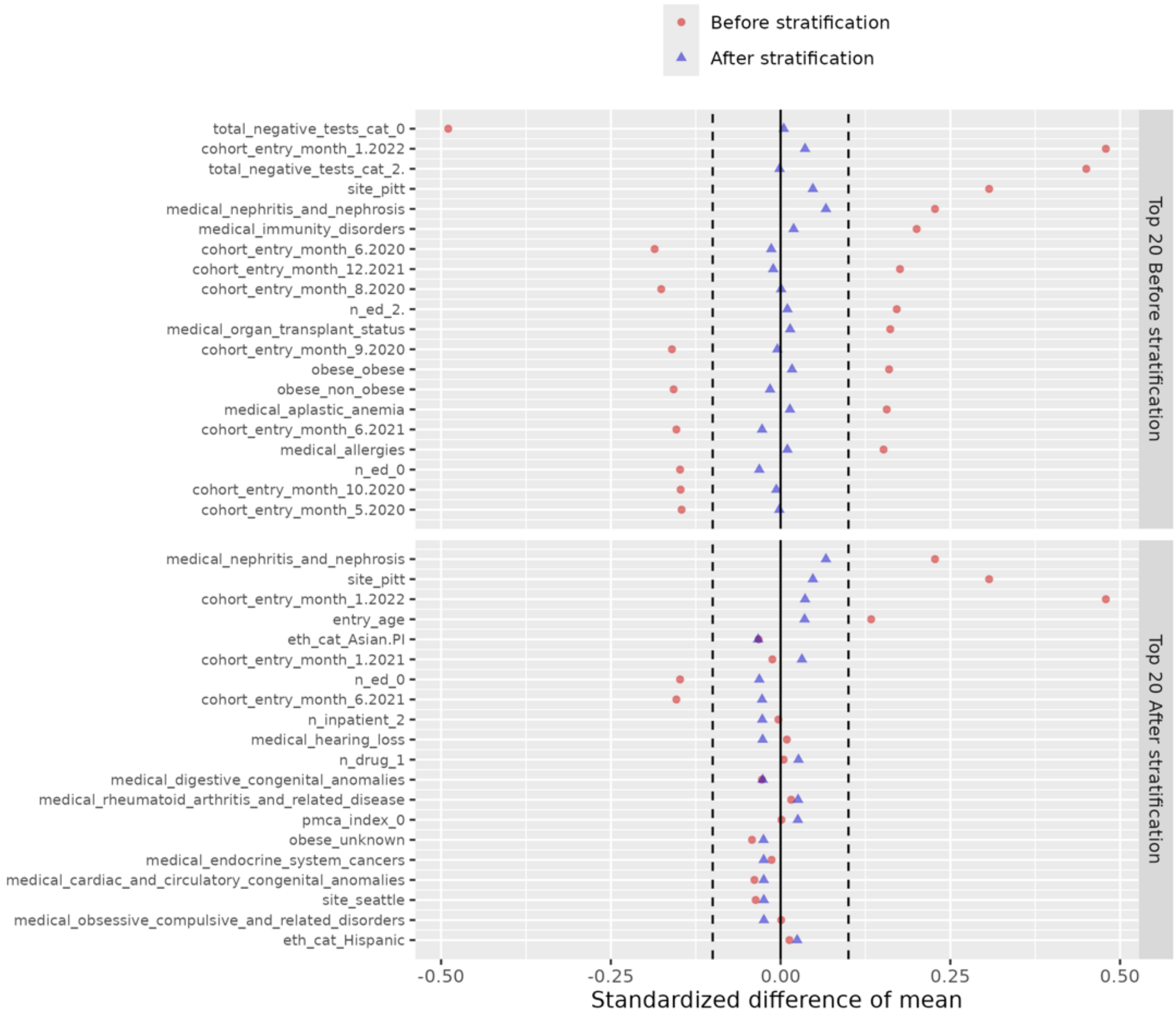

C. Hazard ratio of COVID-19 positive group compared to control group

Supplement Table 23: Estimated hazard ratio in kidney function outcomes between the COVID-19 positive cohort and the control cohort for AKI group.

CDK without excluding ESKD	COVID-19 Groups Event (%)	Control Group Event (%)	HR (95% CI)
Composite outcome (days 28-179)	985 (14.58%)	1746 (11.64%)	1.18 [1.09, 1.27]
Composite outcome (days 180-729)	1251 (18.51%)	2516 (16.77%)	1.15 [1.07, 1.25]
eGFR decline of 50% or more (days 28-179)	224 (3.31%)	405 (2.7%)	1.04 [0.84, 1.29]
eGFR decline of 50% or more (days 180-729)	292 (4.32%)	575 (3.83%)	1.14 [0.94, 1.39]
eGFR decline of 40% or more (days 28-179)	539 (7.98%)	957 (6.38%)	1.09 [0.99, 1.21]
eGFR decline of 40% or more (days 180-729)	679 (10.05%)	1345 (8.97%)	1.15 [0.99, 1.34]
eGFR decline of 30% or more (days 28-179)	971 (14.37%)	1746 (11.64%)	1.16 [1.07, 1.25]
eGFR decline of 30% or more (days 180-729)	1241 (18.36%)	2516 (16.77%)	1.14 [1.06, 1.24]

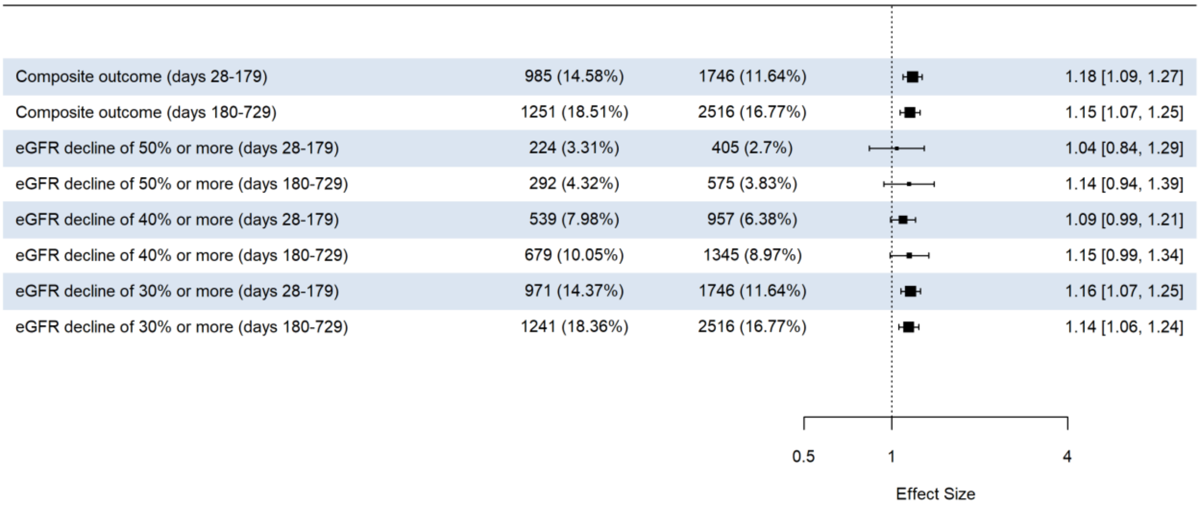

eAppendix 14. More Detailed Version of Table 1 Including Site Information and Detailed Breakdown of Age Distribution

	COVID-19 Negative				*COVID-19 Positive*			
	**No AKI or CKD (N=1383464)**	**CKD (N=14999)**	**AKI (N=14305)**	**Overall (N=1412768)**	**No AKI or CKD (N=477031)**	**CKD (N=6483)**	**AKI (N=3864)**	**Overall (N=487378)**
**Age at Cohort Entry, year**								
**0**	129940 (9.4%)	311 (2.1%)	2714 (19.0%)	132965 (9.4%)	50881 (10.7%)	78 (1.2%)	394 (10.2%)	51353 (10.5%)
**1**	121994 (8.8%)	382 (2.5%)	975 (6.8%)	123351 (8.7%)	36658 (7.7%)	186 (2.9%)	263 (6.8%)	37107 (7.6%)
**2**	96371 (7.0%)	447 (3.0%)	789 (5.5%)	97607 (6.9%)	27017 (5.7%)	184 (2.8%)	200 (5.2%)	27401 (5.6%)
**3**	88968 (6.4%)	403 (2.7%)	626 (4.4%)	89997 (6.4%)	23621 (5.0%)	146 (2.3%)	174 (4.5%)	23941 (4.9%)
**4**	84248 (6.1%)	456 (3.0%)	465 (3.3%)	85169 (6.0%)	22657 (4.7%)	156 (2.4%)	126 (3.3%)	22939 (4.7%)
**5**	81769 (5.9%)	488 (3.3%)	480 (3.4%)	82737 (5.9%)	20644 (4.3%)	150 (2.3%)	120 (3.1%)	20914 (4.3%)
**6**	72583 (5.2%)	434 (2.9%)	472 (3.3%)	73489 (5.2%)	19689 (4.1%)	146 (2.3%)	119 (3.1%)	19954 (4.1%)
**7**	64123 (4.6%)	405 (2.7%)	393 (2.7%)	64921 (4.6%)	19318 (4.0%)	137 (2.1%)	97 (2.5%)	19552 (4.0%)
**8**	58493 (4.2%)	431 (2.9%)	356 (2.5%)	59280 (4.2%)	19061 (4.0%)	148 (2.3%)	102 (2.6%)	19311 (4.0%)
**9**	54729 (4.0%)	432 (2.9%)	366 (2.6%)	55527 (3.9%)	19486 (4.1%)	157 (2.4%)	102 (2.6%)	19745 (4.1%)
**10**	51905 (3.8%)	493 (3.3%)	392 (2.7%)	52790 (3.7%)	20048 (4.2%)	197 (3.0%)	106 (2.7%)	20351 (4.2%)
**11**	51140 (3.7%)	490 (3.3%)	360 (2.5%)	51990 (3.7%)	21013 (4.4%)	203 (3.1%)	76 (2.0%)	21292 (4.4%)
**12**	50758 (3.7%)	578 (3.9%)	444 (3.1%)	51780 (3.7%)	19274 (4.0%)	215 (3.3%)	108 (2.8%)	19597 (4.0%)
**13**	51428 (3.7%)	661 (4.4%)	577 (4.0%)	52666 (3.7%)	20333 (4.3%)	265 (4.1%)	130 (3.4%)	20728 (4.3%)
**14**	52472 (3.8%)	826 (5.5%)	635 (4.4%)	53933 (3.8%)	21398 (4.5%)	366 (5.6%)	180 (4.7%)	21944 (4.5%)
**15**	53378 (3.9%)	1099 (7.3%)	760 (5.3%)	55237 (3.9%)	22009 (4.6%)	499 (7.7%)	235 (6.1%)	22743 (4.7%)
**16**	53213 (3.8%)	1409 (9.4%)	812 (5.7%)	55434 (3.9%)	22286 (4.7%)	608 (9.4%)	247 (6.4%)	23141 (4.7%)
**17**	52051 (3.8%)	1543 (10.3%)	919 (6.4%)	54513 (3.9%)	21841 (4.6%)	732 (11.3%)	300 (7.8%)	22873 (4.7%)
**18**	42630 (3.1%)	1335 (8.9%)	644 (4.5%)	44609 (3.2%)	17323 (3.6%)	641 (9.9%)	218 (5.6%)	18182 (3.7%)
**19**	37173 (2.7%)	1135 (7.6%)	581 (4.1%)	38889 (2.8%)	16653 (3.5%)	604 (9.3%)	295 (7.6%)	17552 (3.6%)
**20**	34098 (2.5%)	1241 (8.3%)	545 (3.8%)	35884 (2.5%)	15821 (3.3%)	665 (10.3%)	272 (7.0%)	16758 (3.4%)
**Sex**								
**Female**	671071 (48.5%)	8963 (59.8%)	6477 (45.3%)	686511 (48.6%)	237969 (49.9%)	4052 (62.5%)	1677 (43.4%)	243698 (50.0%)
**Male**	712393 (51.5%)	6036 (40.2%)	7828 (54.7%)	726257 (51.4%)	239062 (50.1%)	2431 (37.5%)	2187 (56.6%)	243680 (50.0%)
**Race/ Ethnicity**								
**Asian Americans and Pacific Islanders**	67455 (4.9%)	554 (3.7%)	624 (4.4%)	68633 (4.9%)	22661 (4.8%)	193 (3.0%)	141 (3.6%)	22995 (4.7%)
**Non-Hispanic Black**	234968 (17.0%)	3058 (20.4%)	4045 (28.3%)	242071 (17.1%)	83295 (17.5%)	1478 (22.8%)	1158 (30.0%)	85931 (17.6%)
**Hispanic**	285725 (20.7%)	2234 (14.9%)	2673 (18.7%)	290632 (20.6%)	106979 (22.4%)	969 (14.9%)	727 (18.8%)	108675 (22.3%)
**Multiple**	33778 (2.4%)	252 (1.7%)	164 (1.1%)	34194 (2.4%)	9479 (2.0%)	79 (1.2%)	38 (1.0%)	9596 (2.0%)
**Other/Unknown**	135917 (9.8%)	810 (5.4%)	1074 (7.5%)	137801 (9.8%)	46371 (9.7%)	286 (4.4%)	238 (6.2%)	46895 (9.6%)
**Non-Hispanic White**	625621 (45.2%)	8091 (53.9%)	5725 (40.0%)	639437 (45.3%)	208246 (43.7%)	3478 (53.6%)	1562 (40.4%)	213286 (43.8%)
**Site**								

**A**	109645 (7.9%)	633 (4.2%)	449 (3.1%)	110727 (7.8%)	32798 (6.9%)	272 (4.2%)	140 (3.6%)	33210 (6.8%)
**B**	151321 (10.9%)	1262 (8.4%)	779 (5.4%)	153362 (10.9%)	55206 (11.6%)	468 (7.2%)	187 (4.8%)	55861 (11.5%)
**C**	84313 (6.1%)	816 (5.4%)	456 (3.2%)	85585 (6.1%)	18541 (3.9%)	227 (3.5%)	120 (3.1%)	18888 (3.9%)
**D**	44458 (3.2%)	879 (5.9%)	467 (3.3%)	45804 (3.2%)	16023 (3.4%)	377 (5.8%)	131 (3.4%)	16531 (3.4%)
**E**	421 (0.0%)	3 (0.0%)	5 (0.0%)	429 (0.0%)	95 (0.0%)	0 (0%)	1 (0.0%)	96 (0.0%)
**F**	29699 (2.1%)	273 (1.8%)	179 (1.3%)	30151 (2.1%)	14737 (3.1%)	140 (2.2%)	68 (1.8%)	14945 (3.1%)
**G**	530 (0.0%)	26 (0.2%)	6 (0.0%)	562 (0.0%)	110 (0.0%)	2 (0.0%)	1 (0.0%)	113 (0.0%)
**H**	42704 (3.1%)	439 (2.9%)	320 (2.2%)	43463 (3.1%)	8618 (1.8%)	169 (2.6%)	77 (2.0%)	8864 (1.8%)
**I**	15476 (1.1%)	141 (0.9%)	60 (0.4%)	15677 (1.1%)	7593 (1.6%)	76 (1.2%)	43 (1.1%)	7712 (1.6%)
**J**	59857 (4.3%)	1204 (8.0%)	498 (3.5%)	61559 (4.4%)	14766 (3.1%)	381 (5.9%)	101 (2.6%)	15248 (3.1%)
**K**	19641 (1.4%)	236 (1.6%)	113 (0.8%)	19990 (1.4%)	8605 (1.8%)	180 (2.8%)	53 (1.4%)	8838 (1.8%)
**L**	36718 (2.7%)	271 (1.8%)	4 (0.0%)	36993 (2.6%)	9766 (2.0%)	61 (0.9%)	3 (0.1%)	9830 (2.0%)
**M**	19207 (1.4%)	809 (5.4%)	155 (1.1%)	20171 (1.4%)	6815 (1.4%)	260 (4.0%)	50 (1.3%)	7125 (1.5%)
**N**	98646 (7.1%)	55 (0.4%)	5852 (40.9%)	104553 (7.4%)	41773 (8.8%)	31 (0.5%)	1115 (28.9%)	42919 (8.8%)
**O**	122426 (8.8%)	668 (4.5%)	487 (3.4%)	123581 (8.7%)	27068 (5.7%)	206 (3.2%)	167 (4.3%)	27441 (5.6%)
**P**	6066 (0.4%)	133 (0.9%)	79 (0.6%)	6278 (0.4%)	2868 (0.6%)	68 (1.0%)	26 (0.7%)	2962 (0.6%)
**Q**	97556 (7.1%)	1014 (6.8%)	358 (2.5%)	98928 (7.0%)	25771 (5.4%)	310 (4.8%)	131 (3.4%)	26212 (5.4%)
**R**	51734 (3.7%)	684 (4.6%)	253 (1.8%)	52671 (3.7%)	20695 (4.3%)	385 (5.9%)	80 (2.1%)	21160 (4.3%)
**S**	35663 (2.6%)	1165 (7.8%)	442 (3.1%)	37270 (2.6%)	10139 (2.1%)	435 (6.7%)	132 (3.4%)	10706 (2.2%)
**T**	135538 (9.8%)	179 (1.2%)	68 (0.5%)	135785 (9.6%)	63376 (13.3%)	127 (2.0%)	31 (0.8%)	63534 (13.0%)
**U**	2318 (0.2%)	1 (0.0%)	0 (0%)	2319 (0.2%)	857 (0.2%)	1 (0.0%)	0 (0%)	858 (0.2%)
**V**	15410 (1.1%)	164 (1.1%)	120 (0.8%)	15694 (1.1%)	27489 (5.8%)	481 (7.4%)	194 (5.0%)	28164 (5.8%)
**W**	30144 (2.2%)	211 (1.4%)	144 (1.0%)	30499 (2.2%)	4963 (1.0%)	68 (1.0%)	52 (1.3%)	5083 (1.0%)
**X**	43830 (3.2%)	206 (1.4%)	349 (2.4%)	44385 (3.1%)	13302 (2.8%)	93 (1.4%)	74 (1.9%)	13469 (2.8%)
**Y**	45210 (3.3%)	453 (3.0%)	338 (2.4%)	46001 (3.3%)	12945 (2.7%)	161 (2.5%)	101 (2.6%)	13207 (2.7%)
**Z**	35747 (2.6%)	2243 (15.0%)	1860 (13.0%)	39850 (2.8%)	13197 (2.8%)	1106 (17.1%)	629 (16.3%)	14932 (3.1%)
**AA**	32692 (2.4%)	600 (4.0%)	166 (1.2%)	33458 (2.4%)	12870 (2.7%)	330 (5.1%)	67 (1.7%)	13267 (2.7%)
**BB**	16494 (1.2%)	231 (1.5%)	298 (2.1%)	17023 (1.2%)	6045 (1.3%)	68 (1.0%)	90 (2.3%)	6203 (1.3%)
**Cohort Entry Period**								
**03/2020 - 05/2020**	25471 (1.8%)	594 (4.0%)	436 (3.0%)	26501 (1.9%)	4778 (1.0%)	96 (1.5%)	114 (3.0%)	4988 (1.0%)
**03/2021 - 05/2021**	137964 (10.0%)	1497 (10.0%)	1534 (10.7%)	140995 (10.0%)	28730 (6.0%)	448 (6.9%)	280 (7.2%)	29458 (6.0%)
**03/2022 - 05/2022**	114323 (8.3%)	1110 (7.4%)	1401 (9.8%)	116834 (8.3%)	36985 (7.8%)	509 (7.9%)	337 (8.7%)	37831 (7.8%)
**06/2020 - 08/2020**	96457 (7.0%)	1731 (11.5%)	938 (6.6%)	99126 (7.0%)	17368 (3.6%)	248 (3.8%)	169 (4.4%)	17785 (3.6%)
**06/2021 - 08/2021**	135823 (9.8%)	1431 (9.5%)	1477 (10.3%)	138731 (9.8%)	26162 (5.5%)	409 (6.3%)	268 (6.9%)	26839 (5.5%)
**06/2022 - 08/2022**	83688 (6.0%)	958 (6.4%)	1336 (9.3%)	85982 (6.1%)	51659 (10.8%)	757 (11.7%)	456 (11.8%)	52872 (10.8%)
**09/2020 - 11/2020**	140935 (10.2%)	1798 (12.0%)	1506 (10.5%)	144239 (10.2%)	32030 (6.7%)	408 (6.3%)	224 (5.8%)	32662 (6.7%)
**09/2021 - 11/2021**	217656 (15.7%)	1645 (11.0%)	1556 (10.9%)	220857 (15.6%)	49458 (10.4%)	630 (9.7%)	409 (10.6%)	50497 (10.4%)
**09/2022 - 12/2022**	132831 (9.6%)	1216 (8.1%)	1393 (9.7%)	135440 (9.6%)	26934 (5.6%)	422 (6.5%)	272 (7.0%)	27628 (5.7%)
**12/2020 - 02/2021**	135978 (9.8%)	1673 (11.2%)	1341 (9.4%)	138992 (9.8%)	51601 (10.8%)	665 (10.3%)	356 (9.2%)	52622 (10.8%)

**12/2021 - 02/2022**	162338 (11.7%)	1346 (9.0%)	1387 (9.7%)	165071 (11.7%)	151326 (31.7%)	1891 (29.2%)	979 (25.3%)	154196 (31.6%)
**Obesity**								
**Non-Obese**	722415 (52.2%)	7439 (49.6%)	7852 (54.9%)	737706 (52.2%)	214869 (45.0%)	2723 (42.0%)	1910 (49.4%)	219502 (45.0%)
**Obese**	497849 (36.0%)	7541 (50.3%)	4836 (33.8%)	510226 (36.1%)	206568 (43.3%)	3759 (58.0%)	1567 (40.6%)	211894 (43.5%)
**Unknown**	163200 (11.8%)	19 (0.1%)	1617 (11.3%)	164836 (11.7%)	55594 (11.7%)	1 (0.0%)	387 (10.0%)	55982 (11.5%)
**Chronic Disease Status**								
**0**	1006146 (72.7%)	2840 (18.9%)	6272 (43.8%)	1015258 (71.9%)	351288 (73.6%)	1283 (19.8%)	1641 (42.5%)	354212 (72.7%)
**1**	220119 (15.9%)	3280 (21.9%)	2400 (16.8%)	225799 (16.0%)	75835 (15.9%)	1403 (21.6%)	585 (15.1%)	77823 (16.0%)
**2**	157199 (11.4%)	8879 (59.2%)	5633 (39.4%)	171711 (12.2%)	49908 (10.5%)	3797 (58.6%)	1638 (42.4%)	55343 (11.4%)
**Number of Tests**								
**0**	1049258 (75.8%)	9026 (60.2%)	9148 (63.9%)	1067432 (75.6%)	291958 (61.2%)	2415 (37.3%)	1916 (49.6%)	296289 (60.8%)
**1**	218747 (15.8%)	2818 (18.8%)	2534 (17.7%)	224099 (15.9%)	101336 (21.2%)	1459 (22.5%)	709 (18.3%)	103504 (21.2%)
**2**	66084 (4.8%)	1316 (8.8%)	1059 (7.4%)	68459 (4.8%)	41027 (8.6%)	844 (13.0%)	360 (9.3%)	42231 (8.7%)
**>2**	49375 (3.6%)	1839 (12.3%)	1564 (10.9%)	52778 (3.7%)	42710 (9.0%)	1765 (27.2%)	879 (22.7%)	45354 (9.3%)
**Number of Vaccine Doses**								
**0**	1248073 (90.2%)	12690 (84.6%)	13165 (92.0%)	1273928 (90.2%)	416466 (87.3%)	5189 (80.0%)	3399 (88.0%)	425054 (87.2%)
**1**	25661 (1.9%)	417 (2.8%)	260 (1.8%)	26338 (1.9%)	12185 (2.6%)	288 (4.4%)	135 (3.5%)	12608 (2.6%)
**>=2**	109730 (7.9%)	1892 (12.6%)	880 (6.2%)	112502 (8.0%)	48380 (10.1%)	1006 (15.5%)	330 (8.5%)	49716 (10.2%)
**Number of Drugs**								
**0**	379837 (27.5%)	355 (2.4%)	2029 (14.2%)	382221 (27.1%)	117622 (24.7%)	124 (1.9%)	529 (13.7%)	118275 (24.3%)
**1**	173275 (12.5%)	393 (2.6%)	1040 (7.3%)	174708 (12.4%)	59593 (12.5%)	182 (2.8%)	258 (6.7%)	60033 (12.3%)
**2**	136222 (9.8%)	490 (3.3%)	898 (6.3%)	137610 (9.7%)	49144 (10.3%)	181 (2.8%)	228 (5.9%)	49553 (10.2%)
**>2**	694130 (50.2%)	13761 (91.7%)	10338 (72.3%)	718229 (50.8%)	250672 (52.5%)	5996 (92.5%)	2849 (73.7%)	259517 (53.2%)

eAppendix 15. More Detailed Version of Figure 1, Including Variations of Definitions of CKD Stage 2 or Higher and CKD Stage 3 or Higher

Supplemental Table 24. Adjusted hazard ratios for kidney outcomes in COVID-19 Positive vs. Negative Patients by Phase. The kidney outcomes are defined for each subgroup of patients based on pre-existing kidney function status (acute kidney injury during acute phase of COVID-19 infection (AKI), chronic kidney disease stage 2+ (CKD), and no AKI or CKD). Majority is defined as more than 50%.

	COVID-19 Groups Event (%)	Control Group Event (%)	HR (95% CI)
No CKD or AKI			
CKD 2+ (days 28-729)	1569 (0.33%)	3900 (0.28%)	1.17 [1.12, 1.22]
CKD 2+ (majority not returned to 90 and above) (days 28-729)	1354 (0.28%)	3265 (0.24%)	1.18 [1.12, 1.25]
CKD 2+ (not returned to 90 and above) (days 28-729)	1250 (0.26%)	2909 (0.21%)	1.20 [1.13, 1.28]
CKD 3+ (days 28-729)	132 (0.03%)	309 (0.02%)	1.35 [1.13, 1.62]
CKD 3+ (majority not returned to 90 and above) (days 28-729)	98 (0.02%)	224 (0.02%)	1.39 [1.15, 1.68]
CKD 3+ (not returned to 90 and above) (days 28-729)	111 (0.02%)	271 (0.02%)	1.26 [1.08, 1.47]
CKD 3+ (not returned to 60 and above) (days 28-729)	88 (0.02%)	200 (0.01%)	1.35 [1.15, 1.59]
CKD			
Composite outcome (days 28-179)	905 (13.96%)	1746 (11.64%)	1.15 [1.04, 1.27]
Composite outcome (days 180-729)	1167 (18%)	2516 (16.77%)	1.14 [1.06, 1.22]
eGFR decline of 50% or more (days 28-179)	191 (2.95%)	405 (2.7%)	1.00 [0.89, 1.11]
eGFR decline of 50% or more (days 180-729)	246 (3.79%)	575 (3.83%)	1.09 [0.94, 1.26]
eGFR decline of 40% or more (days 28-179)	484 (7.47%)	957 (6.38%)	1.06 [0.93, 1.22]
eGFR decline of 40% or more (days 180-729)	615 (9.49%)	1345 (8.97%)	1.12 [0.98, 1.26]
eGFR decline of 30% or more (days 28-179)	894 (13.79%)	1746 (11.64%)	1.14 [1.03, 1.25]
eGFR decline of 30% or more (days 180-729)	1159 (17.88%)	2516 (16.77%)	1.13 [1.05, 1.20]
AKI			
Composite outcome (days 90-179)	206 (5.33%)	477 (3.33%)	1.29 [1.21, 1.38]
Composite outcome (days 180-729)	370 (9.58%)	796 (5.56%)	1.33 [1.21, 1.47]
eGFR decline of 50% or more (days 90-179)	88 (2.28%)	191 (1.34%)	1.47 [1.06, 2.03]
eGFR decline of 50% or more (days 180-729)	162 (4.19%)	308 (2.15%)	1.42 [1.31, 1.55]
eGFR decline of 40% or more (days 90-179)	129 (3.34%)	312 (2.18%)	1.24 [1.10, 1.41]
eGFR decline of 40% or more (days 180-729)	256 (6.63%)	516 (3.61%)	1.41 [1.21, 1.63]
eGFR decline of 30% or more (days 90-179)	203 (5.25%)	477 (3.33%)	1.27 [1.20, 1.36]
eGFR decline of 30% or more (days 180-729)	365 (9.45%)	796 (5.56%)	1.31 [1.20, 1.43]
CKD 2+ (days 28-729)	194 (5.02%)	505 (3.53%)	1.17 [1.05, 1.31]
CKD 2+ (majority not returned to 90 and above) (days 28-729)	175 (4.53%)	442 (3.09%)	1.16 [1.03, 1.31]
CKD 2+ (not returned to 90 and above) (days 28-729)	159 (4.11%)	399 (2.79%)	1.14 [1.00, 1.31]
CKD 3+ (days 28-729)	62 (1.6%)	117 (0.82%)	1.61 [1.09, 2.37]
CKD 3+ (majority not returned to 90 and above) (days 28-729)	53 (1.37%)	93 (0.65%)	1.68 [1.06, 2.66]
CKD 3+ (not returned to 90 and above) (days 28-729)	59 (1.53%)	104 (0.73%)	1.67 [1.15, 2.44]
CKD 3+ (not returned to 60 and above) (days 28-729)	50 (1.29%)	87 (0.61%)	1.65 [1.01, 2.70]

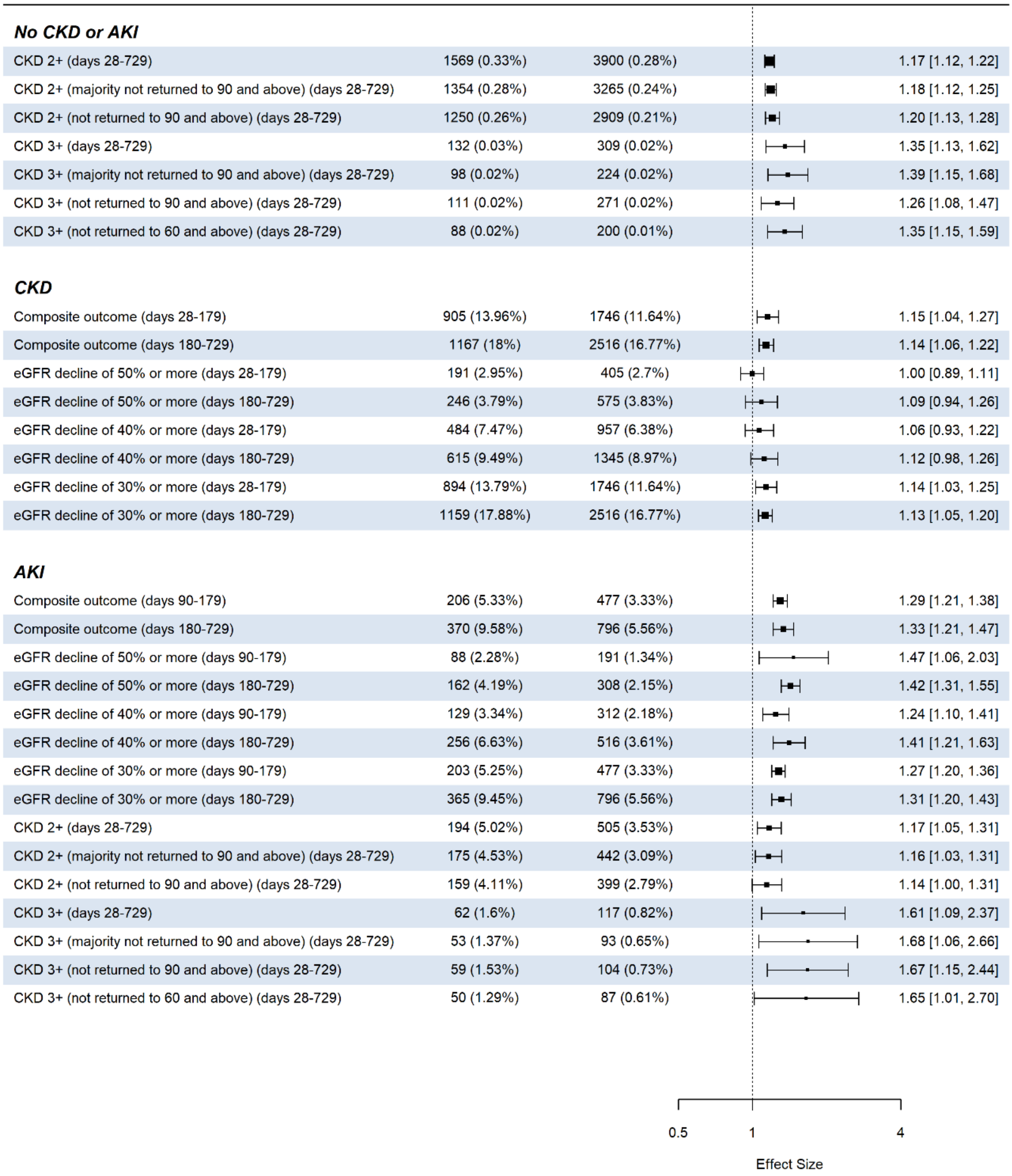

eAppendix 16. Recurrent COVID-19 Infections and Outcomes

Recurrent SARS-CoV-2 infections may influence post-acute outcomes differently than a single infection due to cumulative viral exposure, immune response variability, or differences in clinical severity. While our study primarily focuses on the association between having ≥1 COVID-19 infection and kidney outcomes, we acknowledge that recurrence could act as an effect modifier. Future studies are needed to evaluate the independent and cumulative impacts of recurrent infections, which may provide additional insights into the long-term sequelae of SARS-CoV-2 in pediatric populations.

Supplementary Table 25. Proportions of patients with no reinfection, 1 reinfection, 2 reinfections, or 3 reinfections. Reinfection was identified by a subsequent positive PCR or antigen test, clinical diagnosis, or prescription for nirmatrelvir/ritonavir, occurring at least 60 days after the previous infection. Reinfections within 60 days were considered ongoing infections.

	No reinfection	1 reinfection	2 reinfections	3 infections
**CKD**	6314 (93.58%)	428 (6.23%)	13 (0.15%)	3 (0.03%)
**No CKD or AKI**	459821 (96.39%)	16688 (3.50%)	507 (0.11%)	15 (0.00%)
**AKI**	3603 (93.25%)	248 (6.42%)	13 (0.34%)	0 (0.00%)
