# Supplementary material for: Kidney Function Following COVID-19 in Children and Adolescents
Source: JAMA Netw Open. 2025 Apr 11;8(4):e254129. doi: 10.1001/jamanetworkopen.2025.4129 (PMC11992607; doi:10.1001/jamanetworkopen.2025.4129)
Supplement: Supplement 3. — Data Sharing Statement [file jamanetwopen-e254129-s003.pdf]

Data Sharing Statement

Li. Kidney Function Following COVID-19 in Children and Adolescents. *JAMA Netw Open*. Published April 09, 2025. doi:10.1001/jamanetworkopen.2025.4129

Data
Data available: No
